# Supplementary material for: Impact of abolishing prescription fees in Scotland on hospital admissions and prescribed medicines: an interrupted time series evaluation
Source: BMJ Open. 2018 Dec 18;8(12):e021318. doi: 10.1136/bmjopen-2017-021318 (PMC6303621; doi:10.1136/bmjopen-2017-021318)
Supplement: Supplementary file 1 [file bmjopen-2017-021318supp001.pdf]

Supplementary file 1 – National Health Service Prescription Fee exemption Criteria [1]

Medical Grounds:

- Forms of hypoadrenalism (including Addison's disease) for which specific substitution therapy is essential
- Diabetes insipidus or other forms of hypopituitarism
- Diabetes mellitus (except where treatment is by diet alone)
- Hypoparathyroidism
- Myasthenia gravis
- Myxodoema (hypothyroidism requiring thyroid hormone replacement)
- Epilepsy requiring continuous anti-convulsive therapy
- A permanent fistula, requiring an appliance or continuous surgical dressing
- Continuing physical disability which prevents the patient from leaving their residence without the help of another person
- Cancer (added in 2009) including any prescriptions for current or previous cancer treatment

Pregnant or had a baby in the previous 12 months (these are included with the medical exemptions)

Contraceptives (these are included with the medical exemptions)

Age:

- Under 16 years
- 16-18 years and in full time education
- Over 60 years

Financial situation:

- Those receiving Income Support, Income-Based Job Seeker's Allowance, Income-related Employment and Support Allowance, Pension Credit Guarantee Credit, Universal Credit and people who qualify for full help under the NHS Low Income Scheme are exempt.

References

1 NHS Choices. Get help with prescription costs. London. Gov.uk, 2017. Website: https://www.nhs.uk/nhsengland/Healthcosts/pages/Prescriptioncosts.aspx (accessed 26 June 2018)
