# Supplementary material for: Impact of abolishing prescription fees in Scotland on hospital admissions and prescribed medicines: an interrupted time series evaluation
Source: BMJ Open. 2018 Dec 18;8(12):e021318. doi: 10.1136/bmjopen-2017-021318 (PMC6303621; doi:10.1136/bmjopen-2017-021318)
Supplement: Supplementary file 2 [file bmjopen-2017-021318supp002.pdf]

Farr Institute @ Scotland – NEA group / SCPHRP
Andrew James Williams

Natural Experiment

'Universal' Free Prescriptions in Scotland

eDRIS project number: ED13-0487
Primary contact: Andrew James Williams
Other team members: John Frank, Ruth Jepson, Jill Pell, Chris Dibben, Anne Ludbrook and Marion Bennie.

Background

Following devolution each of the four nations of the United Kingdom (UK) (England, Wales, Scotland and Northern Ireland) have taken different approaches to subsidising and funding prescribed medications (British Broadcasting Corporation, 2011). Only England continues to charge a prescription fee which stands at £7.85 in 2013 (Department of Health, 2013). When Scotland abolished the prescription charge the then Health Secretary, Nicola Sturgeon, described prescription fees as 'a tax on ill-health' (British Broadcasting Corporation, 2011).

For those with chronic conditions one of the purposes of pharmaceuticals is to control the condition and subsequently reduce the need for hospitalisation and more invasive and costly intervention. Purdy et al. (2009) have defined a list of conditions sensitive to ambulatory care which are listed in Appendix 1. The appropriate management and treatment of these conditions in primary care should prevent the need for admission into secondary care (Purdy et al., 2009). The success of pharmaceutical treatments is dependent upon the patient's adherence to the dosage regimen prescribed by the practitioner. Kardas et al. (2013) recently reviewed the determinants of non-adherence. Seven hundred and seventy one determinants of whether someone initiated and/or persisted with a medication were identified which fell into the following five broad clusters; socioeconomic-related, healthcare team- and system-related, condition-related, therapy-related, and patient-related (Kardas et al., 2013). Among the socioeconomic cluster the eight studies which examined cost, all found higher cost to be associated with poorer adherence (Kardas et al., 2013). Therefore, the economic logic behind the abolition of prescription fees is that adherence will improve, and therefore hospital admissions will decrease reducing secondary care costs, which hopefully compensate for the additional cost to the state of the prescriptions (Figure 1).

Previous research has tended to focus on elements of the logic model rather than the whole. Linnet et al. (2013), Yin et al. (2008) and Zhang et al. (2008) focussed upon the first half of the logic model, the effect of prescription fee policies on the dispensing of pharmaceuticals with mixed results. Yin et al. (2008) and Zhang et al. (2008) found that the introduction of prescription subsidies (Medicare part D) for the elderly in America resulted in an increased number of prescriptions and reduced out-of-pocket expenses for the patient. Whereas Linnet et al. (2013) found that within Iceland a small increase in prescription charge did not significantly affect whether a patient cashed/filled a prescription. Booth et al. (2012) when examining the socioeconomic disparities in diabetes mellitus related morbidity and mortality found that the socioeconomic disparities greatly reduced in each of the outcomes studied for those over 65 years of age (Booth et al., 2012). Within Ontario, Canada (the location of the study) those over the age of 65 receive free prescriptions and therefore the decreased socioeconomic disparities was attributed to this policy (Booth et al., 2012). Booth et al. (2012) employed a number of

statistical techniques to test this hypothesis (sensitivity analyses) and continued to find the decreased disparities associated with the free prescription policy. Campbell et al. (2011) and Kulik et al. (2013) employed more experimental study designs to test the logic model. Kulik et al. (2013) undertook a randomised controlled trial into the effect of offering free prescriptions to patients immediately following a myocardial infarction and found improved adherence and reduced patient out-of-pocket expenses and a trend towards improved clinical outcomes. Campbell et al. (2011) used linked data within a natural experimental approach to examine the effect of prescription costs for those with asthma. It was found that those experiencing increases in prescription charges (co-payment) ≥$5(US) were significantly less adherent than those experiencing increases of <$5 and also had higher healthcare usage (Campbell et al., 2011).

Figure 1 – Logic model for study

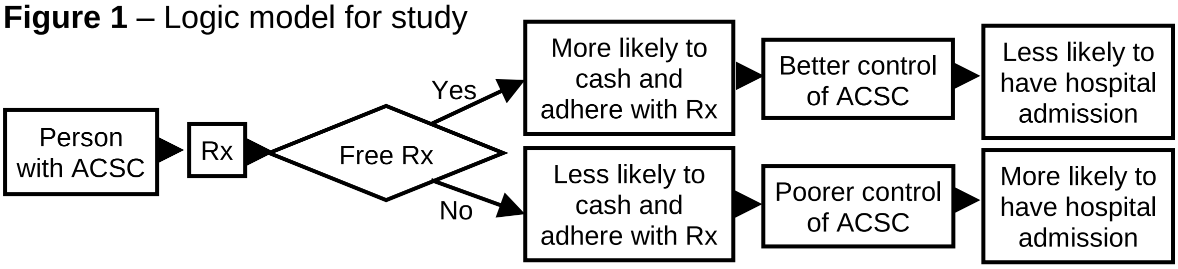

ACSC; ambulatory case sensitive condition, Rx; prescription

Subsequently, there is some evidence that reduced or free prescriptions have positive effects on adherence and health. However, the studies so far have focused upon certain age groups or conditions and yet within Wales, Northern Ireland and Scotland prescriptions are free for all. Three years after the abolition of prescription fees in Wales a process evaluation was published by the health authority (NHS Wales, 2010). This evaluation found that the abolition of prescription fees did not result in a change in the number of prescriptions and their cost to the state. Jill Pell and colleagues are undertaking a similar study examining the first half of the logic model (Figure 1) in Scotland. However, to the best of our knowledge there have been no studies within the UK to examine the whole logic model in relation to the universal free prescription policy.

Aims

To identify whether the stepped reduction and abolition of prescription fees in Scotland resulted in:

a) increase in the number (cost to NHS) of prescriptions for inhaled steroids;
b) reduction in hospital admissions for asthma or chronic obstructive pulmonary disease (COPD).

When compared to prescriptions and admissions for a condition for which prescriptions were historically free (diabetes mellitus) and or those who received free prescriptions due to age.

Moving of from these preliminary aims more refined analysis is planned to address these two further questions.

c) Was any change in hospital admissions for asthma or COPD in the practices which also saw a change in prescribing/dispensing?
d) How did the effect differ across the socioeconomic spectrum?

Farr Institute @ Scotland – NEA group / SCPHRP
Andrew James Williams

Proposal

As a policy the abolition of prescription fees was not introduced experimentally (e.g. with randomisation), and subsequently we propose to address the above aims using time series of routinely collected data as a natural experiment (Craig et al., 2011). The primary sources for the routinely collected data will be the Scottish Morbidity Record (SMR01) and the Prescriptions Information System (PIS). Through extensive discussion with the Principle Pharmacist (Stuart McTaggart) at NHS National Services Scotland, it has been established that individual-level data suitable for this project are not currently available in the PIS. The primary reasons that individual-level data from the PIS cannot be used are the inability to calculate measures of adherence (Fairman and Matheral, 2000, Lehmann et al., 2013), and the fact that currently individual-level data are not available prior to August 2009 when Scotland had already begun reducing prescription fees. Subsequently, we propose to proceed with a practice-level study as outlined below. Currently, the proposed study is limited to Scotland, although through the Farr network a UK wide study may be considered at a later date.

Critical to any natural experiment is the identification of the population who were/are effected by the policy (the intervention sample) and appropriate counterfactual sample(s) (Craig et al., 2011). As prescription fees were reduced and ultimately abolished contemporaneously across Scotland and the data being used only exist in Scotland there is no location-based contemporary counterfactual. However, the existence of prescription fee exemption criteria (Appendix 2) prior to the abolition of fees permits the identification of the intervention and counterfactual samples. Although, as following the abolition of prescription fees there was no need to record exemption criteria, the samples also had to remain identifiable within the PIS and SMR01. Furthermore, as only one exemption criteria needed to be recorded when collecting a prescription, it was and will remain necessary throughout this project to remain aware that although marked exempt for one reason the patient may have also met other exemption criteria.

The exemption criteria (Appendix 2) fall into three groups, age- condition- and income-related all of which could have formed the basis of counterfactual samples. The income-related exemptions group may have permitted a regression discontinuity design, however, it would not be possible to identify this sample following the abolition of fees and consequently no income-based counterfactual was sought. The age-related exemption criteria would form an age-based counterfactual sample. This is made possible as prior to the abolition of fees the PIS exemptions category data would identify this sample and following the fee abolition individual-level PIS data could inform the identification of this group. Those aged 16-18 years in full time education also received free prescriptions (Appendix 2); as this subpopulation can only be identified prior to the abolition of fees they will initially be examined separately to ascertain what approach is most appropriate for this subpopulation. However, wanting to strengthen the causal inference it is planned to examine dispensed prescriptions as a mediator of change in health (admissions) and consequently it is necessary to identify suitably sensitive and specific condition medication pairings. Thus, the condition-related exemption criteria were crucial. These pairings needed to meet the following requirements;

- The condition needed to be chronic (e.g. not ear, nose and throat infections).

- The condition needed to be sensitive to long term pharmaceutical treatment specifically not just ambulatory care in general (e.g. not dental complications).
- Relatively minor changes in adherence (e.g. missed tablets) to the treatment for the condition needed to result in the need for hospital attention (e.g. not schizophrenia or epilepsy).
- The condition needed to be sufficiently prevalent across the life-course to provide sufficient power for the analysis (e.g. not senility/dementia).
- The intervention condition needed to not be a condition-related exemption from prescription fees (Appendix 2) or commonly co-occurring with a condition on that list (e.g. not diabetes mellitus or any of the cardiovascular conditions).
- The counterfactual condition needed to be a condition-related exemption from prescription fees (e.g. diabetes mellitus).
- The pharmaceutical treatment (medicine) needs to be fairly specific to the condition (e.g. not congestive heart failure).

The final samples are outlined in the box below, with asthma/COPD selected as the intervention condition. Asthma/COPD had been noted for their absence from the exemption criteria in the Scottish public consultation about the abolition of prescription fees (Scottish Executive, 2006, The Scottish Parliament, n.d.). These samples have been defined after extensive consultation with expert pharmacists and general practitioners.

**Sample**	**Eligibility criteria**
Intervention	Those aged ≥16-<60 years that had paid for dispensed prescriptions for steroid inhalers which is assumed to indicate diagnosis with asthma or chronic obstructive pulmonary disease (COPD).
Age-based counterfactual	Those age <16 or ≥60 years that had dispensed prescriptions for steroid inhalers which is assumed to indicate diagnosis with asthma or COPD.
Condition-based counterfactual	Those that had dispensed prescriptions for diabetes mellitus treatments which are assumed to indicate diagnosis with said condition.

As only one exemption criteria would have been recorded for each prescription it is not possible to sub-divide the condition-based counterfactual by age. As previously discussed the analysis is forced to examine practice- rather than individual-level data and subsequently the data will be numbers/proportions of relevant prescriptions/admissions for each practice.

Analysis

The studies discussed in the penultimate paragraph of the Background to this document employed a number of statistical methods ranging from the simple univariable analysis to complicated hierarchical multivariable models taking in techniques such as logistic regression (Booth et al., 2012, Kulik et al., 2013, Yin et al., 2008, Zhang et al., 2008) and Cox proportional hazard models (Booth et al., 2012, Kulik et al., 2013). The data to be collected, linked and analysed in the current

project is outlined in Appendix 3, the intention being to produce a panel dataset. For each practice there will be annually 'fixed' socio-demographic data from the General Practice dataset, and monthly (January 2000-December 2013) admissions and prescriptions data. Analysis will be undertaken in Stata (StataCorp, 2009) and will take the format of time series and mediation analysis.

The primary health outcome will be hospital admissions for the respective conditions. The PIS extract dataset will provide outcome data on the potential mediators of any change in the primary outcome. Without individual data and the ability to measure adherence, a number of prescription related metrics will be evaluated as outcomes and mediators. The following metrics will be examined for each of the medication groups (inhaled steroids and diabetes treatments);

- Number of dispensed prescriptions,
- Quantity dispensed,
- Cost of the items dispensed,
- Number of Defined Daily Doses (DDDs) dispensed.

Including the admissions, but excluding costs all the outcomes are count data and consequently will be analysed as such (Poisson regression Generalised Estimating Equations (GEE) or Mixed Models). Cost will be analysed as a continuous outcome. Initially some of the outcomes may be turned into ratios (e.g. DDDs per prescription or prescriptions per general practitioner) in order to smooth the data and account for differences in practice size, to identify the most appropriate set of outcomes. Auto-regression, integration (differencing) and moving average (ARIMA) adjustments will also be explored, to smooth the data and in particular account for the seasonality of dispensing.

Figure 2 – Mediation model for analysis

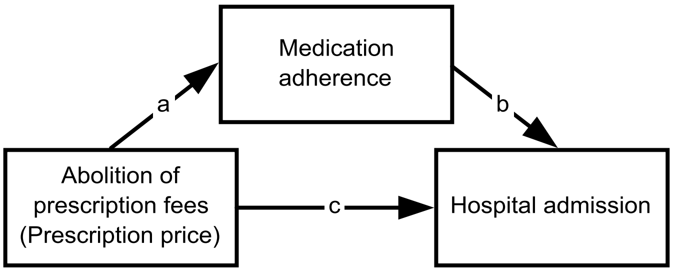

Taking the study aims and the logic model (Figure 1) Figure 2 illustrates the mediation model to be examined through the analysis. The studies discussed in the background provide evidence of relationships **a** and **b** in Figure 2. The preliminary aims of this study (study aims a and b) will be addressed by constructing separate time series of prescriptions and admissions in order to test relationships **a** and **c**. As a practice-level study it will not be possible to directly test relationship **b**. As with previous studies (e.g. Yin et al., 2008, Booth et al., 2012) splines and knots will be employed in order to assess whether changes in admissions and/or prescriptions were contemporary with the reductions and eventual abolition of fees. Furthermore, the use of splines and knots will permit some quantification of any effect of the policy change. The admissions time series will then be adjusted for the prescriptions time series (study aim c), in order to assess the extent to which relationship **c** remains, which will provide information on the nature of relationship **b**. Should relationship **c** remain after adjusting for prescriptions, it would indicate that within the limitations of

this study the policy change had not impacted on health. These analytical steps should strengthen to the study findings.

Given the extent of exemptions criteria (Appendix 2), during the Scottish public consultation it was questioned who would benefit from abolishing the fee (Scottish Executive, 2006, The Scottish Parliament, n.d.). The exemption criteria (Appendix 2) were (and still are in England) considered to be protecting the poorest and sickest who would struggle to pay. It was felt that a reappraisal of the condition-related exemption criteria to consider including conditions like asthma, COPD, dementia might be a sufficient change. As it was, prior to the abolition of prescription fees around 90% of prescriptions were exempt from payment (Personal communication with S. McTaggart, 01/04/2014). In order to examine for the differential impact of the policy across the socioeconomic spectrum (study aim d), the effect of adjusting or stratifying the time series by deprivation and other General Practice characteristics will be explored.

Special cases: As previously discussed those aged 16-18 years will initially be examined separately. Similarly, there is another group of patients who will initially be examined separately. Prior to the abolition of fees, those with chronic conditions were able to purchase discount pre-payment certificates. The price of these certificates changed in line with the reduction in fees. Data published in 2009 demonstrated that there had been a significant increase in the use of pre-payment certificates following the initial reduction in prescription fees (The Scottish Government, 2009). That finding and the different financial decisions related to pre-payment certificates have necessitated the initial examination of this group separately. The use of a pre-payment certificate was captured within the exemptions data within the PIS.

Potential impact

From a research perspective this project would add to the literature on the importance of cost as a determinant of non-adherence with prescribed medications. Given the large number of exemptions from prescription fees prior to the policy change (see Rationale) some have questioned whether universal free prescriptions are just benefiting the less deprived. This study should reveal what the implications of universal free prescriptions have been for those of different socioeconomic status. Some have also questioned whether having to pay for a prescription is an incentive to adherence, although there does not appear to be evidence to back this up, this needs to be considered within this project, the results of which might support or refute that claim. Politically, the project will evaluate an important and possibly costly policy and provide data on the ‘effects’ of the policy. In particular, should a time arise that free prescriptions for all cannot be funded by the state; the stratified component of this project might suggest conditions or circumstances for which free prescriptions are cost effective.

References

Booth, G. L., Bishara, P., Lipscombe, L. L., Shah, B. R., Feig, D. S., Bhattacharyya, O. & Bierman, A. S. 2012. Universal drug coverage and socioeconomic disparities in major diabetes outcomes. *Diabetes Care,* 35**,** 2257-2264.

British Broadcasting Corporation. 2011. *Prescription charges abolished in Scotland* [Online]. London: British Broadcasting Corporation. Available: http://www.bbc.co.uk/news/uk-12928485 [Accessed 24 September 2013].

Campbell, J. D., Allen-Ramey, F., Sajjan, S. G., Maiese, E. M. & Sullivan, S. D. 2011. Increasing pharmaceutical copayments: impact on asthma medication utilization and outcomes. *Am J Manag Care,* 17**,** 703-10.

Craig, P., Cooper, C., Gunnell, D., Haw, S., Lawson, K., Macintyre, S., Ogilvie, D., Petticrew, M., Reeves, B., Sutton, M. & Thompson, S. 2011. *Using natural experiments to evaluate population health interventions: guidance for producers and users of evidence,* London, Medical Research Council. Available: http://www.mrc.ac.uk/documents/pdf/natural-experiments-guidance/ [Accessed 26 February 2014]

Department of Health. 2013. *NHS charges from April 2013 announced* [Online]. London: GOV.UK. Available: https://www.gov.uk/government/news/nhs-charges-from-april-2013-announced [Accessed 15 October 2013].

Fairman, K. & Matheral, B. 2000. Evaluating medication adherence: which measure is right for your program? *Journal of Managed Care Pharmacy,* 6**,** 499-506.

Kardas, P., Lewek, P. & Matyjaszczyk, M. 2013. Determinants of patient adherence: a review of systematic reviews. *Frontiers in Pharmacology,* 4(91).

Kulik, A., Desai, N. R., Shrank, W. H., Antman, E. M., Glynn, R. J., Levin, R., Reisman, L., Brennan, T. & Choudhry, N. K. 2013. Full prescription coverage versus usual prescription coverage after coronary artery bypass graft surgery: analysis from the post-myocardial infarction Free Rx Event and Economic Evaluation (FREEE) randomized trial. *Circulation,* 128**,** S219-S225.

Lehmann, A., Aslani, P., Ahmed, R., Celio, J., Gauchet, A., Bedouch, P., Bugnon, O., Allenet, B. & Schneider, M. P. 2013. Assessing medication adherence: options to consider. *Int J Clin Pharm*, 36(1):55-69.

Linnet, K., Halldórsson, M., Thengilsdóttir, G., Einarsson, Ó. B., Jónsson, K. & Almarsdóttir, A. B. 2013. Primary non-adherence to prescribed medication in general practice: lack of influence of moderate increases in patient copayment. *Family Practice,* 30**,** 69-75.

NHS Choices. 2013. *Get help with prescription costs* [Online]. London: GOV.UK. Available: http://www.nhs.uk/NHSEngland/Healthcosts/Pages/Prescriptioncosts.aspx [Accessed 15 October 2013].

NHS Wales 2010. *Helping to improve Wales's health: free prescriptions three years on,* Cardiff, NHS Wales. Available: http://www.wales.nhs.uk/documents/prescriptions-report-three-years.pdf [Accessed 25 September 2013]

Purdy, S., Griffin, T., Salisbury, C. & Sharp, D. 2009. Ambulatory care sensitive conditions: terminology and disease coding need to be more specific to aid policy makers and clinicians. *Public Health,* 123**,** 169-173.

Scottish Executive. 2006. *Review of NHS prescription charges and exemption arrangements in Scotland - consultation* [Online]. Edinburgh: Scottish Executive. Available:

http://www.scotland.gov.uk/Resource/Doc/90909/0021837.pdf [Accessed 19 February 2014].

StataCorp 2009. Stata statistical software: release 11. College Station, TX: StataCorp LP.

The Scottish Government. 2009. *News; prescription charges* [Online]. Edinburgh: The Scottish Government. Available: http://www.scotland.gov.uk/News/Releases/2009/01/28095040 [Accessed 28 January 2014].

The Scottish Parliament. n.d. *Summary of responses and conclusions: abolition of NHS prescription charges (Scotland) consultation* [Online]. Edinburgh: The Scottish Parliament. Available: http://www.scottish.parliament.uk/S2_MembersBills/Final%20proposals/abolition-prescription-charges-response.pdf [Accessed 19 February 2014].

Yin, W., Basu, A., Zhang, J. X., Rabbani, A., Meltzer, D. O. & Alexander, G. C. 2008. The effect of the Medicare Part D prescription benefit on drug utilization and expenditures. *Ann Intern Med,* 148**,** 169-77.

Zhang, J. X., Yin, W., Sun, S. X. & Alexander, G. C. 2008. The impact of the Medicare Part D prescription benefit on generic drug use. *J Gen Intern Med,* 23**,** 1673-8.

Appendix 1: Ambulatory case sensitive conditions Purdy et al. (2009)

Angina	Nutritional deficiency	Hypokalaemia	Stroke
Asthma	Hypertension	Low birth weight	Pyelonephritis
Cellulitis	Failure to thrive	Neuroses	Diabetes complications
Dental complications	Ruptured appendix	Tuberculosis	Gangrene
Constipation	Deliberate self-harm	Senility/dementia	Schizophrenia
Congestive heart failure	Fractured proximal femur	Influenza and pneumonia	Migraine/acute headache
Iron-deficiency anaemia	Alcohol-related disease	Atrial fibrillation/flutter	Convulsions and epilepsy
Dehydration and gastroenteritis	Perforated/bleeding ulcer	Ear, nose and throat infections	Pelvic inflammatory disease
Other vaccine-preventable diseases	Dyspepsia and other stomach function disorders	Chronic obstructive pulmonary disease	Peripheral vascular disease

Appendix 2: Criteria exempting patients from paying prescription fees in England and Scotland prior to April 2011 (NHS Choices, 2013).

- Condition-related
  - Cancer (from 2009)
  - Those on renal dialysis with a permanent fistula
  - Pregnant
  - Contraceptives
  - Sexually transmitted infection
  - Tuberculosis

    - Hypoadrenalism for which specific substitution therapy is essential
    - Diabetes insipidus or other forms of hypopituitarism
    - Diabetes mellitus, except where treatment is by diet alone
    - Hypoparathyroidism
    - Myasthenia gravis
    - Myxoedema
    - Epilepsy requiring continuous anticonvulsive therapy
    - A continuing physical disability which means the person cannot go out without the help of another person.
- Age-related
    - are 60 or over
    - are under 16
    - are 16-18 and in full-time education
- Means tested, and those in receipt of the following benefits ('income-related')
    - Income Support
    - Income-based Jobseeker's Allowance
    - Income-related Employment and Support Allowance
    - Pension Credit Guarantee Credit
    - Universal Credit
    - Those in receipt of a war pension

Appendix 3: Data
The final study dataset will be compiled from three separate datasets. Two of these datasets will be extracts from the Scottish Morbidity Record (SMR01) and Prescriptions Information System (PIS). The third dataset will be the General Practice data compiled from data published by ISD Scotland (http://www.isdscotland.org/Health-Topics/General-Practice/) and more historical data provided through eDRIS. The General Practice dataset will be linked by the researcher using the practice code. The SMR01 and PIS data extracts will be linked by the researcher using the practice code, year and month. The General Practice dataset will then be linked onto the linked SMR01 and PIS data, and the data rearranged in order that each month of SMR01 and PIS data will only have the relevant years general practice descriptive variables. The final dataset should be in the form of panel data for the time series analysis. The variables in each of the three datasets are listed below.

N.B. Gender and ethnicity data are not available in the practice-level PIS and have subsequently not been sought in the other data.

Farr Institute @ Scotland – NEA group / SCPHRP
Andrew James Williams

General Practice dataset – by practice and year

**Variable**	**Details**
NHS board (code)	Unique single alphabetical character for each health board (publically available)*
NHS board (name)	Name of each health board (publically available)*
Practice Code	The unique NHS Scotland code for the GP Practice (publically available)
Practice name	Name of each General Practice (publically available)*
Number of General Practitioners	(requested for each year through eDRIS)
Dispensing practice	Identifier of practices where medicines are dispensed as well as prescribed (requested for each year through eDRIS)
Contract type	Indicator of which type of contract the practice has with the NHS (requested for each year through eDRIS)
Practice List Size	Total number of registered patients each year (publically available for the years 1999-2013)
Age profile of practice list	Number of patients in each of the following age categories; <16, 16-18, 18-60, ≥60 years (requested for each year through eDRIS)
pc_most_dep15	Percentage of practice patients living in datazones defined as the 15% most deprived (population weighted) (publically available from 2005-2013, earlier years requested through eDRIS)
quint1 – quint5	Number of practice patients matched to the relevant first through fifth quintile of deprivation (publically available from 2005-2013, earlier years requested through eDRIS)
prac_ur8	Urban/rural classification of the postcode of the General Practice (publically available from 2005-2013, earlier years requested through eDRIS)
urban1 – urban8	Number of practice patients in each of the eight urban/rural classifications (publically available from 2005-2013, earlier years requested through eDRIS)
unmatch	Number of practice patients who could not be matched to an urban/rural classification (publically available from 2005-2013, earlier years requested through eDRIS)
mode_ur8	Modal urban/rural classification of the practice patients (publically available from 2005-2013, earlier years requested through eDRIS)

*These data are for linking and/or clustering purposes and would be anonymised in any publications.

Farr Institute @ Scotland – NEA group / SCPHRP
Andrew James Williams

Scottish Morbidity Record (SMR01) extract dataset – by practice, year and month

**Variable**	**Details**
Practice Code	The unique NHS Scotland code for the GP Practice
Year	The year to which the data relates (2000-13)
Month	The month to which the data relates
Diagnosis	The diagnosis (based on DIAGNOSIS 1) to which the data relates (ALL admissions, Asthma/COPD† or Diabetes‡
Age group	The age banding (0-15, 16-18, 19-59, 60+ years) to which the data relates
Admissions	The total number of admissions for practice patients

†International Classification of Disease 10 codes J20, J40X, J41, J42, J43, J44, J45, J46 or J47
‡International Classification of Disease 10 codes E10, E11, E12, E13 or E14

Prescriptions Information System (PIS) extract dataset – by practice, year and month

**Variable**	**Details**
Practice Code	The unique NHS Scotland code for the GP Practice
Year	The year to which the data relates (2000-13)
Month	The month to which the data relates
Category	The medication class to which the data relates (ALL prescriptions, Inhaled Corticosteroids or Diabetes)
Paid/Exempt	A flag to indicate whether the data relates to Paid or Exempt prescriptions
Exemption Category*	The prescription exemption category to which the data relates
Number of Items Paid	The number of prescription items paid
Quantity Paid	The total quantity paid
Gross Ingredient Cost Paid	The total cost of the medicines supplied before discount
DDDs Paid	The total number of defined daily doses paid

*For data from April 2011, exemption category will not be available. This should be replaced by the following age-bandings: 0-15, 16-18, 19-59; 60+ years. Ages should be based on the date of prescribing.
