# Supplementary material for: Impact of abolishing prescription fees in Scotland on hospital admissions and prescribed medicines: an interrupted time series evaluation
Source: BMJ Open. 2018 Dec 18;8(12):e021318. doi: 10.1136/bmjopen-2017-021318 (PMC6303621; doi:10.1136/bmjopen-2017-021318)

Supplementary file 3 Generalized Additive Mixed Models sensitivity analysis

Admissions

Intervention

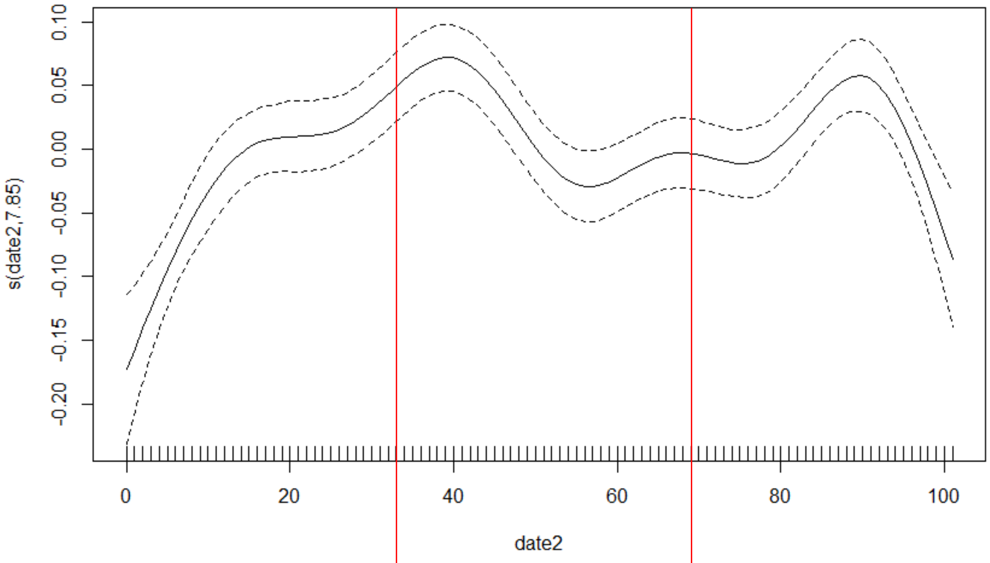

Age counterfactual

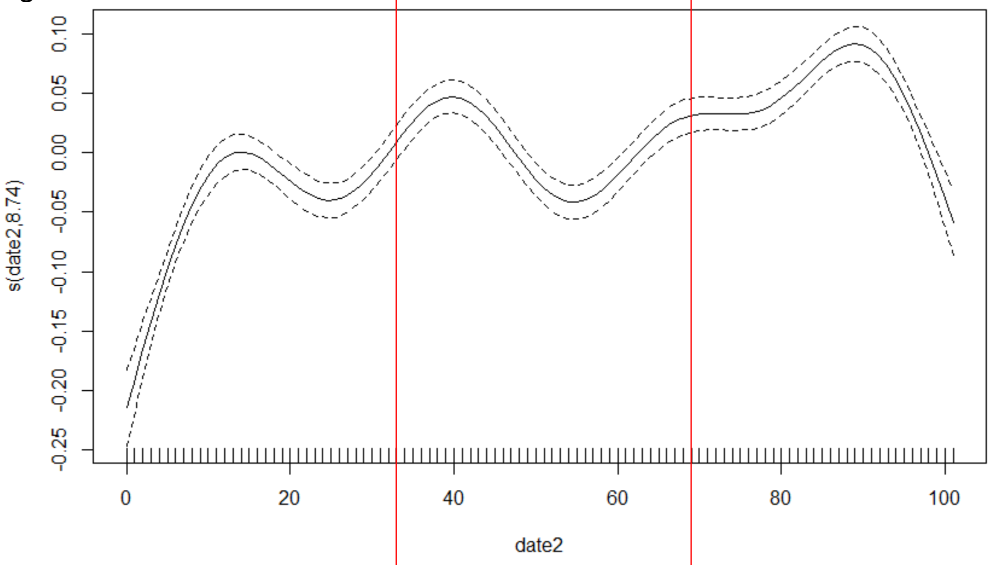

Condition counterfactual

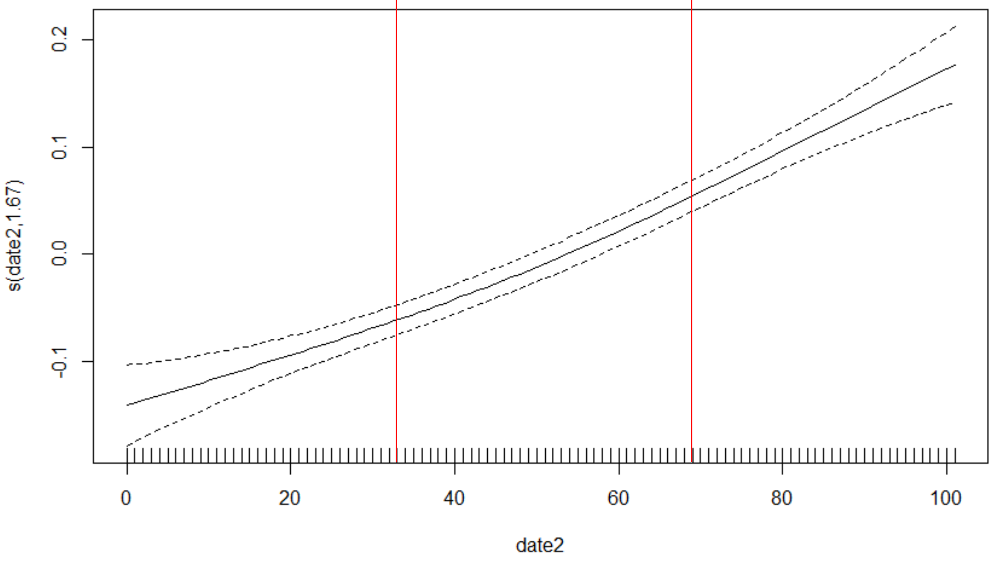

Supplementary file 3 Generalized Additive Mixed Models sensitivity analysis

Defined Daily Doses (DDDs)

Intervention

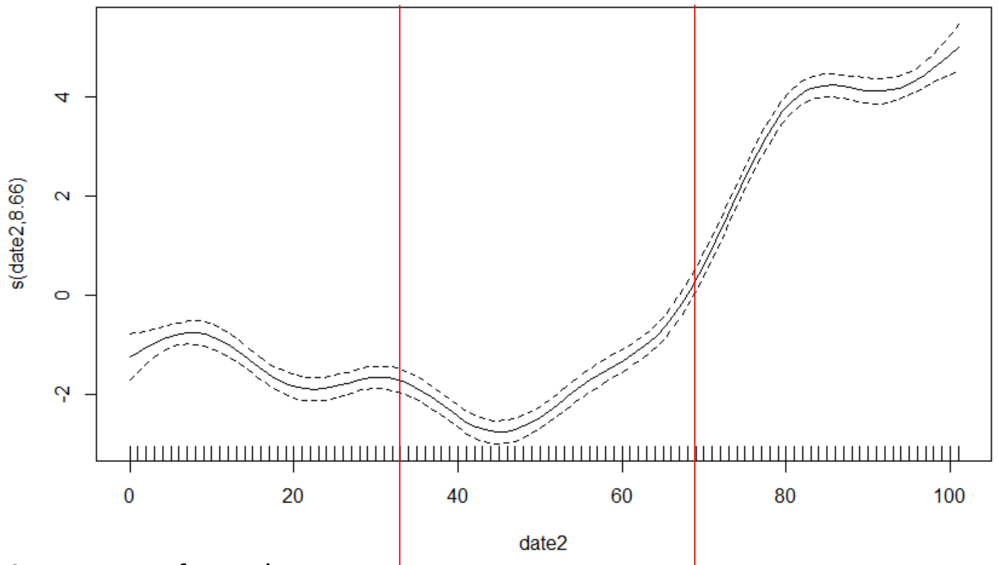

Age counterfactual

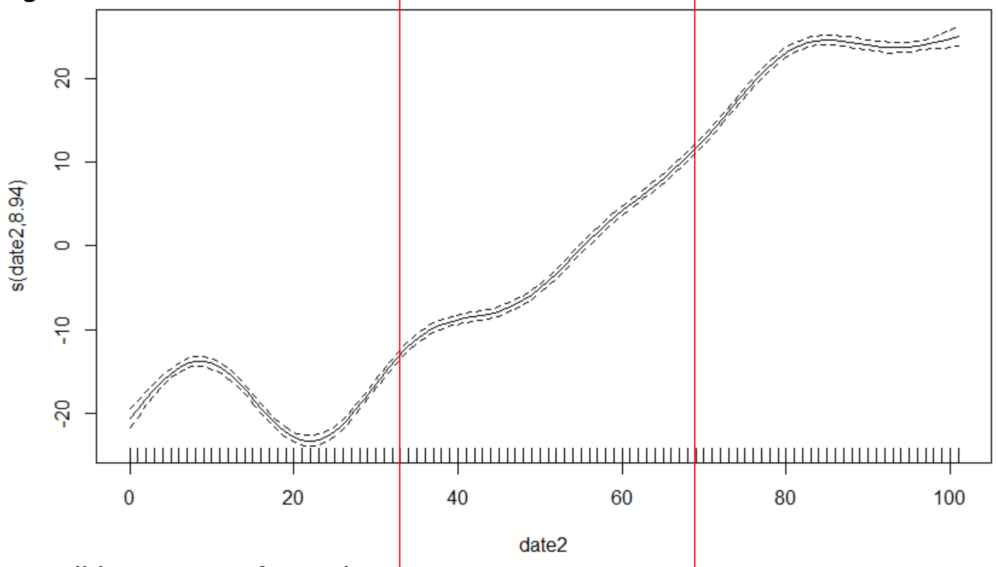

Condition counterfactual

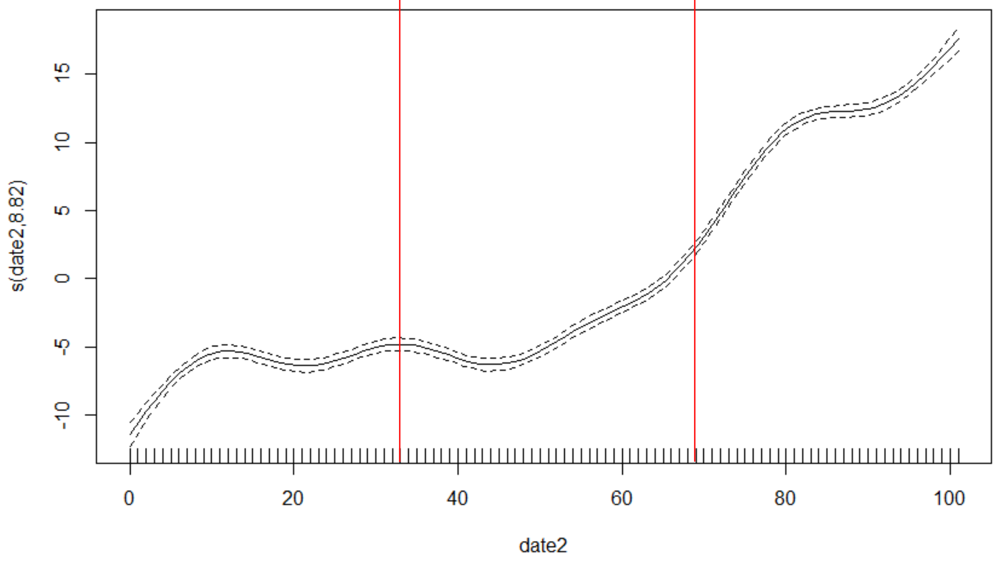

Supplementary file 3 Generalized Additive Mixed Models sensitivity analysis

Cost

Intervention

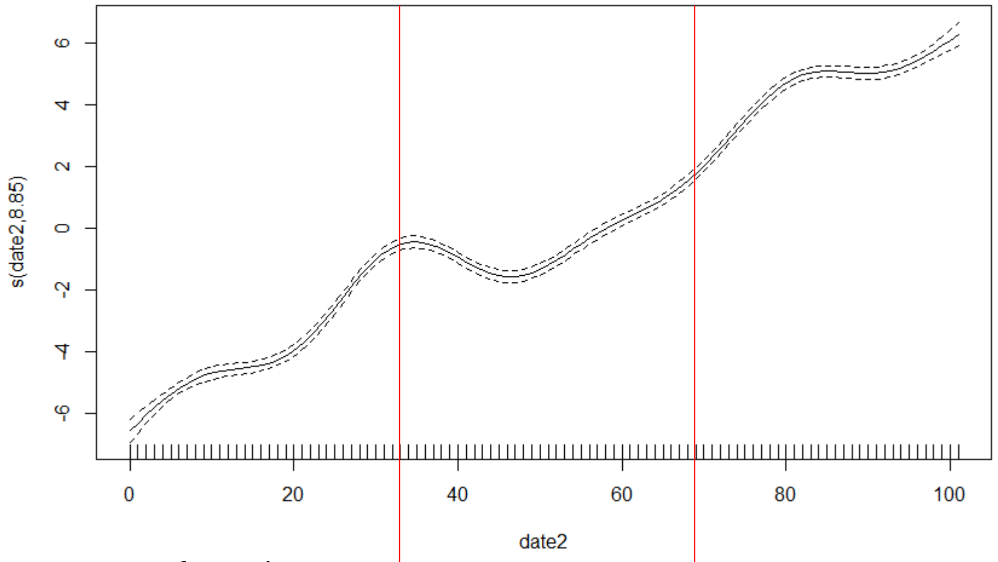

Age counterfactual

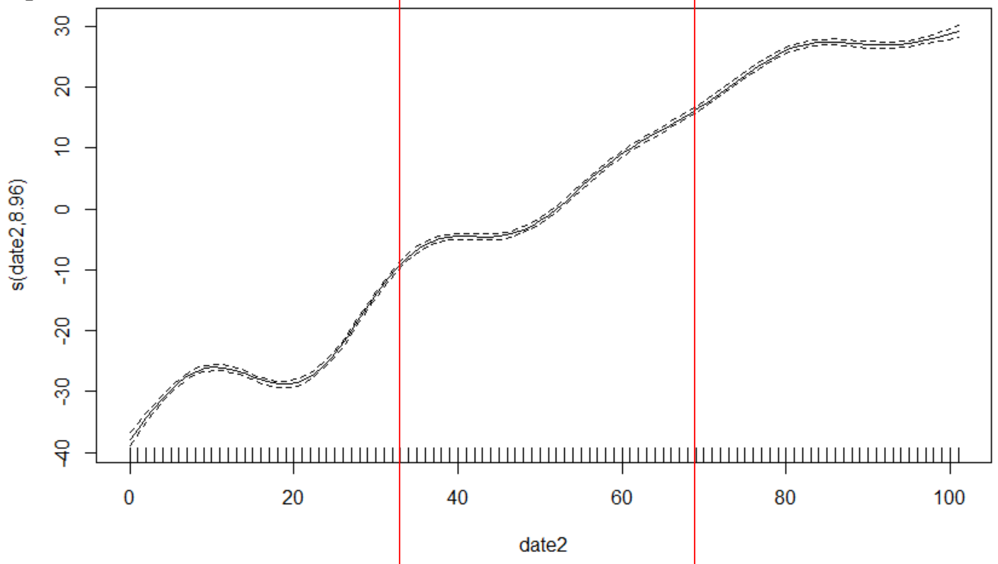

Condition counterfactual

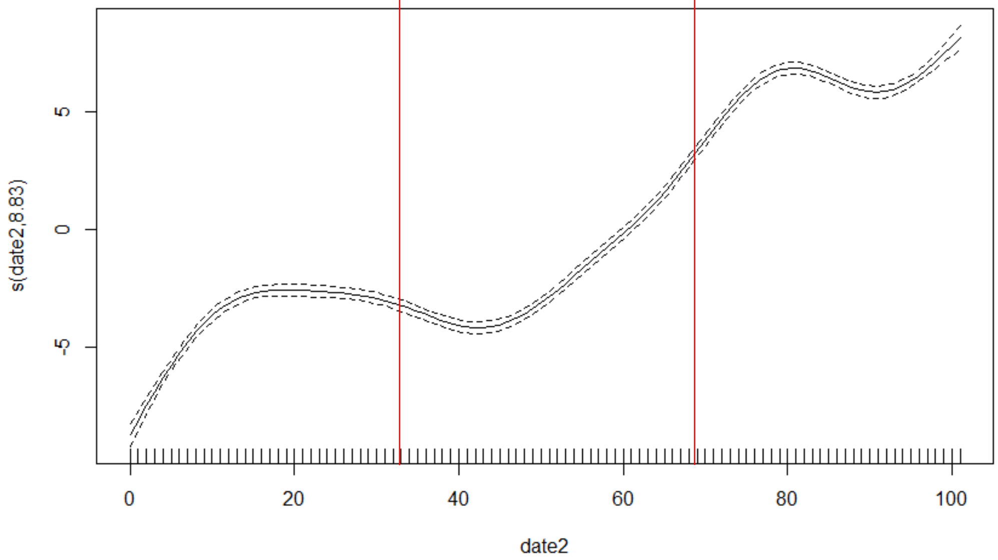

Supplement: Supplementary file 3 [file bmjopen-2017-021318supp003.pdf]
