# Supplementary material for: Impact of abolishing prescription fees in Scotland on hospital admissions and prescribed medicines: an interrupted time series evaluation
Source: BMJ Open. 2018 Dec 18;8(12):e021318. doi: 10.1136/bmjopen-2017-021318 (PMC6303621; doi:10.1136/bmjopen-2017-021318)
Supplement: Supplementary file 4 [file bmjopen-2017-021318supp004.pdf]

Supplementary file 4 Health board clustering sensitivity analysis

Table S4.1 – Results of interrupted time series analysis of the impact of reducing and abolishing prescription fees in Scotland on hospital admissions (incidence rate ratios) and prescribed medicines (defined daily doses and cost) – adjusting for clustering with General Practice and Health Board

	**Intercept**		**Monthly change**		**Reduction step change**		**Reduction change in slope**		**Abolition step change**		**Abolition change in slope**	
**Admissions**	IR	95% CI	IRR	95% CI	IRR	95% CI	IRR	95% CI	IRR	95% CI	IRR	95% CI
Intervention	$9.04e^{-3}$	$7.75e^{-3}$ to $1.06e^{-2}$	1.00	1.00 to 1.01	1.03	0.98 to 1.08	0.99	0.99 to 0.99	0.81	0.72 to 0.90	1.00	0.99 to 1.00
Age-c'fact	$5.70e^{-2}$	$4.95e^{-2}$ to $6.56e^{-2}$	1.00	1.00 to 1.00	1.03	1.01 to 1.06	1.00	0.99 to 1.00	0.95	0.90 to 1.00	1.00	0.99 to 1.00
Condition-c'fact	$4.72e^{-3}$	$4.14e^{-3}$ to $5.38e^{-3}$	1.00	1.00 to 1.01	0.95	0.88 to 1.03	1.00	1.00 to 1.01	1.01	0.86 to 1.20	1.00	1.00 to 1.01
**Admissions 1 month lag**	IR	95% CI	IRR	95% CI	IRR	95% CI	IRR	95% CI	IRR	95% CI	IRR	95% CI
Intervention	$9.07e^{-3}$	$7.77e^{-3}$ to $1.06e^{-2}$	1.01	1.00 to 1.01	1.01	0.96 to 1.06	0.99	0.99 to 0.99	0.78	0.71 to 0.87	1.00	0.99 to 1.00
Age-c'fact	$5.78e^{-2}$	$5.02e^{-2}$ to $6.65e^{-2}$	1.00	1.00 to 1.00	1.03	1.01 to 1.06	1.00	0.99 to 1.00	0.95	0.90 to 1.00	1.00	0.99 to 1.00
Condition-c'fact	$4.74e^{-3}$	$4.16e^{-3}$ to $5.42e^{-3}$	1.00	1.00 to 1.00	0.98	0.91 to 1.06	1.00	1.00 to 1.00	1.07	0.91 to 1.26	1.00	1.00 to 1.01
**Admissions 2 month lag**	IR	95% CI	IRR	95% CI	IRR	95% CI	IRR	95% CI	IRR	95% CI	IRR	95% CI
Intervention	$9.09e^{-3}$	$7.79e^{-3}$ to $1.06e^{-2}$	1.00	1.00 to 1.01	1.01	0.96 to 1.06	0.99	0.99 to 0.99	0.79	0.71 to 0.87	1.00	0.99 to 1.00
Age-c'fact	$5.80e^{-2}$	$5.04e^{-2}$ to $6.68e^{-2}$	1.00	1.00 to 1.00	1.04	1.02 to 1.07	1.00	0.99 to 1.00	0.97	0.92 to 1.02	1.00	1.00 to 1.00
Condition-c'fact	$4.77e^{-3}$	$4.18e^{-3}$ to $5.44e^{-3}$	1.00	1.00 to 1.00	0.99	0.92 to 1.07	1.00	1.00 to 1.01	1.10	0.93 to 1.29	1.00	1.00 to 1.01
**Admissions 3 month lag**	IR	95% CI	IRR	95% CI	IRR	95% CI	IRR	95% CI	IRR	95% CI	IRR	95% CI
Intervention	$9.07e^{-3}$	$7.77e^{-3}$ to $1.06e^{-2}$	1.01	1.00 to 1.01	0.99	0.94 to 1.04	0.99	0.99 to 0.99	0.78	0.70 to 0.86	1.00	0.99 to 1.00
Age-c'fact	$5.77e^{-2}$	$5.01e^{-2}$ to $6.64e^{-2}$	1.00	1.00 to 1.00	1.02	1.00 to 1.05	1.00	0.99 to 1.00	0.94	0.90 to 0.99	1.00	1.00 to 1.00
Condition-c'fact	$4.74e^{-3}$	$4.15e^{-3}$ to $5.40e^{-3}$	1.00	1.00 to 1.00	0.97	0.90 to 1.05	1.00	1.00 to 1.01	1.08	0.92 to 1.26	1.00	1.00 to 1.00
**Defined Daily Doses (DDDs)**	DDDs	95% CI	ΔDDDs	95% CI	ΔDDDs	95% CI	ΔDDDs	95% CI	ΔDDDs	95% CI	ΔDDDs	95% CI
Intervention	33.21	30.30 to 36.12	-0.04	-0.06 to -0.02	-0.11	-0.61 to 0.38	0.04	0.02 to 0.07	6.03	4.93 to 7.13	0.10	0.08 to 0.13
Age-c'fact	-	-	-	-	-	-	-	-	-	-	-	-
Condition-c'fact	59.82	54.81 to 64.82	0.14	0.10 to 0.18	-1.88	-2.90 to -0.86	-0.05	-0.11 to <0.01	6.02	3.72 to 8.31	0.15	0.09 to 0.20
**Cost**	£	95% CI	Δ£	95% CI	Δ£	95% CI	Δ£	95% CI	Δ£	95% CI	Δ£	95% CI
Intervention	22.44	20.11 to 24.77	0.17	0.15 to 0.18	0.23	-0.20 to 0.65	-0.15	-0.17 to -0.13	-0.97	-1.92 to -0.02	-0.10	-0.12 to -0.08
Age-c'fact	67.90	62.04 to 73.76	0.62	0.56 to 0.68	4.74	3.43 to 6.06	0.03	-0.05 to 0.12	14.26	10.87 to 17.65	-0.36	-0.44 to -0.27
Condition-c'fact	29.61	26.75 to 32.47	0.15	0.13 to 0.17	-3.16	-3.70 to -2.62	-0.01	-0.04 to 0.02	1.99	0.78 to 3.20	-0.11	-0.14 to -0.09

95% CI; 95% confidence interval, c'fact; counterfactual, DDDs; Defined daily doses per 100 patients per practice per month, ΔDDDs; change in DDDs, IR; Incidence rate per 100 patients per practice per month, IRR; incidence rate ratio, £s; gross ingredient cost of medicines before any discount per 100 patients per practice per month, Δ£s; change in £s

Supplemental file 4 Health board clustering sensitivity analysis

Table S4.2 - Results of interrupted time series analysis of the impact of reducing and abolishing prescription fees in Scotland on hospital admissions (incidence rate ratios) and prescribed medicines (defined daily doses and cost) stratified by quintile of the Scottish Index of Multiple Deprivation [19] – adjusting for clustering with General Practice and Health Board

	**Intercept**		**Monthly change**		**Reduction step change**		**Reduction change in slope**		**Abolition step change**		**Abolition change in slope**	
**Admissions**	IR	95% CI	IRR	95% CI	IRR	95% CI	IRR	95% CI	IRR	95% CI	IRR	95% CI
Q1 - most deprived	$9.08e^{-2}$	$7.67e^{-2}$ to $1.08e^{-1}$	1.01	1.00 to 1.01	1.02	0.97 to 1.06	0.99	0.99 to 1.00	0.87	0.79 to 0.97	0.99	0.99 to 1.00
Q2	$1.17e^{-2}$	$9.36e^{-3}$ to $1.47e^{-2}$	1.00	1.00 to 1.01	1.10	0.92 to 1.32	0.99	0.98 to 1.00	0.75	0.50 to 1.12	1.00	0.99 to 1.01
Q3	$9.80e^{-3}$	$7.03e^{-3}$ to $1.37e^{-2}$	1.00	0.99 to 1.01	1.10	0.86 to 1.41	1.00	0.98 to 1.01	1.15	0.68 to 1.94	1.00	0.99 to 1.02
Q4	$6.51e^{-3}$	$4.62e^{-3}$ to $9.18e^{-3}$	0.99	0.98 to 1.00	1.50	1.13 to 1.99	1.00	0.99 to 1.02	1.63	0.89 to 2.99	1.02	1.01 to 1.04
Q5 - least deprived	$6.06e^{-3}$	$4.86e^{-3}$ to $7.54e^{-3}$	1.00	0.99 to 1.00	1.00	0.84 to 1.21	1.00	1.00 to 1.01	1.24	0.85 to 1.82	1.00	0.99 to 1.01
**Admissions 1 month lag**	IR	95% CI	IRR	95% CI	IRR	95% CI	IRR	95% CI	IRR	95% CI	IRR	95% CI
Q1 - most deprived	$1.45e^{-2}$	$1.23e^{-2}$ to $1.72e^{-2}$	1.01	1.01 to 1.02	0.94	0.86 to 1.03	0.99	0.98 to 0.99	0.60	0.49 to 0.73	0.99	0.98 to 0.99
Q2	$1.20e^{-2}$	$9.58e^{-3}$ to $1.51e^{-2}$	1.00	1.00 to 1.01	1.12	0.93 to 1.34	0.99	0.98 to 1.00	0.79	0.53 to 1.16	1.00	0.99 to 1.01
Q3	$9.77e^{-3}$	$6.99e^{-3}$ to $1.37e^{-2}$	1.00	0.99 to 1.01	1.00	0.78 to 1.27	1.00	0.99 to 1.01	0.99	0.60 to 1.65	1.00	0.99 to 1.02
Q4	$6.84e^{-3}$	$4.83e^{-3}$ to $9.67e^{-3}$	0.99	0.98 to 1.00	1.53	1.16 to 2.02	1.00	0.99 to 1.01	1.66	0.91 to 3.00	1.02	1.01 to 1.04
Q5 - least deprived	$6.12e^{-3}$	$4.90e^{-3}$ to $7.64e^{-3}$	1.00	0.99 to 1.00	1.03	0.86 to 1.23	1.01	1.00 to 1.01	1.27	0.87 to 1.85	1.00	0.99 to 1.01
**Admissions 2 month lag**	IR	95% CI	IRR	95% CI	IRR	95% CI	IRR	95% CI	IRR	95% CI	IRR	95% CI
Q1 - most deprived	$1.46e^{-2}$	$1.24e^{-2}$ to $1.73e^{-2}$	1.01	1.01 to 1.01	0.94	0.86 to 1.03	0.99	0.98 to 0.99	0.60	0.50 to 0.73	0.99	0.98 to 0.99
Q2	$1.20e^{-2}$	$9.58e^{-3}$ to $1.51e^{-2}$	1.00	1.00 to 1.01	1.11	0.92 to 1.33	0.99	0.98 to 1.00	0.78	0.54 to 1.15	1.00	0.99 to 1.01
Q3	$1.00e^{-2}$	$1.00e^{-2}$ to $1.40e^{-2}$	1.00	0.99 to 1.01	1.07	0.84 to 1.37	1.00	0.99 to 1.01	1.12	0.68 to 1.84	1.00	0.99 to 1.02
Q4	$6.80e^{-3}$	$4.81e^{-3}$ to $9.60e^{-3}$	0.99	0.98 to 1.00	1.57	1.19 to 2.07	1.00	0.98 to 1.01	1.70	0.95 to 3.05	1.02	1.00 to 1.03
Q5 - least deprived	$6.21e^{-3}$	$4.98e^{-3}$ to $7.75e^{-3}$	0.99	0.99 to 1.00	1.09	0.91 to 1.31	1.00	1.00 to 1.01	1.38	0.95 to 1.99	1.00	0.99 to 1.01
**Admissions 3 month lag**	IR	95% CI	IRR	95% CI	IRR	95% CI	IRR	95% CI	IRR	95% CI	IRR	95% CI
Q1 - most deprived	$1.47e^{-2}$	$1.24e^{-2}$ to $1.74e^{-2}$	1.01	1.01 to 1.01	0.94	0.86 to 1.02	0.99	0.98 to 0.99	0.60	0.50 to 0.73	0.99	0.98 to 0.99
Q2	$1.18e^{-2}$	$9.40e^{-3}$ to $1.48e^{-2}$	1.01	1.00 to 1.01	1.03	0.86 to 1.24	0.99	0.98 to 1.00	0.72	0.50 to 1.05	1.00	0.99 to 1.01
Q3	$1.03e^{-2}$	$7.38e^{-3}$ to $1.43e^{-2}$	1.00	0.99 to 1.00	1.18	0.92 to 1.51	1.00	0.99 to 1.01	1.25	0.76 to 2.05	1.01	0.99 to 1.02
Q4	$6.43e^{-3}$	$4.55e^{-3}$ to $9.07e^{-3}$	0.99	0.98 to 1.00	1.34	1.02 to 1.77	1.00	0.98 to 1.01	1.36	0.77 to 2.39	1.01	1.00 to 1.03
Q5 - least deprived	$6.15e^{-3}$	$4.93e^{-3}$ to $7.67e^{-3}$	1.00	0.99 to 1.00	1.07	0.89 to 1.28	1.00	0.99 to 1.01	1.35	0.94 to 1.94	1.00	0.99 to 1.01
**Defined Daily Doses (DDDs)**	DDDs	95% CI	ΔDDDs	95% CI	ΔDDDs	95% CI	ΔDDDs	95% CI	ΔDDDs	95% CI	ΔDDDs	95% CI
Q1 - most deprived	24.46	21.29 to 27.62	-0.07	-0.10 to -0.04	0.25	-0.50 to 1.01	0.08	0.04 to 0.12	6.23	4.59 to 7.86	0.14	0.10 to 0.18
Q2	29.64	25.30 to 33.99	0.05	-0.03 to 0.12	-1.83	-3.80 to 0.15	-0.05	-0.15 to 0.05	2.09	-2.19 to 6.37	0.03	-0.07 to 0.14
Q3	35.64	31.29 to 39.98	-0.06	-0.18 to 0.06	1.11	-2.07 to 4.28	-0.03	-0.20 to 0.13	7.32	0.27 to 14.37	0.15	-0.02 to 0.32
Q4	37.99	34.12 to 41.87	-0.05	-0.14 to 0.03	0.12	-2.06 to 2.31	0.06	-0.05 to 0.17	7.62	2.87 to 12.38	0.09	-0.03 to 0.21
Q5 - least deprived	30.20	28.03 to 32.37	0.02	-0.02 to 0.06	-1.15	-2.24 to -0.05	$9.05e^{-4}$	-0.06 to 0.06	4.21	1.83 to 6.60	0.01	-0.05 to 0.06

Supplemental file 4 Health board clustering sensitivity analysis

	Intercept		Monthly change		Reduction step change		Reduction change in slope		Abolition step change		Abolition change in slope	
**Cost**	£s	95% CI	Δ£s	95% CI	Δ£s	95% CI	Δ£s	95% CI	Δ£s	95% CI	Δ£s	95% CI
Q1 - most deprived	17.49	14.36 to 20.62	0.07	0.05 to 0.09	0.37	-0.24 to 0.98	-0.06	-0.09 to -0.03	1.02	-0.32 to 2.36	-0.02	-0.05 to 0.02
Q2	22.94	17.26 to 17.26	0.21	0.14 to 0.28	-0.88	-2.67 to 0.90	-0.21	-0.30 to -0.12	-2.63	-6.59 to 1.32	-0.18	-0.28 to -0.09
Q3	22.97	19.33 to 26.60	0.20	0.10 to 0.29	0.83	-1.55 to 3.20	-0.25	-0.38 to -0.13	-1.67	-6.95 to 3.62	-0.08	-0.21 to 0.04
Q4	24.86	21.27 to 28.46	0.22	0.14 to 0.30	0.14	-1.92 to 2.21	-0.24	-0.35 to -0.13	-2.63	-7.26 to 2.01	-0.19	-0.30 to -0.08
Q5 - least deprived	20.76	17.57 to 23.94	0.20	0.17 to 0.24	-0.78	-1.72 to 0.16	-0.18	-0.23 to -0.14	-2.67	-4.76 to -0.58	-0.18	-0.23 to -0.13

95% CI; 95% confidence interval; DDDs; Defined daily doses per 100 patients per practice per month, ΔDDDs; change in DDDs, IR; Incidence rate per 100 patients per practice per month, IRR; incidence rate ratio, £s; gross ingredient cost of medicines before any discount per 100 patients per practice per month, Δ£s; change in £s, Q1-5; quintiles of the Scottish Index of Multiple Deprivation from most to least deprived
