# Supplementary material for: Impact of abolishing prescription fees in Scotland on hospital admissions and prescribed medicines: an interrupted time series evaluation
Source: BMJ Open. 2018 Dec 18;8(12):e021318. doi: 10.1136/bmjopen-2017-021318 (PMC6303621; doi:10.1136/bmjopen-2017-021318)

Supplementary file 5 Health board clustering sensitivity analysis

Table S5 - Quadratic time effects

	**Intercept**		**Monthly change**		**Quadratic monthly change**	
**Admissions**	IRR	95% CI	ΔIRR	95% CI	ΔIRR	95% CI
Intervention						
Linear secular and intervention time effects	0.010	0.009 to 0.011	1.005	1.003 to 1.007	-	-
plus quadratic time effect	0.010	0.009 to 0.011	1.008	1.004 to 1.012	1.000	1.000 to 1.000
plus quadratic intervention effects	0.010	0.009 to 0.011	1.005	1.003 to 1.007	-	-
Age-based counterfactual						
Linear secular and intervention time effects	0.066	0.064 to 0.069	1.003	1.002 to 1.004	-	-
plus quadratic time effect	0.065	0.062 to 0.068	1.008	1.006 to 1.010	1.000	1.000 to 1.000
plus quadratic intervention effects	0.067	0.064 to 0.070	1.003	1.002 to 1.004	-	-
Condition-based counterfactual						
Linear secular and intervention time effects	0.005	0.004 to 0.005	1.003	1.000 to 1.006	-	-
plus quadratic time effect	0.005	0.004 to 0.005	1.004	0.998 to 1.011	1.000	1.000 to 1.000
plus quadratic intervention effects	0.005	0.004 to 0.005	1.003	1.000 to 1.006	-	-
**Defined Daily Doses (DDDs)**	DDDs	95% CI	ΔDDDs	95% CI	ΔDDDs	95% CI
Intervention						
Linear secular and intervention time effects	29.648	28.711 to 30.585	-0.036	-0.055 to -0.017	-	-
plus quadratic time effect	29.504	28.549 to 30.459	-0.007	-0.049 to 0.036	-0.001	-0.002 to <0.001
plus quadratic intervention effects	29.662	28.725 to 30.600	-0.037	-0.056 to -0.017	-	-
Age-based counterfactual						
Linear secular and intervention time effects	100.885	98.067 to 103.703	-0.052	-0.113 to 0.009	-	-
plus quadratic time effect	100.370	97.508 to 103.232	0.068	-0.064 to 0.200	-0.004	-0.008 to <0.001
plus quadratic intervention effects	101.024	98.203 to 103.845	-0.053	-0.115 to 0.009	-	-
Condition-based counterfactual						
Linear secular and intervention time effects	55.612	53.698 to 57.527	0.143	0.104 to 0.183	-	-
plus quadratic time effect	55.119	53.169 to 57.069	0.248	0.160 to 0.336	-0.003	-0.006 to -0.001
plus quadratic intervention effects	55.645	53.729 to 57.561	0.141	0.101 to 0.181	-	-
**Cost**	£	95% CI	Δ£	95% CI	Δ£	95% CI
Intervention						
Linear secular and intervention time effects	19.364	18.535 to 20.194	0.165	0.148 to 0.182	-	-
plus quadratic time effect	19.558	18.714 to 20.402	0.124	0.088 to 0.161	0.001	<0.001 to 0.002
plus quadratic intervention effects	19.350	18.520 to 20.180	0.165	0.149 to 0.182	-	-
Age-based counterfactual						
Linear secular and intervention time effects	69.920	67.349 to 72.492	0.621	0.563 to 0.679	-	-
plus quadratic time effect	70.090	67.480 to 72.700	0.579	0.455 to 0.704	0.001	-0.002 to 0.005
plus quadratic intervention effects	69.998	67.422 to 72.574	0.621	0.561 to 0.680	-	-
Condition-based counterfactual						
Linear secular and intervention time effects	26.345	25.349 to 27.341	0.149	0.128 to 0.170	-	-
plus quadratic time effect	25.960	24.945 to 26.976	0.229	0.182 to 0.275	-0.003	-0.004 to -0.001
plus quadratic intervention effects	26.291	26.291 to 27.287	0.150	0.129 to 0.171	-	-

95% CI; 95% confidence interval, DDDs; Defined daily doses per 100 patients per practice per month, ΔDDDs; change in DDDs, IR; Incidence rate per 100 patients per practice per month, IRR; incidence rate ratio, £s; gross ingredient cost of medicines before any discount per 100 patients per practice per month, Δ£s; change in £s

Supplementary file 5 Health board clustering sensitivity analysis

Table S5 (continued) - Quadratic time effects

	Reduction step change		Reduction change in slope		Reduction change in slope (quadratic)	
**Admissions**	ΔIRR	95% CI	ΔIRR	95% CI	ΔIRR	95% CI
Intervention						
Linear secular and intervention time effects	1.032	0.982 to 1.084	0.992	0.989 to 0.994	-	-
plus quadratic time effect	1.030	0.980 to 1.083	0.999	0.990 to 1.007	-	-
plus quadratic intervention effects	1.082	1.018 to 1.150	0.983	0.977 to 0.990	1.000	1.000 to 1.000
Age-based counterfactual						
Linear secular and intervention time effects	1.033	1.007 to 1.059	0.995	0.994 to 0.997	-	-
plus quadratic time effect	1.030	1.004 to 1.057	1.005	1.001 to 1.009	-	-
plus quadratic intervention effects	1.069	1.037 to 1.102	0.989	0.986 to 0.992	1.000	1.000 to 1.000
Condition-based counterfactual						
Linear secular and intervention time effects	0.952	0.880 to 1.029	1.002	0.998 to 1.005	-	-
plus quadratic time effect	0.951	0.879 to 1.028	1.005	0.993 to 1.018	-	-
plus quadratic intervention effects	0.947	0.860 to 1.043	1.002	0.992 to 1.012	1.000	1.000 to 1.000
**Defined Daily Doses (DDDs)**	ΔDDDs	95% CI	ΔDDDs	95% CI	ΔDDDs	95% CI
Intervention						
Linear secular and intervention time effects	-0.113	-0.612 to 0.386	0.042	0.016 to 0.068	-	-
plus quadratic time effect	-0.107	-0.606 to 0.393	0.107	0.020 to 0.194	-	-
plus quadratic intervention effects	-0.230	-0.815 to 0.355	0.065	-0.002 to 0.132	-0.001	-0.002 to 0.001
Age-based counterfactual						
Linear secular and intervention time effects	5.065	3.614 to 6.515	0.681	0.594 to 0.768	-	-
plus quadratic time effect	5.144	3.692 to 6.597	0.957	0.674 to 1.240	-	-
plus quadratic intervention effects	4.749	3.158 to 6.340	0.759	0.548 to 0.970	-0.003	-0.008 to 0.002
Condition-based counterfactual						
Linear secular and intervention time effects	-1.876	-2.897 to -0.855	-0.054	-0.108 to 0.001	-	-
plus quadratic time effect	-1.839	-2.860 to -0.817	0.179	-0.003 to 0.362	-	-
plus quadratic intervention effects	-2.226	-3.401 to -1.052	0.025	-0.114 to 0.164	-0.002	-0.006 to 0.001
**Cost**	Δ£	95% CI	Δ£	95% CI	Δ£	95% CI
Intervention						
Linear secular and intervention time effects	0.230	-0.195 to 0.656	-0.150	-0.173 to -0.128	-	-
plus quadratic time effect	0.217	-0.208 to 0.642	-0.241	-0.316 to -0.165	-	-
plus quadratic intervention effects	0.305	-0.186 to 0.796	-0.165	-0.223 to -0.107	$4.35e^{-4}$	-0.001 to 0.002
Age-based counterfactual						
Linear secular and intervention time effects	4.740	3.425 to 6.055	0.034	-0.051 to 0.119	-	-
plus quadratic time effect	4.707	3.389 to 6.025	-0.063	-0.333 to 0.206	-	-
plus quadratic intervention effects	4.607	3.193 to 6.020	0.070	-0.131 to 0.271	-0.002	-0.006 to 0.003
Condition-based counterfactual						
Linear secular and intervention time effects	-3.159	-3.703 to -2.616	-0.010	-0.039 to 0.018	-	-
plus quadratic time effect	-3.137	-3.681 to -2.594	0.166	0.071 to 0.262	-	-
plus quadratic intervention effects	-2.701	-3.333 to -2.069	-0.105	-0.178 to -0.031	0.003	0.001 to 0.005

95% CI; 95% confidence interval, DDDs; Defined daily doses per 100 patients per practice per month, ΔDDDs; change in DDDs, IR; Incidence rate per 100 patients per practice per month, IRR; incidence rate ratio, £s; gross ingredient cost of medicines before any discount per 100 patients per practice per month, Δ£s; change in £

Supplementary file 5 Health board clustering sensitivity analysis

Table S5 (continued) - Quadratic time effects

	**Abolition step change**		**Abolition change in slope**		**Abolition change in slope (quadratic)**	
**Admissions**	ΔIRR	95% C1	ΔIRR	95% CI	ΔIRR	95% CI
Intervention						
Linear secular and intervention time effects	0.807	0.725 to 0.899	0.995	0.993 to 0.998	-	-
plus quadratic time effect	1.033	0.755 to 1.413	1.009	0.993 to 1.026	-	-
plus quadratic intervention effects	0.750	0.669 to 0.842	1.008	1.000 to 1.016	1.000	0.999 to 1.000
Age-based counterfactual						
Linear secular and intervention time effects	0.948	0.897 to 1.001	0.996	0.995 to 0.998	-	-
plus quadratic time effect	1.356	1.160 to 1.585	1.016	1.008 to 1.024	-	-
plus quadratic intervention effects	0.890	0.840 to 0.943	1.007	1.004 to 1.011	1.000	1.000 to 1.000
Condition-based counterfactual						
Linear secular and intervention time effects	1.015	0.858 to 1.200	1.001	0.997 to 1.005	-	-
plus quadratic time effect	1.171	0.730 to 1.880	1.009	0.985 to 1.034	-	-
plus quadratic intervention effects	0.970	0.812 to 1.159	1.009	0.998 to 1.020	1.000	0.999 to 1.000
**Defined Daily Doses (DDDs)**	ΔDDDs	95% CI	ΔDDDs	95% CI	ΔDDDs	95% CI
Intervention						
Linear secular and intervention time effects	6.034	4.935 to 7.133	0.105	0.078 to 0.132	-	-
plus quadratic time effect	8.441	5.179 to 11.702	0.235	0.067 to 0.403	-	-
plus quadratic intervention effects	5.914	4.754 to 7.074	0.131	0.056 to 0.207	-0.001	-0.003 to 0.001
Age-based counterfactual						
Linear secular and intervention time effects	38.373	34.843 to 41.903	0.336	0.250 to 0.421	-	-
plus quadratic time effect	48.598	38.035 to 59.161	0.889	0.344 to 1.433	-	-
plus quadratic intervention effects	35.666	31.986 to 39.347	0.931	0.702 to 1.159	-0.019	-0.025 to -0.012
Condition-based counterfactual						
Linear secular and intervention time effects	6.024	3.729 to 8.319	0.146	0.090 to 0.203	-	-
plus quadratic time effect	14.645	7.802 to 21.488	0.612	0.259 to 0.965	-	-
plus quadratic intervention effects	6.369	3.954 to 8.784	0.101	-0.054 to 0.256	0.002	-0.003 to 0.006
**Cost**	Δ£	95% CI	Δ£	95% CI	Δ£	95% CI
Intervention						
Linear secular and intervention time effects	-0.965	-1.916 to -0.013	-0.100	-0.124 to -0.077	-	-
plus quadratic time effect	-4.315	-7.148 to -1.482	-0.281	-0.427 to -0.135	-	-
plus quadratic intervention effects	-0.773	-1.774 to 0.229	-0.141	-0.205 to -0.076	0.001	-0.001 to 0.003
Age-based counterfactual						
Linear secular and intervention time effects	14.242	10.857 to 17.627	-0.356	-0.438 to -0.275	-	-
plus quadratic time effect	10.647	0.585 to 20.709	-0.551	-1.069 to -0.032	-	-
plus quadratic intervention effects	12.558	9.036 to 16.080	0.024	-0.191 to 0.238	-0.012	-0.018 to -0.006
Condition-based counterfactual						
Linear secular and intervention time effects	1.997	0.791 to 3.203	-0.115	-0.144 to -0.085	-	-
plus quadratic time effect	8.544	4.956 to 12.131	0.239	0.054 to 0.424	-	-
plus quadratic intervention effects	2.256	0.98 to 3.527	-0.184	-0.266 to -0.102	0.002	<0.001 to 0.004

95% CI; 95% confidence interval, DDDs; Defined daily doses per 100 patients per practice per month, ΔDDDs; change in DDDs, IR; Incidence rate per 100 patients per practice per month, IRR; incidence rate ratio, £s; gross ingredient cost of medicines before any discount per 100 patients per practice per month, Δ£s; change in £s

Supplementary file 5 Health board clustering sensitivity analysis

Figure S5 – Plots comparing the linear and quadratic time effects within the interrupted time series analysis

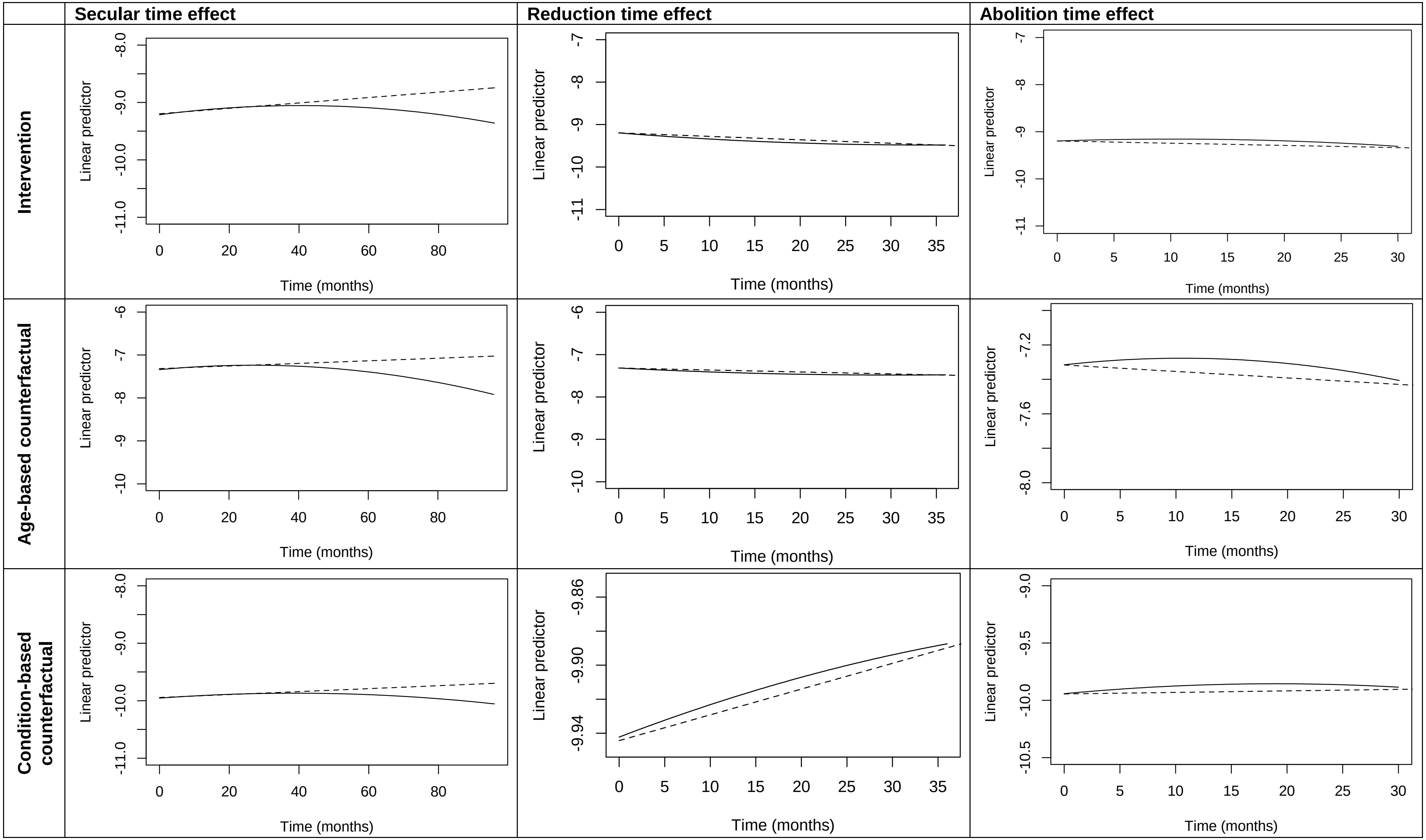

Supplement: Supplementary file 5 [file bmjopen-2017-021318supp005.pdf]
