# Supplementary material for: Impact of abolishing prescription fees in Scotland on hospital admissions and prescribed medicines: an interrupted time series evaluation
Source: BMJ Open. 2018 Dec 18;8(12):e021318. doi: 10.1136/bmjopen-2017-021318 (PMC6303621; doi:10.1136/bmjopen-2017-021318)
Supplement: Author's manuscript [file bmjopen-2017-021318.draft_revisions.pdf]

BMJ Open is committed to open peer review. As part of this commitment we make the peer review history of every article we publish publicly available.

When an article is published we post the peer reviewers' comments and the authors' responses online. We also post the versions of the paper that were used during peer review. These are the versions that the peer review comments apply to.

The versions of the paper that follow are the versions that were submitted during the peer review process. They are not the versions of record or the final published versions. They should not be cited or distributed as the published version of this manuscript.

BMJ Open is an open access journal and the full, final, typeset and author-corrected version of record of the manuscript is available on our site with no access controls, subscription charges or pay-per-view fees (http://bmjopen.bmj.com).

If you have any questions on BMJ Open's open peer review process please email info.bmjopen@bmj.com

BMJ Open

Interrupted time series evaluation of the impact of abolishing prescription fees in Scotland on hospital admissions and prescriptions

Journal:	*BMJ Open*
Manuscript ID	bmjopen-2017-021318
Article Type:	Research
Date Submitted by the Author:	20-Dec-2017
Complete List of Authors:	Williams, Andrew James; University of Exeter, European Centre for Environment and Human Health<br>Henley, W; Institute of Health Research, University of Exeter Medical School, College House, St Luke's Campus, Exeter, Devon, UK, EX1 2LU<br>Frank, John; University of Edinburgh, The Usher Institute of Population Health Sciences and Informatics, College of Medicine and Veterinary Medicine,
Keywords:	universal free prescriptions, administrative data, natural experiment, quasi-experimental, interrupted time series

SCHOLARONE™
Manuscripts

For peer review only

Interrupted time series evaluation of the impact of abolishing prescription fees in Scotland on hospital admissions and prescriptions

Andrew James Williams

European Centre for Environment and Human Health, University of Exeter Medical School, Truro, United Kingdom

William Henley

Institute of Health Research, University of Exeter Medical School, Exeter, United Kingdom

John Frank (corresponding author)

Scottish Collaboration for Public Health Research and Policy, University of Edinburgh, 20 West Richmond Street, Edinburgh EH8 9DX United Kingdom

john.frank@ed.ac.uk, +44(0)131 6 511593

Word count (excluding title page, abstract, references, figures and tables): 3,711

For peer review only

ABSTRACT

Objectives: To identify whether the abolition of prescription fees in Scotland resulted in:

a) increase in the number (cost to NHS) of prescriptions for which there had been a fee;
b) reduction in hospital admissions for conditions related to those medications for which there had been a fee –

when both are compared to prescriptions and admissions for a condition for which prescriptions were historically free.

Design: Retrospective General Practice level interrupted time series analysis using administrative data

Setting: Scotland, United Kingdom

Participants: 796 General Practices across Scotland

Intervention: Reduction in fees per prescribed item from April 2008 leading to the abolition of the fee in April 2011, resulting in universal free prescriptions

Primary and secondary outcome: Hospital admissions recorded in the Scottish Morbidity Record – 01 Inpatient (SMR01) and dispensed prescriptions recorded in the Prescriptions Information System (PIS).

Results: The interrupted time series analysis identified marked step reductions in adult (19-59 years) admissions related to asthma or Chronic Obstructive Pulmonary Disease, compared to older or young people with the same conditions or adults with diabetes mellitus. The prescriptions findings were less coherent and a subsequent sensitivity analysis found that both the admissions and prescriptions data were highly variable above the annual or seasonal level, limiting the ability to interpret the findings of the interrupted time series analysis.

Conclusions: This study did not find any evidence that universal free prescriptions was a demonstrably effective or ineffective policy, in terms of reducing hospital admissions or reducing socioeconomic inequality in hospital admissions, in the context of a universal, publicly administered medical care system, the National Health Service of Scotland.

Key words: prescriptions, admissions, universal free prescriptions, interrupted time series, administrative data, natural experiment, quasi-experimental

For peer review only

ARTICLE SUMMARY

Strengths and limitations of this study

- Administrative data permitted the undertaking of a large long term evaluation of a policy, which otherwise would have been very costly and indeed, perhaps infeasible.
- Data were available for 80% of the practices in Scotland, which could be used to classify those who did and did not experience a change in prescription fees.
- The data were only available at the General Practice not the individual level.
- The time series for both admissions and prescriptions were highly variable, preventing the drawing of specific conclusions from the evaluation.

STUDY PROTOCOL

Available as Supplemental file 1 online.

FUNDING STATEMENT

This work was funded by the Farr Institute @ Scotland, which is funded by the following consortium: Arthritis Research UK, the British Heart Foundation, Cancer Research UK, the Economic and Social Research Council, the Engineering and Physical Sciences Research Council, the Medical Research Council, the National Institute of Health Research, the National Institute for Social Care and Health Research (Welsh Assembly Government), the Chief Scientist Office (Scottish Government Health Directorates), (MRC Grant No: MR/K007017/1). JF and AJW worked for the Scottish Collaboration for Public Health Research and Policy which is funded by the SCPHRP core grant from the Medical Research Council (Grant Number MR/K023209/1) and the Chief Scientist Office of Scotland. WH received support from the National Institute for Health Research (NIHR) Collaboration for Leadership in Applied Health Research and Care (CLAHRC) for the South West Peninsula. The funders played no role in the conceptualisation or realisation of the research and no role in the decision to submit it for publication.

COMPETING INTERESTS

The authors declare that they have no competing interests.

For peer review only - http://bmjopen.bmj.com/site/about/guidelines.xhtml

For peer review only

INTRODUCTION

When the National Health Service was founded in the United Kingdom in 1948, all the services provided were free. However, as early as 1950 expenditure was higher than expected, so a charge (1 shilling) for prescriptions was introduced in 1952.[1] The prescription fee has increased over time and now in England the charge is £8.60 per item.[2] In 1968 a list of medical conditions for which no prescription fee would be charged was introduced; these were conditions which were easily recognisable, lifelong, life threatening and required regular, prescribed medication.[1] Over time further exemptions were identified and the most recent list of exemption criteria is very extensive indeed (Supplemental file 2) [2]. Now, the majority of prescriptions are exempt, meaning that in 2004/05 less than 5% of the cost of pharmaceutical services in Scotland was covered by the prescription fee.[1]

The prescription fee has been described as a 'tax on ill health' (Nicola Sturgeon, then Scottish Health Secretary).[3,4] Although, the exemption criteria (Supplemental file 2) contain many chronic diseases, there are important conditions which are absent such as asthma and degenerative neurological conditions. These omissions have led many to call for the abolition of the fee.[1,5,6] In 2007 Wales was the first UK country to abolish prescription fees, followed by Northern Ireland in 2010 and Scotland in 2011; a fee is still charged in England.[3] Figure 1(a) shows prescription fees in England and Scotland during the time period examined in this study. Patients requiring multiple prescriptions a month could purchase a short-term (3-4 month) or long-term (12 month) Pre-Payment Certificate (PPC) which provided a discount. Alongside the reduction in the prescription fee, Scotland made a marked reduction in PPC shown in Figure 1(b).

Although, the prescription fee only covers a small proportion of the costs of prescription services, abolishing the fee means that the cost needs to be met elsewhere. One of the justifications for abolishing prescription fees has been that this will lead to improved adherence to medication and subsequently reduced medical costs, through less need for expensive secondary medical care, recouping the costs of the prescriptions. Campbell, et al.[7] analysed the impact of changes in pharmacy co-payments in America on asthmatic patients and found that increases as small as $5 resulted in reduced adherence and increased outpatient visits. Kulik, et al.[8] undertook a randomised comparison in American of the health impact of providing patients who had recently had a myocardial infarction with free prescriptions for all

For peer review only - http://bmjopen.bmj.com/site/about/guidelines.xhtml

medications, or only a small selection of cardiovascular medications. It was found that full prescription coverage resulted in significantly improved adherence and trends towards reductions in rates of major vascular events or revascularisation. Furthermore, Booth, et al.[9] in Canada, where all prescriptions are free for those over 65 years of age, found that health inequalities by socioeconomic position were much reduced in this group compared with those under 65 years. The intention of this study was to identify whether the abolition of prescription fees in Scotland resulted in:

a) increase in the number (cost to NHS) of prescriptions for which there had been a fee;
b) reduction in hospital admissions for conditions related to those medications for which there had been a fee.

The comparisons for both prescriptions and admissions were conditions for which prescriptions were historically free. Subsequent research questions were:

c) Was there any change in hospital admissions in the practices which also saw a change in dispensing?
d) How did the effects of fee abolition differ across the socioeconomic spectrum?

METHODS

Retrospective policy analysis As the abolition of prescription fees had already taken place and was not implemented experimentally, it was necessary to use a natural experimental approach: interrupted time series. This required that historical data on policy exposure and outcomes were available.

Data Two large administrative datasets are available in Scotland which made this study feasible; the Prescribing Information System (PIS) and the Scottish Morbidity Record – 01 Inpatient (SMR01). PIS is the database of all community prescribed and dispensed medicines across Scotland. SMR01 is the database of all hospital inpatient admissions across Scotland. Using the unique identifier, the Community Health Index (CHI), it is possible to link individual health records across health databases in Scotland. However, CHI has only been recorded in the PIS since August 2009, and therefore this study was restricted to General Practice-level data. The full time period examined in this study was July 2005 – December 2013 with fee reductions beginning in April 2008 and abolished from April 2011.

For peer review only - http://bmjopen.bmj.com/site/about/guidelines.xhtml

Exposure During the development of the study a group of clinicians and pharmacists were consulted to inform the study design and in particular the definition of exposure to the policy. Starting from the prescription fee exemption criteria (Supplemental file 2), groups for whom prescriptions remained free (counterfactual) and became free needed defining (intervention). Furthermore, the groups needed to be identifiable in the PIS and SMR01 and relate to relatively common conditions for which non-adherence to medication would likely result in the need for secondary medical attention. Subsequently, the subset of pharmaceutical-sensitive ambulatory care-sensitive conditions was used.[10] No data on patient financial situation is recorded in SMR01, which excluded the finance related exemption criteria being used to define intervention and counterfactual populations. Asthma and Chronic Obstructive Pulmonary Disease (COPD) were identified as the most appropriate intervention conditions, with diabetes mellitus being the counterfactual condition, since the medications had been exempt for many years. As well as having a counterfactual group defined by a disease, an additional counterfactual was defined based upon age. The criteria for the intervention and two counterfactual groups is given in Table 1. The need to use the exemption criteria to classify intervention and counterfactuals meant that dispensing data from PIS was used, as exemption is only recorded when the prescription is cashed. This prevented the study's consideration of primary non-adherence, when the patient doesn't cash the prescription.

Table 1 – Intervention and counterfactual group definitions

	**Prescribing Information System (PIS)**	**Scottish Morbidity Record – 01 Inpatient (SMR01)**
**Intervention**	BNF: 3.2 Inhaled steroids<br>Fee: paid<br>Age group: >18 & <60 years	ICD-10: J20, J40X, J41, J42, J43, J44, J45, J46 or J47<br>Age group: >18 & <60 years
**Condition-based counterfactual**	BNF: 6.1.1 or 6.1.2<br>Fee: exempt E - Medical exemption<br>Age group: >18 & <60 years	ICD-10: E10, E11, E12, E13 or E14<br>Age group: >18 & <60 years
**Age-based counterfactual**	BNF: 3.2 Inhaled steroids<br>Fee: exempt A - <16 years, B - ≥16 - ≤18 years and in full time education or C - ≥60 years<br>Age group: <16 years, ≥16 - ≤18 years or ≥60 years	ICD-10: J20, J40X, J41, J42, J43, J44, J45, J46 or J47<br>Age group: <16 years, ≥16 - ≤18 years or ≥60 years

BNF: British National Formulary, ICD-10: International Classification of Disease 10th version

For peer review only - http://bmjopen.bmj.com/site/about/guidelines.xhtml

As the exemption criteria were used to define the intervention and counterfactual groups within the PIS, there was a challenge, in that exemption criteria did not need to be recorded after the fee abolition. Fortuitously, exemption criteria continued to be recorded in many instances. The proportion of prescriptions against each exemption criteria was compared before and after the abolition, to check if reporting had changed. Apart from those receiving free prescriptions due to being aged 60 years or over, reporting of exemption criteria fell markedly, particularly reporting PPCs or full payment. Consequently, assuming that the prevalence of the conditions being studied would not have changed markedly during the pre-post fee-reduction/abolition periods, the proportion eligible for each exemption prior to the policy change was applied to the data after the policy change. This was not an issue for the SMR-01 data, as the variables used to define the groups (Table 1) were not affected by the policy change. The datasets were prepared, cleaned and merged in Stata[11] with the top 1% of prescription data being deleted as these very large values appeared to be errors. In order that the outcomes were comparable between the intervention and counterfactual groups the primary metric of prescriptions used was Defined Daily Doses (DDDs). The cost of the prescriptions was also examined in terms of the gross ingredient cost before any discounts. Number and quantity of prescriptions were also provided in the data set, but were found not to be comparable across intervention and counterfactual groups, so were not analysed.

Interrupted time series analysis The dataset was declared a time series dataset with monthly time periods and any missing time points within practices were filled with zero, assuming no prescriptions or admissions. Admissions included small counts (e.g. number of adult (19-59 years) asthma admissions per practice per month) and therefore these data were analysed using mixed effects Poisson models, offset by practice size to account for variations in practice size. DDDs and costs were transformed into DDDs or cost per 100 patients per practice to account for differences in practice size. The resulting measures were sufficiently normally distributed to be analysed using mixed-effects linear models. Interrupted time series operators were derived in order that any model would fit a new intercept and slope at the time of the intervention, which could be delayed using lags. Calendar month regardless of year was fitted as a categorical variable to account for seasonality. Finally, the data were treated as hierarchical with measurement month (i) nested within general practice (j) and the linear model for costs and DDDs was given by:

For peer review only - http://bmjopen.bmj.com/site/about/guidelines.xhtml

$$y_{ij} = \beta_{0ij} + \beta_1 date_{ij} + \beta_2 jan_{ij} + \beta_3 feb_{ij} + \beta_4 mar_{ij} + \beta_5 may_{ij} + \beta_6 jun_{ij} + \beta_7 jul_{ij} + \beta_8 aug_{ij} + \beta_9 sep_{ij} + \beta_{10} oct_{ij} + \beta_{11} nov_{ij} + \beta_{12} dec_{ij} + \beta_{13} reduction\ period_{ij} + \beta_{14} time\ since\ reductions\ began_{ij} + \beta_{15} abolition\ period_{ij} + \beta_{16} time\ since\ abolition\ began_{ij} + e_j + \mu_{ij}$$

Where y_{ij} is the outcome for practice *j* at time *i* (in months), $date_{ij}$ is the count of months from the beginning of the study period (July 2005), e_j is the difference between the intercept for the estimated overall trend and the intercept for the estimated trend for practice *j*, and μ_{ij} is the difference between the observed outcome value for time *i* at practice *j* and the predicted value, and e_j and μ_{ij} are random effects. Here jan_{ij} to dec_{ij} are dummy variables for each month from January to December, excluding April, which was chosen as the reference category as this was the month in which the policy changes were implemented. $Reduction_{ij}$ *period* and $abolition_{ij}$ *period* are also dummy variables for the periods April 2008 to March 2011, and April 2011 to December 2013 (the end of the study period) respectively. *Time since reductions* $began_{ij}$ and *time since abolition* $began_{ij}$ are counts of months from the beginning to end of the reduction and abolition periods respectively. The meaning of the beta coefficients was: β_0 mean outcome in July 2005 (intercept), β_1 secular trend for time from July 2005, β_{2-12} mean effect for each month excluding April (seasonality), β_{13} change in intercept when the prescription fee was being reduced (reduction step change), β_{14} change in slope (from April 2008 to March 2011) when the prescription fee was being reduced (reduction change in slope), β_{15} change in intercept when the prescription fee was abolished (abolition step change) and β_{16} change in slope (from April 2011) when the prescription fee was abolished (abolition change in slope). The Poisson model for admissions was defined similarly with the logarithm of the admission rate expressed as a linear function of the fixed and random effects, and with the addition of an offset term for the practice size.

Admissions data were analysed with 0, 1, 2 and 3 month lags on the intervention effect. To address research question (c), it was planned that practices where changes in prescriptions and admissions consistent with the hypothesised effect of the policy change be identified, and compared as a secondary analysis. Finally, to address research question (d), practices whose population modal quintile of the Scottish Index of Multiple Deprivation (SIMD) 2012 [12] remained the same

For peer review only - http://bmjopen.bmj.com/site/about/guidelines.xhtml

throughout the study period (to keep the sample size constant) were included in a repetition of the primary analysis stratified by quintile of SIMD. During model development the hierarchical liner time series models were specified with first or second order autoregression or moving averages. The results did not differ substantially between specifications and therefore all the results presented were specified with second order moving average.

RESULTS

Data on prescriptions were available for 796 (80%) practices across Scotland, while admissions data was available for 843 (85%) practices. Across the eight-year study period, practice characteristics will have changed and therefore the characteristics of the practices with prescription data in December 2013 have been presented in Table 2. The majority of practices had 5 or fewer general practitioners, fewer than 10,000 patients, and were in urban areas. Less than 10% of practices had their own dispensary.

For peer review only

For peer review only - http://bmjopen.bmj.com/site/about/guidelines.xhtml

Table 2 – Practice characteristics in December 2013 (n=732)

Characteristic	Summary statistic
Number of GPs	
≤5 general practitioners	447 (61.1%)
>5–≤10 general practitioners	246 (33.6%)
>10 general practitioners	39 (5.3%)
Practice size	
≤5,000 patients	345 (47.1%)
>5,000–≤10,000 patients	305 (41.7%)
>10,000 patients	82 (11.2%)
Dispensing practice	67 (9.2%)
Health Board	
NHS Ayrshire & Arran	37 (5.1%)
NHS Borders	20 (2.7%)
NHS Dumfries & Galloway	26 (3.6%)
NHS Fife	40 (5.5%)
NHS Forth Valley	44 (6.0%)
NHS Grampian	37 (5.1%)
NHS Greater Glasgow & Clyde	215 (29.4%)
NHS Highland	72 (9.8%)
NHS Lanarkshire	60 (8.2%)
NHS Lothian	107 (14.6%)
NHS Orkney	3 (0.4%)
NHS Shetland	4 (0.5%)
NHS Tayside	60 (8.2%)
NHS Western Isles	7 (1.0%)
Modal SIMD quintile of patients	
Quintile 1 (most deprived)	224 (30.6%)
Quintile 2	127 (17.3%)
Quintile 3	155 (21.2%)
Quintile 4	108 (14.8%)
Quintile 5 (least deprived)	118 (16.1%)
Modal Location of patients	
Large Urban Areas	302 (41.3%)
Other Urban Areas	192 (26.2%)
Accessible Small Towns	64 (8.7%)
Remote Small Towns	17 (2.3%)
Very Remote Small Towns	9 (1.2%)
Accessible Rural	61 (8.3%)
Remote Rural	32 (4.4%)
Very Remote Rural	55 (7.5%)

The intercept (constant) and coefficients of the interrupted time series operators for the Poisson and linear mixed effects models of admissions, DDDs and cost are presented in Table 3. Throughout the results, p-values have not been reported and the focus was on estimated effect sizes as the size of the dataset resulted in many statistical but not meaningful significances. The intercept estimates demonstrate that there were pre-existing differences in the level of admissions and prescriptions across the intervention and counterfactual groups at the start of the

For peer review only - http://bmjopen.bmj.com/site/about/guidelines.xhtml

study period (July 2005). The highest rate of admissions, and number (DDDs) and cost of prescriptions were observed in the age-counterfactual group, while the fewest admissions and prescriptions were seen among the intervention group. This demonstrates that asthma and COPD are, as expected, most problematic to the young and old. Despite these differences in quantity, the background time trend in admissions before the abolition of prescription fees is consistent across the groups. The coefficients of the interrupted time series operators are also mostly consistent across groups and increasing degrees of time lag. The exception was that there seemed to have been a significant step reduction in admissions in the intervention group, related to the abolition of prescription fees, although the underlying time trend did not significantly change. The results regarding prescriptions are much less clear, with more marked temporal variability across the study period. However, the largest changes appear in the counterfactual groups, indicating some phenomenon other than the intended effect of the policy change, which we were unable to identify.

For peer review only - http://bmjopen.bmj.com/site/about/guidelines.xhtml

Table 3 – Results of interrupted time series analysis of the impact of reducing and abolishing prescription fees in Scotland on hospital admissions (incidence rate ratios) and prescriptions (defined daily doses and cost)

	**Intercept**		**Monthly change**		**Reduction step change**		**Reduction change in slope**		**Abolition step change**		**Abolition change in slope**	
**Admissions**	IR	95% CI	IRR	95% CI	IRR	95% CI	IRR	95% CI	IRR	95% CI	IRR	95% CI
Intervention	$1.01e^{-2}$	$9.46e^{-3}$ to $1.09e^{-2}$	1.00	1.00 to 1.01	1.03	0.98 to 1.08	0.99	0.99 to 0.99	0.81	0.72 to 0.90	1.00	0.99 to 1.00
Age-c'fact	$6.65e^{-2}$	$6.36e^{-2}$ to $6.95e^{-2}$	1.00	1.00 to 1.00	1.03	1.01 to 1.06	1.00	0.99 to 1.00	0.95	0.90 to 1.00	1.00	0.99 to 1.00
Condition-c'fact	$4.80e^{-3}$	$4.39e^{-3}$ to $5.24e^{-3}$	1.00	1.00 to 1.01	0.95	0.88 to 1.03	1.00	1.00 to 1.01	1.01	0.86 to 1.20	1.00	1.00 to 1.01
**Admissions 1 month lag**	IR	95% CI	IRR	95% CI	IRR	95% CI	IRR	95% CI	IRR	95% CI	IRR	95% CI
Intervention	$1.02e^{-2}$	$9.49e^{-3}$ to $1.09e^{-2}$	1.01	1.00 to 1.01	1.01	0.96 to 1.06	0.99	0.99 to 0.99	0.78	0.71 to 0.87	1.00	0.99 to 1.00
Age-c'fact	$6.74e^{-2}$	$6.45e^{-2}$ to $7.05e^{-2}$	1.00	1.00 to 1.00	1.03	1.01 to 1.06	1.00	0.99 to 1.00	0.95	0.90 to 1.00	1.00	0.99 to 1.00
Condition-c'fact	$4.83e^{-3}$	$4.41e^{-3}$ to $5.28e^{-3}$	1.00	1.00 to 1.00	0.98	0.91 to 1.06	1.00	1.00 to 1.00	1.07	0.91 to 1.26	1.00	1.00 to 1.01
**Admissions 2 month lag**	IR	95% CI	IRR	95% CI	IRR	95% CI	IRR	95% CI	IRR	95% CI	IRR	95% CI
Intervention	$1.02e^{-2}$	$9.51e^{-3}$ to $1.09e^{-2}$	1.00	1.00 to 1.01	1.01	0.96 to 1.06	0.99	0.99 to 0.99	0.79	0.71 to 0.87	1.00	0.99 to 1.00
Age-c'fact	$6.77e^{-2}$	$6.47e^{-2}$ to $7.07e^{-2}$	1.00	1.00 to 1.00	1.04	1.02 to 1.07	1.00	0.99 to 1.00	0.97	0.92 to 1.02	1.00	1.00 to 1.00
Condition-c'fact	$4.85e^{-3}$	$4.44e^{-3}$ to $5.30e^{-3}$	1.00	1.00 to 1.00	0.99	0.92 to 1.07	1.00	1.00 to 1.01	1.10	0.93 to 1.28	1.00	1.00 to 1.01
**Admissions 3 month lag**	IR	95% CI	IRR	95% CI	IRR	95% CI	IRR	95% CI	IRR	95% CI	IRR	95% CI
Intervention	$1.02e^{-2}$	$9.49e^{-2}$ to $1.09e^{-2}$	1.01	1.00 to 1.01	0.99	0.94 to 1.04	0.99	0.99 to 0.99	0.78	0.70 to 0.86	1.00	0.99 to 1.00
Age-c'fact	$6.73e^{-2}$	$6.44e^{-2}$ to $7.04e^{-2}$	1.00	1.00 to 1.00	1.02	1.00 to 1.05	1.00	0.99 to 1.00	0.94	0.90 to 0.99	1.00	1.00 to 1.00
Condition-c'fat	$4.82e^{-3}$	$4.41e^{-3}$ to $5.26e^{-3}$	1.00	1.00 to 1.00	0.97	0.90 to 1.05	1.00	1.00 to 1.01	1.08	0.92 to 1.26	1.00	1.00 to 1.00
**Defined Daily Doses (DDDs)**	DDDs	95% CI	ΔDDDs	95% CI	ΔDDDs	95% CI	ΔDDDs	95% CI	ΔDDDs	95% CI	ΔDDDs	95% CI
Intervention	29.65	28.71 to 30.59	-0.04	-0.06 to -0.02	-0.11	-0.61 to 0.39	0.04	0.02 to 0.07	6.03	4.94 to 7.13	0.10	0.08 to 0.13
Age-c'fact	100.88	98.07 to 103.70	-0.05	-0.11 to 0.01	5.06	3.61 to 6.52	0.68	0.59 to 0.77	38.37	34.84 to 41.90	0.34	0.25 to 0.42
Condition-c'fact	55.61	53.70 to 57.53	0.14	0.10 to 0.18	-1.88	-2.90 to -0.85	-0.05	-0.11 to 0.00	6.02	3.73 to 8.32	0.15	0.09 to 0.20
**Cost**	£s	95% CI	Δ£s	95% CI	Δ£s	95% CI	Δ£s	95% CI	Δ£s	95% CI	Δ£s	95% CI
Intervention	19.36	18.53 to 20.19	0.16	0.15 to 0.18	0.23	-0.19 to 0.66	-0.15	-0.17 to -0.13	-0.96	-1.92 to -0.01	-0.10	-0.12 to -0.08
Age-c'fact	69.92	67.35 to 72.49	0.62	0.56 to 0.68	4.74	3.42 to 6.06	0.03	-0.05 to 0.12	14.24	10.86 to 17.63	-0.36	-0.44 to -0.28
Condition-c'fact	26.35	25.35 to 27.34	0.15	0.13 to 0.17	-3.16	-3.70 to -2.62	-0.01	-0.04 to 0.02	2.00	0.79 to 3.20	-0.11	-0.14 to -0.08

95% CI; 95% confidence interval, c'fact; counterfactual, DDDs; Defined daily doses per 100 patients per practice per month, ΔDDDs; change in DDDs, IR; Incidence rate per 100 patients per practice per month, IRR; incidence rate ratio, £s; gross ingredient cost of medicines before any discount per 100 patients per practice per month, Δ£s; change in £s

For peer review only - http://bmjopen.bmj.com/site/about/guidelines.xhtml

The results of the analysis undertaken to examine whether the impact of the policy differed by socioeconomic position are presented in Table 4. These analyses were undertaken in an endeavour to assess whether the financial exemptions to the prescription fee (Table 2) had an impact on the results, as these exemptions could not be accounted for directly in the main analysis. Only the results for the intervention group have been presented, as individual financial position should not have had an impact on the counterfactual groups. The intercepts of the admissions analyses are consistent with known inequalities, with the highest rate of admissions among the most deprived (Q1), and the lowest rate of admissions in the least deprived (Q5). The results for the interrupted time series operators are similar to the main analysis, with the most marked differences being observed in terms of step changes as opposed to changes in the underlying time trend. Although not statistically significant, there seems to be some evidence of reductions in admissions among the most deprived quintiles (Q1 and Q2) in relation to the fee-reduction policy, compared to the least deprived quintiles (Q4 and Q5). However, any effect is not linear across the SIMD quintiles. Again, the results of the analysis of prescriptions were less coherent. The intercepts of the models demonstrate that most prescriptions were from practices with mostly Q3 and Q4 patients. This makes sense as those in the lower quintiles are more likely to have a financial exemption. These quintiles also demonstrate the largest step increases in DDDs when the fees were abolished, which is consistent with this being an effect of the policy.

For peer review only - http://bmjopen.bmj.com/site/about/guidelines.xhtml

Table 4 - Results of interrupted time series analysis of the impact of reducing and abolishing prescription fees in Scotland on hospital admissions (incidence rate ratios) stratified by quintile of the Scottish Index of Multiple Deprivation

	Intercept		Monthly change		Reduction step change		Reduction change in slope		Abolition step change		Abolition change in slope	
**Admissions**	IR	95% CI	IRR	95% CI	IRR	95% CI	IRR	95% CI	IRR	95% CI	IRR	95% CI
Q1 - most deprived	$1.52e^{-2}$	$1.35e^{-2}$ to $1.71e^{-2}$	1.01	1.01 to 1.01	1.00	0.91 to 1.09	0.99	0.98 to 0.99	0.64	0.52 to 0.78	0.99	0.98 to 0.99
Q2	$1.17e^{-2}$	$9.36e^{-3}$ to $1.47e^{-2}$	1.00	1.00 to 1.01	1.10	0.92 to 1.32	0.99	0.98 to 1.00	0.75	0.50 to 1.12	1.00	0.99 to 1.01
Q3	$9.99e^{-3}$	$7.45e^{-3}$ to $1.34e^{-2}$	1.00	0.99 to 1.01	1.10	0.86 to 1.41	1.00	0.98 to 1.01	1.15	0.68 to 1.94	1.00	0.99 to 1.02
Q4	$6.49e^{-3}$	$4.64e^{-3}$ to $9.07e^{-3}$	0.99	0.98 to 1.00	1.50	1.13 to 1.99	1.00	0.99 to 1.02	1.63	0.89 to 2.99	1.02	1.01 to 1.04
Q5 - least deprived	$6.06e^{-3}$	$4.86e^{-3}$ to $7.54e^{-3}$	1.00	0.99 to 1.00	1.00	0.84 to 1.21	1.00	1.00 to 1.01	1.24	0.85 to 1.82	1.00	0.99 to 1.01
**Admissions 1 month lag**	IR	95% CI	IRR	95% CI	IRR	95% CI	IRR	95% CI	IRR	95% CI	IRR	95% CI
Q1 - most deprived	$1.50e^{-2}$	$1.33e^{-2}$ to $1.69e^{-2}$	1.01	1.01 to 1.02	0.94	0.86 to 1.03	0.99	0.98 to 0.99	0.60	0.49 to 0.73	0.99	0.98 to 0.99
Q2	$1.20e^{-2}$	$9.58e^{-3}$ to $1.51e^{-2}$	1.00	1.00 to 1.01	1.12	0.93 to 1.34	0.99	0.98 to 1.00	0.79	0.53 to 1.16	1.00	0.99 to 1.01
Q3	$9.97e^{-3}$	$7.40e^{-3}$ to $1.34e^{-2}$	1.00	0.99 to 1.01	1.00	0.78 to 1.27	1.00	0.99 to 1.01	0.99	0.60 to 1.65	1.00	0.99 to 1.02
Q4	$6.81e^{-3}$	$4.85e^{-3}$ to $9.56e^{-3}$	0.99	0.98 to 1.00	1.53	1.16 to 0.02	1.00	0.99 to 1.01	1.66	0.91 to 3.00	1.02	1.01 to 1.04
Q5 - least deprived	$6.12e^{-3}$	$4.90e^{-3}$ to $7.64e^{-3}$	1.00	0.99 to 1.00	1.03	0.86 to 1.23	1.01	1.00 to 1.01	1.27	0.87 to 1.85	1.00	0.99 to 1.01
**Admissions 2 month lag**	IR	95% CI	IRR	95% CI	IRR	95% CI	IRR	95% CI	IRR	95% CI	IRR	95% CI
Q1 - most deprived	$1.51e^{-2}$	$1.34e^{-2}$ to $1.70e^{-2}$	1.01	1.01 to 1.01	0.94	0.86 to 1.03	0.99	0.98 to 0.99	0.60	0.50 to 0.73	0.99	0.98 to 0.99
Q2	$1.20e^{-2}$	$9.58e^{-3}$ to $1.51e^{-2}$	1.00	1.00 to 1.01	1.11	0.92 to 1.33	0.99	0.98 to 1.00	0.78	0.54 to 1.15	1.00	0.99 to 1.01
Q3	$1.02e^{-2}$	$7.59e^{-3}$ to $1.37e^{-2}$	1.00	0.99 to 1.01	1.07	0.84 to 1.37	1.00	0.99 to 1.01	1.12	0.68 to 1.84	1.00	0.99 to 1.02
Q4	$6.77e^{-3}$	$4.83e^{-3}$ to $9.48e^{-3}$	0.99	0.98 to 1.00	1.57	1.19 to 2.07	1.00	0.98 to 1.01	1.70	0.95 to 3.05	1.02	1.00 to 1.03
Q5 - least deprived	$6.21e^{-3}$	$4.98e^{-3}$ to $7.75e^{-3}$	0.99	0.99 to 1.00	1.09	0.91 to 1.31	1.00	1.00 to 1.01	1.38	0.95 to 1.99	1.00	0.99 to 1.01
**Admissions 3 month lag**	IR	95% CI	IRR	95% CI	IRR	95%CI	IRR	95% CI	IRR	95% CI	IRR	95% CI
Q1 - most deprived	$1.52e^{-2}$	$1.35e^{-3}$ to $1.71e^{-2}$	1.01	1.01 to 1.01	0.94	0.86 to 1.02	0.99	0.98 to 0.99	0.60	0.50 to 0.73	0.99	0.98 to 0.99
Q2	$1.18e^{-2}$	$9.40e^{-3}$ to $1.48e^{-2}$	1.01	1.00 to 1.01	1.03	0.86 to 1.24	0.99	0.98 to 1.00	0.72	0.50 to 1.05	1.00	0.99 to 1.01
Q3	$1.05e^{-2}$	$7.82e^{-3}$ to $1.41e^{-2}$	1.00	0.99 to 1.00	1.18	0.92 to 1.51	1.00	0.99 to 1.01	1.25	0.76 to 2.05	1.01	0.99 to 1.02
Q4	$6.40e^{-3}$	$4.58e^{-3}$ to $8.96e^{-3}$	0.99	0.98 to 1.00	1.34	1.02 to 1.77	1.00	0.98 to 1.01	1.36	0.77 to 2.39	1.01	1.00 to 1.03
Q5 - least deprived	$6.15e^{-3}$	$4.93e^{-3}$ to $7.67e^{-3}$	1.00	0.99 to 1.00	1.07	0.89 to 1.28	1.00	0.99 to 1.01	1.35	0.94 to 1.94	1.00	0.99 to 1.01
**Defined Daily Doses (DDDs)**	DDDs	95% CI	ΔDDDs	95% CI	ΔDDDs	95% CI	ΔDDDs	95% CI	ΔDDDs	95% CI	ΔDDDs	95% CI
Q1 - most deprived	21.56	20.13 to 22.99	-0.07	-0.10 to -0.04	0.25	-0.50 to 1.01	0.08	0.04 to 0.12	6.23	4.59 to 7.86	0.14	0.10 to 0.18
Q2	29.47	25.42 to 33.51	0.05	-0.03 to 0.12	-1.83	-3.80 to 0.15	-0.05	-0.15 to 0.05	2.09	-2.19 to 6.37	0.03	-0.07 to 0.14
Q3	35.64	31.29 to 39.98	-0.06	-0.18 to 0.06	1.11	-2.07 to 4.28	-0.03	-0.20 to 0.13	7.32	0.27 to 14.37	0.15	-0.02 to 0.32
Q4	37.89	34.33 to 41.46	-0.05	-0.14 to 0.03	0.12	-2.06 to 2.31	0.06	-0.05 to 0.17	7.62	2.87 to 12.38	0.09	-0.03 to 0.21
Q5 - least deprived	30.20	28.03 to 32.37	0.02	-0.02 to 0.06	-1.15	-2.24 to -0.05	$9.05e^{-4}$	-0.06 to 0.06	4.21	1.83 to 6.60	0.01	-0.05 to 0.06
**Cost**	£s	95% CI	Δ£s	95% CI	Δ£s	95% CI	Δ£s	95% CI	Δ£s	95% CI	Δ£s	95% CI
Q1 - most deprived	14.25	12.94 to 15.55	0.07	0.05 to 0.09	0.37	-0.24 to 0.98	-0.06	-0.09 to -0.03	1.02	-0.32 to 2.36	-0.02	-0.05 to 0.02
Q2	21.61	17.63 to 25.58	0.21	0.14 to 0.28	-0.88	-2.67 to 0.90	-0.21	-0.30 to -0.12	-2.63	-6.59 to 1.32	-0.18	-0.28 to -0.09
Q3	22.97	19.33 to 26.60	0.20	0.10 to 0.29	0.83	-1.55 to 3.20	-0.25	-0.38 to -0.13	-1.67	-6.95 to 3.62	-0.08	-0.21 to 0.04

For peer review only - http://bmjopen.bmj.com/site/about/guidelines.xhtml

Q4	24.84	21.53 to 28.15	0.22	0.14 to 0.30	0.14	-1.92 to 2.21	-0.24	-0.35 to -0.13	-2.63	-7.26 to 2.01	-0.19	-0.30 to -0.08
Q5 - least deprived	19.78	17.59 to 21.97	0.20	0.17 to 0.24	-0.78	-1.72 to 0.16	-0.18	-0.23 to -0.14	-2.67	-4.76 to -0.58	-0.18	-0.23 to -0.13

95% CI; 95% confidence interval; DDDs; Defined daily doses per 100 patients per practice per month, ΔDDDs; change in DDDs, IR; Incidence rate per 100 patients per practice per month, IRR; incidence rate ratio, £s; gross ingredient cost of medicines before any discount per 100 patients per practice per month, Δ£s; change in £s, Q1-5; quintiles of the Scottish Index of Multiple Deprivation from most to least deprived.

For peer review only

In response to the incoherence of the findings for DDDs and costs, sensitivity analyses were undertaken, instead of the analyses originally planned to address the third research question (c). Using the Generalized Additive Mixed Models package in R [13-17], the models were fitted without the interrupted time series operators, but fitting 8 knots evenly distributed across the time series. These models allow change points in the time series at times other than those related to the policy to be revealed. Plots of each of the models undertaken as part of the sensitivity analyses are show in Supplemental file 3, with the dates of reduction and abolition overlaid. The admissions data for the condition-counterfactual group, show that following the adjustment for seasonality there was a small but linear increase in diabetes-related admissions among adults. However, the admissions data for the intervention and age-counterfactual groups show marked changes which are neither consistent year to year, nor easily related to the policy changes. The patterns of admissions in both the intervention and age-counterfactual groups are similar. Both the DDDs and cost of prescriptions data for all three groups show similar patterns of increases, with occasional change points. These change points appear to be spaced in time similarly to the reduction and abolition of prescription fees, but around 12 months after the policy changes. The magnitude of the changes in prescribed DDDs and costs are also greater in the counterfactuals than the intervention group, arguing against any causal effects of the intervention.

DISCUSSION

Using Scottish administrative hospital admissions and prescriptions data it was possible to undertake an evaluation of the impact of a major policy change. The evaluation included 80% of the general practices in Scotland and analysed changes over an eight-year period. Interrupted time series analysis offered a natural experimental approach for evaluating the impact of policy changes which occurred on specific dates. There was a marked step reduction in adult asthma or COPD related admissions around the time of the abolition of prescription fees (April 2011), compared to the counterfactual groups. This effect was also consistent with known health inequalities and the prescription fee policy prior to the change. The data on prescriptions were less coherent, with marked changes in the intervention and counterfactual groups. Sensitivity analyses revealed that there was considerable variation in almost all the time series that neither related to the policy change,

For peer review only - http://bmjopen.bmj.com/site/about/guidelines.xhtml

season or annual changes. It was therefore difficult to draw any firm conclusions about the impact of the policy change.

More rigorous and conclusive results would be generated by an experimental study. However, a prospective study of this size and duration would have been considerably more costly, and potentially impossible to fund. Policy changes like the one evaluated are also rarely introduced experimentally. The prescription fee exemption criteria, rather ironically, made this study possible. One challenge to this study was that the prescription fee exemption criteria did not need to be reported following the policy change, so the assumption was made that the proportions eligible for each exemption criteria would not vary markedly in the relatively short period being studied following the policy change. This assumption was supported by the finding that the proportion receiving free prescriptions due to older age remained the same, as these people, possibly through habit, continued to report their exemption. However, the variability in the prescriptions time series limited our ability to draw any firm conclusions regarding prescriptions. It is a weakness of the study that it was only possible to examine General Practice-level, rather than individual-level data. This prevented the assessment of primary non-adherence, and was the result of data not being collected which would permit individual linkage. This linkage is now possible, but not for the years before the policy change. Both SMR-01 and PIS are administrative datasets, collected for billing and reimbursement purposes, and therefore data which is most useful to research is often not collected.

Unlike previous studies into the impact of universal free prescriptions, it has not been possible to identify a clear impact of the policy change in Scotland. The other evaluations of prescription fees have been located in the United States of America and Canada, where the health systems are quite different to the United Kingdom's National Health Service. Kulik, et al.[8] were able to undertake a randomised controlled trial offering post-myocardial infarction patients universal free prescriptions, compared to free prescriptions for medication for the myocardial infarction. The participants in that study had a well-defined need for prescriptions, whereas in the current study – although the intervention group had a long-term condition (asthma or COPD) – the need for regular medication among adults with these conditions will vary considerably between patients. Like the current study, both Campbell, et al.[7] and Booth, et al.[9] used administrative data permitting larger retrospective studies. The study from Campbell, et al.[7] is very similar to the current

For peer review only - http://bmjopen.bmj.com/site/about/guidelines.xhtml

study but examined changes in monthly co-payments. They contrasted the impact of increases of greater or less than $5, which is approximately half the cost of the current English prescription fee. However, this was an increase from the average monthly co-payment of around $17, which is considerably larger than the English prescription fee. Booth, et al.[9] examined the whole diabetic population over 65 years of age, who are more likely to be on numerous medications than the adult (19-59 years of age with asthma or COPD) intervention group in this study. Finally, the data analysed in this study showed that prior to the policy change less than 13% of prescriptions were paid for, including pre-payment certificates. Subsequently, considering the proportion of that 13% that are for acute conditions, such as bacterial infections, this study may have been looking for a needle in a haystack.

Overall, in this study no marked impact of the abolition of prescription fees in Scotland on prescriptions or hospital admissions has been identifiable. However, there were some signs of some impact, specifically a step reduction, within one month, in adult admissions for asthma or COPD. Although, the availability of administrative data made this study possible, it has also brought the biggest limitations to the study. As the data were primarily collected for administrative and financial purposes, often the data were understandably not optimal for this study. This is one of the most significant challenges to the use of big data in research [18-20]. The prescriptions data were particularly messy, even having dropped the top 1% of values. This study also encountered one of the other challenges to research using big data; 'over sensitivity of statistical hypothesis testing' [18,21]. These are at least two of the topics for further research in the field of big data. Based on this study, it is not possible to conclude whether universal free prescriptions is an effective policy in terms of improving health or reducing inequalities. However, neither does the study provide evidence that the policy is ineffective. There has been some concern that not charging for prescriptions could lead to over-use or stock-piling, which for many medications is risky, as well as being a waste of limited resources. The increases in prescriptions in the counterfactual groups may support this hypothesis. Charging a reasonable fee per prescription may limit this; however, the size of the fee required to cover the administrative costs of the payment system may lead to the fee being high enough to inappropriately limit pharmaceutical use. There are still many unanswered questions, which demonstrate that there is a need for rigorous evaluation to be developed alongside the adoption of any new policy.

For peer review only - http://bmjopen.bmj.com/site/about/guidelines.xhtml

AUTHOR CONTRIBUTIONS

AJW contributed to the conceptualisation of the project, undertook all the statistical analysis and drafted the paper. JF contributed to the conceptualisation of the project, supervised the analysis, reviewed and approved the paper. WH supported AJW with the statistical analysis, reviewed and approved the paper. We would like to acknowledge the support from the other members of the Farr Institute @ Scotland Natural Experimental Approaches research group; Corri Black (University of Aberdeen), Chris Dibben (University of Edinburgh), Ruth Jepson (University of Edinburgh), Anne Ludbrook (University of Aberdeen), and Jill Pell (University of Glasgow). Marion Bennie (University of Strathclyde) also supported this project and helped convene the following clinicians and pharmacists to inform the definition of the intervention and control groups: Simon Hurding, Sean MacBride-Stewart and Margaret Ryan. Finally, we would like to thank Stuart McTaggart and Doug Kidd for their help in accessing and understanding the data.

DATA SHARING STATEMENT

The data analysed within this project are administrative data; the Prescribing Information System (PIS) and the Scottish Morbidity Record – 01 Inpatient (SMR01). Anyone wishing to access the data should apply to the electronic Data Research and Innovation Service (eDRIS): www.isdscotland.org/Products-and-Services/EDRIS/index.asp?Co=Y. Applications to access the data will need to be approved by the Public Benefit and Privacy Panel for Health and Social Care (PBPP): www.informationgovernance.scot.nhs.uk/pbpphsc/. Instructions and guidance on how to apply to access the data can be found here: www.informationgovernance.scot.nhs.uk/pbpphsc/home/for-applicants/.

REFERENCES

1 Scottish Executive. Review of NHS prescription charges and exemption arrangements in Scotland - consultation. Edinburgh. Scottish Executive, 2006. Website: http://www.scotland.gov.uk/Resource/Doc/90909/0021837.pdf (accessed 13 December 2017)

For peer review only - http://bmjopen.bmj.com/site/about/guidelines.xhtml

2 NHS Choices. Get help with prescription costs. London. Gov.uk, 2017. Website: https://www.nhs.uk/nhsengland/Healthcosts/pages/Prescriptioncosts.aspx (accessed 13 December 2017)

3 British Broadcasting Corporation. Prescription charges abolished in Scotland. London. British Broadcasting Corporation, 2011. Website: http://www.bbc.co.uk/news/uk-12928485 (accessed 13 December 2017)

4 Roehr B. Cost keeps one in 10 Canadians from filling prescription. *BMJ* 2012;344. doi:10.1136/bmj.e514 [published Online: 20 January 2012]

5 Citizens Advice Scotland. Prescription charges. Edinburgh. The Scottish Association of Citizens Advice Bureaux - Citizens Advice Scotland, 2006. Website: http://www.cas.org.uk/publications/prescription-charges (accessed 13 December 2017)

6 The Scottish Parliament. Summary of responses and conclusions: abolition of NHS prescription charges (Scotland) consultation. Edinburgh. The Scottlish Parliament, n.d. Website: http://www.scottish.parliament.uk/S2_MembersBills/Final%20proposals/abolition-prescription-charges-response.pdf (accessed 13 December 2017)

7 Campbell JD, Allen-Ramey F, Sajjan SG, et al. Increasing pharmaceutical copayments: impact on asthma medication utilization and outcomes. *Am J Manag Care* 2011;17:703-10.

8 Kulik A, Desai NR, Shrank WH, et al. Full prescription coverage versus usual prescription coverage after coronary artery bypass graft surgery: analysis from the post-myocardial infarction Free Rx Event and Economic Evaluation (FREEE) randomized trial. *Circulation* 2013;128:S219-S25. doi:10.1161/circulationaha.112.000337 [published Online: 20 September 2013]

9 Booth GL, Bishara P, Lipscombe LL, et al. Universal drug coverage and socioeconomic disparities in major diabetes outcomes. *Diabetes Care* 2012;35:2257-64. doi:10.2337/dc12-0364 [published Online: 23 October 2012]

10 Purdy S, Griffin T, Salisbury C, et al. Ambulatory care sensitive conditions: terminology and disease coding need to be more specific to aid policy makers and clinicians. *Public Health* 2009;123:169-73. doi:10.1016/j.puhe.2008.11.001 [published Online: 13 January 2009]

For peer review only - http://bmjopen.bmj.com/site/about/guidelines.xhtml

11 Stata statistical software: release 14 [program]. College Station, TX: StataCorp LP, 2015.

12 Scottish Government. The Scottish Index of Multiple Deprivation. Edinburgh. Scottish Government, 2017. Website: http://www.gov.scot/Topics/Statistics/SIMD (accessed 13 December 2017)

13 Wood SN. Thin plate regression splines. *J R Stat Soc Series B Stat Methodol* 2003;65:95-114. doi:10.1111/1467-9868.00374 [published Online: 28 January 2003]

14 Wood SN. Stable and efficient multiple smoothing parameter estimation for generalized additive models. *J Am Stat Assoc* 2004;99:673-86. doi:10.1198/016214504000000980 [published Online: 01 January 2012]

15 Wood SN. Fast stable restricted maximum likelihood and marginal likelihood estimation of semiparametric generalized linear models. *J R Stat Soc Series B Stat Methodol* 2011;73:3-36. doi:10.1111/j.1467-9868.2010.00749.x [published Online: 14 September 2010]

16 Wood SN. *Generalized additive models: an introduction with R*. Second edition. ed. Boca Raton, FL: CRC Press/Taylor & Francis Group, 2017.

17 Wood SN, Pya N, Säfken B. Smoothing parameter and model selection for general smooth models. *J Am Stat Assoc* 2016;111:1548-63. doi:10.1080/01621459.2016.1180986 [published Online: 04 January 2017]

18 Stricker BH. Epidemiology and 'big data'. *Eur J Epidemiol* 2017;32:535-36. doi:10.1007/s10654-017-0294-3 [published Online: 17 August 2017]

19 The Academy of Medical Sciences. Improving the health of the public by 2040: optimising the research environment for a healthier, fairer future. London. The Academy of Medical Sciences, 2016. Website: http://www.acmedsci.ac.uk/viewFile/57e4b532a4308.pdf (accessed 13 December 2017)

20 Royal Statistical Society. The opportunities and ethics of big data. London. Royal Statistical Society, 2016. Website: http://www.rss.org.uk/Images/PDF/influencing-change/2016/rss-report-opps-and-ethics-of-big-data-feb-2016.pdf (accessed 13 December 2017)

21 Kitchin R. Big Data, new epistemologies and paradigm shifts. *Big Data Soc* 2014;1:2053951714528481. doi:10.1177/2053951714528481 [published Online: 01 April 2014]

For peer review only

Figure 1 – Prescription fees in Scotland compared to England; (a) fee per item, (b) equivalent number of items purchased per month using prepayment certificates

For peer review only

For peer review only - http://bmjopen.bmj.com/site/about/guidelines.xhtml

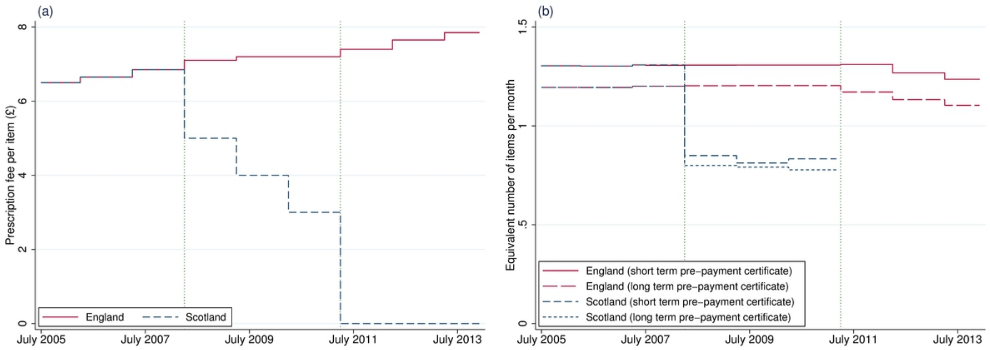

Figure 1 – Prescription fees in Scotland compared to England; (a) fee per item, (b) equivalent number of items purchased per month using prepayment certificates

101x36mm (300 x 300 DPI)

Supplemental file 1 Study protocol

Farr Institute @ Scotland – NEA group / SCPHRP
Andrew James Williams

Natural Experiment

'Universal' Free Prescriptions in Scotland

eDRIS project number: ED13-0487
Primary contact: Andrew James Williams
Other team members: John Frank, Ruth Jepson, Jill Pell, Chris Dibben, Anne Ludbrook and Marion Bennie.

Background

Following devolution each of the four nations of the United Kingdom (UK) (England, Wales, Scotland and Northern Ireland) have taken different approaches to subsidising and funding prescribed medications (British Broadcasting Corporation, 2011). Only England continues to charge a prescription fee which stands at £7.85 in 2013 (Department of Health, 2013). When Scotland abolished the prescription charge the then Health Secretary, Nicola Sturgeon, described prescription fees as 'a tax on ill-health' (British Broadcasting Corporation, 2011).

For those with chronic conditions one of the purposes of pharmaceuticals is to control the condition and subsequently reduce the need for hospitalisation and more invasive and costly intervention. Purdy et al. (2009) have defined a list of conditions sensitive to ambulatory care which are listed in Appendix 1. The appropriate management and treatment of these conditions in primary care should prevent the need for admission into secondary care (Purdy et al., 2009). The success of pharmaceutical treatments is dependent upon the patient's adherence to the dosage regimen prescribed by the practitioner. Kardas et al. (2013) recently reviewed the determinants of non-adherence. Seven hundred and seventy one determinants of whether someone initiated and/or persisted with a medication were identified which fell into the following five broad clusters; socioeconomic-related, healthcare team- and system-related, condition-related, therapy-related, and patient-related (Kardas et al., 2013). Among the socioeconomic cluster the eight studies which examined cost, all found higher cost to be associated with poorer adherence (Kardas et al., 2013). Therefore, the economic logic behind the abolition of prescription fees is that adherence will improve, and therefore hospital admissions will decrease reducing secondary care costs, which hopefully compensate for the additional cost to the state of the prescriptions (Figure 1).

Previous research has tended to focus on elements of the logic model rather than the whole. Linnet et al. (2013), Yin et al. (2008) and Zhang et al. (2008) focussed upon the first half of the logic model, the effect of prescription fee policies on the dispensing of pharmaceuticals with mixed results. Yin et al. (2008) and Zhang et al. (2008) found that the introduction of prescription subsidies (Medicare part D) for the elderly in America resulted in an increased number of prescriptions and reduced out-of-pocket expenses for the patient. Whereas Linnet et al. (2013) found that within Iceland a small increase in prescription charge did not significantly affect whether a patient cashed/filled a prescription. Booth et al. (2012) when examining the socioeconomic disparities in diabetes mellitus related morbidity and mortality found that the socioeconomic disparities greatly reduced in each of the outcomes studied for those over 65 years of age (Booth et al., 2012). Within Ontario, Canada (the location of the study) those over the age of 65 receive free prescriptions and therefore the decreased socioeconomic disparities was attributed to this policy (Booth et al., 2012). Booth et al. (2012) employed a number of

For peer review only - http://bmjopen.bmj.com/site/about/guidelines.xhtml

Supplemental file 1 Study protocol

Farr Institute @ Scotland – NEA group / SCPHRP
Andrew James Williams

statistical techniques to test this hypothesis (sensitivity analyses) and continued to find the decreased disparities associated with the free prescription policy. Campbell et al. (2011) and Kulik et al. (2013) employed more experimental study designs to test the logic model. Kulik et al. (2013) undertook a randomised controlled trial into the effect of offering free prescriptions to patients immediately following a myocardial infarction and found improved adherence and reduced patient out-of-pocket expenses and a trend towards improved clinical outcomes. Campbell et al. (2011) used linked data within a natural experimental approach to examine the effect of prescription costs for those with asthma. It was found that those experiencing increases in prescription charges (co-payment) ≥$5(US) were significantly less adherent than those experiencing increases of <$5 and also had higher healthcare usage (Campbell et al., 2011).

Figure 1 – Logic model for study

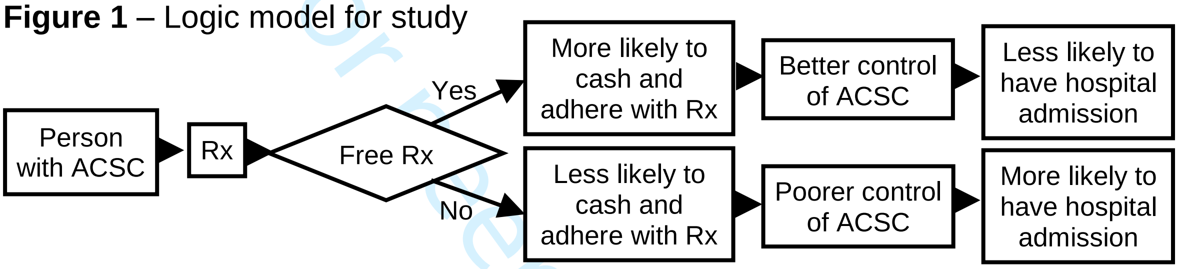

ACSC; ambulatory case sensitive condition, Rx; prescription

Subsequently, there is some evidence that reduced or free prescriptions have positive effects on adherence and health. However, the studies so far have focused upon certain age groups or conditions and yet within Wales, Northern Ireland and Scotland prescriptions are free for all. Three years after the abolition of prescription fees in Wales a process evaluation was published by the health authority (NHS Wales, 2010). This evaluation found that the abolition of prescription fees did not result in a change in the number of prescriptions and their cost to the state. Jill Pell and colleagues are undertaking a similar study examining the first half of the logic model (Figure 1) in Scotland. However, to the best of our knowledge there have been no studies within the UK to examine the whole logic model in relation to the universal free prescription policy.

Aims

To identify whether the stepped reduction and abolition of prescription fees in Scotland resulted in:

a) increase in the number (cost to NHS) of prescriptions for inhaled steroids;
b) reduction in hospital admissions for asthma or chronic obstructive pulmonary disease (COPD).

When compared to prescriptions and admissions for a condition for which prescriptions were historically free (diabetes mellitus) and or those who received free prescriptions due to age.

Moving of from these preliminary aims more refined analysis is planned to address these two further questions.

c) Was any change in hospital admissions for asthma or COPD in the practices which also saw a change in prescribing/dispensing?
d) How did the effect differ across the socioeconomic spectrum?

For peer review only - http://bmjopen.bmj.com/site/about/guidelines.xhtml

Supplemental file 1 Study protocol

Farr Institute @ Scotland – NEA group / SCPHRP
Andrew James Williams

Proposal

As a policy the abolition of prescription fees was not introduced experimentally (e.g. with randomisation), and subsequently we propose to address the above aims using time series of routinely collected data as a natural experiment (Craig et al., 2011). The primary sources for the routinely collected data will be the Scottish Morbidity Record (SMR01) and the Prescriptions Information System (PIS). Through extensive discussion with the Principle Pharmacist (Stuart McTaggart) at NHS National Services Scotland, it has been established that individual-level data suitable for this project are not currently available in the PIS. The primary reasons that individual-level data from the PIS cannot be used are the inability to calculate measures of adherence (Fairman and Matheral, 2000, Lehmann et al., 2013), and the fact that currently individual-level data are not available prior to August 2009 when Scotland had already begun reducing prescription fees. Subsequently, we propose to proceed with a practice-level study as outlined below. Currently, the proposed study is limited to Scotland, although through the Farr network a UK wide study may be considered at a later date.

Critical to any natural experiment is the identification of the population who were/are effected by the policy (the intervention sample) and appropriate counterfactual sample(s) (Craig et al., 2011). As prescription fees were reduced and ultimately abolished contemporaneously across Scotland and the data being used only exist in Scotland there is no location-based contemporary counterfactual. However, the existence of prescription fee exemption criteria (Appendix 2) prior to the abolition of fees permits the identification of the intervention and counterfactual samples. Although, as following the abolition of prescription fees there was no need to record exemption criteria, the samples also had to remain identifiable within the PIS and SMR01. Furthermore, as only one exemption criteria needed to be recorded when collecting a prescription, it was and will remain necessary throughout this project to remain aware that although marked exempt for one reason the patient may have also met other exemption criteria.

The exemption criteria (Appendix 2) fall into three groups, age- condition- and income-related all of which could have formed the basis of counterfactual samples. The income-related exemptions group may have permitted a regression discontinuity design, however, it would not be possible to identify this sample following the abolition of fees and consequently no income-based counterfactual was sought. The age-related exemption criteria would form an age-based counterfactual sample. This is made possible as prior to the abolition of fees the PIS exemptions category data would identify this sample and following the fee abolition individual-level PIS data could inform the identification of this group. Those aged 16-18 years in full time education also received free prescriptions (Appendix 2); as this subpopulation can only be identified prior to the abolition of fees they will initially be examined separately to ascertain what approach is most appropriate for this subpopulation. However, wanting to strengthen the causal inference it is planned to examine dispensed prescriptions as a mediator of change in health (admissions) and consequently it is necessary to identify suitably sensitive and specific condition medication pairings. Thus, the condition-related exemption criteria were crucial. These pairings needed to meet the following requirements;

- The condition needed to be chronic (e.g. not ear, nose and throat infections).

For peer review only - http://bmjopen.bmj.com/site/about/guidelines.xhtml

Supplemental file 1 Study protocol

Farr Institute @ Scotland – NEA group / SCPHRP
Andrew James Williams

- The condition needed to be sensitive to long term pharmaceutical treatment specifically not just ambulatory care in general (e.g. not dental complications).
- Relatively minor changes in adherence (e.g. missed tablets) to the treatment for the condition needed to result in the need for hospital attention (e.g. not schizophrenia or epilepsy).
- The condition needed to be sufficiently prevalent across the life-course to provide sufficient power for the analysis (e.g. not senility/dementia).
- The intervention condition needed to not be a condition-related exemption from prescription fees (Appendix 2) or commonly co-occurring with a condition on that list (e.g. not diabetes mellitus or any of the cardiovascular conditions).
- The counterfactual condition needed to be a condition-related exemption from prescription fees (e.g. diabetes mellitus).
- The pharmaceutical treatment (medicine) needs to be fairly specific to the condition (e.g. not congestive heart failure).

The final samples are outlined in the box below, with asthma/COPD selected as the intervention condition. Asthma/COPD had been noted for their absence from the exemption criteria in the Scottish public consultation about the abolition of prescription fees (Scottish Executive, 2006, The Scottish Parliament, n.d.). These samples have been defined after extensive consultation with expert pharmacists and general practitioners.

**Sample**	**Eligibility criteria**
Intervention	Those aged ≥16-<60 years that had paid for dispensed prescriptions for steroid inhalers which is assumed to indicate diagnosis with asthma or chronic obstructive pulmonary disease (COPD).
Age-based counterfactual	Those age <16 or ≥60 years that had dispensed prescriptions for steroid inhalers which is assumed to indicate diagnosis with asthma or COPD.
Condition-based counterfactual	Those that had dispensed prescriptions for diabetes mellitus treatments which are assumed to indicate diagnosis with said condition.

As only one exemption criteria would have been recorded for each prescription it is not possible to sub-divide the condition-based counterfactual by age. As previously discussed the analysis is forced to examine practice- rather than individual-level data and subsequently the data will be numbers/proportions of relevant prescriptions/admissions for each practice.

Analysis

The studies discussed in the penultimate paragraph of the Background to this document employed a number of statistical methods ranging from the simple univariable analysis to complicated hierarchical multivariable models taking in techniques such as logistic regression (Booth et al., 2012, Kulik et al., 2013, Yin et al., 2008, Zhang et al., 2008) and Cox proportional hazard models (Booth et al., 2012, Kulik et al., 2013). The data to be collected, linked and analysed in the current

For peer review only - http://bmjopen.bmj.com/site/about/guidelines.xhtml

project is outlined in Appendix 3, the intention being to produce a panel dataset. For each practice there will be annually 'fixed' socio-demographic data from the General Practice dataset, and monthly (January 2000-December 2013) admissions and prescriptions data. Analysis will be undertaken in Stata (StataCorp, 2009) and will take the format of time series and mediation analysis.

The primary health outcome will be hospital admissions for the respective conditions. The PIS extract dataset will provide outcome data on the potential mediators of any change in the primary outcome. Without individual data and the ability to measure adherence, a number of prescription related metrics will be evaluated as outcomes and mediators. The following metrics will be examined for each of the medication groups (inhaled steroids and diabetes treatments);

- Number of dispensed prescriptions,
- Quantity dispensed,
- Cost of the items dispensed,
- Number of Defined Daily Doses (DDDs) dispensed.

Including the admissions, but excluding costs all the outcomes are count data and consequently will be analysed as such (Poisson regression Generalised Estimating Equations (GEE) or Mixed Models). Cost will be analysed as a continuous outcome. Initially some of the outcomes may be turned into ratios (e.g. DDDs per prescription or prescriptions per general practitioner) in order to smooth the data and account for differences in practice size, to identify the most appropriate set of outcomes. Auto-regression, integration (differencing) and moving average (ARIMA) adjustments will also be explored, to smooth the data and in particular account for the seasonality of dispensing.

Figure 2 – Mediation model for analysis

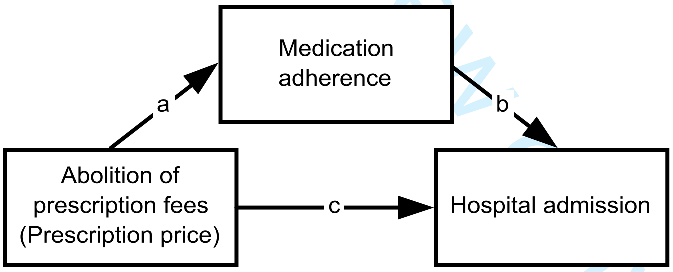

Taking the study aims and the logic model (Figure 1) Figure 2 illustrates the mediation model to be examined through the analysis. The studies discussed in the background provide evidence of relationships **a** and **b** in Figure 2. The preliminary aims of this study (study aims a and b) will be addressed by constructing separate time series of prescriptions and admissions in order to test relationships **a** and **c**. As a practice-level study it will not be possible to directly test relationship **b**. As with previous studies (e.g. Yin et al., 2008, Booth et al., 2012) splines and knots will be employed in order to assess whether changes in admissions and/or prescriptions were contemporary with the reductions and eventual abolition of fees. Furthermore, the use of splines and knots will permit some quantification of any effect of the policy change. The admissions time series will then be adjusted for the prescriptions time series (study aim c), in order to assess the extent to which relationship **c** remains, which will provide information on the nature of relationship **b**. Should relationship **c** remain after adjusting for prescriptions, it would indicate that within the limitations of

For peer review only - http://bmjopen.bmj.com/site/about/guidelines.xhtml

this study the policy change had not impacted on health. These analytical steps should strengthen to the study findings.

Given the extent of exemptions criteria (Appendix 2), during the Scottish public consultation it was questioned who would benefit from abolishing the fee (Scottish Executive, 2006, The Scottish Parliament, n.d.). The exemption criteria (Appendix 2) were (and still are in England) considered to be protecting the poorest and sickest who would struggle to pay. It was felt that a reappraisal of the condition-related exemption criteria to consider including conditions like asthma, COPD, dementia might be a sufficient change. As it was, prior to the abolition of prescription fees around 90% of prescriptions were exempt from payment (Personal communication with S. McTaggart, 01/04/2014). In order to examine for the differential impact of the policy across the socioeconomic spectrum (study aim d), the effect of adjusting or stratifying the time series by deprivation and other General Practice characteristics will be explored.

Special cases: As previously discussed those aged 16-18 years will initially be examined separately. Similarly, there is another group of patients who will initially be examined separately. Prior to the abolition of fees, those with chronic conditions were able to purchase discount pre-payment certificates. The price of these certificates changed in line with the reduction in fees. Data published in 2009 demonstrated that there had been a significant increase in the use of pre-payment certificates following the initial reduction in prescription fees (The Scottish Government, 2009). That finding and the different financial decisions related to pre-payment certificates have necessitated the initial examination of this group separately. The use of a pre-payment certificate was captured within the exemptions data within the PIS.

Potential impact

From a research perspective this project would add to the literature on the importance of cost as a determinant of non-adherence with prescribed medications. Given the large number of exemptions from prescription fees prior to the policy change (see Rationale) some have questioned whether universal free prescriptions are just benefiting the less deprived. This study should reveal what the implications of universal free prescriptions have been for those of different socioeconomic status. Some have also questioned whether having to pay for a prescription is an incentive to adherence, although there does not appear to be evidence to back this up, this needs to be considered within this project, the results of which might support or refute that claim. Politically, the project will evaluate an important and possibly costly policy and provide data on the ‘effects’ of the policy. In particular, should a time arise that free prescriptions for all cannot be funded by the state; the stratified component of this project might suggest conditions or circumstances for which free prescriptions are cost effective.

For peer review only - http://bmjopen.bmj.com/site/about/guidelines.xhtml

Supplemental file 1 Study protocol

Farr Institute @ Scotland – NEA group / SCPHRP
Andrew James Williams

References

Booth, G. L., Bishara, P., Lipscombe, L. L., Shah, B. R., Feig, D. S., Bhattacharyya, O. & Bierman, A. S. 2012. Universal drug coverage and socioeconomic disparities in major diabetes outcomes. *Diabetes Care,* 35**,** 2257-2264.

British Broadcasting Corporation. 2011. *Prescription charges abolished in Scotland* [Online]. London: British Broadcasting Corporation. Available: http://www.bbc.co.uk/news/uk-12928485 [Accessed 24 September 2013].

Campbell, J. D., Allen-Ramey, F., Sajjan, S. G., Maiese, E. M. & Sullivan, S. D. 2011. Increasing pharmaceutical copayments: impact on asthma medication utilization and outcomes. *Am J Manag Care,* 17**,** 703-10.

Craig, P., Cooper, C., Gunnell, D., Haw, S., Lawson, K., Macintyre, S., Ogilvie, D., Petticrew, M., Reeves, B., Sutton, M. & Thompson, S. 2011. *Using natural experiments to evaluate population health interventions: guidance for producers and users of evidence,* London, Medical Research Council. Available: http://www.mrc.ac.uk/documents/pdf/natural-experiments-guidance/ [Accessed 26 February 2014]

Department of Health. 2013. *NHS charges from April 2013 announced* [Online]. London: GOV.UK. Available: https://www.gov.uk/government/news/nhs-charges-from-april-2013-announced [Accessed 15 October 2013].

Fairman, K. & Matheral, B. 2000. Evaluating medication adherence: which measure is right for your program? *Journal of Managed Care Pharmacy,* 6**,** 499-506.

Kardas, P., Lewek, P. & Matyjaszczyk, M. 2013. Determinants of patient adherence: a review of systematic reviews. *Frontiers in Pharmacology,* 4(91).

Kulik, A., Desai, N. R., Shrank, W. H., Antman, E. M., Glynn, R. J., Levin, R., Reisman, L., Brennan, T. & Choudhry, N. K. 2013. Full prescription coverage versus usual prescription coverage after coronary artery bypass graft surgery: analysis from the post-myocardial infarction Free Rx Event and Economic Evaluation (FREEE) randomized trial. *Circulation,* 128**,** S219-S225.

Lehmann, A., Aslani, P., Ahmed, R., Celio, J., Gauchet, A., Bedouch, P., Bugnon, O., Allenet, B. & Schneider, M. P. 2013. Assessing medication adherence: options to consider. *Int J Clin Pharm*, 36(1):55-69.

Linnet, K., Halldórsson, M., Thengilsdóttir, G., Einarsson, Ó. B., Jónsson, K. & Almarsdóttir, A. B. 2013. Primary non-adherence to prescribed medication in general practice: lack of influence of moderate increases in patient copayment. *Family Practice,* 30**,** 69-75.

NHS Choices. 2013. *Get help with prescription costs* [Online]. London: GOV.UK. Available: http://www.nhs.uk/NHSEngland/Healthcosts/Pages/Prescriptioncosts.aspx [Accessed 15 October 2013].

NHS Wales 2010. *Helping to improve Wales's health: free prescriptions three years on,* Cardiff, NHS Wales. Available: http://www.wales.nhs.uk/documents/prescriptions-report-three-years.pdf [Accessed 25 September 2013]

Purdy, S., Griffin, T., Salisbury, C. & Sharp, D. 2009. Ambulatory care sensitive conditions: terminology and disease coding need to be more specific to aid policy makers and clinicians. *Public Health,* 123**,** 169-173.

Scottish Executive. 2006. *Review of NHS prescription charges and exemption arrangements in Scotland - consultation* [Online]. Edinburgh: Scottish Executive. Available:

For peer review only - http://bmjopen.bmj.com/site/about/guidelines.xhtml

Supplemental file 1 Study protocol

http://www.scotland.gov.uk/Resource/Doc/90909/0021837.pdf [Accessed 19 February 2014].
StataCorp 2009. Stata statistical software: release 11. College Station, TX: StataCorp LP.
The Scottish Government. 2009. *News; prescription charges* [Online]. Edinburgh: The Scottish Government. Available: http://www.scotland.gov.uk/News/Releases/2009/01/28095040 [Accessed 28 January 2014].
The Scottish Parliament. n.d. *Summary of responses and conclusions: abolition of NHS prescription charges (Scotland) consultation* [Online]. Edinburgh: The Scottish Parliament. Available: http://www.scottish.parliament.uk/S2_MembersBills/Final%20proposals/abolition-prescription-charges-response.pdf [Accessed 19 February 2014].
Yin, W., Basu, A., Zhang, J. X., Rabbani, A., Meltzer, D. O. & Alexander, G. C. 2008. The effect of the Medicare Part D prescription benefit on drug utilization and expenditures. *Ann Intern Med,* 148**,** 169-77.
Zhang, J. X., Yin, W., Sun, S. X. & Alexander, G. C. 2008. The impact of the Medicare Part D prescription benefit on generic drug use. *J Gen Intern Med,* 23**,** 1673-8.

Appendix 1: Ambulatory case sensitive conditions Purdy et al. (2009)

Angina	Nutritional deficiency	Hypokalaemia	Stroke
Asthma	Hypertension	Low birth weight	Pyelonephritis
Cellulitis	Failure to thrive	Neuroses	Diabetes complications
Dental complications	Ruptured appendix	Tuberculosis	Gangrene
Constipation	Deliberate self-harm	Senility/dementia	Schizophrenia
Congestive heart failure	Fractured proximal femur	Influenza and pneumonia	Migraine/acute headache
Iron-deficiency anaemia	Alcohol-related disease	Atrial fibrillation/flutter	Convulsions and epilepsy
Dehydration and gastroenteritis	Perforated/bleeding ulcer	Ear, nose and throat infections	Pelvic inflammatory disease
Other vaccine-preventable diseases	Dyspepsia and other stomach function disorders	Chronic obstructive pulmonary disease	Peripheral vascular disease

Appendix 2: Criteria exempting patients from paying prescription fees in England and Scotland prior to April 2011 (NHS Choices, 2013).

- Condition-related
  - Cancer (from 2009)
  - Those on renal dialysis with a permanent fistula
  - Pregnant
  - Contraceptives
  - Sexually transmitted infection
  - Tuberculosis

For peer review only - http://bmjopen.bmj.com/site/about/guidelines.xhtml

  - Hypoadrenalism for which specific substitution therapy is essential
  - Diabetes insipidus or other forms of hypopituitarism
  - Diabetes mellitus, except where treatment is by diet alone
  - Hypoparathyroidism
  - Myasthenia gravis
  - Myxoedema
  - Epilepsy requiring continuous anticonvulsive therapy
  - A continuing physical disability which means the person cannot go out without the help of another person.
- Age-related
  - are 60 or over
  - are under 16
  - are 16-18 and in full-time education
- Means tested, and those in receipt of the following benefits ('income-related')
  - Income Support
  - Income-based Jobseeker's Allowance
  - Income-related Employment and Support Allowance
  - Pension Credit Guarantee Credit
  - Universal Credit
  - Those in receipt of a war pension

Appendix 3: Data
The final study dataset will be compiled from three separate datasets. Two of these datasets will be extracts from the Scottish Morbidity Record (SMR01) and Prescriptions Information System (PIS). The third dataset will be the General Practice data compiled from data published by ISD Scotland (http://www.isdscotland.org/Health-Topics/General-Practice/) and more historical data provided through eDRIS. The General Practice dataset will be linked by the researcher using the practice code. The SMR01 and PIS data extracts will be linked by the researcher using the practice code, year and month. The General Practice dataset will then be linked onto the linked SMR01 and PIS data, and the data rearranged in order that each month of SMR01 and PIS data will only have the relevant years general practice descriptive variables. The final dataset should be in the form of panel data for the time series analysis. The variables in each of the three datasets are listed below.

N.B. Gender and ethnicity data are not available in the practice-level PIS and have subsequently not been sought in the other data.

For peer review only - http://bmjopen.bmj.com/site/about/guidelines.xhtml

Supplemental file 1 Study protocol

Farr Institute @ Scotland – NEA group / SCPHRP
Andrew James Williams

General Practice dataset – by practice and year

Variable	Details
NHS board (code)	Unique single alphabetical character for each health board (publically available)*
NHS board (name)	Name of each health board (publically available)*
Practice Code	The unique NHS Scotland code for the GP Practice (publically available)
Practice name	Name of each General Practice (publically available)*
Number of General Practitioners	(requested for each year through eDRIS)
Dispensing practice	Identifier of practices where medicines are dispensed as well as prescribed (requested for each year through eDRIS)
Contract type	Indicator of which type of contract the practice has with the NHS (requested for each year through eDRIS)
Practice List Size	Total number of registered patients each year (publically available for the years 1999-2013)
Age profile of practice list	Number of patients in each of the following age categories; <16, 16-18, 18-60, ≥60 years (requested for each year through eDRIS)
pc_most_dep15	Percentage of practice patients living in datazones defined as the 15% most deprived (population weighted) (publically available from 2005-2013, earlier years requested through eDRIS)
quint1 – quint5	Number of practice patients matched to the relevant first through fifth quintile of deprivation (publically available from 2005-2013, earlier years requested through eDRIS)
prac_ur8	Urban/rural classification of the postcode of the General Practice (publically available from 2005-2013, earlier years requested through eDRIS)
urban1 – urban8	Number of practice patients in each of the eight urban/rural classifications (publically available from 2005-2013, earlier years requested through eDRIS)
unmatch	Number of practice patients who could not be matched to an urban/rural classification (publically available from 2005-2013, earlier years requested through eDRIS)
mode_ur8	Modal urban/rural classification of the practice patients (publically available from 2005-2013, earlier years requested through eDRIS)

*These data are for linking and/or clustering purposes and would be anonymised in any publications.

For peer review only

Supplemental file 1 Study protocol

Farr Institute @ Scotland – NEA group / SCPHRP
Andrew James Williams

Scottish Morbidity Record (SMR01) extract dataset – by practice, year and month

**Variable**	**Details**
Practice Code	The unique NHS Scotland code for the GP Practice
Year	The year to which the data relates (2000-13)
Month	The month to which the data relates
Diagnosis	The diagnosis (based on DIAGNOSIS 1) to which the data relates (ALL admissions, Asthma/COPD† or Diabetes‡
Age group	The age banding (0-15, 16-18, 19-59, 60+ years) to which the data relates
Admissions	The total number of admissions for practice patients

†International Classification of Disease 10 codes J20, J40X, J41, J42, J43, J44, J45, J46 or J47
‡International Classification of Disease 10 codes E10, E11, E12, E13 or E14

Prescriptions Information System (PIS) extract dataset – by practice, year and month

**Variable**	**Details**
Practice Code	The unique NHS Scotland code for the GP Practice
Year	The year to which the data relates (2000-13)
Month	The month to which the data relates
Category	The medication class to which the data relates (ALL prescriptions, Inhaled Corticosteroids or Diabetes)
Paid/Exempt	A flag to indicate whether the data relates to Paid or Exempt prescriptions
Exemption Category*	The prescription exemption category to which the data relates
Number of Items Paid	The number of prescription items paid
Quantity Paid	The total quantity paid
Gross Ingredient Cost Paid	The total cost of the medicines supplied before discount
DDDs Paid	The total number of defined daily doses paid

*For data from April 2011, exemption category will not be available. This should be replaced by the following age-bandings: 0-15, 16-18, 19-59; 60+ years. Ages should be based on the date of prescribing.

For peer review only - http://bmjopen.bmj.com/site/about/guidelines.xhtml

Supplemental file 2 – National Health Service Prescription Fee exemption Criteria [1]

Medical Grounds:

- Forms of hypoadrenalism (including Addison's disease) for which specific substitution therapy is essential
- Diabetes insipidus or other forms of hypopituitarism
- Diabetes mellitus (except where treatment is by diet alone)
- Hypoparathyroidism
- Myasthenia gravis
- Myxodoema, or other conditions which require supplemental thyroid hormone
- Epilepsy requiring continuous anti-convulsive therapy
- A permanent fistula, requiring an appliance or continuous surgical dressing
- Continuing physical disability which prevents the patient from leaving his/her residence without the help of another person
- Cancer (added in 2009)

Pregnant or nursing mothers (these are included with the medical exemptions)
Contraceptives (these are included with the medical exemptions)
Age:

- Under 16 years
- 16-18 years and in full time education
- Over 60 years

Financial situation:

- Those receiving Income Support, Income-Based Job Seeker's Allowance, Income-related Employment and Support Allowance, Pension Credit Guarantee Credit, Universal Credit and people who qualify for full help under the NHS Low Income Scheme are exempt.

References

1 NHS Choices. Get help with prescription costs. London. Gov.uk, 2017. Website: https://www.nhs.uk/nhsengland/Healthcosts/pages/Prescriptioncosts.aspx (accessed 13 December 2017)

For peer review only

Supplemental file 3 Sensitivity analysis

Admissions

Intervention

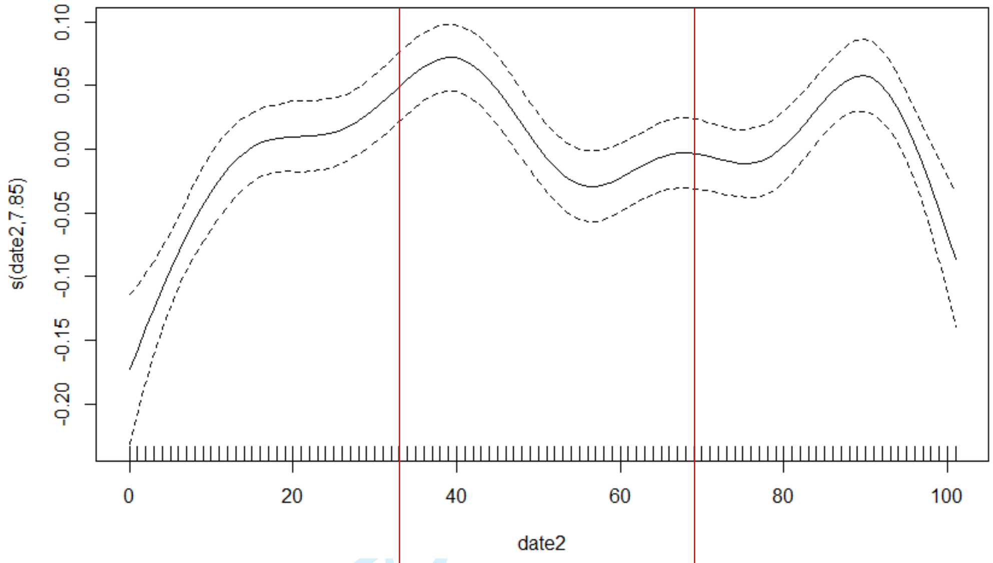

Age counterfactual

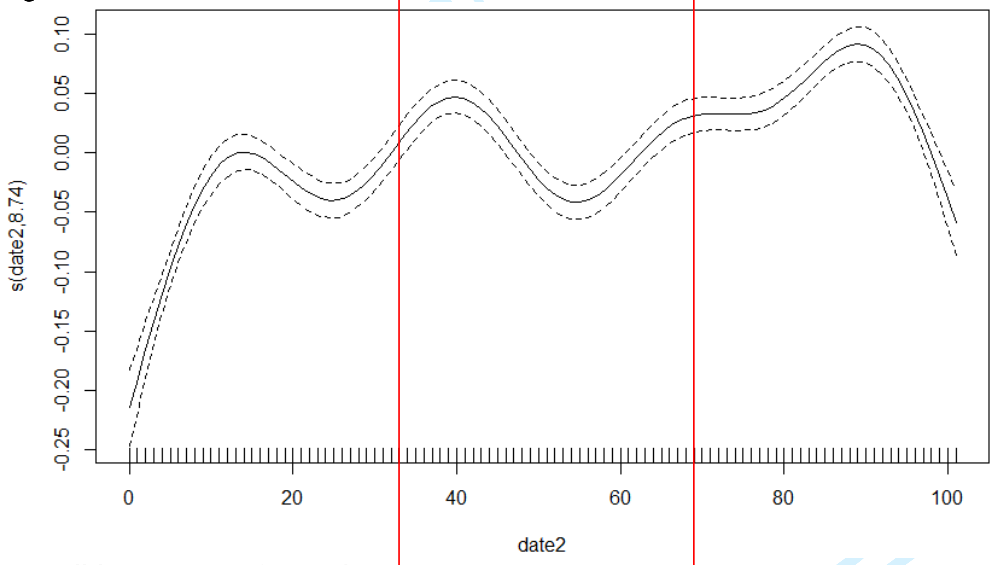

Condition counterfactual

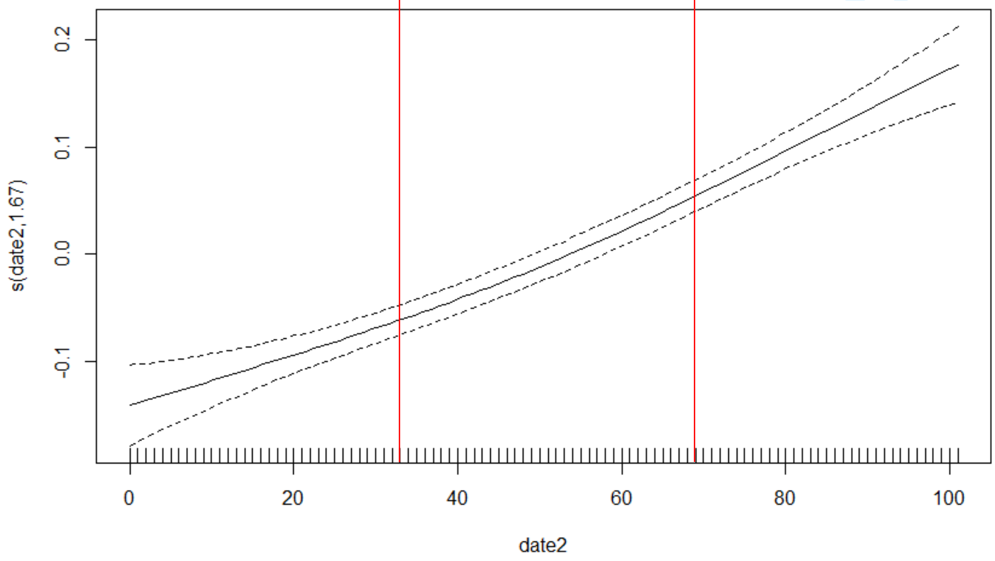

Supplemental file 3 Sensitivity analysis

Defined Daily Doses (DDDs)

Intervention

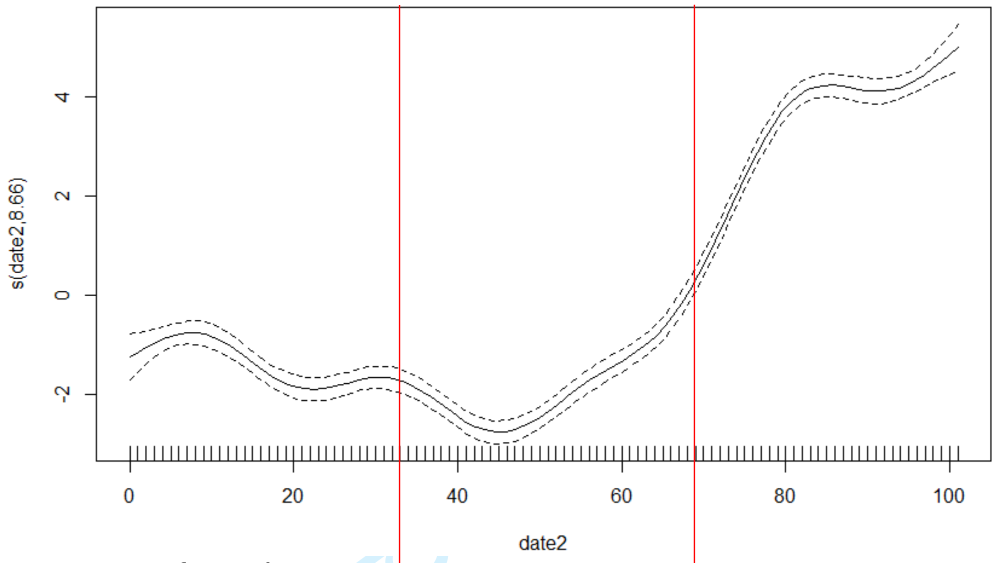

Age counterfactual

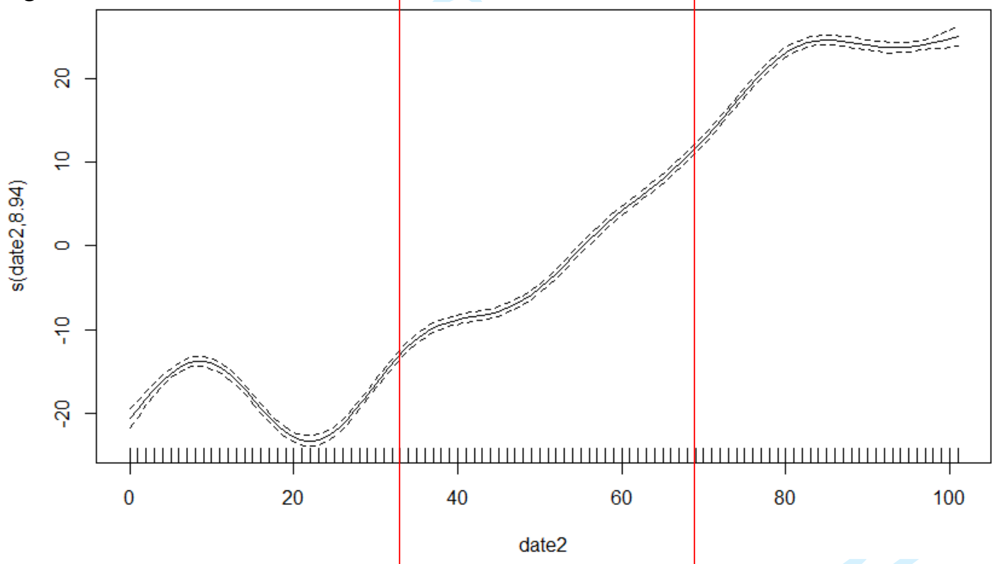

Condition counterfactual

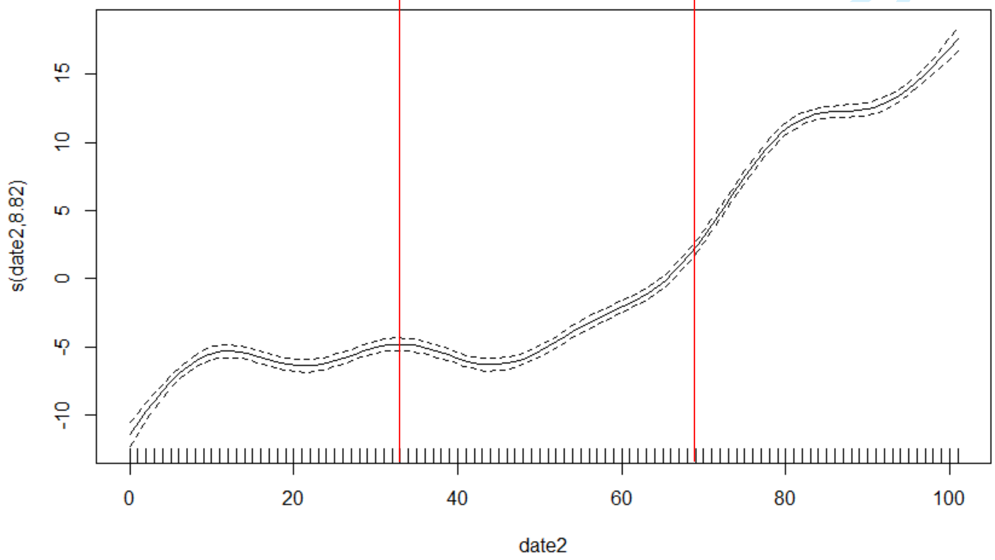

Supplemental file 3 Sensitivity analysis

Cost

Intervention

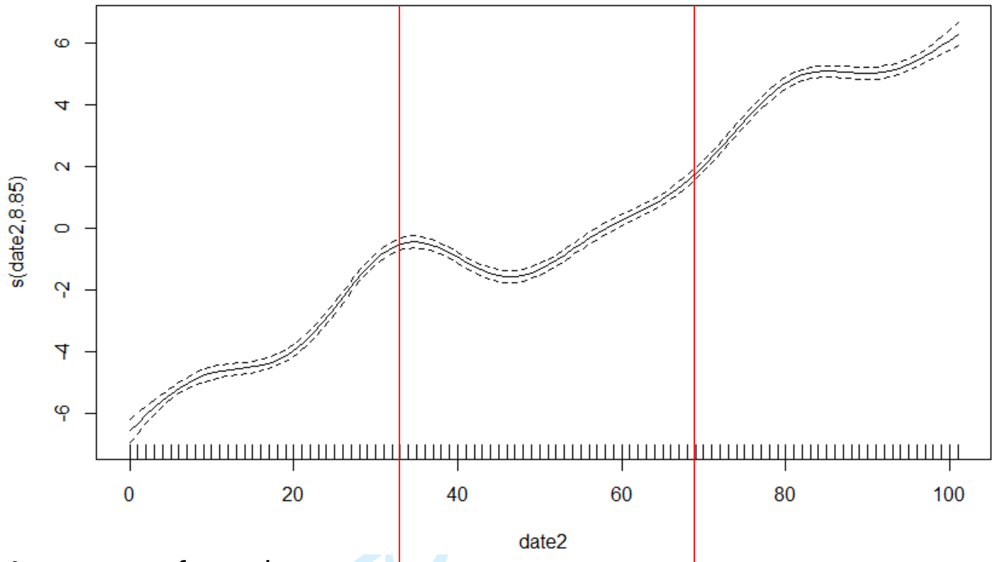

Age counterfactual

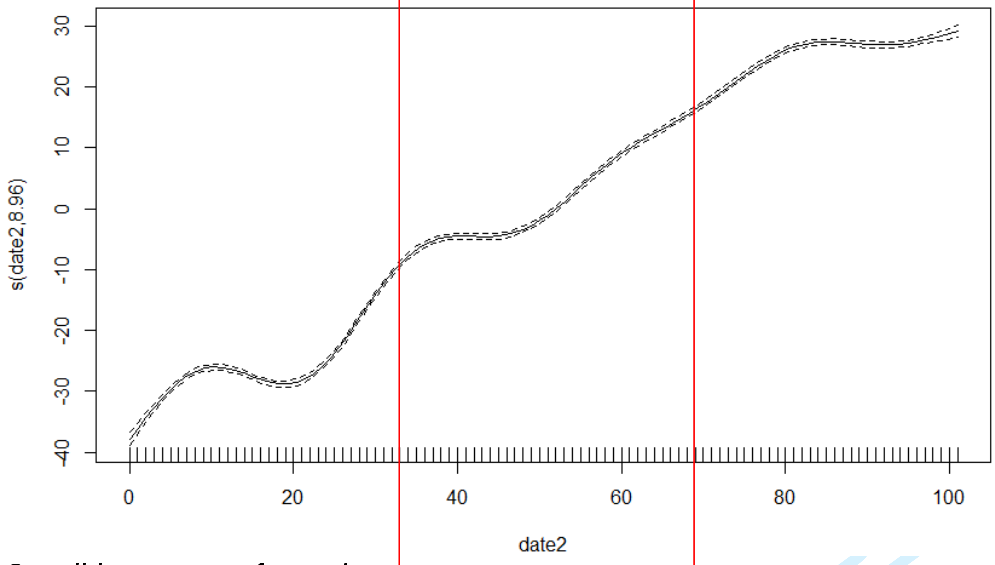

Condition counterfactual

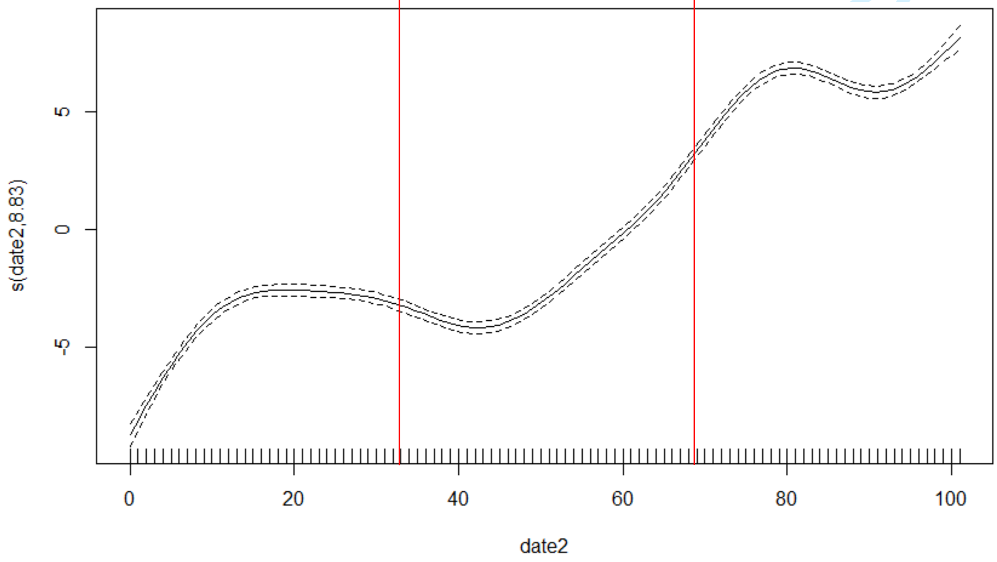

STROBE 2007 (v4) Statement—Checklist of items that should be included in reports of *cohort studies*

Section/Topic	Item #	Recommendation	Reported on page #
**Title and abstract**	1	(*a*) Indicate the study's design with a commonly used term in the title or the abstract	1
		(*b*) Provide in the abstract an informative and balanced summary of what was done and what was found	2
**Introduction**			
Background/rationale	2	Explain the scientific background and rationale for the investigation being reported	4-5
Objectives	3	State specific objectives, including any prespecified hypotheses	5
**Methods**			
Study design	4	Present key elements of study design early in the paper	5-9
Setting	5	Describe the setting, locations, and relevant dates, including periods of recruitment, exposure, follow-up, and data collection	5-6
Participants	6	(*a*) Give the eligibility criteria, and the sources and methods of selection of participants. Describe methods of follow-up	6-7
		(*b*) For matched studies, give matching criteria and number of exposed and unexposed	N/A
Variables	7	Clearly define all outcomes, exposures, predictors, potential confounders, and effect modifiers. Give diagnostic criteria, if applicable	5-9
Data sources/ measurement	8*	For each variable of interest, give sources of data and details of methods of assessment (measurement). Describe comparability of assessment methods if there is more than one group	5-9
Bias	9	Describe any efforts to address potential sources of bias	5-16
Study size	10	Explain how the study size was arrived at	9
Quantitative variables	11	Explain how quantitative variables were handled in the analyses. If applicable, describe which groupings were chosen and why	6-9
Statistical methods	12	(*a*) Describe all statistical methods, including those used to control for confounding	7-9
		(*b*) Describe any methods used to examine subgroups and interactions	7-9
		(*c*) Explain how missing data were addressed	NA
		(*d*) If applicable, explain how loss to follow-up was addressed	NA
		(*e*) Describe any sensitivity analyses	16
**Results**			

For peer review only

Participants	13*	(a) Report numbers of individuals at each stage of study—eg numbers potentially eligible, examined for eligibility, confirmed eligible, included in the study, completing follow-up, and analysed	9-10
		(b) Give reasons for non-participation at each stage	NA
		(c) Consider use of a flow diagram	NA
Descriptive data	14*	(a) Give characteristics of study participants (eg demographic, clinical, social) and information on exposures and potential confounders	Table 2 page 9-10
		(b) Indicate number of participants with missing data for each variable of interest	NA – complete case analysis
		(c) Summarise follow-up time (eg, average and total amount)	5
Outcome data	15*	Report numbers of outcome events or summary measures over time	Tables 3 and 4
Main results	16	(*a*) Give unadjusted estimates and, if applicable, confounder-adjusted estimates and their precision (eg, 95% confidence interval). Make clear which confounders were adjusted for and why they were included	Tables 3 and 4
		(*b*) Report category boundaries when continuous variables were categorized	NA
		(*c*) If relevant, consider translating estimates of relative risk into absolute risk for a meaningful time period	NA
Other analyses	17	Report other analyses done—eg analyses of subgroups and interactions, and sensitivity analyses	13-16
**Discussion**			
Key results	18	Summarise key results with reference to study objectives	16-17
**Limitations**			
Interpretation	20	Give a cautious overall interpretation of results considering objectives, limitations, multiplicity of analyses, results from similar studies, and other relevant evidence	16-18
Generalisability	21	Discuss the generalisability (external validity) of the study results	16-18
**Other information**			
Funding	22	Give the source of funding and the role of the funders for the present study and, if applicable, for the original study on which the present article is based	3

*Give information separately for cases and controls in case-control studies and, if applicable, for exposed and unexposed groups in cohort and cross-sectional studies.

Note: An Explanation and Elaboration article discusses each checklist item and gives methodological background and published examples of transparent reporting. The STROBE checklist is best used in conjunction with this article (freely available on the Web sites of PLoS Medicine at http://www.plosmedicine.org/, Annals of Internal Medicine at http://www.annals.org/, and Epidemiology at http://www.epidem.com/). Information on the STROBE Initiative is available at www.strobe-statement.org.

For peer review only

Interrupted time series evaluation of the impact of abolishing prescription fees in Scotland on hospital admissions and prescribed medicines

Journal:	*BMJ Open*
Manuscript ID	bmjopen-2017-021318.R1
Article Type:	Research
Date Submitted by the Author:	18-Apr-2018
Complete List of Authors:	Williams, Andrew James; University of Exeter, European Centre for Environment and Human Health<br>Henley, W; Institute of Health Research, University of Exeter Medical School, College House, St Luke's Campus, Exeter, Devon, UK, EX1 2LU<br>Frank, John; University of Edinburgh, The Usher Institute of Population Health Sciences and Informatics, College of Medicine and Veterinary Medicine,
**Primary Subject Heading**:	Health policy
Secondary Subject Heading:	General practice / Family practice, Health economics, Health services research, Public health, Pharmacology and therapeutics
Keywords:	administrative data, natural experiment, quasi-experimental, interrupted time series, Fees, Pharmaceutical, co-payment

SCHOLARONE™
Manuscripts

Interrupted time series evaluation of the impact of abolishing prescription fees in Scotland on hospital admissions and prescribed medicines

Andrew James Williams
European Centre for Environment and Human Health, University of Exeter Medical School, Truro, United Kingdom

William Henley
Institute of Health Research, University of Exeter Medical School, Exeter, United Kingdom

John Frank (corresponding author)
Scottish Collaboration for Public Health Research and Policy, University of Edinburgh, 20 West Richmond Street, Edinburgh EH8 9DX United Kingdom
john.frank@ed.ac.uk, +44(0)131 6 511593

Word count (excluding title page, abstract, references, figures and tables): 3,933

For peer review only

For peer review only - http://bmjopen.bmj.com/site/about/guidelines.xhtml

ABSTRACT

Objectives: To identify whether the abolition of prescription fees in Scotland resulted in:

a) increase in the number (cost to NHS) of medicines prescribed for which there had been a fee (inhaled corticosteroids);
b) reduction in hospital admissions for conditions related to those medications for which there had been a fee (asthma or Chronic Obstructive Pulmonary Disease (COPD)) –

when both are compared to prescribed medicines and admissions for a condition (diabetes mellitus) for which prescriptions were historically free.

Design: Natural experimental retrospective General Practice level interrupted time series (ITS) analysis using administrative data

Setting: General Practices, Scotland, United Kingdom

Participants: 732 (73.6%) General Practices across Scotland with valid dispensed medicines and hospital admissions data during the study period (July 2005 – December 2013)

Intervention: Reduction in fees per dispensed item from April 2008 leading to the abolition of the fee in April 2011, resulting in universal free prescriptions

Primary and secondary outcome: Hospital admissions recorded in the Scottish Morbidity Record – 01 Inpatient (SMR01) and dispensed medicines recorded in the Prescriptions Information System (PIS).

Results: The ITS analysis identified marked step reductions in adult (19-59 years) admissions related to asthma or COPD (the intervention group), compared to older or young people with the same conditions or adults with diabetes mellitus (the counterfactual groups). The prescriptions findings were less coherent and a subsequent sensitivity analysis found that both the admissions and prescriptions data were highly variable above the annual or seasonal level, limiting the ability to interpret the findings of the ITS analysis.

Conclusions: This study did not find sufficient evidence that universal free prescriptions was a demonstrably effective or ineffective policy, in terms of reducing hospital admissions or reducing socioeconomic inequality in hospital admissions, in the context of a universal, publicly administered medical care system, the National Health Service of Scotland.

For peer review only - http://bmjopen.bmj.com/site/about/guidelines.xhtml

For peer review only

Key words: Fees, Pharmaceutical, interrupted time series, administrative data, natural experiment, quasi-experimental, co-payment

ARTICLE SUMMARY

Strengths and limitations of this study

- Administrative data permitted the undertaking of a large long-term evaluation of a policy, which otherwise would have been very costly and indeed, perhaps infeasible.
- Data were available for over 70% of the practices in Scotland, which could be used to classify those who did and did not experience a change in prescription fees.
- The data were only available at the General Practice not the individual level.
- The time series for both admissions and prescriptions were highly variable, preventing the drawing of specific conclusions from the evaluation.

STUDY PROTOCOL

Available as Supplemental file 1 online.

FUNDING STATEMENT

This work was funded by the Farr Institute @ Scotland, which is funded by the following consortium: Arthritis Research UK, the British Heart Foundation, Cancer Research UK, the Economic and Social Research Council, the Engineering and Physical Sciences Research Council, the Medical Research Council, the National Institute of Health Research, the National Institute for Social Care and Health Research (Welsh Assembly Government), the Chief Scientist Office (Scottish Government Health Directorates), (MRC Grant No: MR/K007017/1). JF and AJW worked for the Scottish Collaboration for Public Health Research and Policy which is funded by the SCPHRP core grant from the Medical Research Council (Grant Number MR/K023209/1) and the Chief Scientist Office of Scotland. WH received support from the National Institute for Health Research (NIHR) Collaboration for Leadership in Applied Health Research and Care (CLAHRC) for the South West Peninsula. The funders played no role in the conceptualisation or realisation of the research and no role in the decision to submit it for publication.

For peer review only - http://bmjopen.bmj.com/site/about/guidelines.xhtml

COMPETING INTERESTS

The authors declare that they have no competing interests.

For peer review only

For peer review only - http://bmjopen.bmj.com/site/about/guidelines.xhtml

INTRODUCTION

When the National Health Service was founded in the United Kingdom in 1948, all the services provided were free. However, as early as 1950 expenditure was higher than expected, so a charge (1 shilling) for prescribed items (including medicines, medical devices, dressing, borderline substances etc.) was introduced in 1952.[1] This prescription fee has increased over time and now in England the charge is £8.80 per item.[2] In 1968 a list of medical conditions for which no prescription fee would be charged was introduced; these were easily recognisable, lifelong, life threatening conditions that required regular, prescribed medication.[1] Over time further exemptions were identified and the most recent list of exemption criteria is very extensive indeed (Supplemental file 2) [2]. Now, the majority of prescriptions are exempt, meaning that in 2004/05 less than 5% of the cost of pharmaceutical services in Scotland was covered by the prescription fee.[1]

The prescription fee has been described as a ‘tax on ill health’ (Nicola Sturgeon, then Scottish Health Secretary).[3,4] Although, the exemption criteria (Supplemental file 2) contain many chronic diseases, important conditions such as asthma and degenerative neurological conditions are missing. These omissions have led many to call for the abolition of the fee.[1,5,6] In 2007 Wales was the first country in the United Kingdom (UK) to abolish prescription fees, followed by Northern Ireland in 2010 and Scotland in 2011; a fee is still charged in England.[3] Figure 1(a) shows prescription fees in England and Scotland during the time period examined in this study. Patients requiring multiple prescriptions a month could purchase a short-term (3-4 month) or long-term (12 month) Pre-Payment Certificate (PPC) which provided a discount. Alongside the prescription fee reduction, Scotland made a marked reduction in PPC cost (Figure 1b).

Although, the prescription fee only covers a small proportion of the costs of prescription services, abolishing the fee means that the cost needs to be met elsewhere. One of the justifications for abolishing prescription fees has been that this will lead to improved adherence to medication and subsequently reduced medical costs, through less need for expensive secondary medical care. Campbell, et al.[7] found that for asthmatic patients in the United States of America (USA), an increase as small as $5 in pharmacy co-payments resulted in reduced adherence and increased outpatient visits. Kulik, et al.[8] undertook a randomised comparison in the USA of the health impact of providing patients with recent myocardial infarction with

For peer review only - http://bmjopen.bmj.com/site/about/guidelines.xhtml

free prescriptions for all medications, versus only a small selection of cardiovascular medications. It was found that full prescription cost coverage resulted in significantly improved adherence and trends towards reductions in rates of major vascular events or revascularisation [8]. In Canada, where all prescriptions are free for those over 65 years of age, health inequalities by socioeconomic position among those with diabetes are much reduced in this age group compared with those under 65 [9]. Thus far, the only published evaluations of the policy change across the UK have come from Wales and only included prescriptions data and anecdotal reports around other outcomes.[10-13] Hence, the intention of this study was to identify whether the abolition of prescription fees in Scotland resulted in:

a) increase in the number (cost to NHS) of medicines prescribed for which there had been a fee (inhaled corticosteroids);
b) reduction in hospital admissions for conditions related to those medications for which there had been a fee (asthma and Chronic Obstructive Pulmonary Disease (COPD)).

The comparisons for both prescriptions and admissions were conditions for which prescriptions were historically free (diabetes mellitus, or the young or elderly). Subsequent research questions were:

c) Was there any change in hospital admissions in the practices which also saw a change in dispensing?
d) How did the effects of fee abolition differ across the socioeconomic spectrum?

METHODS

Retrospective policy analysis As the abolition of prescription fees had already taken place and was not implemented experimentally, it was necessary to use a natural experimental approach: interrupted time series. This required that historical data on policy exposure and outcomes were available.

Data Two large administrative datasets are available in Scotland which made this study feasible; the Prescribing Information System (PIS) and the Scottish Morbidity Record – 01 Inpatient (SMR01). PIS is the database of all community prescribed and dispensed medicines across Scotland. SMR01 is the database of all hospital inpatient admissions across Scotland. Using the unique identifier, the Community Health Index (CHI), it is possible to link individual health records across health databases in Scotland. However, CHI has only been recorded in the PIS since

For peer review only - http://bmjopen.bmj.com/site/about/guidelines.xhtml

August 2009, and therefore this study was restricted to General Practice-level data. The time period examined in this study was July 2005 – December 2013 with fee reductions beginning in April 2008 and abolished from April 2011.

Exposure During study development, a group of clinicians and pharmacists were consulted to inform the study design, in particular the definition of exposure to the policy. Starting from the prescription fee exemption criteria (Supplemental file 2), groups for whom prescriptions remained free (counterfactual) and became free (intervention) needed defining. The groups needed to be identifiable in the PIS and SMR01 and relate to relatively common conditions for which non-adherence to medication would likely result in the need for hospital admission. Subsequently, the subset of pharmaceutical-sensitive ambulatory care-sensitive conditions was used.[14] No data on patient financial situation is recorded in SMR01, which meant that intervention and counterfactual groups could not be defined in terms of the finance related prescription fee exemption criteria. Research question (d) was included so that some examination of the impact of financial situation was undertaken. Asthma and COPD, common adulthood conditions with condition-specific mediations for which the prescription fee was charged, were identified as the most appropriate intervention conditions. Diabetes mellitus, medication for which had been exempt from the prescription fee for many years, constituting the counterfactual condition. In addition to a condition-based counterfactual group, an age-based counterfactual group was used. The criteria for the intervention and two counterfactual groups is given in Table 1. The need to use the exemption criteria to classify intervention and counterfactuals meant that dispensing data from PIS was used, as exemption is only recorded when the prescription is dispensed. This prevented the study's consideration of primary non-adherence, when the patient does not have the prescription dispensed.

For peer review only - http://bmjopen.bmj.com/site/about/guidelines.xhtml

Table 1 – Intervention and counterfactual group definitions

	Prescribing Information System (PIS)	Scottish Morbidity Record – 01 Inpatient (SMR01)
**Intervention**	BNF: 3.2 Inhaled steroids<br>Fee: paid<br>Age group: >18 & <60 years	ICD-10: J20, J40X, J41, J42, J43, J44, J45, J46 or J47<br>Age group: >18 & <60 years
**Condition-based counterfactual**	BNF: 6.1.1 or 6.1.2<br>Fee: exempt E - Medical exemption<br>Age group: >18 & <60 years	ICD-10: E10, E11, E12, E13 or E14<br>Age group: >18 & <60 years
**Age-based counterfactual**	BNF: 3.2 Inhaled steroids<br>Fee: exempt A - <16 years, B - ≥16 - ≤18 years and in full time education or C - ≥60 years<br>Age group: <16 years, ≥16 - ≤18 years or ≥60 years	ICD-10: J20, J40X, J41, J42, J43, J44, J45, J46 or J47<br>Age group: <16 years, ≥16 - ≤18 years or ≥60 years

BNF: British National Formulary, ICD-10: International Classification of Disease 10th version

Using the exemption criteria to define the intervention and counterfactual groups within the PIS presented a challenge, as the exemption criteria did not need to be recorded after the fee abolition. Fortuitously, exemption criteria continued to be recorded in many instances. The proportion of prescriptions against each exemption criteria was compared before and after the abolition, to check if reporting had changed. Apart from those receiving free prescriptions due to being aged 60 years or over, reporting of exemption criteria fell markedly, particularly reporting PPCs or full payment. Consequently, given that Scottish Public Health Observatory data [15-17] did not show any marked changes in the trends prevalence of asthma, COPD or diabetes during the study period, the proportion eligible for each exemption prior to the policy change was applied to the PIS data after the policy change. This was not an issue for the SMR-01 data, as the variables used to define the groups (Table 1) were not affected by the policy change. The datasets were prepared, cleaned and merged in Stata [18] with the top 1% of prescription data being deleted as these very large values appeared to be errors (most likely due to keying errors during manual data entry). To make the outcomes comparable between groups, the primary metric of prescriptions used was Defined Daily Doses (DDDs). The prescription cost was also examined in terms of the gross ingredient cost before any discounts (dispensing costs are met by an overall service remuneration model within the NHS in Scotland rather than an additional cost per item). Number and quantity of prescriptions were provided in the data set but were found not to be comparable across study groups,

For peer review only - http://bmjopen.bmj.com/site/about/guidelines.xhtml

so were not analysed. Data on the following characteristics of each General Practice was also collected: Health Board, number of General Practitioners, list size (by age group), whether the practice had its own dispensary, the practice NHS contract type, the quintile of the Scottish Index of Multiple Deprivation (SIMD) [19] of the practice postcode, the urban rural classification of the practice postcode, the number of patients registered at the practice living in a postcode defined as the 15% most deprived in Scotland, and the number of registered patients from each SIMD quintile and urban rural classification.

Interrupted time series analysis The dataset was declared a time series dataset with monthly time periods and any missing time points within practices were filled with zero, assuming no relevant prescriptions or admissions occurred from that practice that month. Admissions included small counts (e.g. number of adult (19-59 years) asthma admissions per practice per month) so these data were analysed using mixed effects Poisson models, offset by practice size to account for variations in practice size. DDDs and costs were transformed into DDDs or cost per 100 patients per practice to account for differences in practice size. The resulting measures were sufficiently normally distributed to be analysed using mixed-effects linear models. Interrupted time series operators were derived so that any model would fit a new intercept and slope at the time of the intervention, which could be delayed using lags. Calendar month was fitted as a categorical variable to account for seasonality. Finally, the data were treated as hierarchical with measurement month (i) nested within General Practice (j) and the linear model for costs and DDDs was given by:

$$\begin{aligned} y_{ij} = \beta_{0ij} &+ \beta_1 date_{ij} + \beta_2 jan_{ij} + \beta_3 feb_{ij} + \beta_4 mar_{ij} + \beta_5 may_{ij} + \beta_6 jun_{ij} + \beta_7 jul_{ij} \\ &+ \beta_8 aug_{ij} + \beta_9 sep_{ij} + \beta_{10} oct_{ij} + \beta_{11} nov_{ij} + \beta_{12} dec_{ij} \\ &+ \beta_{13} reduction\ period_{ij} + \beta_{14} time\ since\ reductions\ began_{ij} \\ &+ \beta_{15} abolition\ period_{ij} + \beta_{16} time\ since\ abolition\ began_{ij} + e_j + \mu_{ij} \end{aligned}$$

Where y_{ij} is the outcome for practice *j* at time *i* (in months), $date_{ij}$ is the count of months from the beginning of the study period (July 2005), e_j is the difference between the intercept for the estimated overall trend and the intercept for the estimated trend for practice *j*, and μ_{ij} is the difference between the observed outcome value for time *i* at practice *j* and the predicted value, and e_j and μ_{ij} are random

For peer review only - http://bmjopen.bmj.com/site/about/guidelines.xhtml

effects. Jan_{ij} to dec_{ij} are dummy variables for each month from January to December, with April being the reference category as this was the month in which the policy changes were implemented. $Reduction_{ij}$ *period* and $abolition_{ij}$ *period* are dummy variables for the periods April 2008 to March 2011, and April 2011 to December 2013 (the end of the study period) respectively. *Time since reductions* $began_{ij}$ and *time since abolition* $began_{ij}$ are counts of months from the beginning to end of the reduction and abolition periods respectively. The meaning of the beta coefficients were: β_0 mean outcome in July 2005 (intercept), β_1 secular trend for time from July 2005, $\beta_{2\text{-}12}$ mean effect for each month excluding April (seasonality), β_{13} change in intercept when the prescription fee was being reduced (reduction step change), β_{14} change in slope (from April 2008 to March 2011) when the prescription fee was being reduced (reduction change in slope), β_{15} change in intercept when the prescription fee was abolished (abolition step change) and β_{16} change in slope (from April 2011) when the prescription fee was abolished (abolition change in slope). The Poisson model for admissions was defined similarly with the logarithm of the admission rate expressed as a linear function of the fixed and random effects, and with the addition of an offset term for the practice size.

Admissions data were analysed with 0, 1, 2 and 3 month lags on the intervention effect. To address research question (c), it was planned that practices where changes in prescriptions and admissions consistent with the hypothesised effect of the policy change be identified and compared as a secondary analysis. Finally, to address research question (d), practices whose population modal quintile of the SIMD 2012 [19] remained the same throughout the study period (constant sample size) were included in a repetition of the primary analysis stratified by quintile of SIMD. During model development the hierarchical linear time series models were specified with first or second order autoregression or moving averages. The results did not differ substantially between specifications and therefore all the results presented were specified with second order moving average.

RESULTS

Across the eight-year study period, the number of practices and their characteristics changed. The characteristics of the practices with prescriptions data at the end of the study period (December 2013) are presented in Table 2. Data on prescribed medicines were available for 732 (73.6%) of the 994 General Practices across

For peer review only - http://bmjopen.bmj.com/site/about/guidelines.xhtml

Scotland at the end of 2013, while admissions data was available for 754 (75.9%) practices. The majority of practices had 5 or fewer general practitioners, fewer than 10,000 patients, and were in urban areas. Less than 10% of practices had their own dispensary.

Table 2 – Practice characteristics in December 2013 (n=732)

Characteristic	Summary statistic
Number of GPs	
≤5 general practitioners	447 (61.1%)
>5–≤10 general practitioners	246 (33.6%)
>10 general practitioners	39 (5.3%)
Practice size	
≤5,000 patients	345 (47.1%)
>5,000–≤10,000 patients	305 (41.7%)
>10,000 patients	82 (11.2%)
Dispensing practice	67 (9.2%)
Health Board	
NHS Ayrshire & Arran	37 (5.1%)
NHS Borders	20 (2.7%)
NHS Dumfries & Galloway	26 (3.6%)
NHS Fife	40 (5.5%)
NHS Forth Valley	44 (6.0%)
NHS Grampian	37 (5.1%)
NHS Greater Glasgow & Clyde	215 (29.4%)
NHS Highland	72 (9.8%)
NHS Lanarkshire	60 (8.2%)
NHS Lothian	107 (14.6%)
NHS Orkney	3 (0.4%)
NHS Shetland	4 (0.5%)
NHS Tayside	60 (8.2%)
NHS Western Isles	7 (1.0%)
Modal SIMD quintile of patients	
Quintile 1 (most deprived)	224 (30.6%)
Quintile 2	127 (17.3%)
Quintile 3	155 (21.2%)
Quintile 4	108 (14.8%)
Quintile 5 (least deprived)	118 (16.1%)
Modal Location of patients	
Large Urban Areas	302 (41.3%)
Other Urban Areas	192 (26.2%)
Accessible Small Towns	64 (8.7%)
Remote Small Towns	17 (2.3%)
Very Remote Small Towns	9 (1.2%)
Accessible Rural	61 (8.3%)
Remote Rural	32 (4.4%)
Very Remote Rural	55 (7.5%)

GP; general practitioner, NHS; National Health Service, SIMD; Scottish Index of Multiple Deprivation [19]

For peer review only

For peer review only - http://bmjopen.bmj.com/site/about/guidelines.xhtml

The intercept (constant) and coefficients of the interrupted time series operators for the Poisson and linear mixed effects models of admissions, DDDs and cost are presented in Table 3. Throughout the results, p-values have not been reported and the focus was on estimated effect sizes, as the size of the dataset resulted in many statistical but not meaningful significances. The intercept estimates demonstrate that there were pre-existing differences in the level of admissions and prescriptions across the intervention and counterfactual groups at the start of the study period (July 2005). The highest rate of admissions, and number (DDDs) and cost of prescribed medicines were observed in the age-counterfactual group, while the fewest admissions and prescribed medicines were seen among the intervention group. This demonstrates that asthma and COPD are, as expected, most problematic to the young and old. Despite these differences in quantity, the background time trend in admissions before the abolition of prescription fees is consistent across the groups. The coefficients of the interrupted time series operators are also mostly consistent across groups and increasing degrees of time lag. A notable exception was a significant step reduction in admissions in the intervention group, related to the abolition of prescription fees, although the underlying time trend did not significantly change. The results regarding prescribed medicines are much less clear, with more marked temporal variability across the study period. However, the largest changes appear in the counterfactual groups, indicating some phenomenon other than the intended effect of the policy change, which we were unable to identify.

For peer review only - http://bmjopen.bmj.com/site/about/guidelines.xhtml

Table 3 – Results of interrupted time series analysis of the impact of reducing and abolishing prescription fees in Scotland on hospital admissions (incidence rate ratios) and prescribed medicines (defined daily doses and cost)

	**Intercept**		**Monthly change**		**Reduction step change**		**Reduction change in slope**		**Abolition step change**		**Abolition change in slope**	
**Admissions**	IR	95% CI	IRR	95% CI	IRR	95% CI	IRR	95% CI	IRR	95% CI	IRR	95% CI
Intervention	$1.01e^{-2}$	$9.46e^{-3}$ to $1.09e^{-2}$	1.00	1.00 to 1.01	1.03	0.98 to 1.08	0.99	0.99 to 0.99	0.81	0.72 to 0.90	1.00	0.99 to 1.00
Age-c'fact	$6.65e^{-2}$	$6.36e^{-2}$ to $6.95e^{-2}$	1.00	1.00 to 1.00	1.03	1.01 to 1.06	1.00	0.99 to 1.00	0.95	0.90 to 1.00	1.00	0.99 to 1.00
Condition-c'fact	$4.80e^{-3}$	$4.39e^{-3}$ to $5.24e^{-3}$	1.00	1.00 to 1.01	0.95	0.88 to 1.03	1.00	1.00 to 1.01	1.01	0.86 to 1.20	1.00	1.00 to 1.01
**Admissions 1 month lag**	IR	95% CI	IRR	95% CI	IRR	95% CI	IRR	95% CI	IRR	95% CI	IRR	95% CI
Intervention	$1.02e^{-2}$	$9.49e^{-3}$ to $1.09e^{-2}$	1.01	1.00 to 1.01	1.01	0.96 to 1.06	0.99	0.99 to 0.99	0.78	0.71 to 0.87	1.00	0.99 to 1.00
Age-c'fact	$6.74e^{-2}$	$6.45e^{-2}$ to $7.05e^{-2}$	1.00	1.00 to 1.00	1.03	1.01 to 1.06	1.00	0.99 to 1.00	0.95	0.90 to 1.00	1.00	0.99 to 1.00
Condition-c'fact	$4.83e^{-3}$	$4.41e^{-3}$ to $5.28e^{-3}$	1.00	1.00 to 1.00	0.98	0.91 to 1.06	1.00	1.00 to 1.00	1.07	0.91 to 1.26	1.00	1.00 to 1.01
**Admissions 2 month lag**	IR	95% CI	IRR	95% CI	IRR	95% CI	IRR	95% CI	IRR	95% CI	IRR	95% CI
Intervention	$1.02e^{-2}$	$9.51e^{-3}$ to $1.09e^{-2}$	1.00	1.00 to 1.01	1.01	0.96 to 1.06	0.99	0.99 to 0.99	0.79	0.71 to 0.87	1.00	0.99 to 1.00
Age-c'fact	$6.77e^{-2}$	$6.47e^{-2}$ to $7.07e^{-2}$	1.00	1.00 to 1.00	1.04	1.02 to 1.07	1.00	0.99 to 1.00	0.97	0.92 to 1.02	1.00	1.00 to 1.00
Condition-c'fact	$4.85e^{-3}$	$4.44e^{-3}$ to $5.30e^{-3}$	1.00	1.00 to 1.00	0.99	0.92 to 1.07	1.00	1.00 to 1.01	1.10	0.93 to 1.28	1.00	1.00 to 1.01
**Admissions 3 month lag**	IR	95% CI	IRR	95% CI	IRR	95% CI	IRR	95% CI	IRR	95% CI	IRR	95% CI
Intervention	$1.02e^{-2}$	$9.49e^{-2}$ to $1.09e^{-2}$	1.01	1.00 to 1.01	0.99	0.94 to 1.04	0.99	0.99 to 0.99	0.78	0.70 to 0.86	1.00	0.99 to 1.00
Age-c'fact	$6.73e^{-2}$	$6.44e^{-2}$ to $7.04e^{-2}$	1.00	1.00 to 1.00	1.02	1.00 to 1.05	1.00	0.99 to 1.00	0.94	0.90 to 0.99	1.00	1.00 to 1.00
Condition-c'fat	$4.82e^{-3}$	$4.41e^{-3}$ to $5.26e^{-3}$	1.00	1.00 to 1.00	0.97	0.90 to 1.05	1.00	1.00 to 1.01	1.08	0.92 to 1.26	1.00	1.00 to 1.00
**Defined Daily Doses (DDDs)**	DDDs	95% CI	ΔDDDs	95% CI	ΔDDDs	95% CI	ΔDDDs	95% CI	ΔDDDs	95% CI	ΔDDDs	95% CI
Intervention	29.65	28.71 to 30.59	-0.04	-0.06 to -0.02	-0.11	-0.61 to 0.39	0.04	0.02 to 0.07	6.03	4.94 to 7.13	0.10	0.08 to 0.13
Age-c'fact	100.88	98.07 to 103.70	-0.05	-0.11 to 0.01	5.06	3.61 to 6.52	0.68	0.59 to 0.77	38.37	34.84 to 41.90	0.34	0.25 to 0.42
Condition-c'fact	55.61	53.70 to 57.53	0.14	0.10 to 0.18	-1.88	-2.90 to -0.85	-0.05	-0.11 to 0.00	6.02	3.73 to 8.32	0.15	0.09 to 0.20
**Cost**	£s	95% CI	Δ£s	95% CI	Δ£s	95% CI	Δ£s	95% CI	Δ£s	95% CI	Δ£s	95% CI
Intervention	19.36	18.53 to 20.19	0.16	0.15 to 0.18	0.23	-0.19 to 0.66	-0.15	-0.17 to -0.13	-0.96	-1.92 to -0.01	-0.10	-0.12 to -0.08
Age-c'fact	69.92	67.35 to 72.49	0.62	0.56 to 0.68	4.74	3.42 to 6.06	0.03	-0.05 to 0.12	14.24	10.86 to 17.63	-0.36	-0.44 to -0.28
Condition-c'fact	26.35	25.35 to 27.34	0.15	0.13 to 0.17	-3.16	-3.70 to -2.62	-0.01	-0.04 to 0.02	2.00	0.79 to 3.20	-0.11	-0.14 to -0.08

95% CI; 95% confidence interval, c'fact; counterfactual, DDDs; Defined daily doses per 100 patients per practice per month, ΔDDDs; change in DDDs, IR; Incidence rate per 100 patients per practice per month, IRR; incidence rate ratio, £s; gross ingredient cost of medicines before any discount per 100 patients per practice per month, Δ£s; change in £s

For peer review only - http://bmjopen.bmj.com/site/about/guidelines.xhtml

For peer review only

The results of the analysis undertaken to examine whether the impact of the policy differed by socioeconomic position are presented in Table 4. These analyses aimed to assess whether the financial exemptions to the prescription fee (Supplemental file 2) had an impact on the results, as these exemptions could not be accounted for directly in the main analysis. Only the results for the intervention group have been presented, as individual financial position should not have had an impact on the counterfactual groups. The intercepts of the admissions analyses are consistent with known inequalities, with the highest rate of admissions among the most deprived (Q1), and the lowest rate of admissions in the least deprived (Q5). The results for the interrupted time series operators are similar to the main analysis, with the most marked differences being observed in terms of step changes as opposed to changes in the underlying time trend. Although not statistically significant, there seems to be some evidence of reductions in admissions among the most deprived quintiles (Q1 and Q2) in relation to the fee-reduction policy, compared to the least deprived quintiles (Q4 and Q5). However, any effect is not linear across the SIMD quintiles. Again, the results of the analysis of prescriptions were less coherent. The intercepts of the models demonstrate that most prescriptions were from practices with mostly Q3 and Q4 patients. This makes sense as those in the lower quintiles are more likely to have a financial exemption. These quintiles also demonstrate the largest step increases in DDDs when the fees were abolished, which is consistent with this being an effect of the policy.

For peer review only

Table 4 - Results of interrupted time series analysis of the impact of reducing and abolishing prescription fees in Scotland on hospital admissions (incidence rate ratios) and prescribed medicines (defined daily doses and cost) stratified by quintile of the Scottish Index of Multiple Deprivation [19]

	Intercept		Monthly change		Reduction step change		Reduction change in slope		Abolition step change		Abolition change in slope	
**Admissions**	IR	95% CI	IRR	95% CI	IRR	95% CI	IRR	95% CI	IRR	95% CI	IRR	95% CI
Q1 - most deprived	$1.52e^{-2}$	$1.35e^{-2}$ to $1.71e^{-2}$	1.01	1.01 to 1.01	1.00	0.91 to 1.09	0.99	0.98 to 0.99	0.64	0.52 to 0.78	0.99	0.98 to 0.99
Q2	$1.17e^{-2}$	$9.36e^{-3}$ to $1.47e^{-2}$	1.00	1.00 to 1.01	1.10	0.92 to 1.32	0.99	0.98 to 1.00	0.75	0.50 to 1.12	1.00	0.99 to 1.01
Q3	$9.99e^{-3}$	$7.45e^{-3}$ to $1.34e^{-2}$	1.00	0.99 to 1.01	1.10	0.86 to 1.41	1.00	0.98 to 1.01	1.15	0.68 to 1.94	1.00	0.99 to 1.02
Q4	$6.49e^{-3}$	$4.64e^{-3}$ to $9.07e^{-3}$	0.99	0.98 to 1.00	1.50	1.13 to 1.99	1.00	0.99 to 1.02	1.63	0.89 to 2.99	1.02	1.01 to 1.04
Q5 - least deprived	$6.06e^{-3}$	$4.86e^{-3}$ to $7.54e^{-3}$	1.00	0.99 to 1.00	1.00	0.84 to 1.21	1.00	1.00 to 1.01	1.24	0.85 to 1.82	1.00	0.99 to 1.01
**Admissions 1 month lag**	IR	95% CI	IRR	95% CI	IRR	95% CI	IRR	95% CI	IRR	95% CI	IRR	95% CI
Q1 - most deprived	$1.50e^{-2}$	$1.33e^{-2}$ to $1.69e^{-2}$	1.01	1.01 to 1.02	0.94	0.86 to 1.03	0.99	0.98 to 0.99	0.60	0.49 to 0.73	0.99	0.98 to 0.99
Q2	$1.20e^{-2}$	$9.58e^{-3}$ to $1.51e^{-2}$	1.00	1.00 to 1.01	1.12	0.93 to 1.34	0.99	0.98 to 1.00	0.79	0.53 to 1.16	1.00	0.99 to 1.01
Q3	$9.97e^{-3}$	$7.40e^{-3}$ to $1.34e^{-2}$	1.00	0.99 to 1.01	1.00	0.78 to 1.27	1.00	0.99 to 1.01	0.99	0.60 to 1.65	1.00	0.99 to 1.02
Q4	$6.81e^{-3}$	$4.85e^{-3}$ to $9.56e^{-3}$	0.99	0.98 to 1.00	1.53	1.16 to 0.02	1.00	0.99 to 1.01	1.66	0.91 to 3.00	1.02	1.01 to 1.04
Q5 - least deprived	$6.12e^{-3}$	$4.90e^{-3}$ to $7.64e^{-3}$	1.00	0.99 to 1.00	1.03	0.86 to 1.23	1.01	1.00 to 1.01	1.27	0.87 to 1.85	1.00	0.99 to 1.01
**Admissions 2 month lag**	IR	95% CI	IRR	95% CI	IRR	95% CI	IRR	95% CI	IRR	95% CI	IRR	95% CI
Q1 - most deprived	$1.51e^{-2}$	$1.34e^{-2}$ to $1.70e^{-2}$	1.01	1.01 to 1.01	0.94	0.86 to 1.03	0.99	0.98 to 0.99	0.60	0.50 to 0.73	0.99	0.98 to 0.99
Q2	$1.20e^{-2}$	$9.58e^{-3}$ to $1.51e^{-2}$	1.00	1.00 to 1.01	1.11	0.92 to 1.33	0.99	0.98 to 1.00	0.78	0.54 to 1.15	1.00	0.99 to 1.01
Q3	$1.02e^{-2}$	$7.59e^{-3}$ to $1.37e^{-2}$	1.00	0.99 to 1.01	1.07	0.84 to 1.37	1.00	0.99 to 1.01	1.12	0.68 to 1.84	1.00	0.99 to 1.02
Q4	$6.77e^{-3}$	$4.83e^{-3}$ to $9.48e^{-3}$	0.99	0.98 to 1.00	1.57	1.19 to 2.07	1.00	0.98 to 1.01	1.70	0.95 to 3.05	1.02	1.00 to 1.03
Q5 - least deprived	$6.21e^{-3}$	$4.98e^{-3}$ to $7.75e^{-3}$	0.99	0.99 to 1.00	1.09	0.91 to 1.31	1.00	1.00 to 1.01	1.38	0.95 to 1.99	1.00	0.99 to 1.01
**Admissions 3 month lag**	IR	95% CI	IRR	95% CI	IRR	95%CI	IRR	95% CI	IRR	95% CI	IRR	95% CI
Q1 - most deprived	$1.52e^{-2}$	$1.35e^{-3}$ to $1.71e^{-2}$	1.01	1.01 to 1.01	0.94	0.86 to 1.02	0.99	0.98 to 0.99	0.60	0.50 to 0.73	0.99	0.98 to 0.99
Q2	$1.18e^{-2}$	$9.40e^{-3}$ to $1.48e^{-2}$	1.01	1.00 to 1.01	1.03	0.86 to 1.24	0.99	0.98 to 1.00	0.72	0.50 to 1.05	1.00	0.99 to 1.01
Q3	$1.05e^{-2}$	$7.82e^{-3}$ to $1.41e^{-2}$	1.00	0.99 to 1.00	1.18	0.92 to 1.51	1.00	0.99 to 1.01	1.25	0.76 to 2.05	1.01	0.99 to 1.02
Q4	$6.40e^{-3}$	$4.58e^{-3}$ to $8.96e^{-3}$	0.99	0.98 to 1.00	1.34	1.02 to 1.77	1.00	0.98 to 1.01	1.36	0.77 to 2.39	1.01	1.00 to 1.03
Q5 - least deprived	$6.15e^{-3}$	$4.93e^{-3}$ to $7.67e^{-3}$	1.00	0.99 to 1.00	1.07	0.89 to 1.28	1.00	0.99 to 1.01	1.35	0.94 to 1.94	1.00	0.99 to 1.01
**Defined Daily Doses (DDDs)**	DDDs	95% CI	ΔDDDs	95% CI	ΔDDDs	95% CI	ΔDDDs	95% CI	ΔDDDs	95% CI	ΔDDDs	95% CI
Q1 - most deprived	21.56	20.13 to 22.99	-0.07	-0.10 to -0.04	0.25	-0.50 to 1.01	0.08	0.04 to 0.12	6.23	4.59 to 7.86	0.14	0.10 to 0.18
Q2	29.47	25.42 to 33.51	0.05	-0.03 to 0.12	-1.83	-3.80 to 0.15	-0.05	-0.15 to 0.05	2.09	-2.19 to 6.37	0.03	-0.07 to 0.14
Q3	35.64	31.29 to 39.98	-0.06	-0.18 to 0.06	1.11	-2.07 to 4.28	-0.03	-0.20 to 0.13	7.32	0.27 to 14.37	0.15	-0.02 to 0.32
Q4	37.89	34.33 to 41.46	-0.05	-0.14 to 0.03	0.12	-2.06 to 2.31	0.06	-0.05 to 0.17	7.62	2.87 to 12.38	0.09	-0.03 to 0.21
Q5 - least deprived	30.20	28.03 to 32.37	0.02	-0.02 to 0.06	-1.15	-2.24 to -0.05	$9.05e^{-4}$	-0.06 to 0.06	4.21	1.83 to 6.60	0.01	-0.05 to 0.06
**Cost**	£s	95% CI	Δ£s	95% CI	Δ£s	95% CI	Δ£s	95% CI	Δ£s	95% CI	Δ£s	95% CI
Q1 - most deprived	14.25	12.94 to 15.55	0.07	0.05 to 0.09	0.37	-0.24 to 0.98	-0.06	-0.09 to -0.03	1.02	-0.32 to 2.36	-0.02	-0.05 to 0.02
Q2	21.61	17.63 to 25.58	0.21	0.14 to 0.28	-0.88	-2.67 to 0.90	-0.21	-0.30 to -0.12	-2.63	-6.59 to 1.32	-0.18	-0.28 to -0.09
Q3	22.97	19.33 to 26.60	0.20	0.10 to 0.29	0.83	-1.55 to 3.20	-0.25	-0.38 to -0.13	-1.67	-6.95 to 3.62	-0.08	-0.21 to 0.04

For peer review only

For peer review only - http://bmjopen.bmj.com/site/about/guidelines.xhtml

Q4	24.84	21.53 to 28.15	0.22	0.14 to 0.30	0.14	-1.92 to 2.21	-0.24	-0.35 to -0.13	-2.63	-7.26 to 2.01	-0.19	-0.30 to -0.08
Q5 - least deprived	19.78	17.59 to 21.97	0.20	0.17 to 0.24	-0.78	-1.72 to 0.16	-0.18	-0.23 to -0.14	-2.67	-4.76 to -0.58	-0.18	-0.23 to -0.13

95% CI; 95% confidence interval; DDDs; Defined daily doses per 100 patients per practice per month, ΔDDDs; change in DDDs, IR; Incidence rate per 100 patients per practice per month, IRR; incidence rate ratio, £s; gross ingredient cost of medicines before any discount per 100 patients per practice per month, Δ£s; change in £s, Q1-5; quintiles of the Scottish Index of Multiple Deprivation from most to least deprived.

For peer review only

For peer review only - http://bmjopen.bmj.com/site/about/guidelines.xhtml

In response to the incoherence of the findings for DDDs and costs, sensitivity analyses were undertaken, instead of the analyses originally planned to address the third research question (c). Using the Generalized Additive Mixed Models package in R [20-24], the models were fitted without the interrupted time series operators, but fitting 8 knots evenly distributed across the time series. These models allow change points in the time series at times other than those related to the policy to be revealed and relaxes the assumption that any time effects were linear. Plots of each of the models undertaken as part of the sensitivity analyses are show in Supplemental file 3, with the dates of reduction and abolition overlaid. The admissions data for the condition-counterfactual group, show that following the adjustment for seasonality there was a small but linear increase in diabetes-related admissions among adults, which as expected appears not to change in relation to the prescription fee policy changes. However, the admissions data for the intervention and age-counterfactual groups show marked changes which are neither consistent year to year, nor easily related to the policy changes. The patterns of admissions in both the intervention and age-counterfactual groups are similar. Both the DDDs and cost of prescribed medicines data for all three groups show similar patterns of increases, with occasional change points. These change points appear to be spaced in time similarly to the reduction and abolition of prescription fees, but around 12 months after the policy changes. The magnitude of the changes in prescribed DDDs and costs are also greater in the counterfactuals than the intervention group, arguing against any causal effects of the intervention.

DISCUSSION

Using Scottish administrative hospital admissions and prescribed medicines data it was possible to undertake an evaluation of the impact of a major policy change. The evaluation included over 70% of the General Practices in Scotland and analysed changes over an eight-year period. Interrupted time series analysis offered a natural experimental approach for evaluating the impact of policy changes which occurred on specific dates. There was a marked step reduction in adult asthma or COPD related admissions around the time of the abolition of prescription fees (April 2011), compared to the counterfactual groups. This effect was consistent with known health inequalities and the prescription fee policy prior to the change. The data on prescribed medicines were less coherent, with marked changes in the intervention

For peer review only - http://bmjopen.bmj.com/site/about/guidelines.xhtml

and counterfactual groups. Sensitivity analyses revealed that there was considerable variation in almost all the time series that neither related to the policy change, season or annual changes. It was therefore difficult to draw any firm conclusions about the impact of the policy change.

More rigorous and conclusive results would be generated by an experimental study. However, a prospective study of this size and duration would have been considerably more costly, and potentially impossible to fund. Policy changes like the one evaluated are also rarely introduced experimentally. The prescription fee exemption criteria, rather ironically, made this study possible. One challenge to this study was that the prescription fee exemption criteria did not need to be reported following the policy change, so the assumption was made that the proportions eligible for each exemption criteria would not vary markedly in the relatively short period being studied following the policy change. This assumption was supported by the finding that the proportion receiving free prescribed medicines due to older age remained the same, as these people, possibly through habit, continued to report their exemption. However, the variability in the prescriptions time series limited our ability to draw any firm conclusions regarding prescribed medicines. It is a weakness of the study that it was only possible to examine General Practice-level, rather than individual-level data. This prevented the assessment of primary non-adherence and was the result of data not being collected which would permit individual linkage. This linkage is now possible, but not for the years before the policy change. Both SMR-01 and PIS are administrative datasets, collected for billing and reimbursement purposes, and therefore data which is most useful to research is often not collected.

Unlike previous studies into the impact of universal free prescriptions, it has not been possible to identify a clear impact of the policy change in Scotland. The other evaluations of prescription fees have been located in the USA and Canada, where the health systems are quite different to the UK's National Health Service. Kulik, et al.[8] were able to undertake a randomised controlled trial offering post-myocardial infarction patients universal free prescriptions, compared to free prescriptions for medication for the myocardial infarction. The participants in that study had a well-defined need for prescriptions, whereas in the current study – although the intervention group had a long-term condition (asthma or COPD) – the need for regular medication among adults with these conditions will vary considerably between patients. Like the current study, both Campbell, et al.[7] and

For peer review only - http://bmjopen.bmj.com/site/about/guidelines.xhtml

Booth, et al.[9] used administrative data permitting larger retrospective studies. The study from Campbell, et al.[7] is very similar to the current study but examined changes in monthly co-payments. They contrasted the impact of increases of greater or less than $5, which is approximately half the cost of the current English prescription fee. However, this was an increase from the average monthly co-payment of around $17, which is considerably larger than the English prescription fee. Booth, et al.[9] examined the whole diabetic population over 65 years of age, who are more likely to be on numerous medications than the adult (19-59 years of age with asthma or COPD) intervention group in this study. Finally, the data analysed in this study showed that prior to the policy change less than 13% of prescriptions were paid in full or by pre-payment certificate. Subsequently, considering the proportion of that 13% that are for acute conditions, such as bacterial infections, this study may have been looking for a needle in a haystack.

Overall, in this study no marked impact of the abolition of prescription fees in Scotland on prescribed medicines or hospital admissions has been identifiable. However, there were some signs of some impact, specifically a step reduction, within one month, in adult admissions for asthma or COPD. Although, the availability of administrative data made this study possible, it has also brought the biggest limitations to the study. As the data were primarily collected for administrative and financial purposes, it was understandably not optimal for this study, one of the most significant challenges to the use of big data in research [25-27]. Although the PIS data were particularly messy, even having dropped the top 1% of values, the central recording of community prescriptions in Scotland, unlike England, made this study possible. This study also encountered one of the other challenges to research using big data; 'over sensitivity of statistical hypothesis testing' [25,28]. These are at least two of the topics for further research in the field of big data. Based on this study, it is not possible to conclude whether universal free prescriptions is an effective policy in terms of improving health or reducing inequalities. However, neither does the study provide evidence that the policy is ineffective. There has been some concern that not charging for prescriptions could lead to over-use or stock-piling, which for many medications is risky, as well as being a waste of limited resources. The increases in prescriptions in the counterfactual groups may support this hypothesis. Charging a reasonable fee per prescription may limit this; however, the size of the fee required to cover the administrative costs of the payment system may lead to the fee being

For peer review only - http://bmjopen.bmj.com/site/about/guidelines.xhtml

high enough to inappropriately limit pharmaceutical use. Rigorous evaluation need to accompany any health policy changes to inform our understanding of the most efficient health systems and services.

AUTHOR CONTRIBUTIONS

AJW contributed to the conceptualisation of the project, undertook all the statistical analysis and drafted the paper. JF contributed to the conceptualisation of the project, supervised the analysis, reviewed and approved the paper. WH supported AJW with the statistical analysis, reviewed and approved the paper. We would like to acknowledge the support from the other members of the Farr Institute @ Scotland Natural Experimental Approaches research group; Corri Black (University of Aberdeen), Chris Dibben (University of Edinburgh), Ruth Jepson (University of Edinburgh), Anne Ludbrook (University of Aberdeen), and Jill Pell (University of Glasgow). Marion Bennie (University of Strathclyde) also supported this project and helped convene the following clinicians and pharmacists to inform the definition of the intervention and control groups: Simon Hurding, Sean MacBride-Stewart and Margaret Ryan. Finally, we would like to thank Stuart McTaggart and Doug Kidd for their help in accessing and understanding the data.

DATA SHARING STATEMENT

The data analysed within this project are administrative data; the Prescribing Information System (PIS) and the Scottish Morbidity Record – 01 Inpatient (SMR01). Anyone wishing to access the data should apply to the electronic Data Research and Innovation Service (eDRIS): www.isdscotland.org/Products-and-Services/EDRIS/index.asp?Co=Y. Applications to access the data will need to be approved by the Public Benefit and Privacy Panel for Health and Social Care (PBPP): www.informationgovernance.scot.nhs.uk/pbpphsc/. Instructions and guidance on how to apply to access the data can be found here: www.informationgovernance.scot.nhs.uk/pbpphsc/home/for-applicants/.

REFERENCES

1 Scottish Executive. Review of NHS prescription charges and exemption arrangements in Scotland - consultation. Edinburgh. Scottish Executive, 2006.

For peer review only - http://bmjopen.bmj.com/site/about/guidelines.xhtml

Website: http://www.scotland.gov.uk/Resource/Doc/90909/0021837.pdf (accessed 11 April 2018)

2 NHS Choices. Get help with prescription costs. London. Gov.uk, 2017. Website: https://www.nhs.uk/nhsengland/Healthcosts/pages/Prescriptioncosts.aspx (accessed 11 April 2018)

3 British Broadcasting Corporation. Prescription charges abolished in Scotland. London. British Broadcasting Corporation, 2011. Website: http://www.bbc.co.uk/news/uk-12928485 (accessed 11 April 2018)

4 Roehr B. Cost keeps one in 10 Canadians from filling prescription. *BMJ* 2012;344. doi:10.1136/bmj.e514 [published Online: 20 January 2012]

5 Citizens Advice Scotland. Prescription charges. Edinburgh. The Scottish Association of Citizens Advice Bureaux - Citizens Advice Scotland, 2006. Website: http://www.cas.org.uk/publications/prescription-charges (accessed 11 April 2018)

6 The Scottish Parliament. Summary of responses and conclusions: abolition of NHS prescription charges (Scotland) consultation. Edinburgh. The Scottlish Parliament, n.d. Website: http://www.scottish.parliament.uk/S2_MembersBills/Final%20proposals/abolition-prescription-charges-response.pdf (accessed 11 April 2018)

7 Campbell JD, Allen-Ramey F, Sajjan SG, et al. Increasing pharmaceutical copayments: impact on asthma medication utilization and outcomes. *Am J Manag Care* 2011;17:703-10.

8 Kulik A, Desai NR, Shrank WH, et al. Full prescription coverage versus usual prescription coverage after coronary artery bypass graft surgery: analysis from the post-myocardial infarction Free Rx Event and Economic Evaluation (FREEE) randomized trial. *Circulation* 2013;128:S219-S25. doi:10.1161/circulationaha.112.000337 [published Online: 20 September 2013]

9 Booth GL, Bishara P, Lipscombe LL, et al. Universal drug coverage and socioeconomic disparities in major diabetes outcomes. *Diabetes Care* 2012;35:2257-64. doi:10.2337/dc12-0364 [published Online: 23 October 2012]

10 NHS Wales. Helping to improve Wales's health: free prescriptions three years on. Cardiff. NHS Wales, 2010. Website:

For peer review only - http://bmjopen.bmj.com/site/about/guidelines.xhtml

http://www.wales.nhs.uk/documents/prescriptions-report-three-years.pdf (accessed 11 April 2018)

11 Jewell T. Have dispensed items really risen with free prescriptions? *BMJ* 2008;337. doi:10.1136/bmj.a1581 [published Online: 10 September 2008]

12 Groves S, Cohen D, Alam MF, et al. Abolition of prescription charges in Wales: the impact on medicines use in those who used to pay. *International Journal of Pharmacy Practice* 2010;18:332-40. doi:10.1111/j.2042-7174.2010.00063.x [published Online: 5 October 2010]

13 Dhippayom T, Walker R. Impact of the reduction of the prescription charge in Wales on the prescribing of non-sedating antihistamines in primary care. *Health Policy* 2008;87:309-15. doi:10.1016/j.healthpol.2008.01.006 [published Online: 6 March 2008]

14 Purdy S, Griffin T, Salisbury C, et al. Ambulatory care sensitive conditions: terminology and disease coding need to be more specific to aid policy makers and clinicians. *Public Health* 2009;123:169-73. doi:10.1016/j.puhe.2008.11.001 [published Online: 13 January 2009]

15 The Scottish Public Health Observatory. Asthma: key points. Glasgow. The Scottish Public Health Observatory, 2017. Website: http://www.scotpho.org.uk/health-wellbeing-and-disease/asthma/key-points/ (accessed 12 April 2018)

16 The Scottish Public Health Observatory. Chronic obstructive pulmonary disease (COPD): key points. Glasgow. The Scottish Public Health Observatory, 2017. Website: http://www.scotpho.org.uk/health-wellbeing-and-disease/chronic-obstructive-pulmonary-disease-copd/key-points/ (accessed 12 April 2018)

17 The Scottish Public Health Observatory. Diabetes: key points. Glasgow. The Scottish Public Health Observatory, 2017. Website: http://www.scotpho.org.uk/health-wellbeing-and-disease/diabetes/key-points/ (accessed 12 April 2018)

18 Stata statistical software: release 14 [program]. College Station, TX: StataCorp LP, 2015.

19 Scottish Government. The Scottish Index of Multiple Deprivation. Edinburgh. Scottish Government, 2017. Website: http://www.gov.scot/Topics/Statistics/SIMD (accessed 11 April 2018)

For peer review only

20 Wood SN. Thin plate regression splines. *J R Stat Soc Series B Stat Methodol* 2003;65:95-114. doi:10.1111/1467-9868.00374 [published Online: 28 January 2003]

21 Wood SN. Stable and efficient multiple smoothing parameter estimation for generalized additive models. *J Am Stat Assoc* 2004;99:673-86. doi:10.1198/016214504000000980 [published Online: 01 January 2012]

22 Wood SN. Fast stable restricted maximum likelihood and marginal likelihood estimation of semiparametric generalized linear models. *J R Stat Soc Series B Stat Methodol* 2011;73:3-36. doi:10.1111/j.1467-9868.2010.00749.x [published Online: 14 September 2010]

23 Wood SN. *Generalized additive models: an introduction with R*. Second edition. ed. Boca Raton, FL: CRC Press/Taylor & Francis Group, 2017.

24 Wood SN, Pya N, Säfken B. Smoothing parameter and model selection for general smooth models. *J Am Stat Assoc* 2016;111:1548-63. doi:10.1080/01621459.2016.1180986 [published Online: 04 January 2017]

25 Stricker BH. Epidemiology and 'big data'. *Eur J Epidemiol* 2017;32:535-36. doi:10.1007/s10654-017-0294-3 [published Online: 17 August 2017]

26 The Academy of Medical Sciences. Improving the health of the public by 2040: optimising the research environment for a healthier, fairer future. London. The Academy of Medical Sciences, 2016. Website: http://www.acmedsci.ac.uk/viewFile/57e4b532a4308.pdf (accessed 11 April 2018)

27 Royal Statistical Society. The opportunities and ethics of big data. London. Royal Statistical Society, 2016. Website: http://www.rss.org.uk/Images/PDF/influencing-change/2016/rss-report-opps-and-ethics-of-big-data-feb-2016.pdf (accessed 11 April 2018)

28 Kitchin R. Big Data, new epistemologies and paradigm shifts. *Big Data Soc* 2014;1:2053951714528481. doi:10.1177/2053951714528481 [published Online: 01 April 2014]

Figure 1 – Prescription fees in Scotland compared to England; (a) fee per item, (b) equivalent number of items purchased per month using prepayment certificates

For peer review only - http://bmjopen.bmj.com/site/about/guidelines.xhtml

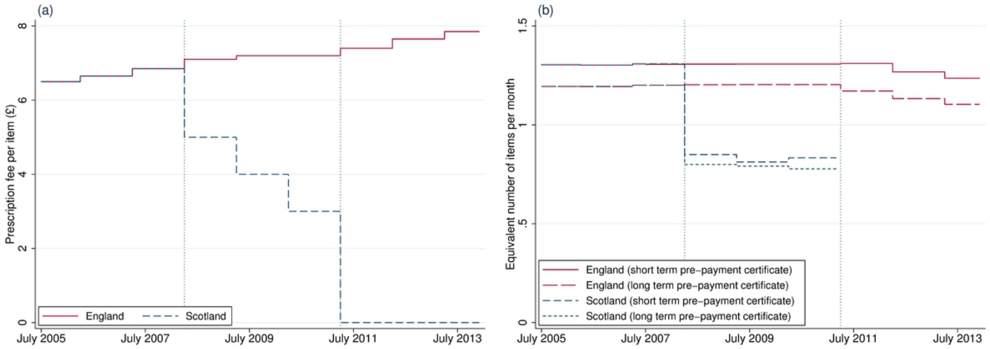

Figure 1 – Prescription fees in Scotland compared to England; (a) fee per item, (b) equivalent number of items purchased per month using prepayment certificates

101x36mm (300 x 300 DPI)

Supplemental file 1 Study protocol

Farr Institute @ Scotland – NEA group / SCPHRP
Andrew James Williams

Natural Experiment

'Universal' Free Prescriptions in Scotland

eDRIS project number: ED13-0487
Primary contact: Andrew James Williams
Other team members: John Frank, Ruth Jepson, Jill Pell, Chris Dibben, Anne Ludbrook and Marion Bennie.

Background

Following devolution each of the four nations of the United Kingdom (UK) (England, Wales, Scotland and Northern Ireland) have taken different approaches to subsidising and funding prescribed medications (British Broadcasting Corporation, 2011). Only England continues to charge a prescription fee which stands at £7.85 in 2013 (Department of Health, 2013). When Scotland abolished the prescription charge the then Health Secretary, Nicola Sturgeon, described prescription fees as 'a tax on ill-health' (British Broadcasting Corporation, 2011).

For those with chronic conditions one of the purposes of pharmaceuticals is to control the condition and subsequently reduce the need for hospitalisation and more invasive and costly intervention. Purdy et al. (2009) have defined a list of conditions sensitive to ambulatory care which are listed in Appendix 1. The appropriate management and treatment of these conditions in primary care should prevent the need for admission into secondary care (Purdy et al., 2009). The success of pharmaceutical treatments is dependent upon the patient's adherence to the dosage regimen prescribed by the practitioner. Kardas et al. (2013) recently reviewed the determinants of non-adherence. Seven hundred and seventy one determinants of whether someone initiated and/or persisted with a medication were identified which fell into the following five broad clusters; socioeconomic-related, healthcare team- and system-related, condition-related, therapy-related, and patient-related (Kardas et al., 2013). Among the socioeconomic cluster the eight studies which examined cost, all found higher cost to be associated with poorer adherence (Kardas et al., 2013). Therefore, the economic logic behind the abolition of prescription fees is that adherence will improve, and therefore hospital admissions will decrease reducing secondary care costs, which hopefully compensate for the additional cost to the state of the prescriptions (Figure 1).

Previous research has tended to focus on elements of the logic model rather than the whole. Linnet et al. (2013), Yin et al. (2008) and Zhang et al. (2008) focussed upon the first half of the logic model, the effect of prescription fee policies on the dispensing of pharmaceuticals with mixed results. Yin et al. (2008) and Zhang et al. (2008) found that the introduction of prescription subsidies (Medicare part D) for the elderly in America resulted in an increased number of prescriptions and reduced out-of-pocket expenses for the patient. Whereas Linnet et al. (2013) found that within Iceland a small increase in prescription charge did not significantly affect whether a patient cashed/filled a prescription. Booth et al. (2012) when examining the socioeconomic disparities in diabetes mellitus related morbidity and mortality found that the socioeconomic disparities greatly reduced in each of the outcomes studied for those over 65 years of age (Booth et al., 2012). Within Ontario, Canada (the location of the study) those over the age of 65 receive free prescriptions and therefore the decreased socioeconomic disparities was attributed to this policy (Booth et al., 2012). Booth et al. (2012) employed a number of

For peer review only - http://bmjopen.bmj.com/site/about/guidelines.xhtml

statistical techniques to test this hypothesis (sensitivity analyses) and continued to find the decreased disparities associated with the free prescription policy. Campbell et al. (2011) and Kulik et al. (2013) employed more experimental study designs to test the logic model. Kulik et al. (2013) undertook a randomised controlled trial into the effect of offering free prescriptions to patients immediately following a myocardial infarction and found improved adherence and reduced patient out-of-pocket expenses and a trend towards improved clinical outcomes. Campbell et al. (2011) used linked data within a natural experimental approach to examine the effect of prescription costs for those with asthma. It was found that those experiencing increases in prescription charges (co-payment) ≥$5(US) were significantly less adherent than those experiencing increases of <$5 and also had higher healthcare usage (Campbell et al., 2011).

Figure 1 – Logic model for study

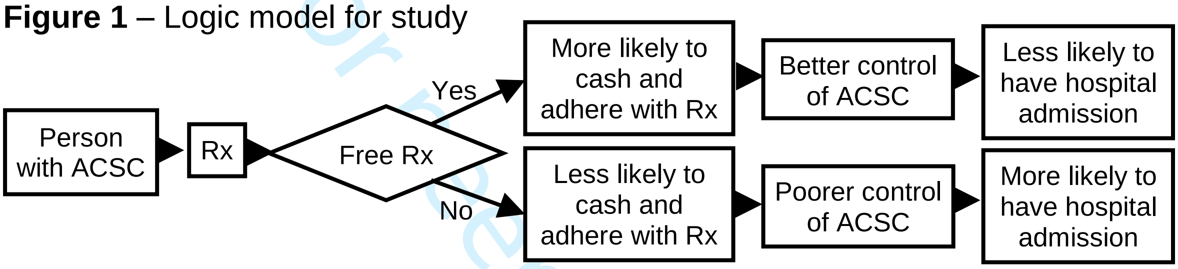

ACSC; ambulatory case sensitive condition, Rx; prescription

Subsequently, there is some evidence that reduced or free prescriptions have positive effects on adherence and health. However, the studies so far have focused upon certain age groups or conditions and yet within Wales, Northern Ireland and Scotland prescriptions are free for all. Three years after the abolition of prescription fees in Wales a process evaluation was published by the health authority (NHS Wales, 2010). This evaluation found that the abolition of prescription fees did not result in a change in the number of prescriptions and their cost to the state. Jill Pell and colleagues are undertaking a similar study examining the first half of the logic model (Figure 1) in Scotland. However, to the best of our knowledge there have been no studies within the UK to examine the whole logic model in relation to the universal free prescription policy.

Aims

To identify whether the stepped reduction and abolition of prescription fees in Scotland resulted in:

a) increase in the number (cost to NHS) of prescriptions for inhaled steroids;
b) reduction in hospital admissions for asthma or chronic obstructive pulmonary disease (COPD).

When compared to prescriptions and admissions for a condition for which prescriptions were historically free (diabetes mellitus) and or those who received free prescriptions due to age.

Moving of from these preliminary aims more refined analysis is planned to address these two further questions.

c) Was any change in hospital admissions for asthma or COPD in the practices which also saw a change in prescribing/dispensing?
d) How did the effect differ across the socioeconomic spectrum?

For peer review only - http://bmjopen.bmj.com/site/about/guidelines.xhtml

Supplemental file 1 Study protocol

Farr Institute @ Scotland – NEA group / SCPHRP
Andrew James Williams

Proposal

As a policy the abolition of prescription fees was not introduced experimentally (e.g. with randomisation), and subsequently we propose to address the above aims using time series of routinely collected data as a natural experiment (Craig et al., 2011). The primary sources for the routinely collected data will be the Scottish Morbidity Record (SMR01) and the Prescriptions Information System (PIS). Through extensive discussion with the Principle Pharmacist (Stuart McTaggart) at NHS National Services Scotland, it has been established that individual-level data suitable for this project are not currently available in the PIS. The primary reasons that individual-level data from the PIS cannot be used are the inability to calculate measures of adherence (Fairman and Matheral, 2000, Lehmann et al., 2013), and the fact that currently individual-level data are not available prior to August 2009 when Scotland had already begun reducing prescription fees. Subsequently, we propose to proceed with a practice-level study as outlined below. Currently, the proposed study is limited to Scotland, although through the Farr network a UK wide study may be considered at a later date.

Critical to any natural experiment is the identification of the population who were/are effected by the policy (the intervention sample) and appropriate counterfactual sample(s) (Craig et al., 2011). As prescription fees were reduced and ultimately abolished contemporaneously across Scotland and the data being used only exist in Scotland there is no location-based contemporary counterfactual. However, the existence of prescription fee exemption criteria (Appendix 2) prior to the abolition of fees permits the identification of the intervention and counterfactual samples. Although, as following the abolition of prescription fees there was no need to record exemption criteria, the samples also had to remain identifiable within the PIS and SMR01. Furthermore, as only one exemption criteria needed to be recorded when collecting a prescription, it was and will remain necessary throughout this project to remain aware that although marked exempt for one reason the patient may have also met other exemption criteria.

The exemption criteria (Appendix 2) fall into three groups, age- condition- and income-related all of which could have formed the basis of counterfactual samples. The income-related exemptions group may have permitted a regression discontinuity design, however, it would not be possible to identify this sample following the abolition of fees and consequently no income-based counterfactual was sought. The age-related exemption criteria would form an age-based counterfactual sample. This is made possible as prior to the abolition of fees the PIS exemptions category data would identify this sample and following the fee abolition individual-level PIS data could inform the identification of this group. Those aged 16-18 years in full time education also received free prescriptions (Appendix 2); as this subpopulation can only be identified prior to the abolition of fees they will initially be examined separately to ascertain what approach is most appropriate for this subpopulation. However, wanting to strengthen the causal inference it is planned to examine dispensed prescriptions as a mediator of change in health (admissions) and consequently it is necessary to identify suitably sensitive and specific condition medication pairings. Thus, the condition-related exemption criteria were crucial. These pairings needed to meet the following requirements;

- The condition needed to be chronic (e.g. not ear, nose and throat infections).

For peer review only - http://bmjopen.bmj.com/site/about/guidelines.xhtml

Supplemental file 1 Study protocol

- The condition needed to be sensitive to long term pharmaceutical treatment specifically not just ambulatory care in general (e.g. not dental complications).
- Relatively minor changes in adherence (e.g. missed tablets) to the treatment for the condition needed to result in the need for hospital attention (e.g. not schizophrenia or epilepsy).
- The condition needed to be sufficiently prevalent across the life-course to provide sufficient power for the analysis (e.g. not senility/dementia).
- The intervention condition needed to not be a condition-related exemption from prescription fees (Appendix 2) or commonly co-occurring with a condition on that list (e.g. not diabetes mellitus or any of the cardiovascular conditions).
- The counterfactual condition needed to be a condition-related exemption from prescription fees (e.g. diabetes mellitus).
- The pharmaceutical treatment (medicine) needs to be fairly specific to the condition (e.g. not congestive heart failure).

The final samples are outlined in the box below, with asthma/COPD selected as the intervention condition. Asthma/COPD had been noted for their absence from the exemption criteria in the Scottish public consultation about the abolition of prescription fees (Scottish Executive, 2006, The Scottish Parliament, n.d.). These samples have been defined after extensive consultation with expert pharmacists and general practitioners.

**Sample**	**Eligibility criteria**
Intervention	Those aged ≥16-<60 years that had paid for dispensed prescriptions for steroid inhalers which is assumed to indicate diagnosis with asthma or chronic obstructive pulmonary disease (COPD).
Age-based counterfactual	Those age <16 or ≥60 years that had dispensed prescriptions for steroid inhalers which is assumed to indicate diagnosis with asthma or COPD.
Condition-based counterfactual	Those that had dispensed prescriptions for diabetes mellitus treatments which are assumed to indicate diagnosis with said condition.

As only one exemption criteria would have been recorded for each prescription it is not possible to sub-divide the condition-based counterfactual by age. As previously discussed the analysis is forced to examine practice- rather than individual-level data and subsequently the data will be numbers/proportions of relevant prescriptions/admissions for each practice.

Analysis

The studies discussed in the penultimate paragraph of the Background to this document employed a number of statistical methods ranging from the simple univariable analysis to complicated hierarchical multivariable models taking in techniques such as logistic regression (Booth et al., 2012, Kulik et al., 2013, Yin et al., 2008, Zhang et al., 2008) and Cox proportional hazard models (Booth et al., 2012, Kulik et al., 2013). The data to be collected, linked and analysed in the current

For peer review only - http://bmjopen.bmj.com/site/about/guidelines.xhtml

project is outlined in Appendix 3, the intention being to produce a panel dataset. For each practice there will be annually 'fixed' socio-demographic data from the General Practice dataset, and monthly (January 2000-December 2013) admissions and prescriptions data. Analysis will be undertaken in Stata (StataCorp, 2009) and will take the format of time series and mediation analysis.

The primary health outcome will be hospital admissions for the respective conditions. The PIS extract dataset will provide outcome data on the potential mediators of any change in the primary outcome. Without individual data and the ability to measure adherence, a number of prescription related metrics will be evaluated as outcomes and mediators. The following metrics will be examined for each of the medication groups (inhaled steroids and diabetes treatments);

- Number of dispensed prescriptions,
- Quantity dispensed,
- Cost of the items dispensed,
- Number of Defined Daily Doses (DDDs) dispensed.

Including the admissions, but excluding costs all the outcomes are count data and consequently will be analysed as such (Poisson regression Generalised Estimating Equations (GEE) or Mixed Models). Cost will be analysed as a continuous outcome. Initially some of the outcomes may be turned into ratios (e.g. DDDs per prescription or prescriptions per general practitioner) in order to smooth the data and account for differences in practice size, to identify the most appropriate set of outcomes. Auto-regression, integration (differencing) and moving average (ARIMA) adjustments will also be explored, to smooth the data and in particular account for the seasonality of dispensing.

Figure 2 – Mediation model for analysis

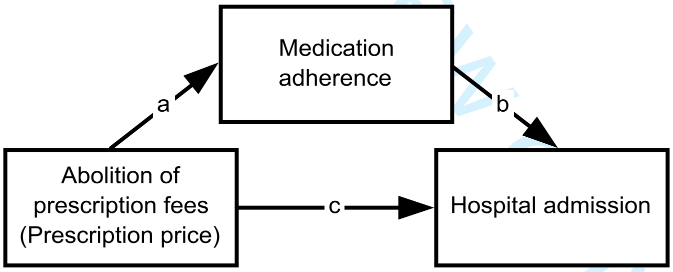

Taking the study aims and the logic model (Figure 1) Figure 2 illustrates the mediation model to be examined through the analysis. The studies discussed in the background provide evidence of relationships **a** and **b** in Figure 2. The preliminary aims of this study (study aims a and b) will be addressed by constructing separate time series of prescriptions and admissions in order to test relationships **a** and **c**. As a practice-level study it will not be possible to directly test relationship **b**. As with previous studies (e.g. Yin et al., 2008, Booth et al., 2012) splines and knots will be employed in order to assess whether changes in admissions and/or prescriptions were contemporary with the reductions and eventual abolition of fees. Furthermore, the use of splines and knots will permit some quantification of any effect of the policy change. The admissions time series will then be adjusted for the prescriptions time series (study aim c), in order to assess the extent to which relationship **c** remains, which will provide information on the nature of relationship **b**. Should relationship **c** remain after adjusting for prescriptions, it would indicate that within the limitations of

For peer review only - http://bmjopen.bmj.com/site/about/guidelines.xhtml

this study the policy change had not impacted on health. These analytical steps should strengthen to the study findings.

Given the extent of exemptions criteria (Appendix 2), during the Scottish public consultation it was questioned who would benefit from abolishing the fee (Scottish Executive, 2006, The Scottish Parliament, n.d.). The exemption criteria (Appendix 2) were (and still are in England) considered to be protecting the poorest and sickest who would struggle to pay. It was felt that a reappraisal of the condition-related exemption criteria to consider including conditions like asthma, COPD, dementia might be a sufficient change. As it was, prior to the abolition of prescription fees around 90% of prescriptions were exempt from payment (Personal communication with S. McTaggart, 01/04/2014). In order to examine for the differential impact of the policy across the socioeconomic spectrum (study aim d), the effect of adjusting or stratifying the time series by deprivation and other General Practice characteristics will be explored.

Special cases: As previously discussed those aged 16-18 years will initially be examined separately. Similarly, there is another group of patients who will initially be examined separately. Prior to the abolition of fees, those with chronic conditions were able to purchase discount pre-payment certificates. The price of these certificates changed in line with the reduction in fees. Data published in 2009 demonstrated that there had been a significant increase in the use of pre-payment certificates following the initial reduction in prescription fees (The Scottish Government, 2009). That finding and the different financial decisions related to pre-payment certificates have necessitated the initial examination of this group separately. The use of a pre-payment certificate was captured within the exemptions data within the PIS.

Potential impact

From a research perspective this project would add to the literature on the importance of cost as a determinant of non-adherence with prescribed medications. Given the large number of exemptions from prescription fees prior to the policy change (see Rationale) some have questioned whether universal free prescriptions are just benefiting the less deprived. This study should reveal what the implications of universal free prescriptions have been for those of different socioeconomic status. Some have also questioned whether having to pay for a prescription is an incentive to adherence, although there does not appear to be evidence to back this up, this needs to be considered within this project, the results of which might support or refute that claim. Politically, the project will evaluate an important and possibly costly policy and provide data on the 'effects' of the policy. In particular, should a time arise that free prescriptions for all cannot be funded by the state; the stratified component of this project might suggest conditions or circumstances for which free prescriptions are cost effective.

For peer review only - http://bmjopen.bmj.com/site/about/guidelines.xhtml

Supplemental file 1 Study protocol

Farr Institute @ Scotland – NEA group / SCPHRP
Andrew James Williams

References

Booth, G. L., Bishara, P., Lipscombe, L. L., Shah, B. R., Feig, D. S., Bhattacharyya, O. & Bierman, A. S. 2012. Universal drug coverage and socioeconomic disparities in major diabetes outcomes. *Diabetes Care,* 35**,** 2257-2264.

British Broadcasting Corporation. 2011. *Prescription charges abolished in Scotland* [Online]. London: British Broadcasting Corporation. Available: http://www.bbc.co.uk/news/uk-12928485 [Accessed 24 September 2013].

Campbell, J. D., Allen-Ramey, F., Sajjan, S. G., Maiese, E. M. & Sullivan, S. D. 2011. Increasing pharmaceutical copayments: impact on asthma medication utilization and outcomes. *Am J Manag Care,* 17**,** 703-10.

Craig, P., Cooper, C., Gunnell, D., Haw, S., Lawson, K., Macintyre, S., Ogilvie, D., Petticrew, M., Reeves, B., Sutton, M. & Thompson, S. 2011. *Using natural experiments to evaluate population health interventions: guidance for producers and users of evidence,* London, Medical Research Council. Available: http://www.mrc.ac.uk/documents/pdf/natural-experiments-guidance/ [Accessed 26 February 2014]

Department of Health. 2013. *NHS charges from April 2013 announced* [Online]. London: GOV.UK. Available: https://www.gov.uk/government/news/nhs-charges-from-april-2013-announced [Accessed 15 October 2013].

Fairman, K. & Matheral, B. 2000. Evaluating medication adherence: which measure is right for your program? *Journal of Managed Care Pharmacy,* 6**,** 499-506.

Kardas, P., Lewek, P. & Matyjaszczyk, M. 2013. Determinants of patient adherence: a review of systematic reviews. *Frontiers in Pharmacology,* 4(91).

Kulik, A., Desai, N. R., Shrank, W. H., Antman, E. M., Glynn, R. J., Levin, R., Reisman, L., Brennan, T. & Choudhry, N. K. 2013. Full prescription coverage versus usual prescription coverage after coronary artery bypass graft surgery: analysis from the post-myocardial infarction Free Rx Event and Economic Evaluation (FREEE) randomized trial. *Circulation,* 128**,** S219-S225.

Lehmann, A., Aslani, P., Ahmed, R., Celio, J., Gauchet, A., Bedouch, P., Bugnon, O., Allenet, B. & Schneider, M. P. 2013. Assessing medication adherence: options to consider. *Int J Clin Pharm*, 36(1):55-69.

Linnet, K., Halldórsson, M., Thengilsdóttir, G., Einarsson, Ó. B., Jónsson, K. & Almarsdóttir, A. B. 2013. Primary non-adherence to prescribed medication in general practice: lack of influence of moderate increases in patient copayment. *Family Practice,* 30**,** 69-75.

NHS Choices. 2013. *Get help with prescription costs* [Online]. London: GOV.UK. Available: http://www.nhs.uk/NHSEngland/Healthcosts/Pages/Prescriptioncosts.aspx [Accessed 15 October 2013].

NHS Wales 2010. *Helping to improve Wales's health: free prescriptions three years on,* Cardiff, NHS Wales. Available: http://www.wales.nhs.uk/documents/prescriptions-report-three-years.pdf [Accessed 25 September 2013]

Purdy, S., Griffin, T., Salisbury, C. & Sharp, D. 2009. Ambulatory care sensitive conditions: terminology and disease coding need to be more specific to aid policy makers and clinicians. *Public Health,* 123**,** 169-173.

Scottish Executive. 2006. *Review of NHS prescription charges and exemption arrangements in Scotland - consultation* [Online]. Edinburgh: Scottish Executive. Available:

For peer review only - http://bmjopen.bmj.com/site/about/guidelines.xhtml

http://www.scotland.gov.uk/Resource/Doc/90909/0021837.pdf [Accessed 19 February 2014].

StataCorp 2009. Stata statistical software: release 11. College Station, TX: StataCorp LP.

The Scottish Government. 2009. *News; prescription charges* [Online]. Edinburgh: The Scottish Government. Available: http://www.scotland.gov.uk/News/Releases/2009/01/28095040 [Accessed 28 January 2014].

The Scottish Parliament. n.d. *Summary of responses and conclusions: abolition of NHS prescription charges (Scotland) consultation* [Online]. Edinburgh: The Scottish Parliament. Available: http://www.scottish.parliament.uk/S2_MembersBills/Final%20proposals/abolition-prescription-charges-response.pdf [Accessed 19 February 2014].

Yin, W., Basu, A., Zhang, J. X., Rabbani, A., Meltzer, D. O. & Alexander, G. C. 2008. The effect of the Medicare Part D prescription benefit on drug utilization and expenditures. *Ann Intern Med,* 148**,** 169-77.

Zhang, J. X., Yin, W., Sun, S. X. & Alexander, G. C. 2008. The impact of the Medicare Part D prescription benefit on generic drug use. *J Gen Intern Med,* 23**,** 1673-8.

Appendix 1: Ambulatory case sensitive conditions Purdy et al. (2009)

Angina	Nutritional deficiency	Hypokalaemia	Stroke
Asthma	Hypertension	Low birth weight	Pyelonephritis
Cellulitis	Failure to thrive	Neuroses	Diabetes complications
Dental complications	Ruptured appendix	Tuberculosis	Gangrene
Constipation	Deliberate self-harm	Senility/dementia	Schizophrenia
Congestive heart failure	Fractured proximal femur	Influenza and pneumonia	Migraine/acute headache
Iron-deficiency anaemia	Alcohol-related disease	Atrial fibrillation/flutter	Convulsions and epilepsy
Dehydration and gastroenteritis	Perforated/bleeding ulcer	Ear, nose and throat infections	Pelvic inflammatory disease
Other vaccine-preventable diseases	Dyspepsia and other stomach function disorders	Chronic obstructive pulmonary disease	Peripheral vascular disease

Appendix 2: Criteria exempting patients from paying prescription fees in England and Scotland prior to April 2011 (NHS Choices, 2013).

- Condition-related
  - Cancer (from 2009)
  - Those on renal dialysis with a permanent fistula
  - Pregnant
  - Contraceptives
  - Sexually transmitted infection
  - Tuberculosis

For peer review only - http://bmjopen.bmj.com/site/about/guidelines.xhtml

Supplemental file 1 Study protocol

  - Hypoadrenalism for which specific substitution therapy is essential
  - Diabetes insipidus or other forms of hypopituitarism
  - Diabetes mellitus, except where treatment is by diet alone
  - Hypoparathyroidism
  - Myasthenia gravis
  - Myxoedema
  - Epilepsy requiring continuous anticonvulsive therapy
  - A continuing physical disability which means the person cannot go out without the help of another person.
- Age-related
  - are 60 or over
  - are under 16
  - are 16-18 and in full-time education
- Means tested, and those in receipt of the following benefits ('income-related')
  - Income Support
  - Income-based Jobseeker's Allowance
  - Income-related Employment and Support Allowance
  - Pension Credit Guarantee Credit
  - Universal Credit
  - Those in receipt of a war pension

Appendix 3: Data
The final study dataset will be compiled from three separate datasets. Two of these datasets will be extracts from the Scottish Morbidity Record (SMR01) and Prescriptions Information System (PIS). The third dataset will be the General Practice data compiled from data published by ISD Scotland (http://www.isdscotland.org/Health-Topics/General-Practice/) and more historical data provided through eDRIS. The General Practice dataset will be linked by the researcher using the practice code. The SMR01 and PIS data extracts will be linked by the researcher using the practice code, year and month. The General Practice dataset will then be linked onto the linked SMR01 and PIS data, and the data rearranged in order that each month of SMR01 and PIS data will only have the relevant years general practice descriptive variables. The final dataset should be in the form of panel data for the time series analysis. The variables in each of the three datasets are listed below.

N.B. Gender and ethnicity data are not available in the practice-level PIS and have subsequently not been sought in the other data.

For peer review only - http://bmjopen.bmj.com/site/about/guidelines.xhtml

Supplemental file 1 Study protocol

Farr Institute @ Scotland – NEA group / SCPHRP
Andrew James Williams

General Practice dataset – by practice and year

Variable	Details
NHS board (code)	Unique single alphabetical character for each health board (publically available)*
NHS board (name)	Name of each health board (publically available)*
Practice Code	The unique NHS Scotland code for the GP Practice (publically available)
Practice name	Name of each General Practice (publically available)*
Number of General Practitioners	(requested for each year through eDRIS)
Dispensing practice	Identifier of practices where medicines are dispensed as well as prescribed (requested for each year through eDRIS)
Contract type	Indicator of which type of contract the practice has with the NHS (requested for each year through eDRIS)
Practice List Size	Total number of registered patients each year (publically available for the years 1999-2013)
Age profile of practice list	Number of patients in each of the following age categories; <16, 16-18, 18-60, ≥60 years (requested for each year through eDRIS)
pc_most_dep15	Percentage of practice patients living in datazones defined as the 15% most deprived (population weighted) (publically available from 2005-2013, earlier years requested through eDRIS)
quint1 – quint5	Number of practice patients matched to the relevant first through fifth quintile of deprivation (publically available from 2005-2013, earlier years requested through eDRIS)
prac_ur8	Urban/rural classification of the postcode of the General Practice (publically available from 2005-2013, earlier years requested through eDRIS)
urban1 – urban8	Number of practice patients in each of the eight urban/rural classifications (publically available from 2005-2013, earlier years requested through eDRIS)
unmatch	Number of practice patients who could not be matched to an urban/rural classification (publically available from 2005-2013, earlier years requested through eDRIS)
mode_ur8	Modal urban/rural classification of the practice patients (publically available from 2005-2013, earlier years requested through eDRIS)

*These data are for linking and/or clustering purposes and would be anonymised in any publications.

For peer review only - http://bmjopen.bmj.com/site/about/guidelines.xhtml

Supplemental file 1 Study protocol

Farr Institute @ Scotland – NEA group / SCPHRP
Andrew James Williams

Scottish Morbidity Record (SMR01) extract dataset – by practice, year and month

**Variable**	**Details**
Practice Code	The unique NHS Scotland code for the GP Practice
Year	The year to which the data relates (2000-13)
Month	The month to which the data relates
Diagnosis	The diagnosis (based on DIAGNOSIS 1) to which the data relates (ALL admissions, Asthma/COPD† or Diabetes‡
Age group	The age banding (0-15, 16-18, 19-59, 60+ years) to which the data relates
Admissions	The total number of admissions for practice patients

†International Classification of Disease 10 codes J20, J40X, J41, J42, J43, J44, J45, J46 or J47
‡International Classification of Disease 10 codes E10, E11, E12, E13 or E14

Prescriptions Information System (PIS) extract dataset – by practice, year and month

**Variable**	**Details**
Practice Code	The unique NHS Scotland code for the GP Practice
Year	The year to which the data relates (2000-13)
Month	The month to which the data relates
Category	The medication class to which the data relates (ALL prescriptions, Inhaled Corticosteroids or Diabetes)
Paid/Exempt	A flag to indicate whether the data relates to Paid or Exempt prescriptions
Exemption Category*	The prescription exemption category to which the data relates
Number of Items Paid	The number of prescription items paid
Quantity Paid	The total quantity paid
Gross Ingredient Cost Paid	The total cost of the medicines supplied before discount
DDDs Paid	The total number of defined daily doses paid

*For data from April 2011, exemption category will not be available. This should be replaced by the following age-bandings: 0-15, 16-18, 19-59; 60+ years. Ages should be based on the date of prescribing.

For peer review only - http://bmjopen.bmj.com/site/about/guidelines.xhtml

Supplemental file 2 – National Health Service Prescription Fee exemption Criteria [1]

Medical Grounds:

- Forms of hypoadrenalism (including Addison's disease) for which specific substitution therapy is essential
- Diabetes insipidus or other forms of hypopituitarism
- Diabetes mellitus (except where treatment is by diet alone)
- Hypoparathyroidism
- Myasthenia gravis
- Myxodoema (hypothyroidism requiring thyroid hormone replacement)
- Epilepsy requiring continuous anti-convulsive therapy
- A permanent fistula, requiring an appliance or continuous surgical dressing
- Continuing physical disability which prevents the patient from leaving their residence without the help of another person
- Cancer (added in 2009) including any prescriptions for current or previous cancer treatment

Pregnant or had a baby in the previous 12 months (these are included with the medical exemptions)
Contraceptives (these are included with the medical exemptions)
Age:

- Under 16 years
- 16-18 years and in full time education
- Over 60 years

Financial situation:

- Those receiving Income Support, Income-Based Job Seeker's Allowance, Income-related Employment and Support Allowance, Pension Credit Guarantee Credit, Universal Credit and people who qualify for full help under the NHS Low Income Scheme are exempt.

For peer review only

References

1 NHS Choices. Get help with prescription costs. London. Gov.uk, 2017. Website: https://www.nhs.uk/nhsengland/Healthcosts/pages/Prescriptioncosts.aspx (accessed 11 April 2018)

Supplemental file 3 Sensitivity analysis

Admissions

Intervention

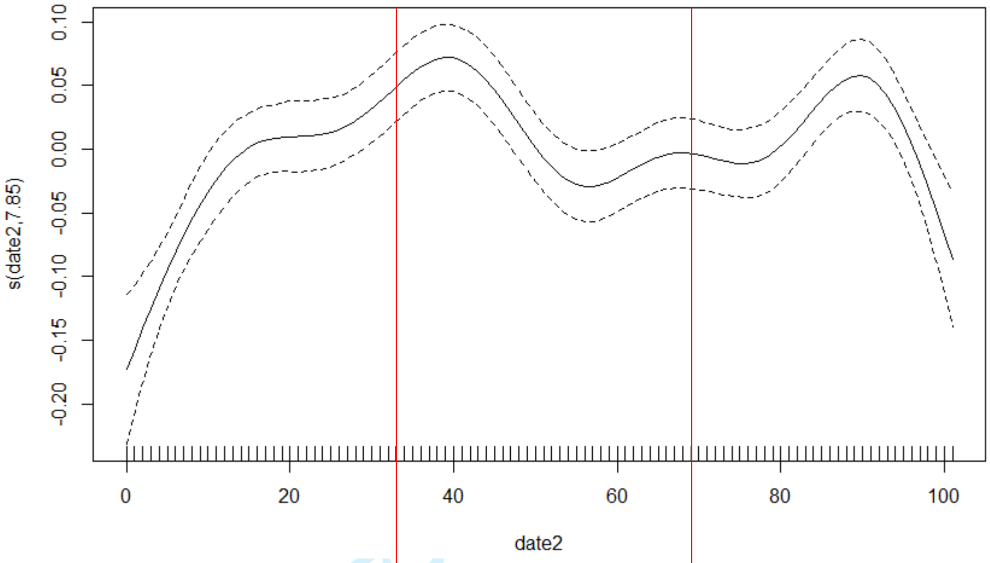

Age counterfactual

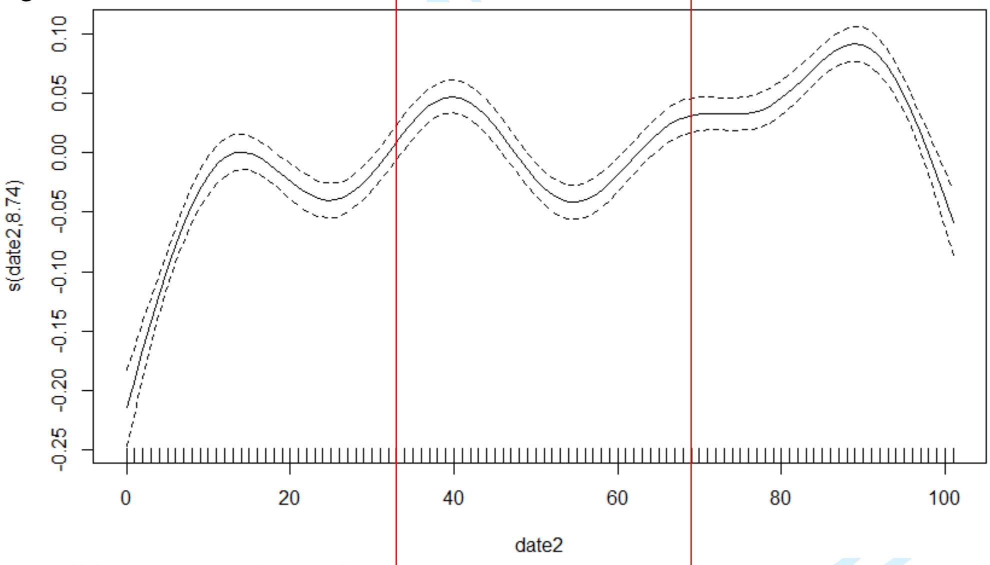

Condition counterfactual

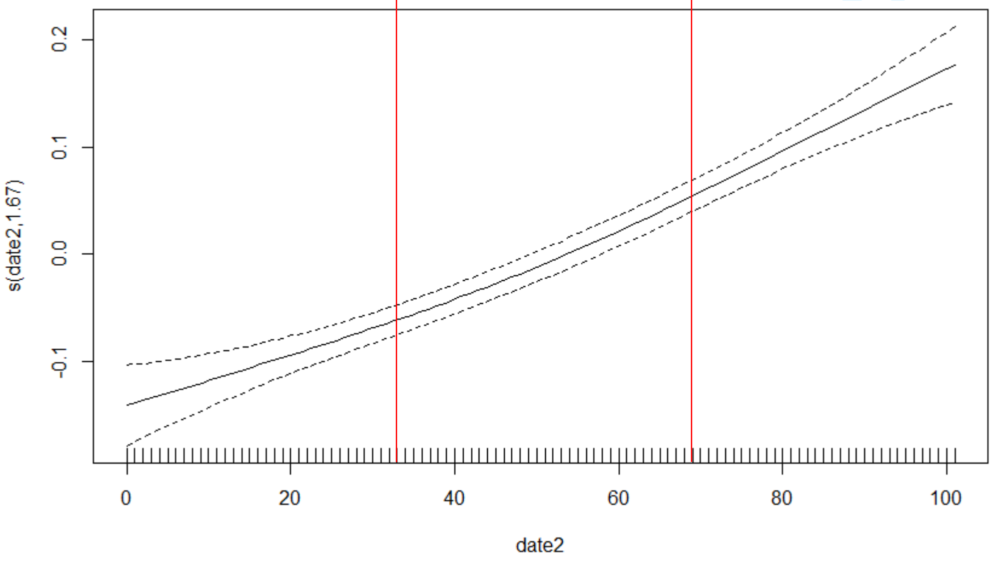

Supplemental file 3 Sensitivity analysis

Defined Daily Doses (DDDs)

Intervention

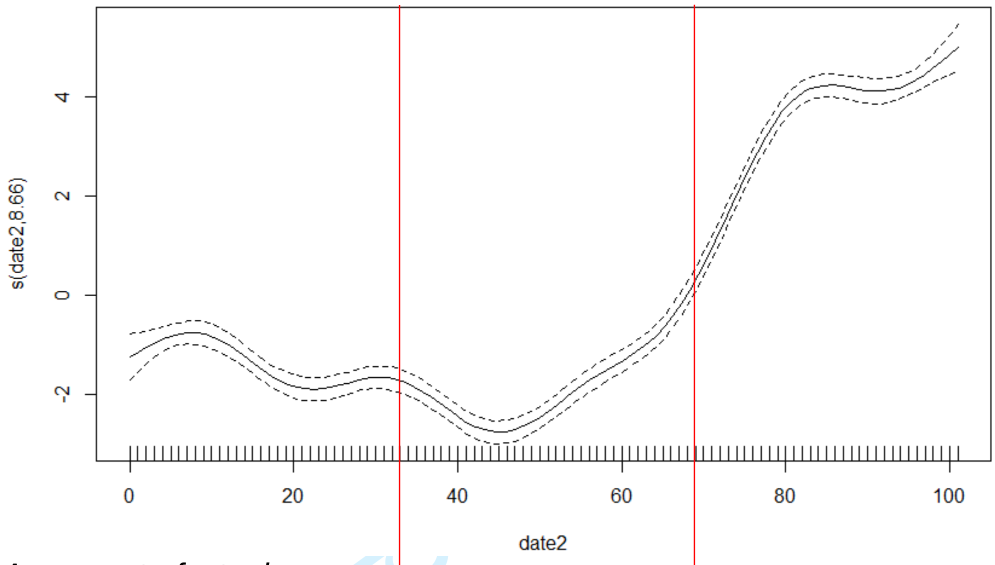

Age counterfactual

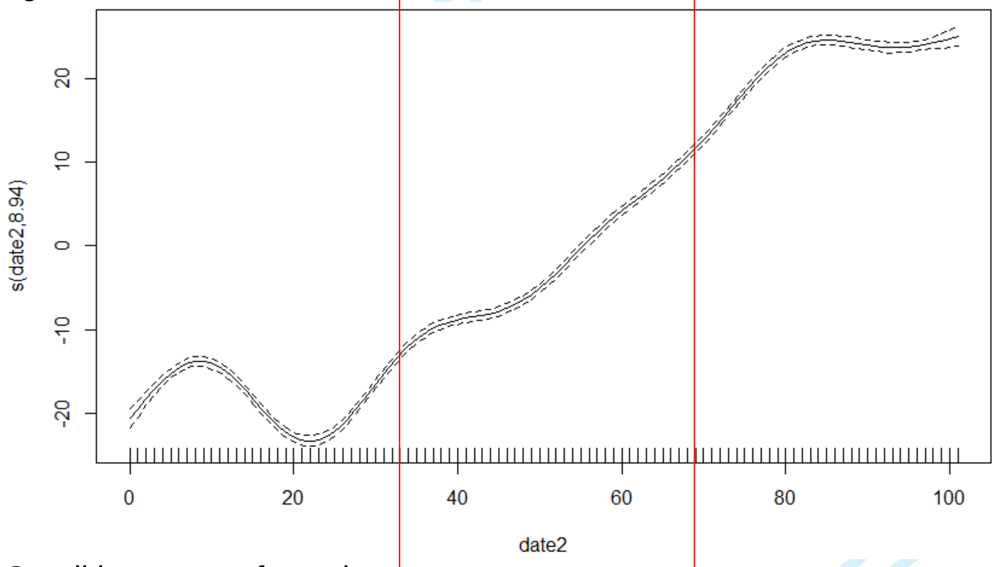

Condition counterfactual

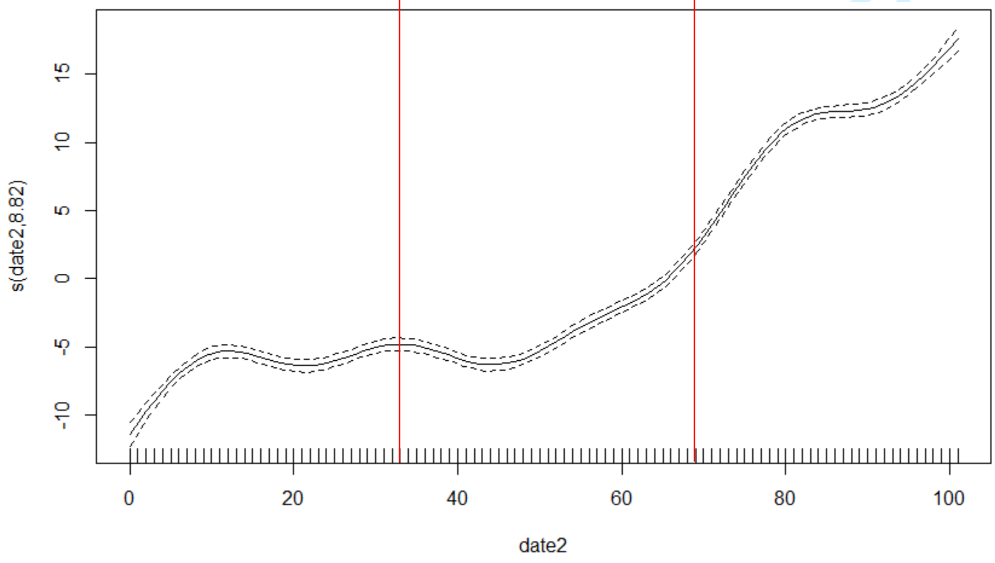

Supplemental file 3 Sensitivity analysis

Cost

Intervention

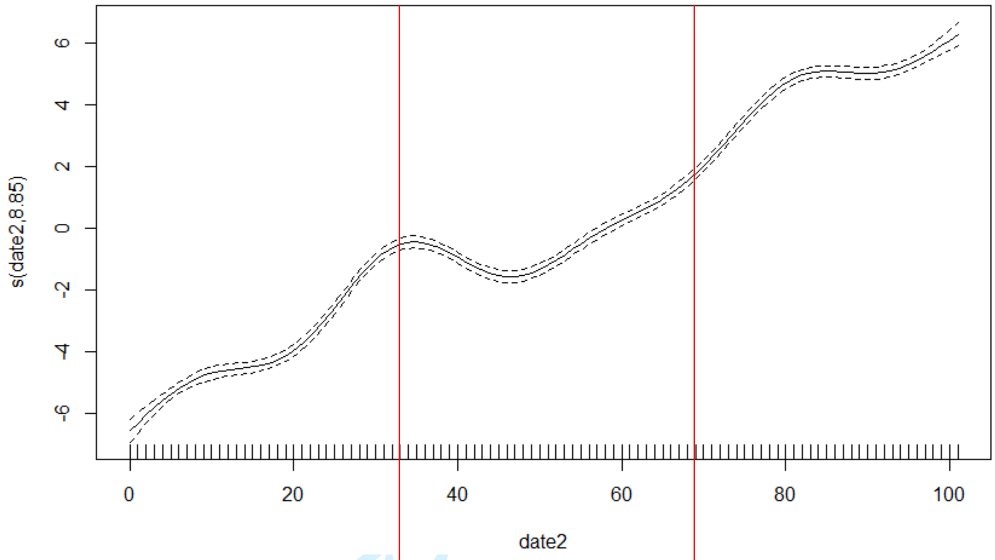

Age counterfactual

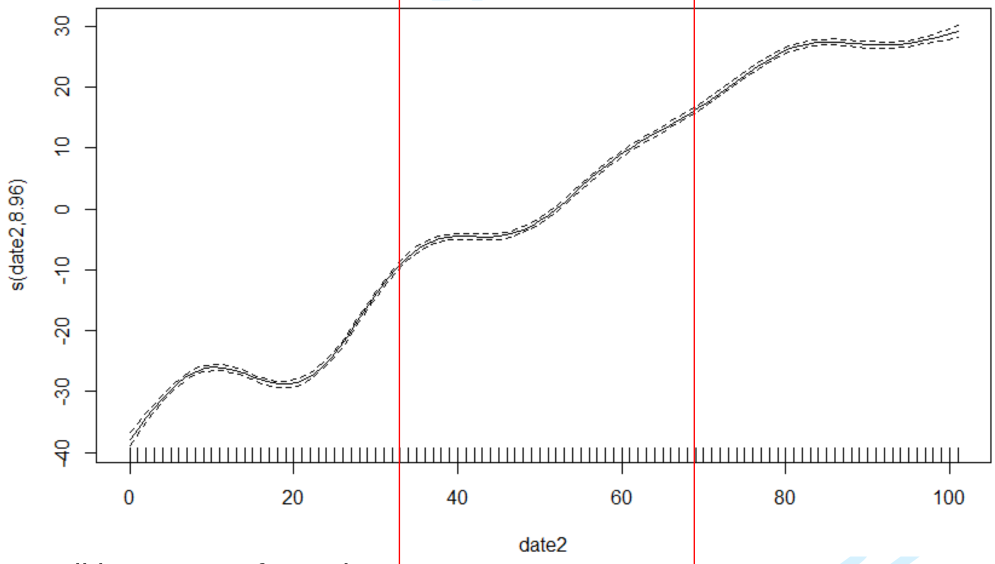

Condition counterfactual

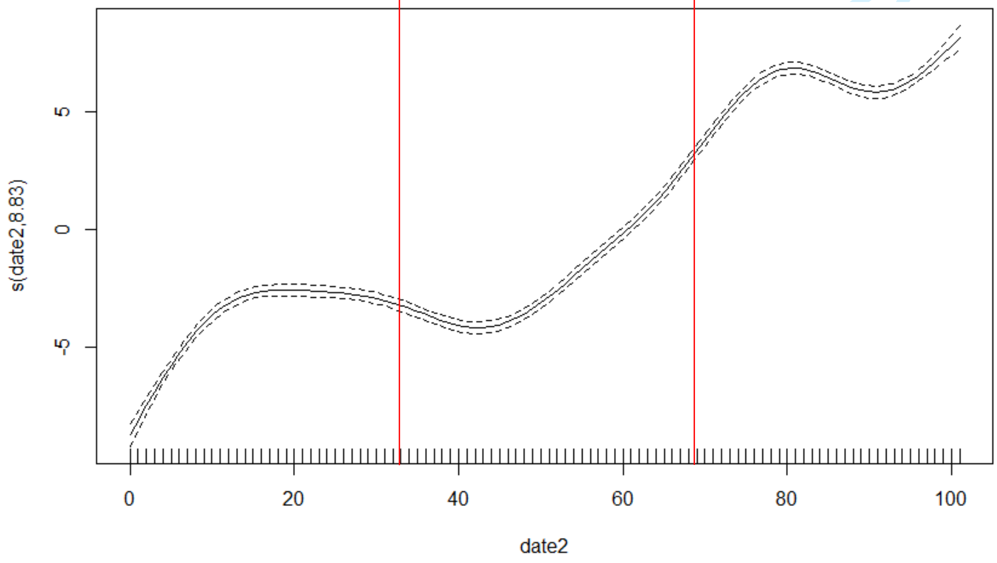

STROBE 2007 (v4) Statement—Checklist of items that should be included in reports of *cohort studies*

Section/Topic	Item #	Recommendation	Reported on page #
**Title and abstract**	1	(*a*) Indicate the study's design with a commonly used term in the title or the abstract	1
		(*b*) Provide in the abstract an informative and balanced summary of what was done and what was found	2
**Introduction**			
Background/rationale	2	Explain the scientific background and rationale for the investigation being reported	5-6
Objectives	3	State specific objectives, including any prespecified hypotheses	6
**Methods**			
Study design	4	Present key elements of study design early in the paper	6-10
Setting	5	Describe the setting, locations, and relevant dates, including periods of recruitment, exposure, follow-up, and data collection	6-7
Participants	6	(*a*) Give the eligibility criteria, and the sources and methods of selection of participants. Describe methods of follow-up	6-9
		(*b*) For matched studies, give matching criteria and number of exposed and unexposed	N/A
Variables	7	Clearly define all outcomes, exposures, predictors, potential confounders, and effect modifiers. Give diagnostic criteria, if applicable	6-10
Data sources/ measurement	8*	For each variable of interest, give sources of data and details of methods of assessment (measurement). Describe comparability of assessment methods if there is more than one group	6-10
Bias	9	Describe any efforts to address potential sources of bias	6-17
Study size	10	Explain how the study size was arrived at	10-11
Quantitative variables	11	Explain how quantitative variables were handled in the analyses. If applicable, describe which groupings were chosen and why	6-10
Statistical methods	12	(*a*) Describe all statistical methods, including those used to control for confounding	9-10
		(*b*) Describe any methods used to examine subgroups and interactions	9-10
		(*c*) Explain how missing data were addressed	NA
		(*d*) If applicable, explain how loss to follow-up was addressed	NA
		(*e*) Describe any sensitivity analyses	17
**Results**			

For peer review only

Participants	13*	(a) Report numbers of individuals at each stage of study—eg numbers potentially eligible, examined for eligibility, confirmed eligible, included in the study, completing follow-up, and analysed	10-11
		(b) Give reasons for non-participation at each stage	NA
		(c) Consider use of a flow diagram	NA
Descriptive data	14*	(a) Give characteristics of study participants (eg demographic, clinical, social) and information on exposures and potential confounders	Table 2 page 11
		(b) Indicate number of participants with missing data for each variable of interest	NA – complete case analysis
		(c) Summarise follow-up time (eg, average and total amount)	6-7
Outcome data	15*	Report numbers of outcome events or summary measures over time	Tables 3 and 4
Main results	16	(*a*) Give unadjusted estimates and, if applicable, confounder-adjusted estimates and their precision (eg, 95% confidence interval). Make clear which confounders were adjusted for and why they were included	Tables 3 and 4
		(*b*) Report category boundaries when continuous variables were categorized	NA
		(*c*) If relevant, consider translating estimates of relative risk into absolute risk for a meaningful time period	NA
Other analyses	17	Report other analyses done—eg analyses of subgroups and interactions, and sensitivity analyses	14-17
**Discussion**			
Key results	18	Summarise key results with reference to study objectives	17-18
**Limitations**			
Interpretation	20	Give a cautious overall interpretation of results considering objectives, limitations, multiplicity of analyses, results from similar studies, and other relevant evidence	17-20
Generalisability	21	Discuss the generalisability (external validity) of the study results	17-20
**Other information**			
Funding	22	Give the source of funding and the role of the funders for the present study and, if applicable, for the original study on which the present article is based	3

*Give information separately for cases and controls in case-control studies and, if applicable, for exposed and unexposed groups in cohort and cross-sectional studies.

Note: An Explanation and Elaboration article discusses each checklist item and gives methodological background and published examples of transparent reporting. The STROBE checklist is best used in conjunction with this article (freely available on the Web sites of PLoS Medicine at http://www.plosmedicine.org/, Annals of Internal Medicine at http://www.annals.org/, and Epidemiology at http://www.epidem.com/). Information on the STROBE Initiative is available at www.strobe-statement.org.

For peer review only

BMJ Open

Interrupted time series evaluation of the impact of abolishing prescription fees in Scotland on hospital admissions and prescribed medicines

Journal:	*BMJ Open*
Manuscript ID	bmjopen-2017-021318.R2
Article Type:	Research
Date Submitted by the Author:	03-Jul-2018
Complete List of Authors:	Williams, Andrew James; University of Exeter, European Centre for Environment and Human Health<br>Henley, W; Institute of Health Research, University of Exeter Medical School, College House, St Luke's Campus, Exeter, Devon, UK, EX1 2LU<br>Frank, John; University of Edinburgh, The Usher Institute of Population Health Sciences and Informatics, College of Medicine and Veterinary Medicine,
**Primary Subject Heading**:	Health policy
Secondary Subject Heading:	General practice / Family practice, Health economics, Health services research, Public health, Pharmacology and therapeutics
Keywords:	administrative data, natural experiment, quasi-experimental, interrupted time series, Fees, Pharmaceutical, co-payment

SCHOLARONE™
Manuscripts

Interrupted time series evaluation of the impact of abolishing prescription fees in Scotland on hospital admissions and prescribed medicines

Andrew James Williams

European Centre for Environment and Human Health, University of Exeter Medical School, Truro, United Kingdom

William Henley

Institute of Health Research, University of Exeter Medical School, Exeter, United Kingdom

John Frank (corresponding author)

Scottish Collaboration for Public Health Research and Policy, University of Edinburgh, 20 West Richmond Street, Edinburgh EH8 9DX United Kingdom

john.frank@ed.ac.uk, +44(0)131 6 511593

Word count (excluding title page, abstract, references, figures and tables): 4,316

For peer review only

ABSTRACT

Objectives: To identify whether the abolition of prescription fees in Scotland resulted in:

a) increase in the number (cost to NHS) of medicines prescribed for which there had been a fee (inhaled corticosteroids);
b) reduction in hospital admissions for conditions related to those medications for which there had been a fee (asthma or Chronic Obstructive Pulmonary Disease (COPD)) –

when both are compared to prescribed medicines and admissions for a condition (diabetes mellitus) for which prescriptions were historically free.

Design: Natural experimental retrospective General Practice level interrupted time series (ITS) analysis using administrative data

Setting: General Practices, Scotland, United Kingdom

Participants: 732 (73.6%) General Practices across Scotland with valid dispensed medicines and hospital admissions data during the study period (July 2005 – December 2013)

Intervention: Reduction in fees per dispensed item from April 2008 leading to the abolition of the fee in April 2011, resulting in universal free prescriptions

Primary and secondary outcome: Hospital admissions recorded in the Scottish Morbidity Record – 01 Inpatient (SMR01) and dispensed medicines recorded in the Prescriptions Information System (PIS).

Results: The ITS analysis identified marked step reductions in adult (19-59 years) admissions related to asthma or COPD (the intervention group), compared to older or young people with the same conditions or adults with diabetes mellitus (the counterfactual groups). The prescriptions findings were less coherent and subsequent sensitivity analyses found that both the admissions and prescriptions data were highly variable above the annual or seasonal level, limiting the ability to interpret the findings of the ITS analysis.

Conclusions: This study did not find sufficient evidence that universal free prescriptions was a demonstrably effective or ineffective policy, in terms of reducing hospital admissions or reducing socioeconomic inequality in hospital admissions, in the context

For peer review only

For peer review only - http://bmjopen.bmj.com/site/about/guidelines.xhtml

of a universal, publicly administered medical care system, the National Health Service of Scotland.

Key words: Fees, Pharmaceutical, interrupted time series, administrative data, natural experiment, quasi-experimental, co-payment

ARTICLE SUMMARY

Strengths and limitations of this study

- Administrative data permitted the undertaking of a large long-term evaluation of a policy, which otherwise would have been very costly and indeed, perhaps infeasible.
- Data were available for over 70% of the practices in Scotland, which could be used to classify those who did and did not experience a change in prescription fees.
- The data were only available at the General Practice not the individual level.
- The time series for both admissions and prescriptions were highly variable, preventing the drawing of specific conclusions from the evaluation.

FUNDING STATEMENT

This work was funded by the Farr Institute @ Scotland, which is funded by the following consortium: Arthritis Research UK, the British Heart Foundation, Cancer Research UK, the Economic and Social Research Council, the Engineering and Physical Sciences Research Council, the Medical Research Council, the National Institute of Health Research, the National Institute for Social Care and Health Research (Welsh Assembly Government), the Chief Scientist Office (Scottish Government Health Directorates), (MRC Grant No: MR/K007017/1). JF and AJW worked for the Scottish Collaboration for Public Health Research and Policy which is funded by the SCPHRP core grant from the Medical Research Council (Grant Number MR/K023209/1) and the Chief Scientist Office of Scotland. WH received support from the National Institute for Health Research (NIHR) Collaboration for Leadership in Applied Health Research and Care (CLAHRC)

For peer review only - http://bmjopen.bmj.com/site/about/guidelines.xhtml

for the South West Peninsula. The funders played no role in the conceptualisation or realisation of the research and no role in the decision to submit it for publication.

COMPETING INTERESTS

The authors declare that they have no competing interests.

For peer review only

For peer review only - http://bmjopen.bmj.com/site/about/guidelines.xhtml

INTRODUCTION

When the National Health Service was founded in the United Kingdom in 1948, all the services provided were free. However, as early as 1950 expenditure was higher than expected, so a charge (1 shilling) for prescribed items (including medicines, medical devices, dressings, borderline substances etc.) was introduced in 1952.[1] This prescription fee has increased over time and now in England the charge is £8.80 per item.[2] In 1968 a list of medical conditions for which no prescription fee would be charged was introduced; these were easily recognisable, lifelong, life threatening conditions that required regular, prescribed medication.[1] Over time further exemptions were identified and the most recent list of exemption criteria is very extensive indeed (Supplemental file 1) [2]. Now, the majority of prescriptions are exempt, meaning that in 2004/05 less than 5% of the cost of pharmaceutical services in Scotland was covered by the prescription fee.[1]

The prescription fee has been described as a 'tax on ill health' (Nicola Sturgeon, then Scottish Health Secretary).[3,4] Although, the exemption criteria (Supplemental file 1) contain many chronic diseases, important conditions such as asthma and degenerative neurological conditions are missing. These omissions have led many to call for the abolition of the fee.[1,5,6] In 2007 Wales was the first country in the United Kingdom (UK) to abolish prescription fees, followed by Northern Ireland in 2010 and Scotland in 2011; a fee is still charged in England.[3] Figure 1(a) shows prescription fees in England and Scotland during the time period examined in this study. Patients requiring multiple prescriptions a month could purchase a short-term (3-4 month) or long-term (12 month) Pre-Payment Certificate (PPC) which provided a discount. Alongside the prescription fee reduction, Scotland made a marked reduction in PPC cost (Figure 1b).

Although, the prescription fee only covers a small proportion of the costs of prescription services, abolishing the fee means that the cost needs to be met elsewhere. One of the justifications for abolishing prescription fees has been that this will lead to improved adherence to medication and subsequently reduced medical costs, through less need for expensive secondary medical care. Campbell, et al.[7] found that for asthmatic patients in the United States of America (USA), an increase as small as $5

For peer review only - http://bmjopen.bmj.com/site/about/guidelines.xhtml

in pharmacy co-payments resulted in reduced adherence and increased outpatient visits. Kulik, et al.[8] undertook a randomised comparison in the USA of the health impact of providing patients with recent myocardial infarction with free prescriptions for all medications, versus only a small selection of cardiovascular medications. It was found that full prescription cost coverage resulted in significantly improved adherence and trends towards reductions in rates of major vascular events or revascularisation [8]. In Canada, where all prescriptions are free for those over 65 years of age, health inequalities by socioeconomic position among those with diabetes are much reduced in this age group compared with those under 65 [9]. Thus far, the only published evaluations of the policy change across the UK have come from Wales and only included prescriptions data and anecdotal reports around other outcomes.[10-13] Hence, the intention of this study was to identify whether the abolition of prescription fees in Scotland resulted in (see Supplemental file 2 for the study protocol):

a) increase in the number (cost to NHS) of medicines prescribed for which there had been a fee (inhaled corticosteroids);
b) Reduction in hospital admissions for conditions related to those medications for which there had been a fee (asthma and Chronic Obstructive Pulmonary Disease (COPD)).

The comparisons for both prescriptions and admissions were conditions for which prescriptions were historically free (diabetes mellitus, or the young or elderly). Subsequent research questions were:

c) Was there any change in hospital admissions in the practices which also saw a change in dispensing?
d) How did the effects of fee abolition differ across the socioeconomic spectrum?

METHODS

Retrospective policy analysis As the abolition of prescription fees had already taken place and was not implemented experimentally, it was necessary to use a natural experimental approach: interrupted time series. This required historical data on policy exposure and outcomes.

For peer review only - http://bmjopen.bmj.com/site/about/guidelines.xhtml

Data Two large administrative datasets are available in Scotland which made this study feasible; the Prescribing Information System (PIS) and the Scottish Morbidity Record – 01 Inpatient (SMR01). PIS is the database of all community prescribed and dispensed medicines across Scotland. SMR01 is the database of all hospital inpatient admissions across Scotland. Using the unique identifier, the Community Health Index (CHI), it is possible to link individual health records across health databases in Scotland. However, CHI has only been recorded in the PIS since August 2009, and therefore this study was restricted to General Practice-level data. The time period examined in this study was July 2005 – December 2013 with fee reductions beginning in April 2008 and abolished from April 2011.

Exposure During study development, a group of clinicians and pharmacists were consulted to inform the study design, in particular the definition of exposure to the policy. Starting from the prescription fee exemption criteria (Supplemental file 1), groups for whom prescriptions remained free (counterfactual) and became free (intervention) needed defining. The groups needed to be identifiable in the PIS and SMR01 and relate to relatively common conditions for which non-adherence to medication would likely result in the need for hospital admission. Subsequently, the subset of pharmaceutical-sensitive ambulatory care-sensitive conditions was used.[14] No data on patient financial situation is recorded in SMR01, which meant that intervention and counterfactual groups could not be defined in terms of the finance related prescription fee exemption criteria. Research question (d) was included so that some examination of the impact of financial situation was undertaken. Asthma and COPD, common adulthood conditions with condition-specific mediations for which the prescription fee was charged, were identified as the most appropriate intervention conditions. Diabetes mellitus, medication for which had been exempt from the prescription fee for many years, constituting the counterfactual condition. In addition to a condition-based counterfactual group, an age-based counterfactual group was used. The criteria for the intervention and two counterfactual groups are given in Table 1. The need to use the exemption criteria to classify intervention and counterfactuals meant that dispensing data from PIS was used, as exemption is only recorded when the prescription is

For peer review only - http://bmjopen.bmj.com/site/about/guidelines.xhtml

dispensed. This prevented the study's consideration of primary non-adherence, when the patient does not have the prescription dispensed.

For peer review only

For peer review only - http://bmjopen.bmj.com/site/about/guidelines.xhtml

Table 1 – Intervention and counterfactual group definitions

	Prescribing Information System (PIS)	Scottish Morbidity Record – 01 Inpatient (SMR01)
**Intervention**	BNF: 3.2 Inhaled steroids<br>Fee: paid<br>Age group: >18 & <60 years	ICD-10: J20, J40X, J41, J42, J43, J44, J45, J46 or J47<br>Age group: >18 & <60 years
**Condition-based counterfactual**	BNF: 6.1.1 or 6.1.2<br>Fee: exempt E - Medical exemption<br>Age group: >18 & <60 years	ICD-10: E10, E11, E12, E13 or E14<br>Age group: >18 & <60 years
**Age-based counterfactual**	BNF: 3.2 Inhaled steroids<br>Fee: exempt A - <16 years, B - ≥16 - ≤18 years and in full time education or C - ≥60 years<br>Age group: <16 years, ≥16 - ≤18 years or ≥60 years	ICD-10: J20, J40X, J41, J42, J43, J44, J45, J46 or J47<br>Age group: <16 years, ≥16 - ≤18 years or ≥60 years

BNF: British National Formulary, ICD-10: International Classification of Disease 10th version

Using the exemption criteria to define the intervention and counterfactual groups within the PIS presented a challenge, as the exemption criteria did not need to be recorded after the fee abolition. Fortuitously, exemption criteria continued to be recorded in many instances. The proportion of prescriptions against each exemption criteria was compared before and after the abolition, to check if reporting had changed. Apart from those receiving free prescriptions due to being aged 60 years or over, reporting of exemption criteria fell markedly, particularly reporting PPCs or full payment. Consequently, given that Scottish Public Health Observatory data [15-17] did not show any marked changes in the prevalence of asthma, COPD or diabetes during the study period, the proportion eligible for each exemption prior to the policy change was applied to the PIS data after the policy change. This was not an issue for the SMR-01 data, as the variables used to define the groups (Table 1) were not affected by the policy change. The datasets were prepared, cleaned and merged in Stata [18] with the top 1% of prescription data being deleted as these very large values appeared to be errors (most likely due to keying errors during manual data entry). To make the outcomes comparable between groups, the primary metric of prescriptions used was Defined Daily Doses (DDDs). The prescription cost was also examined in terms of the gross ingredient cost before any discounts (dispensing costs are met by an overall service remuneration model within the NHS in Scotland rather than an additional cost per item).

For peer review only - http://bmjopen.bmj.com/site/about/guidelines.xhtml

Number and quantity of prescriptions were provided in the data set but were found not to be comparable across study groups, so were not analysed. Data on the following characteristics of each General Practice was also collected: Health Board, number of General Practitioners, list size (by age group), whether the practice had its own dispensary, the practice NHS contract type, the quintile of the Scottish Index of Multiple Deprivation (SIMD) [19] of the practice postcode, the urban rural classification of the practice postcode, the number of patients registered at the practice living in a postcode defined as the 15% most deprived in Scotland, and the number of registered patients from each SIMD quintile and urban rural classification.

Interrupted time series analysis The dataset was declared a time series dataset with monthly time periods and any missing time points within practices were filled with zero. As these data are related to instances when someone of a specific age with a specific condition from a specific general practice had been admitted to hospital or dispensed a medicine; missing data represent months when no prescriptions of that type or admissions for that condition occurred from that practice. Admissions included small and zero counts (e.g. number of adult (19-59 years) asthma admissions per practice per month) so these data were analysed using mixed effects Poisson models, offset by practice size to account for variations in practice size. DDDs and costs were transformed into DDDs or cost per 100 patients per practice to account for differences in practice size. The resulting measures were sufficiently normally distributed to be analysed using mixed-effects linear models. Interrupted time series operators were derived so that any model would fit a new intercept and slope at the time of the intervention, which could be delayed using lags. Calendar month was fitted as a categorical variable to account for seasonality. Finally, the data were treated as hierarchical with measurement month (i) nested within General Practice (j) and the linear model for costs and DDDs was given by:

$$y_{ij} = \beta_{0ij} + \beta_1 date_{ij} + \beta_2 jan_{ij} + \beta_3 feb_{ij} + \beta_4 mar_{ij} + \beta_5 may_{ij} + \beta_6 jun_{ij} + \beta_7 jul_{ij} + \beta_8 aug_{ij} + \beta_9 sep_{ij} + \beta_{10} oct_{ij} + \beta_{11} nov_{ij} + \beta_{12} dec_{ij} + \beta_{13} reduction\ period_{ij} + \beta_{14}\ time\ since\ reductions\ began_{ij} + \beta_{15} abolition\ period_{ij} + \beta_{16}\ time\ since\ abolition\ began_{ij} + e_j + \mu_{ij}$$

For peer review only - http://bmjopen.bmj.com/site/about/guidelines.xhtml

Where y_{ij} is the outcome for practice *j* at time *i* (in months), $date_{ij}$ is the count of months from the beginning of the study period (July 2005), e_j is the difference between the intercept for the estimated overall trend and the intercept for the estimated trend for practice *j*, and μ_{ij} is the difference between the observed outcome value for time *i* at practice *j* and the predicted value, and e_j and μ_{ij} are random effects. Jan_{ij} to dec_{ij} are dummy variables for each month from January to December, with April being the reference category as this was the month in which the policy changes were implemented. $Reduction_{ij}$ *period* and $abolition_{ij}$ *period* are dummy variables for the periods April 2008 to March 2011, and April 2011 to December 2013 (the end of the study period) respectively. *Time since reductions* $began_{ij}$ and *time since abolition* $began_{ij}$ are counts of months from the beginning to end of the reduction and abolition periods respectively. The meaning of the beta coefficients were: β_0 mean outcome in July 2005 (intercept), β_1 secular trend for time from July 2005, $\beta_{2\text{-}12}$ mean effect for each month excluding April (seasonality), β_{13} change in intercept when the prescription fee was being reduced (reduction step change), β_{14} change in slope (from April 2008 to March 2011) when the prescription fee was being reduced (reduction change in slope), β_{15} change in intercept when the prescription fee was abolished (abolition step change) and β_{16} change in slope (from April 2011) when the prescription fee was abolished (abolition change in slope). The Poisson model for admissions was defined similarly with the logarithm of the admission rate expressed as a linear function of the fixed and random effects, and with the addition of an offset term for the practice size.

Admissions data were analysed with 0, 1, 2 and 3 month lags on the intervention effect. To address research question (c), it was planned that practices where changes in prescriptions and admissions consistent with the hypothesised effect of the policy change be identified and compared as a secondary analysis. Finally, to address research question (d), practices whose population modal quintile of the SIMD 2012 [19] remained the same throughout the study period (constant sample size) were included in a repetition of the primary analysis stratified by quintile of SIMD. During model development the hierarchical linear time series models were specified with first or second order autoregression or moving averages. The results did not differ substantially

For peer review only

between specifications and therefore all the results presented were specified with second order moving average.

Patient and public involvement

In the early phases of development (January 2015), this project was presented to the Public Panel of the Scottish Farr Institute (http://www.farrinstitute.org/farr-scotland). The ensuing discussion demonstrated the interest of the panel members in the project, which is critical for secondary data projects such as this, and helped shape the analysis and interpretation of the findings. In particular, the panel members brought to the researchers' attention the issue of stock-piling of prescribed medicines following the fee abolition. As a secondary data analysis there was limited potential for public engagement during the conduct of the study, but the results will be disseminated to the Public Panel and more widely through the Farr Institute website.

RESULTS

Across the eight-year study period, the number of practices and their characteristics changed. The characteristics of the practices with prescriptions data at the end of the study period (December 2013) are presented in Table 2. Data on prescribed medicines were available for 732 (73.6%) of the 994 General Practices across Scotland at the end of 2013, while admissions data was available for 754 (75.9%) practices. The majority of practices had 5 or fewer general practitioners, fewer than 10,000 patients, and were in urban areas. Less than 10% of practices had their own dispensary.

For peer review only - http://bmjopen.bmj.com/site/about/guidelines.xhtml

Table 2 – Practice characteristics in December 2013 (n=732)

**Characteristic**	**Summary statistic**
Number of GPs	
≤5 general practitioners	447 (61.1%)
>5–≤10 general practitioners	246 (33.6%)
>10 general practitioners	39 (5.3%)
Practice size	
≤5,000 patients	345 (47.1%)
>5,000–≤10,000 patients	305 (41.7%)
>10,000 patients	82 (11.2%)
Dispensing practice	67 (9.2%)
Health Board	
NHS Ayrshire & Arran	37 (5.1%)
NHS Borders	20 (2.7%)
NHS Dumfries & Galloway	26 (3.6%)
NHS Fife	40 (5.5%)
NHS Forth Valley	44 (6.0%)
NHS Grampian	37 (5.1%)
NHS Greater Glasgow & Clyde	215 (29.4%)
NHS Highland	72 (9.8%)
NHS Lanarkshire	60 (8.2%)
NHS Lothian	107 (14.6%)
NHS Orkney	3 (0.4%)
NHS Shetland	4 (0.5%)
NHS Tayside	60 (8.2%)
NHS Western Isles	7 (1.0%)
Modal SIMD quintile of patients	
Quintile 1 (most deprived)	224 (30.6%)
Quintile 2	127 (17.3%)
Quintile 3	155 (21.2%)
Quintile 4	108 (14.8%)
Quintile 5 (least deprived)	118 (16.1%)
Modal Location of patients	
Large Urban Areas	302 (41.3%)
Other Urban Areas	192 (26.2%)
Accessible Small Towns	64 (8.7%)
Remote Small Towns	17 (2.3%)
Very Remote Small Towns	9 (1.2%)
Accessible Rural	61 (8.3%)
Remote Rural	32 (4.4%)
Very Remote Rural	55 (7.5%)

GP; general practitioner, NHS; National Health Service, SIMD; Scottish Index of Multiple Deprivation [19]

For peer review only

For peer review only - http://bmjopen.bmj.com/site/about/guidelines.xhtml

The intercept (constant) and coefficients of the interrupted time series operators for the Poisson and linear mixed effects models of admissions, DDDs and cost are presented in Table 3. Throughout the results, p-values have not been reported and the focus was on estimated effect sizes, as the size of the dataset resulted in many statistical but not meaningful significances. The intercept estimates demonstrate that there were pre-existing differences in the level of admissions and prescriptions across the intervention and counterfactual groups at the start of the study period (July 2005). The highest rate of admissions, and number (DDDs) and cost of prescribed medicines were observed in the age-based counterfactual group, while the fewest admissions and prescribed medicines were seen among the intervention group. This demonstrates that asthma and COPD are, as expected, most problematic to the young and old. Despite these differences in quantity, the background secular time trend in admissions before the abolition of prescription fees is consistent across the groups. The coefficients of the interrupted time series operators are also mostly consistent across groups and increasing degrees of time lag. A notable exception was a significant step reduction in admissions in the intervention group, related to the abolition of prescription fees, although the underlying time trend did not significantly change. The results regarding prescribed medicines are much less clear, with more marked temporal variability across the study period. However, the largest changes appear in the counterfactual groups, indicating some phenomenon other than the intended effect of the policy change, which we were unable to identify.

For peer review only - http://bmjopen.bmj.com/site/about/guidelines.xhtml

Table 3 – Results of interrupted time series analysis of the impact of reducing and abolishing prescription fees in Scotland on hospital admissions (incidence rate ratios) and prescribed medicines (defined daily doses and cost)

	Intercept		Monthly change		Reduction step change		Reduction change in slope		Abolition step change		Abolition char	
**Admissions**	IR	95% CI	IRR	95% CI	IRR	95% CI	IRR	95% CI	IRR	95% CI	IRR	95%
Intervention	$1.01e^{-2}$	$9.46e^{-3}$ to $1.09e^{-2}$	1.00	1.00 to 1.01	1.03	0.98 to 1.08	0.99	0.99 to 0.99	0.81	0.72 to 0.90	1.00	0.9
Age-c'fact	$6.65e^{-2}$	$6.36e^{-2}$ to $6.95e^{-2}$	1.00	1.00 to 1.00	1.03	1.01 to 1.06	1.00	0.99 to 1.00	0.95	0.90 to 1.00	1.00	0.9
Condition-c'fact	$4.80e^{-3}$	$4.39e^{-3}$ to $5.24e^{-3}$	1.00	1.00 to 1.01	0.95	0.88 to 1.03	1.00	1.00 to 1.01	1.01	0.86 to 1.20	1.00	1.0
**Admissions 1 month lag**	IR	95% CI	IRR	95% CI	IRR	95% CI	IRR	95% CI	IRR	95% CI	IRR	95%
Intervention	$1.02e^{-2}$	$9.49e^{-3}$ to $1.09e^{-2}$	1.01	1.00 to 1.01	1.01	0.96 to 1.06	0.99	0.99 to 0.99	0.78	0.71 to 0.87	1.00	0.9
Age-c'fact	$6.74e^{-2}$	$6.45e^{-2}$ to $7.05e^{-2}$	1.00	1.00 to 1.00	1.03	1.01 to 1.06	1.00	0.99 to 1.00	0.95	0.90 to 1.00	1.00	0.9
Condition-c'fact	$4.83e^{-3}$	$4.41e^{-3}$ to $5.28e^{-3}$	1.00	1.00 to 1.00	0.98	0.91 to 1.06	1.00	1.00 to 1.00	1.07	0.91 to 1.26	1.00	1.0
**Admissions 2 month lag**	IR	95% CI	IRR	95% CI	IRR	95% CI	IRR	95% CI	IRR	95% CI	IRR	95%
Intervention	$1.02e^{-2}$	$9.51e^{-3}$ to $1.09e^{-2}$	1.00	1.00 to 1.01	1.01	0.96 to 1.06	0.99	0.99 to 0.99	0.79	0.71 to 0.87	1.00	0.9
Age-c'fact	$6.77e^{-2}$	$6.47e^{-2}$ to $7.07e^{-2}$	1.00	1.00 to 1.00	1.04	1.02 to 1.07	1.00	0.99 to 1.00	0.97	0.92 to 1.02	1.00	1.0
Condition-c'fact	$4.85e^{-3}$	$4.44e^{-3}$ to $5.30e^{-3}$	1.00	1.00 to 1.00	0.99	0.92 to 1.07	1.00	1.00 to 1.01	1.10	0.93 to 1.28	1.00	1.0
**Admissions 3 month lag**	IR	95% CI	IRR	95% CI	IRR	95% CI	IRR	95% CI	IRR	95% CI	IRR	95%
Intervention	$1.02e^{-2}$	$9.49e^{-2}$ to $1.09e^{-2}$	1.01	1.00 to 1.01	0.99	0.94 to 1.04	0.99	0.99 to 0.99	0.78	0.70 to 0.86	1.00	0.9
Age-c'fact	$6.73e^{-2}$	$6.44e^{-2}$ to $7.04e^{-2}$	1.00	1.00 to 1.00	1.02	1.00 to 1.05	1.00	0.99 to 1.00	0.94	0.90 to 0.99	1.00	1.0
Condition-c'fat	$4.82e^{-3}$	$4.41e^{-3}$ to $5.26e^{-3}$	1.00	1.00 to 1.00	0.97	0.90 to 1.05	1.00	1.00 to 1.01	1.08	0.92 to 1.26	1.00	1.0
**Defined Daily Doses (DDDs)**	DDDs	95% CI	ΔDDDs	95% CI	ΔDDDs	95% CI	ΔDDDs	95% CI	ΔDDDs	95% CI	ΔDDDs	95%
Intervention	29.65	28.71 to 30.59	-0.04	-0.06 to -0.02	-0.11	-0.61 to 0.39	0.04	0.02 to 0.07	6.03	4.94 to 7.13	0.10	0.0
Age-c'fact	100.88	98.07 to 103.70	-0.05	-0.11 to 0.01	5.06	3.61 to 6.52	0.68	0.59 to 0.77	38.37	34.84 to 41.90	0.34	0.2
Condition-c'fact	55.61	53.70 to 57.53	0.14	0.10 to 0.18	-1.88	-2.90 to -0.85	-0.05	-0.11 to 0.00	6.02	3.73 to 8.32	0.15	0.0
**Cost**	£s	95% CI	Δ£s	95% CI	Δ£s	95% CI	Δ£s	95% CI	Δ£s	95% CI	Δ£s	95%
Intervention	19.36	18.53 to 20.19	0.16	0.15 to 0.18	0.23	-0.19 to 0.66	-0.15	-0.17 to -0.13	-0.96	-1.92 to -0.01	-0.10	-0.12
Age-c'fact	69.92	67.35 to 72.49	0.62	0.56 to 0.68	4.74	3.42 to 6.06	0.03	-0.05 to 0.12	14.24	10.86 to 17.63	-0.36	-0.44
Condition-c'fact	26.35	25.35 to 27.34	0.15	0.13 to 0.17	-3.16	-3.70 to -2.62	-0.01	-0.04 to 0.02	2.00	0.79 to 3.20	-0.11	-0.14

95% CI; 95% confidence interval, c'fact; counterfactual, DDDs; Defined daily doses per 100 patients per practice per month, ΔDDDs; change in DDDs, IR; Incidence rate per 100 patients per practice per month, IRR; incidence rate ratio, £s; gross ingredient cost of medicines before any discount per 100 patients per practice per month, Δ£s; change in £s

For peer review only - http://bmjopen.bmj.com/site/about/guidelines.xhtml

The results of the analysis undertaken to examine whether the impact of the policy differed by socioeconomic position are presented in Table 4. These analyses aimed to assess whether the financial exemptions to the prescription fee (Supplemental file 1) had an impact on the results, as these exemptions could not be accounted for directly in the main analysis. Only the results for the intervention group have been presented, as individual financial position should not have had an impact on the counterfactual groups. The intercepts of the admissions analyses are consistent with known inequalities, with the highest rate of admissions among the most deprived (Q1), and the lowest rate of admissions in the least deprived (Q5). The results for the interrupted time series operators are similar to the main analysis, with the most marked differences being observed in terms of step changes as opposed to changes in the underlying time trend. Although not statistically significant, there seems to be some evidence of reductions in admissions among the most deprived quintiles (Q1 and Q2) in relation to the fee-reduction policy, compared to the least deprived quintiles (Q4 and Q5). However, any effect is not linear across the SIMD quintiles. Again, the results of the analysis of prescriptions were less coherent. The intercepts of the models demonstrate that most paid prescriptions were from practices with mostly Q3 and Q4 patients, who are likely to have had poorer health than Q5 patients, but would be less likely to be eligible for free prescriptions for financial reasons than Q1 and Q2 patients. These quintiles also demonstrate the largest step increases in DDDs when the fees were abolished, which is consistent with this being an effect of the policy.

For peer review only

Table 4 - Results of interrupted time series analysis of the impact of reducing and abolishing prescription fees in Scotland on hospital admissions (incidence rate ratios) and prescribed medicines (defined daily doses and cost) stratified by quintile of the Scottish Index of Multiple Deprivation [19]

	Intercept		Monthly change		Reduction step change		Reduction change in slope		Abolition step change		Abolition cha slope	
**Admissions**	IR	95% CI	IRR	95% CI	IRR	95% CI	IRR	95% CI	IRR	95% CI	IRR	95%
Q1 - most deprived	$1.52e^{-2}$	$1.35e^{-2}$ to $1.71e^{-2}$	1.01	1.01 to 1.01	1.00	0.91 to 1.09	0.99	0.98 to 0.99	0.64	0.52 to 0.78	0.99	0.9
Q2	$1.17e^{-2}$	$9.36e^{-3}$ to $1.47e^{-2}$	1.00	1.00 to 1.01	1.10	0.92 to 1.32	0.99	0.98 to 1.00	0.75	0.50 to 1.12	1.00	0.9
Q3	$9.99e^{-3}$	$7.45e^{-3}$ to $1.34e^{-2}$	1.00	0.99 to 1.01	1.10	0.86 to 1.41	1.00	0.98 to 1.01	1.15	0.68 to 1.94	1.00	0.9
Q4	$6.49e^{-3}$	$4.64e^{-3}$ to $9.07e^{-3}$	0.99	0.98 to 1.00	1.50	1.13 to 1.99	1.00	0.99 to 1.02	1.63	0.89 to 2.99	1.02	1.0
Q5 - least deprived	$6.06e^{-3}$	$4.86e^{-3}$ to $7.54e^{-3}$	1.00	0.99 to 1.00	1.00	0.84 to 1.21	1.00	1.00 to 1.01	1.24	0.85 to 1.82	1.00	0.9
**Admissions 1 month lag**	IR	95% CI	IRR	95% CI	IRR	95% CI	IRR	95% CI	IRR	95% CI	IRR	95%
Q1 - most deprived	$1.50e^{-2}$	$1.33e^{-2}$ to $1.69e^{-2}$	1.01	1.01 to 1.02	0.94	0.86 to 1.03	0.99	0.98 to 0.99	0.60	0.49 to 0.73	0.99	0.9
Q2	$1.20e^{-2}$	$9.58e^{-3}$ to $1.51e^{-2}$	1.00	1.00 to 1.01	1.12	0.93 to 1.34	0.99	0.98 to 1.00	0.79	0.53 to 1.16	1.00	0.9
Q3	$9.97e^{-3}$	$7.40e^{-3}$ to $1.34e^{-2}$	1.00	0.99 to 1.01	1.00	0.78 to 1.27	1.00	0.99 to 1.01	0.99	0.60 to 1.65	1.00	0.9
Q4	$6.81e^{-3}$	$4.85e^{-3}$ to $9.56e^{-3}$	0.99	0.98 to 1.00	1.53	1.16 to 0.02	1.00	0.99 to 1.01	1.66	0.91 to 3.00	1.02	1.0
Q5 - least deprived	$6.12e^{-3}$	$4.90e^{-3}$ to $7.64e^{-3}$	1.00	0.99 to 1.00	1.03	0.86 to 1.23	1.01	1.00 to 1.01	1.27	0.87 to 1.85	1.00	0.9
**Admissions 2 month lag**	IR	95% CI	IRR	95% CI	IRR	95% CI	IRR	95% CI	IRR	95% CI	IRR	95%
Q1 - most deprived	$1.51e^{-2}$	$1.34e^{-2}$ to $1.70e^{-2}$	1.01	1.01 to 1.01	0.94	0.86 to 1.03	0.99	0.98 to 0.99	0.60	0.50 to 0.73	0.99	0.9
Q2	$1.20e^{-2}$	$9.58e^{-3}$ to $1.51e^{-2}$	1.00	1.00 to 1.01	1.11	0.92 to 1.33	0.99	0.98 to 1.00	0.78	0.54 to 1.15	1.00	0.9
Q3	$1.02e^{-2}$	$7.59e^{-3}$ to $1.37e^{-2}$	1.00	0.99 to 1.01	1.07	0.84 to 1.37	1.00	0.99 to 1.01	1.12	0.68 to 1.84	1.00	0.9
Q4	$6.77e^{-3}$	$4.83e^{-3}$ to $9.48e^{-3}$	0.99	0.98 to 1.00	1.57	1.19 to 2.07	1.00	0.98 to 1.01	1.70	0.95 to 3.05	1.02	1.0
Q5 - least deprived	$6.21e^{-3}$	$4.98e^{-3}$ to $7.75e^{-3}$	0.99	0.99 to 1.00	1.09	0.91 to 1.31	1.00	1.00 to 1.01	1.38	0.95 to 1.99	1.00	0.9
**Admissions 3 month lag**	IR	95% CI	IRR	95% CI	IRR	95%CI	IRR	95% CI	IRR	95% CI	IRR	95%
Q1 - most deprived	$1.52e^{-2}$	$1.35e^{-3}$ to $1.71e^{-2}$	1.01	1.01 to 1.01	0.94	0.86 to 1.02	0.99	0.98 to 0.99	0.60	0.50 to 0.73	0.99	0.9
Q2	$1.18e^{-2}$	$9.40e^{-3}$ to $1.48e^{-2}$	1.01	1.00 to 1.01	1.03	0.86 to 1.24	0.99	0.98 to 1.00	0.72	0.50 to 1.05	1.00	0.9
Q3	$1.05e^{-2}$	$7.82e^{-3}$ to $1.41e^{-2}$	1.00	0.99 to 1.00	1.18	0.92 to 1.51	1.00	0.99 to 1.01	1.25	0.76 to 2.05	1.01	0.9
Q4	$6.40e^{-3}$	$4.58e^{-3}$ to $8.96e^{-3}$	0.99	0.98 to 1.00	1.34	1.02 to 1.77	1.00	0.98 to 1.01	1.36	0.77 to 2.39	1.01	1.0
Q5 - least deprived	$6.15e^{-3}$	$4.93e^{-3}$ to $7.67e^{-3}$	1.00	0.99 to 1.00	1.07	0.89 to 1.28	1.00	0.99 to 1.01	1.35	0.94 to 1.94	1.00	0.9
**Defined Daily Doses (DDDs)**	DDDs	95% CI	ΔDDDs	95% CI	ΔDDDs	95% CI	ΔDDDs	95% CI	ΔDDDs	95% CI	ΔDDDs	95%
Q1 - most deprived	21.56	20.13 to 22.99	-0.07	-0.10 to -0.04	0.25	-0.50 to 1.01	0.08	0.04 to 0.12	6.23	4.59 to 7.86	0.14	0.1
Q2	29.47	25.42 to 33.51	0.05	-0.03 to 0.12	-1.83	-3.80 to 0.15	-0.05	-0.15 to 0.05	2.09	-2.19 to 6.37	0.03	-0.0
Q3	35.64	31.29 to 39.98	-0.06	-0.18 to 0.06	1.11	-2.07 to 4.28	-0.03	-0.20 to 0.13	7.32	0.27 to 14.37	0.15	-0.0
Q4	37.89	34.33 to 41.46	-0.05	-0.14 to 0.03	0.12	-2.06 to 2.31	0.06	-0.05 to 0.17	7.62	2.87 to 12.38	0.09	-0.0
Q5 - least deprived	30.20	28.03 to 32.37	0.02	-0.02 to 0.06	-1.15	-2.24 to -0.05	$9.05e^{-4}$	-0.06 to 0.06	4.21	1.83 to 6.60	0.01	-0.0
**Cost**	£s	95% CI	Δ£s	95% CI	Δ£s	95% CI	Δ£s	95% CI	Δ£s	95% CI	Δ£s	95%
Q1 - most deprived	14.25	12.94 to 15.55	0.07	0.05 to 0.09	0.37	-0.24 to 0.98	-0.06	-0.09 to -0.03	1.02	-0.32 to 2.36	-0.02	-0.0
Q2	21.61	17.63 to 25.58	0.21	0.14 to 0.28	-0.88	-2.67 to 0.90	-0.21	-0.30 to -0.12	-2.63	-6.59 to 1.32	-0.18	-0.28
Q3	22.97	19.33 to 26.60	0.20	0.10 to 0.29	0.83	-1.55 to 3.20	-0.25	-0.38 to -0.13	-1.67	-6.95 to 3.62	-0.08	-0.2
Q4	24.84	21.53 to 28.15	0.22	0.14 to 0.30	0.14	-1.92 to 2.21	-0.24	-0.35 to -0.13	-2.63	-7.26 to 2.01	-0.19	-0.30

For peer review only - http://bmjopen.bmj.com/site/about/guidelines.xhtml

Q5 - least deprived	19.78	17.59 to 21.97	0.20	0.17 to 0.24	-0.78	-1.72 to 0.16	-0.18	-0.23 to -0.14	-2.67	-4.76 to -0.58	-0.18	-0.2[illegible]

95% CI; 95% confidence interval; DDDs; Defined daily doses per 100 patients per practice per month, ΔDDDs; change in DDDs, IR; Incidence rate per 100 patients per practice per month, IRR; incidence rate ratio, £s; gross ingredient cost of medicines before any discount per 100 patients per practice per month, Δ£s; change in £s, Q1-5; quintiles of the Scottish Index of Multiple Deprivation from most to least deprived.

For peer review only

For peer review only - http://bmjopen.bmj.com/site/about/guidelines.xhtml

In response to the incoherence of the findings for DDDs and costs and reviewer comments, several sensitivity analyses were undertaken, instead of the analyses originally planned to address the third research question (c). Firstly, using the Generalized Additive Mixed Models (GAMM) package in R [20-24], the models were fitted without the interrupted time series operators but fitting 8 knots evenly distributed across the time series. These models allow change points in the time series at times other than those related to the policy to be revealed and relax the assumption that any time effects were linear. Plots of each of the models undertaken as part of the sensitivity analyses are show in Supplemental file 3, with the dates of reduction and abolition overlaid. The admissions data for the condition-based counterfactual group, show that following the adjustment for seasonality there was a small but linear increase in diabetes-related admissions among adults, which as expected appears not to change in relation to the prescription fee policy changes. However, the admissions data for the intervention and age-based counterfactual groups show marked changes which are neither consistent year to year, nor easily related to the policy changes. The patterns of admissions in both the intervention and age-based counterfactual groups are similar. Both the DDDs and cost of prescribed medicines data for all three groups show similar patterns of increases, with occasional change points. These change points appear to be spaced in time similarly to the reduction and abolition of prescription fees, but around 12 months after the policy changes. The magnitude of the changes in prescribed DDDs and costs are also greater in the counterfactuals than the intervention group, arguing against any causal effect of the intervention.

The second sensitivity analysis undertaken was to adjust for clustering by Health Board as well as General Practice. The results of these models are presented in Supplemental File 4, apart from changes in the intercepts, the estimated coefficients did not change. As a third sensitivity analysis, in light of the results of the GAMM models, the interrupted time series models were run with either a quadratic secular time effect added, or quadratic effects for time since reduction and time since abolition. The results of these models are presented in Supplemental File 5 alongside plots comparing the linear and quadratic effects. The coefficients of the quadratic time effects were minimal and resulted in very few marked changes in the other estimated coefficients. The

For peer review only - http://bmjopen.bmj.com/site/about/guidelines.xhtml

second and third sensitivity analyses demonstrate that the findings of the interrupted time series models were robust to some changes in the clustering of the data and time effects. However, the unusual trends in the time series identified within the first sensitivity analysis and the inconsistency between the findings and the hypothesised effects prevent the drawing of firm conclusions from this study.

DISCUSSION

Using Scottish administrative hospital admissions and prescribed medicines data it was possible to undertake an evaluation of the impact of a major policy change. The evaluation included over 70% of the General Practices in Scotland and analysed changes over an eight-year period. Interrupted time series analysis offered a natural experimental approach for evaluating the impact of policy changes which occurred on specific dates. There was a marked step reduction in adult asthma or COPD related admissions around the time of the abolition of prescription fees (April 2011), compared to the counterfactual groups. This effect was consistent with known health inequalities and the prescription fee policy prior to the change. The data on prescribed medicines were less coherent, with marked changes in the intervention and counterfactual groups. Sensitivity analyses revealed that there was considerable variation in almost all the time series that neither related to the policy change, season or annual changes. It was therefore difficult to draw any firm conclusions about the impact of the policy change.

More rigorous and conclusive results would be generated by an experimental study. However, a prospective study of this size and duration would have been considerably more costly, and potentially impossible to fund. Policy changes like the one evaluated are also rarely introduced experimentally. The prescription fee exemption criteria, rather ironically, made this study possible. One challenge to this study was that the prescription fee exemption criteria did not need to be reported following the policy change, so the assumption was made that the proportions eligible for each exemption criteria would not vary markedly in the relatively short period being studied following the policy change. This assumption was supported by the finding that the proportion receiving free prescribed medicines due to older age remained the same, as these people, possibly through habit, continued to report their exemption. However, the

For peer review only - http://bmjopen.bmj.com/site/about/guidelines.xhtml

variability in the prescriptions time series limited our ability to draw any firm conclusions regarding prescribed medicines. It is a weakness of the study that it was only possible to examine General Practice-level, rather than individual-level data. This prevented the assessment of primary non-adherence and was the result of data not being collected which would permit individual linkage. This linkage is now possible, but not for the years before the policy change. Both SMR-01 and PIS are administrative datasets, collected for billing and reimbursement purposes, and therefore data which are most useful to research are often not collected.

Unlike previous studies into the impact of universal free prescriptions, it has not been possible to identify a clear impact of the policy change in Scotland. The other evaluations of prescription fees have been located in the USA and Canada, where the health systems are quite different to the UK's National Health Service. Kulik, et al.[8] were able to undertake a randomised controlled trial offering post-myocardial infarction patients universal free prescriptions, compared to free prescriptions only for medication for the myocardial infarction. The participants in that study had a well-defined need for prescriptions, whereas in the current study – although the intervention group had a long-term condition (asthma or COPD) – the need for regular medication among adults with these conditions will vary considerably between patients. Like the current study, both Campbell, et al.[7] and Booth, et al.[9] used administrative data permitting larger retrospective studies. The study from Campbell, et al.[7] is very similar to the current study but examined changes in monthly co-payments. They contrasted the impact of increases of greater or less than $5, which is approximately half the cost of the current English prescription fee. However, this was an increase from the average monthly co-payment of around $17, which is considerably larger than the English prescription fee. Booth, et al.[9] examined the whole diabetic population over 65 years of age, who are more likely to be on numerous medications than the adult (19-59 years of age with asthma or COPD) intervention group in this study and therefore would have faced higher prescription costs in a health system where their prescriptions were not free. Finally, the data analysed in this study showed that prior to the policy change less than 13% of prescriptions were paid in full or by pre-payment certificate. Subsequently,

For peer review only - http://bmjopen.bmj.com/site/about/guidelines.xhtml

considering the proportion of that 13% that are for acute conditions, such as bacterial infections, this study may have been looking for a needle in a haystack.

Overall, in this study no marked impact of the abolition of prescription fees in Scotland on prescribed medicines or hospital admissions has been identifiable. However, there were some signs of some impact, specifically a step reduction, within one month of abolition, in adult admissions for asthma or COPD. Although, the availability of administrative data made this study possible, it has also brought the biggest limitations to the study. As the data were primarily collected for administrative and financial purposes, it was understandably not optimal for this study, one of the most significant challenges to the use of big data in research [25-27]. Although the PIS data were particularly messy, even having dropped the top 1% of values, the central recording of community prescriptions in Scotland, unlike England, made this study possible. This study also encountered one of the other challenges to research using big data; 'over sensitivity of statistical hypothesis testing' [25,28]. These are at least two of the topics for further research in the field of big data. Based on this study, it is not possible to conclude whether universal free prescriptions is an effective policy in terms of improving health or reducing inequalities. However, neither does the study provide evidence that the policy is ineffective. There has been some concern that not charging for prescriptions could lead to over-use or stock-piling, which for many medications is risky, as well as being a waste of limited resources. The increases in prescriptions in the counterfactual groups may support this hypothesis. Charging a reasonable fee per prescription may limit this; however, the size of the fee required to cover the administrative costs of the payment system may lead to the fee being high enough to inappropriately limit pharmaceutical use. Rigorous evaluation need to accompany any health policy changes to inform our understanding of the most efficient health systems and services.

AUTHOR CONTRIBUTIONS

AJW contributed to the conceptualisation of the project, undertook all the statistical analysis and drafted the paper. JF contributed to the conceptualisation of the project, supervised the analysis, reviewed and approved the paper. WH supported AJW with the

For peer review only - http://bmjopen.bmj.com/site/about/guidelines.xhtml

statistical analysis, reviewed and approved the paper. We would like to acknowledge the support from the other members of the Farr Institute @ Scotland Natural Experimental Approaches research group; Corri Black (University of Aberdeen), Chris Dibben (University of Edinburgh), Ruth Jepson (University of Edinburgh), Anne Ludbrook (University of Aberdeen), and Jill Pell (University of Glasgow). Marion Bennie (University of Strathclyde) also supported this project and helped convene the following clinicians and pharmacists to inform the definition of the intervention and control groups: Simon Hurding, Sean MacBride-Stewart and Margaret Ryan. Finally, we would like to thank Stuart McTaggart and Doug Kidd for their help in accessing and understanding the data.

DATA SHARING STATEMENT

The data analysed within this project are administrative data; the Prescribing Information System (PIS) and the Scottish Morbidity Record – 01 Inpatient (SMR01). Anyone wishing to access the data should apply to the electronic Data Research and Innovation Service (eDRIS): www.isdscotland.org/Products-and-Services/EDRIS/index.asp?Co=Y. Applications to access the data will need to be approved by the Public Benefit and Privacy Panel for Health and Social Care (PBPP): www.informationgovernance.scot.nhs.uk/pbpphsc/. Instructions and guidance on how to apply to access the data can be found here: www.informationgovernance.scot.nhs.uk/pbpphsc/home/for-applicants/.

REFERENCES

1 Scottish Executive. Review of NHS prescription charges and exemption arrangements in Scotland - consultation. Edinburgh. Scottish Executive, 2006. Website: http://www.scotland.gov.uk/Resource/Doc/90909/0021837.pdf (accessed 26 June 2018)

2 NHS Choices. Get help with prescription costs. London. Gov.uk, 2017. Website: https://www.nhs.uk/nhsengland/Healthcosts/pages/Prescriptioncosts.aspx (accessed 26 June 2018)

For peer review only - http://bmjopen.bmj.com/site/about/guidelines.xhtml

3 British Broadcasting Corporation. Prescription charges abolished in Scotland. London. British Broadcasting Corporation, 2011. Website: http://www.bbc.co.uk/news/uk-12928485 (accessed 26 June 2018)

4 Roehr B. Cost keeps one in 10 Canadians from filling prescription. *BMJ* 2012;344. doi:10.1136/bmj.e514 [published Online: 20 January 2012]

5 Citizens Advice Scotland. Prescription charges. Edinburgh. The Scottish Association of Citizens Advice Bureaux - Citizens Advice Scotland, 2006. Website: http://www.cas.org.uk/publications/prescription-charges (accessed 26 June 2018)

6 The Scottish Parliament. Summary of responses and conclusions: abolition of NHS prescription charges (Scotland) consultation. Edinburgh. The Scottlish Parliament, n.d. Website: http://www.scottish.parliament.uk/S2_MembersBills/Final%20proposals/abolition-prescription-charges-response.pdf (accessed 26 June 2018)

7 Campbell JD, Allen-Ramey F, Sajjan SG, et al. Increasing pharmaceutical copayments: impact on asthma medication utilization and outcomes. *Am J Manag Care* 2011;17:703-10.

8 Kulik A, Desai NR, Shrank WH, et al. Full prescription coverage versus usual prescription coverage after coronary artery bypass graft surgery: analysis from the post-myocardial infarction Free Rx Event and Economic Evaluation (FREEE) randomized trial. *Circulation* 2013;128:S219-S25. doi:10.1161/circulationaha.112.000337 [published Online: 20 September 2013]

9 Booth GL, Bishara P, Lipscombe LL, et al. Universal drug coverage and socioeconomic disparities in major diabetes outcomes. *Diabetes Care* 2012;35:2257-64. doi:10.2337/dc12-0364 [published Online: 23 October 2012]

10 NHS Wales. Helping to improve Wales's health: free prescriptions three years on. Cardiff. NHS Wales, 2010. Website: http://www.wales.nhs.uk/documents/prescriptions-report-three-years.pdf (accessed 26 June 2018)

11 Jewell T. Have dispensed items really risen with free prescriptions? *BMJ* 2008;337. doi:10.1136/bmj.a1581 [published Online: 10 September 2008]

For peer review only

12 Groves S, Cohen D, Alam MF, et al. Abolition of prescription charges in Wales: the impact on medicines use in those who used to pay. *International Journal of Pharmacy Practice* 2010;18:332-40. doi:10.1111/j.2042-7174.2010.00063.x [published Online: 5 October 2010]

13 Dhippayom T, Walker R. Impact of the reduction of the prescription charge in Wales on the prescribing of non-sedating antihistamines in primary care. *Health Policy* 2008;87:309-15. doi:10.1016/j.healthpol.2008.01.006 [published Online: 6 March 2008]

14 Purdy S, Griffin T, Salisbury C, et al. Ambulatory care sensitive conditions: terminology and disease coding need to be more specific to aid policy makers and clinicians. *Public Health* 2009;123:169-73. doi:10.1016/j.puhe.2008.11.001 [published Online: 13 January 2009]

15 The Scottish Public Health Observatory. Asthma: key points. Glasgow. The Scottish Public Health Observatory, 2017. Website: http://www.scotpho.org.uk/health-wellbeing-and-disease/asthma/key-points/ (accessed 26 June 2018)

16 The Scottish Public Health Observatory. Chronic Obstructive Pulmonary Disease (COPD): key points. Glasgow. The Scottish Public Health Observatory, 2017. Website: http://www.scotpho.org.uk/health-wellbeing-and-disease/chronic-obstructive-pulmonary-disease-copd/key-points/ (accessed 26 June 2018)

17 The Scottish Public Health Observatory. Diabetes: key points. Glasgow. The Scottish Public Health Observatory, 2017. Website: http://www.scotpho.org.uk/health-wellbeing-and-disease/diabetes/key-points/ (accessed 26 June 2018)

18 Stata statistical software: release 14 [program]. College Station, TX: StataCorp LP, 2015.

19 Scottish Government. The Scottish Index of Multiple Deprivation. Edinburgh. Scottish Government, 2017. Website: http://www.gov.scot/Topics/Statistics/SIMD (accessed 26 June 2018)

20 Wood SN. Thin plate regression splines. *J R Stat Soc Series B Stat Methodol* 2003;65:95-114. doi:10.1111/1467-9868.00374 [published Online: 28 January 2003]

For peer review only

For peer review only - http://bmjopen.bmj.com/site/about/guidelines.xhtml

21 Wood SN. Stable and efficient multiple smoothing parameter estimation for generalized additive models. *J Am Stat Assoc* 2004;99:673-86. doi:10.1198/016214504000000980 [published Online: 01 January 2012]

22 Wood SN. Fast stable restricted maximum likelihood and marginal likelihood estimation of semiparametric generalized linear models. *J R Stat Soc Series B Stat Methodol* 2011;73:3-36. doi:10.1111/j.1467-9868.2010.00749.x [published Online: 14 September 2010]

23 Wood SN. *Generalized additive models: an introduction with R*. Second edition. ed. Boca Raton, FL: CRC Press/Taylor & Francis Group, 2017.

24 Wood SN, Pya N, Säfken B. Smoothing parameter and model selection for general smooth models. *J Am Stat Assoc* 2016;111:1548-63. doi:10.1080/01621459.2016.1180986 [published Online: 04 January 2017]

25 Stricker BH. Epidemiology and 'big data'. *Eur J Epidemiol* 2017;32:535-36. doi:10.1007/s10654-017-0294-3 [published Online: 17 August 2017]

26 The Academy of Medical Sciences. Improving the health of the public by 2040: optimising the research environment for a healthier, fairer future. London. The Academy of Medical Sciences, 2016. Website: https://acmedsci.ac.uk/file-download/41399-5807581429f81.pdf (accessed 26 June 2018)

27 Royal Statistical Society. The opportunities and ethics of big data. London. Royal Statistical Society, 2016. Website: http://www.rss.org.uk/Images/PDF/influencing-change/2016/rss-report-opps-and-ethics-of-big-data-feb-2016.pdf (accessed 26 June 2018)

28 Kitchin R. Big Data, new epistemologies and paradigm shifts. *Big Data Soc* 2014;1:2053951714528481. doi:10.1177/2053951714528481 [published Online: 01 April 2014]

Figure 1 – Prescription fees in Scotland compared to England; (a) fee per item, (b) equivalent number of items purchased per month using prepayment certificates

For peer review only - http://bmjopen.bmj.com/site/about/guidelines.xhtml

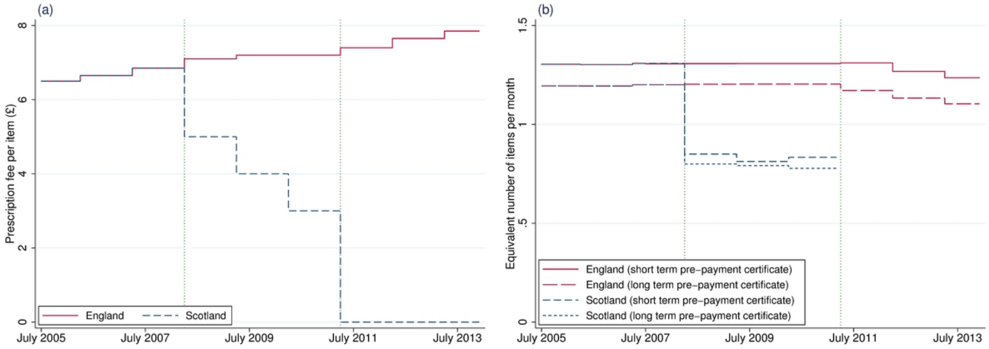

Figure 1 – Prescription fees in Scotland compared to England; (a) fee per item, (b) equivalent number of items purchased per month using prepayment certificates

101x36mm (300 x 300 DPI)

Supplemental file 1 – National Health Service Prescription Fee exemption Criteria [1]

Medical Grounds:

- Forms of hypoadrenalism (including Addison's disease) for which specific substitution therapy is essential
- Diabetes insipidus or other forms of hypopituitarism
- Diabetes mellitus (except where treatment is by diet alone)
- Hypoparathyroidism
- Myasthenia gravis
- Myxodoema (hypothyroidism requiring thyroid hormone replacement)
- Epilepsy requiring continuous anti-convulsive therapy
- A permanent fistula, requiring an appliance or continuous surgical dressing
- Continuing physical disability which prevents the patient from leaving their residence without the help of another person
- Cancer (added in 2009) including any prescriptions for current or previous cancer treatment

Pregnant or had a baby in the previous 12 months (these are included with the medical exemptions)
Contraceptives (these are included with the medical exemptions)
Age:

- Under 16 years
- 16-18 years and in full time education
- Over 60 years

Financial situation:

- Those receiving Income Support, Income-Based Job Seeker's Allowance, Income-related Employment and Support Allowance, Pension Credit Guarantee Credit, Universal Credit and people who qualify for full help under the NHS Low Income Scheme are exempt.

References

1 NHS Choices. Get help with prescription costs. London. Gov.uk, 2017. Website: https://www.nhs.uk/nhsengland/Healthcosts/pages/Prescriptioncosts.aspx (accessed 26 June 2018)

For peer review only

Supplemental file 2 Study protocol

Farr Institute @ Scotland – NEA group / SCPHRP
Andrew James Williams

Natural Experiment

'Universal' Free Prescriptions in Scotland

eDRIS project number: ED13-0487
Primary contact: Andrew James Williams
Other team members: John Frank, Ruth Jepson, Jill Pell, Chris Dibben, Anne Ludbrook and Marion Bennie.

Background

Following devolution each of the four nations of the United Kingdom (UK) (England, Wales, Scotland and Northern Ireland) have taken different approaches to subsidising and funding prescribed medications (British Broadcasting Corporation, 2011). Only England continues to charge a prescription fee which stands at £7.85 in 2013 (Department of Health, 2013). When Scotland abolished the prescription charge the then Health Secretary, Nicola Sturgeon, described prescription fees as 'a tax on ill-health' (British Broadcasting Corporation, 2011).

For those with chronic conditions one of the purposes of pharmaceuticals is to control the condition and subsequently reduce the need for hospitalisation and more invasive and costly intervention. Purdy et al. (2009) have defined a list of conditions sensitive to ambulatory care which are listed in Appendix 1. The appropriate management and treatment of these conditions in primary care should prevent the need for admission into secondary care (Purdy et al., 2009). The success of pharmaceutical treatments is dependent upon the patient's adherence to the dosage regimen prescribed by the practitioner. Kardas et al. (2013) recently reviewed the determinants of non-adherence. Seven hundred and seventy one determinants of whether someone initiated and/or persisted with a medication were identified which fell into the following five broad clusters; socioeconomic-related, healthcare team- and system-related, condition-related, therapy-related, and patient-related (Kardas et al., 2013). Among the socioeconomic cluster the eight studies which examined cost, all found higher cost to be associated with poorer adherence (Kardas et al., 2013). Therefore, the economic logic behind the abolition of prescription fees is that adherence will improve, and therefore hospital admissions will decrease reducing secondary care costs, which hopefully compensate for the additional cost to the state of the prescriptions (Figure 1).

Previous research has tended to focus on elements of the logic model rather than the whole. Linnet et al. (2013), Yin et al. (2008) and Zhang et al. (2008) focussed upon the first half of the logic model, the effect of prescription fee policies on the dispensing of pharmaceuticals with mixed results. Yin et al. (2008) and Zhang et al. (2008) found that the introduction of prescription subsidies (Medicare part D) for the elderly in America resulted in an increased number of prescriptions and reduced out-of-pocket expenses for the patient. Whereas Linnet et al. (2013) found that within Iceland a small increase in prescription charge did not significantly affect whether a patient cashed/filled a prescription. Booth et al. (2012) when examining the socioeconomic disparities in diabetes mellitus related morbidity and mortality found that the socioeconomic disparities greatly reduced in each of the outcomes studied for those over 65 years of age (Booth et al., 2012). Within Ontario, Canada (the location of the study) those over the age of 65 receive free prescriptions and therefore the decreased socioeconomic disparities was attributed to this policy (Booth et al., 2012). Booth et al. (2012) employed a number of

For peer review only - http://bmjopen.bmj.com/site/about/guidelines.xhtml

statistical techniques to test this hypothesis (sensitivity analyses) and continued to find the decreased disparities associated with the free prescription policy. Campbell et al. (2011) and Kulik et al. (2013) employed more experimental study designs to test the logic model. Kulik et al. (2013) undertook a randomised controlled trial into the effect of offering free prescriptions to patients immediately following a myocardial infarction and found improved adherence and reduced patient out-of-pocket expenses and a trend towards improved clinical outcomes. Campbell et al. (2011) used linked data within a natural experimental approach to examine the effect of prescription costs for those with asthma. It was found that those experiencing increases in prescription charges (co-payment) ≥$5(US) were significantly less adherent than those experiencing increases of <$5 and also had higher healthcare usage (Campbell et al., 2011).

Figure 1 – Logic model for study

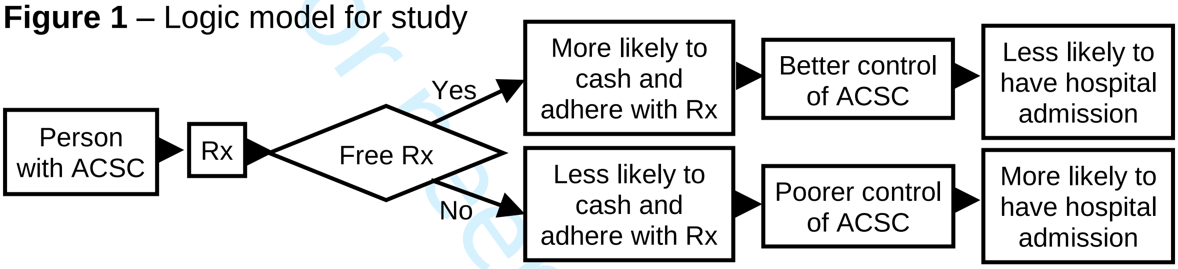

ACSC; ambulatory case sensitive condition, Rx; prescription

Subsequently, there is some evidence that reduced or free prescriptions have positive effects on adherence and health. However, the studies so far have focused upon certain age groups or conditions and yet within Wales, Northern Ireland and Scotland prescriptions are free for all. Three years after the abolition of prescription fees in Wales a process evaluation was published by the health authority (NHS Wales, 2010). This evaluation found that the abolition of prescription fees did not result in a change in the number of prescriptions and their cost to the state. Jill Pell and colleagues are undertaking a similar study examining the first half of the logic model (Figure 1) in Scotland. However, to the best of our knowledge there have been no studies within the UK to examine the whole logic model in relation to the universal free prescription policy.

Aims

To identify whether the stepped reduction and abolition of prescription fees in Scotland resulted in:

a) increase in the number (cost to NHS) of prescriptions for inhaled steroids;
b) reduction in hospital admissions for asthma or chronic obstructive pulmonary disease (COPD).

When compared to prescriptions and admissions for a condition for which prescriptions were historically free (diabetes mellitus) and or those who received free prescriptions due to age.

Moving of from these preliminary aims more refined analysis is planned to address these two further questions.

c) Was any change in hospital admissions for asthma or COPD in the practices which also saw a change in prescribing/dispensing?
d) How did the effect differ across the socioeconomic spectrum?

For peer review only - http://bmjopen.bmj.com/site/about/guidelines.xhtml

Supplemental file 2 Study protocol

Farr Institute @ Scotland – NEA group / SCPHRP
Andrew James Williams

Proposal

As a policy the abolition of prescription fees was not introduced experimentally (e.g. with randomisation), and subsequently we propose to address the above aims using time series of routinely collected data as a natural experiment (Craig et al., 2011). The primary sources for the routinely collected data will be the Scottish Morbidity Record (SMR01) and the Prescriptions Information System (PIS). Through extensive discussion with the Principle Pharmacist (Stuart McTaggart) at NHS National Services Scotland, it has been established that individual-level data suitable for this project are not currently available in the PIS. The primary reasons that individual-level data from the PIS cannot be used are the inability to calculate measures of adherence (Fairman and Matheral, 2000, Lehmann et al., 2013), and the fact that currently individual-level data are not available prior to August 2009 when Scotland had already begun reducing prescription fees. Subsequently, we propose to proceed with a practice-level study as outlined below. Currently, the proposed study is limited to Scotland, although through the Farr network a UK wide study may be considered at a later date.

Critical to any natural experiment is the identification of the population who were/are effected by the policy (the intervention sample) and appropriate counterfactual sample(s) (Craig et al., 2011). As prescription fees were reduced and ultimately abolished contemporaneously across Scotland and the data being used only exist in Scotland there is no location-based contemporary counterfactual. However, the existence of prescription fee exemption criteria (Appendix 2) prior to the abolition of fees permits the identification of the intervention and counterfactual samples. Although, as following the abolition of prescription fees there was no need to record exemption criteria, the samples also had to remain identifiable within the PIS and SMR01. Furthermore, as only one exemption criteria needed to be recorded when collecting a prescription, it was and will remain necessary throughout this project to remain aware that although marked exempt for one reason the patient may have also met other exemption criteria.

The exemption criteria (Appendix 2) fall into three groups, age- condition- and income-related all of which could have formed the basis of counterfactual samples. The income-related exemptions group may have permitted a regression discontinuity design, however, it would not be possible to identify this sample following the abolition of fees and consequently no income-based counterfactual was sought. The age-related exemption criteria would form an age-based counterfactual sample. This is made possible as prior to the abolition of fees the PIS exemptions category data would identify this sample and following the fee abolition individual-level PIS data could inform the identification of this group. Those aged 16-18 years in full time education also received free prescriptions (Appendix 2); as this subpopulation can only be identified prior to the abolition of fees they will initially be examined separately to ascertain what approach is most appropriate for this subpopulation. However, wanting to strengthen the causal inference it is planned to examine dispensed prescriptions as a mediator of change in health (admissions) and consequently it is necessary to identify suitably sensitive and specific condition medication pairings. Thus, the condition-related exemption criteria were crucial. These pairings needed to meet the following requirements;

- The condition needed to be chronic (e.g. not ear, nose and throat infections).

For peer review only - http://bmjopen.bmj.com/site/about/guidelines.xhtml

Supplemental file 2 Study protocol

- The condition needed to be sensitive to long term pharmaceutical treatment specifically not just ambulatory care in general (e.g. not dental complications).
- Relatively minor changes in adherence (e.g. missed tablets) to the treatment for the condition needed to result in the need for hospital attention (e.g. not schizophrenia or epilepsy).
- The condition needed to be sufficiently prevalent across the life-course to provide sufficient power for the analysis (e.g. not senility/dementia).
- The intervention condition needed to not be a condition-related exemption from prescription fees (Appendix 2) or commonly co-occurring with a condition on that list (e.g. not diabetes mellitus or any of the cardiovascular conditions).
- The counterfactual condition needed to be a condition-related exemption from prescription fees (e.g. diabetes mellitus).
- The pharmaceutical treatment (medicine) needs to be fairly specific to the condition (e.g. not congestive heart failure).

The final samples are outlined in the box below, with asthma/COPD selected as the intervention condition. Asthma/COPD had been noted for their absence from the exemption criteria in the Scottish public consultation about the abolition of prescription fees (Scottish Executive, 2006, The Scottish Parliament, n.d.). These samples have been defined after extensive consultation with expert pharmacists and general practitioners.

**Sample**	**Eligibility criteria**
Intervention	Those aged ≥16-<60 years that had paid for dispensed prescriptions for steroid inhalers which is assumed to indicate diagnosis with asthma or chronic obstructive pulmonary disease (COPD).
Age-based counterfactual	Those age <16 or ≥60 years that had dispensed prescriptions for steroid inhalers which is assumed to indicate diagnosis with asthma or COPD.
Condition-based counterfactual	Those that had dispensed prescriptions for diabetes mellitus treatments which are assumed to indicate diagnosis with said condition.

As only one exemption criteria would have been recorded for each prescription it is not possible to sub-divide the condition-based counterfactual by age. As previously discussed the analysis is forced to examine practice- rather than individual-level data and subsequently the data will be numbers/proportions of relevant prescriptions/admissions for each practice.

Analysis

The studies discussed in the penultimate paragraph of the Background to this document employed a number of statistical methods ranging from the simple univariable analysis to complicated hierarchical multivariable models taking in techniques such as logistic regression (Booth et al., 2012, Kulik et al., 2013, Yin et al., 2008, Zhang et al., 2008) and Cox proportional hazard models (Booth et al., 2012, Kulik et al., 2013). The data to be collected, linked and analysed in the current

For peer review only - http://bmjopen.bmj.com/site/about/guidelines.xhtml

project is outlined in Appendix 3, the intention being to produce a panel dataset. For each practice there will be annually 'fixed' socio-demographic data from the General Practice dataset, and monthly (January 2000-December 2013) admissions and prescriptions data. Analysis will be undertaken in Stata (StataCorp, 2009) and will take the format of time series and mediation analysis.

The primary health outcome will be hospital admissions for the respective conditions. The PIS extract dataset will provide outcome data on the potential mediators of any change in the primary outcome. Without individual data and the ability to measure adherence, a number of prescription related metrics will be evaluated as outcomes and mediators. The following metrics will be examined for each of the medication groups (inhaled steroids and diabetes treatments);

- Number of dispensed prescriptions,
- Quantity dispensed,
- Cost of the items dispensed,
- Number of Defined Daily Doses (DDDs) dispensed.

Including the admissions, but excluding costs all the outcomes are count data and consequently will be analysed as such (Poisson regression Generalised Estimating Equations (GEE) or Mixed Models). Cost will be analysed as a continuous outcome. Initially some of the outcomes may be turned into ratios (e.g. DDDs per prescription or prescriptions per general practitioner) in order to smooth the data and account for differences in practice size, to identify the most appropriate set of outcomes. Auto-regression, integration (differencing) and moving average (ARIMA) adjustments will also be explored, to smooth the data and in particular account for the seasonality of dispensing.

Figure 2 – Mediation model for analysis

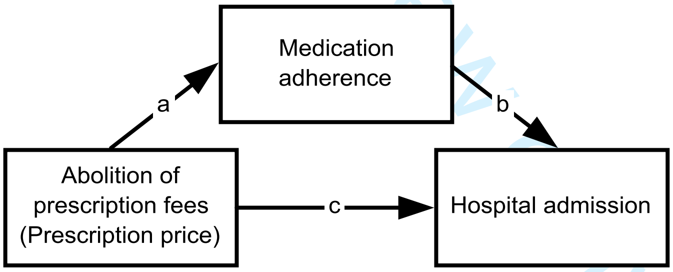

Taking the study aims and the logic model (Figure 1) Figure 2 illustrates the mediation model to be examined through the analysis. The studies discussed in the background provide evidence of relationships **a** and **b** in Figure 2. The preliminary aims of this study (study aims a and b) will be addressed by constructing separate time series of prescriptions and admissions in order to test relationships **a** and **c**. As a practice-level study it will not be possible to directly test relationship **b**. As with previous studies (e.g. Yin et al., 2008, Booth et al., 2012) splines and knots will be employed in order to assess whether changes in admissions and/or prescriptions were contemporary with the reductions and eventual abolition of fees. Furthermore, the use of splines and knots will permit some quantification of any effect of the policy change. The admissions time series will then be adjusted for the prescriptions time series (study aim c), in order to assess the extent to which relationship **c** remains, which will provide information on the nature of relationship **b**. Should relationship **c** remain after adjusting for prescriptions, it would indicate that within the limitations of

For peer review only - http://bmjopen.bmj.com/site/about/guidelines.xhtml

this study the policy change had not impacted on health. These analytical steps should strengthen to the study findings.

Given the extent of exemptions criteria (Appendix 2), during the Scottish public consultation it was questioned who would benefit from abolishing the fee (Scottish Executive, 2006, The Scottish Parliament, n.d.). The exemption criteria (Appendix 2) were (and still are in England) considered to be protecting the poorest and sickest who would struggle to pay. It was felt that a reappraisal of the condition-related exemption criteria to consider including conditions like asthma, COPD, dementia might be a sufficient change. As it was, prior to the abolition of prescription fees around 90% of prescriptions were exempt from payment (Personal communication with S. McTaggart, 01/04/2014). In order to examine for the differential impact of the policy across the socioeconomic spectrum (study aim d), the effect of adjusting or stratifying the time series by deprivation and other General Practice characteristics will be explored.

Special cases: As previously discussed those aged 16-18 years will initially be examined separately. Similarly, there is another group of patients who will initially be examined separately. Prior to the abolition of fees, those with chronic conditions were able to purchase discount pre-payment certificates. The price of these certificates changed in line with the reduction in fees. Data published in 2009 demonstrated that there had been a significant increase in the use of pre-payment certificates following the initial reduction in prescription fees (The Scottish Government, 2009). That finding and the different financial decisions related to pre-payment certificates have necessitated the initial examination of this group separately. The use of a pre-payment certificate was captured within the exemptions data within the PIS.

Potential impact

From a research perspective this project would add to the literature on the importance of cost as a determinant of non-adherence with prescribed medications. Given the large number of exemptions from prescription fees prior to the policy change (see Rationale) some have questioned whether universal free prescriptions are just benefiting the less deprived. This study should reveal what the implications of universal free prescriptions have been for those of different socioeconomic status. Some have also questioned whether having to pay for a prescription is an incentive to adherence, although there does not appear to be evidence to back this up, this needs to be considered within this project, the results of which might support or refute that claim. Politically, the project will evaluate an important and possibly costly policy and provide data on the ‘effects’ of the policy. In particular, should a time arise that free prescriptions for all cannot be funded by the state; the stratified component of this project might suggest conditions or circumstances for which free prescriptions are cost effective.

For peer review only - http://bmjopen.bmj.com/site/about/guidelines.xhtml

Supplemental file 2 Study protocol

Farr Institute @ Scotland – NEA group / SCPHRP
Andrew James Williams

References

Booth, G. L., Bishara, P., Lipscombe, L. L., Shah, B. R., Feig, D. S., Bhattacharyya, O. & Bierman, A. S. 2012. Universal drug coverage and socioeconomic disparities in major diabetes outcomes. *Diabetes Care,* 35**,** 2257-2264.

British Broadcasting Corporation. 2011. *Prescription charges abolished in Scotland* [Online]. London: British Broadcasting Corporation. Available: http://www.bbc.co.uk/news/uk-12928485 [Accessed 24 September 2013].

Campbell, J. D., Allen-Ramey, F., Sajjan, S. G., Maiese, E. M. & Sullivan, S. D. 2011. Increasing pharmaceutical copayments: impact on asthma medication utilization and outcomes. *Am J Manag Care,* 17**,** 703-10.

Craig, P., Cooper, C., Gunnell, D., Haw, S., Lawson, K., Macintyre, S., Ogilvie, D., Petticrew, M., Reeves, B., Sutton, M. & Thompson, S. 2011. *Using natural experiments to evaluate population health interventions: guidance for producers and users of evidence,* London, Medical Research Council. Available: http://www.mrc.ac.uk/documents/pdf/natural-experiments-guidance/ [Accessed 26 February 2014]

Department of Health. 2013. *NHS charges from April 2013 announced* [Online]. London: GOV.UK. Available: https://www.gov.uk/government/news/nhs-charges-from-april-2013-announced [Accessed 15 October 2013].

Fairman, K. & Matheral, B. 2000. Evaluating medication adherence: which measure is right for your program? *Journal of Managed Care Pharmacy,* 6**,** 499-506.

Kardas, P., Lewek, P. & Matyjaszczyk, M. 2013. Determinants of patient adherence: a review of systematic reviews. *Frontiers in Pharmacology,* 4(91).

Kulik, A., Desai, N. R., Shrank, W. H., Antman, E. M., Glynn, R. J., Levin, R., Reisman, L., Brennan, T. & Choudhry, N. K. 2013. Full prescription coverage versus usual prescription coverage after coronary artery bypass graft surgery: analysis from the post-myocardial infarction Free Rx Event and Economic Evaluation (FREEE) randomized trial. *Circulation,* 128**,** S219-S225.

Lehmann, A., Aslani, P., Ahmed, R., Celio, J., Gauchet, A., Bedouch, P., Bugnon, O., Allenet, B. & Schneider, M. P. 2013. Assessing medication adherence: options to consider. *Int J Clin Pharm*, 36(1):55-69.

Linnet, K., Halldórsson, M., Thengilsdóttir, G., Einarsson, Ó. B., Jónsson, K. & Almarsdóttir, A. B. 2013. Primary non-adherence to prescribed medication in general practice: lack of influence of moderate increases in patient copayment. *Family Practice,* 30**,** 69-75.

NHS Choices. 2013. *Get help with prescription costs* [Online]. London: GOV.UK. Available: http://www.nhs.uk/NHSEngland/Healthcosts/Pages/Prescriptioncosts.aspx [Accessed 15 October 2013].

NHS Wales 2010. *Helping to improve Wales's health: free prescriptions three years on,* Cardiff, NHS Wales. Available: http://www.wales.nhs.uk/documents/prescriptions-report-three-years.pdf [Accessed 25 September 2013]

Purdy, S., Griffin, T., Salisbury, C. & Sharp, D. 2009. Ambulatory care sensitive conditions: terminology and disease coding need to be more specific to aid policy makers and clinicians. *Public Health,* 123**,** 169-173.

Scottish Executive. 2006. *Review of NHS prescription charges and exemption arrangements in Scotland - consultation* [Online]. Edinburgh: Scottish Executive. Available:

For peer review only - http://bmjopen.bmj.com/site/about/guidelines.xhtml

Supplemental file 2 Study protocol

http://www.scotland.gov.uk/Resource/Doc/90909/0021837.pdf [Accessed 19 February 2014].

StataCorp 2009. Stata statistical software: release 11. College Station, TX: StataCorp LP.

The Scottish Government. 2009. *News; prescription charges* [Online]. Edinburgh: The Scottish Government. Available: http://www.scotland.gov.uk/News/Releases/2009/01/28095040 [Accessed 28 January 2014].

The Scottish Parliament. n.d. *Summary of responses and conclusions: abolition of NHS prescription charges (Scotland) consultation* [Online]. Edinburgh: The Scottish Parliament. Available: http://www.scottish.parliament.uk/S2_MembersBills/Final%20proposals/abolition-prescription-charges-response.pdf [Accessed 19 February 2014].

Yin, W., Basu, A., Zhang, J. X., Rabbani, A., Meltzer, D. O. & Alexander, G. C. 2008. The effect of the Medicare Part D prescription benefit on drug utilization and expenditures. *Ann Intern Med,* 148**,** 169-77.

Zhang, J. X., Yin, W., Sun, S. X. & Alexander, G. C. 2008. The impact of the Medicare Part D prescription benefit on generic drug use. *J Gen Intern Med,* 23**,** 1673-8.

Appendix 1: Ambulatory case sensitive conditions Purdy et al. (2009)

Angina	Nutritional deficiency	Hypokalaemia	Stroke
Asthma	Hypertension	Low birth weight	Pyelonephritis
Cellulitis	Failure to thrive	Neuroses	Diabetes complications
Dental complications	Ruptured appendix	Tuberculosis	Gangrene
Constipation	Deliberate self-harm	Senility/dementia	Schizophrenia
Congestive heart failure	Fractured proximal femur	Influenza and pneumonia	Migraine/acute headache
Iron-deficiency anaemia	Alcohol-related disease	Atrial fibrillation/flutter	Convulsions and epilepsy
Dehydration and gastroenteritis	Perforated/bleeding ulcer	Ear, nose and throat infections	Pelvic inflammatory disease
Other vaccine-preventable diseases	Dyspepsia and other stomach function disorders	Chronic obstructive pulmonary disease	Peripheral vascular disease

Appendix 2: Criteria exempting patients from paying prescription fees in England and Scotland prior to April 2011 (NHS Choices, 2013).

- Condition-related
  - Cancer (from 2009)
  - Those on renal dialysis with a permanent fistula
  - Pregnant
  - Contraceptives
  - Sexually transmitted infection
  - Tuberculosis

For peer review only - http://bmjopen.bmj.com/site/about/guidelines.xhtml

Supplemental file 2 Study protocol

    - Hypoadrenalism for which specific substitution therapy is essential
    - Diabetes insipidus or other forms of hypopituitarism
    - Diabetes mellitus, except where treatment is by diet alone
    - Hypoparathyroidism
    - Myasthenia gravis
    - Myxoedema
    - Epilepsy requiring continuous anticonvulsive therapy
    - A continuing physical disability which means the person cannot go out without the help of another person.
- Age-related
    - are 60 or over
    - are under 16
    - are 16-18 and in full-time education
- Means tested, and those in receipt of the following benefits ('income-related')
    - Income Support
    - Income-based Jobseeker's Allowance
    - Income-related Employment and Support Allowance
    - Pension Credit Guarantee Credit
    - Universal Credit
    - Those in receipt of a war pension

Appendix 3: Data
The final study dataset will be compiled from three separate datasets. Two of these datasets will be extracts from the Scottish Morbidity Record (SMR01) and Prescriptions Information System (PIS). The third dataset will be the General Practice data compiled from data published by ISD Scotland (http://www.isdscotland.org/Health-Topics/General-Practice/) and more historical data provided through eDRIS. The General Practice dataset will be linked by the researcher using the practice code. The SMR01 and PIS data extracts will be linked by the researcher using the practice code, year and month. The General Practice dataset will then be linked onto the linked SMR01 and PIS data, and the data rearranged in order that each month of SMR01 and PIS data will only have the relevant years general practice descriptive variables. The final dataset should be in the form of panel data for the time series analysis. The variables in each of the three datasets are listed below.

N.B. Gender and ethnicity data are not available in the practice-level PIS and have subsequently not been sought in the other data.

For peer review only - http://bmjopen.bmj.com/site/about/guidelines.xhtml

Supplemental file 2 Study protocol

Farr Institute @ Scotland – NEA group / SCPHRP
Andrew James Williams

General Practice dataset – by practice and year

**Variable**	**Details**
NHS board (code)	Unique single alphabetical character for each health board (publically available)*
NHS board (name)	Name of each health board (publically available)*
Practice Code	The unique NHS Scotland code for the GP Practice (publically available)
Practice name	Name of each General Practice (publically available)*
Number of General Practitioners	(requested for each year through eDRIS)
Dispensing practice	Identifier of practices where medicines are dispensed as well as prescribed (requested for each year through eDRIS)
Contract type	Indicator of which type of contract the practice has with the NHS (requested for each year through eDRIS)
Practice List Size	Total number of registered patients each year (publically available for the years 1999-2013)
Age profile of practice list	Number of patients in each of the following age categories; <16, 16-18, 18-60, ≥60 years (requested for each year through eDRIS)
pc_most_dep15	Percentage of practice patients living in datazones defined as the 15% most deprived (population weighted) (publically available from 2005-2013, earlier years requested through eDRIS)
quint1 – quint5	Number of practice patients matched to the relevant first through fifth quintile of deprivation (publically available from 2005-2013, earlier years requested through eDRIS)
prac_ur8	Urban/rural classification of the postcode of the General Practice (publically available from 2005-2013, earlier years requested through eDRIS)
urban1 – urban8	Number of practice patients in each of the eight urban/rural classifications (publically available from 2005-2013, earlier years requested through eDRIS)
unmatch	Number of practice patients who could not be matched to an urban/rural classification (publically available from 2005-2013, earlier years requested through eDRIS)
mode_ur8	Modal urban/rural classification of the practice patients (publically available from 2005-2013, earlier years requested through eDRIS)

*These data are for linking and/or clustering purposes and would be anonymised in any publications.

For peer review only - http://bmjopen.bmj.com/site/about/guidelines.xhtml

Supplemental file 2 Study protocol

Farr Institute @ Scotland – NEA group / SCPHRP
Andrew James Williams

Scottish Morbidity Record (SMR01) extract dataset – by practice, year and month

Variable	Details
Practice Code	The unique NHS Scotland code for the GP Practice
Year	The year to which the data relates (2000-13)
Month	The month to which the data relates
Diagnosis	The diagnosis (based on DIAGNOSIS 1) to which the data relates (ALL admissions, Asthma/COPD† or Diabetes‡
Age group	The age banding (0-15, 16-18, 19-59, 60+ years) to which the data relates
Admissions	The total number of admissions for practice patients

†International Classification of Disease 10 codes J20, J40X, J41, J42, J43, J44, J45, J46 or J47
‡International Classification of Disease 10 codes E10, E11, E12, E13 or E14

Prescriptions Information System (PIS) extract dataset – by practice, year and month

Variable	Details
Practice Code	The unique NHS Scotland code for the GP Practice
Year	The year to which the data relates (2000-13)
Month	The month to which the data relates
Category	The medication class to which the data relates (ALL prescriptions, Inhaled Corticosteroids or Diabetes)
Paid/Exempt	A flag to indicate whether the data relates to Paid or Exempt prescriptions
Exemption Category*	The prescription exemption category to which the data relates
Number of Items Paid	The number of prescription items paid
Quantity Paid	The total quantity paid
Gross Ingredient Cost Paid	The total cost of the medicines supplied before discount
DDDs Paid	The total number of defined daily doses paid

*For data from April 2011, exemption category will not be available. This should be replaced by the following age-bandings: 0-15, 16-18, 19-59; 60+ years. Ages should be based on the date of prescribing.

For peer review only - http://bmjopen.bmj.com/site/about/guidelines.xhtml

Supplemental file 3 Generalized Additive Mixed Models sensitivity analysis

Admissions

Intervention

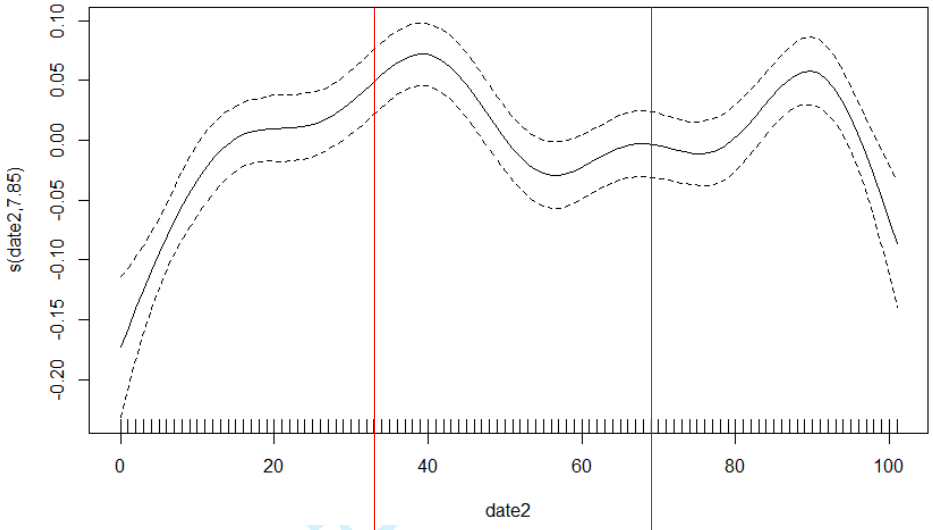

Age counterfactual

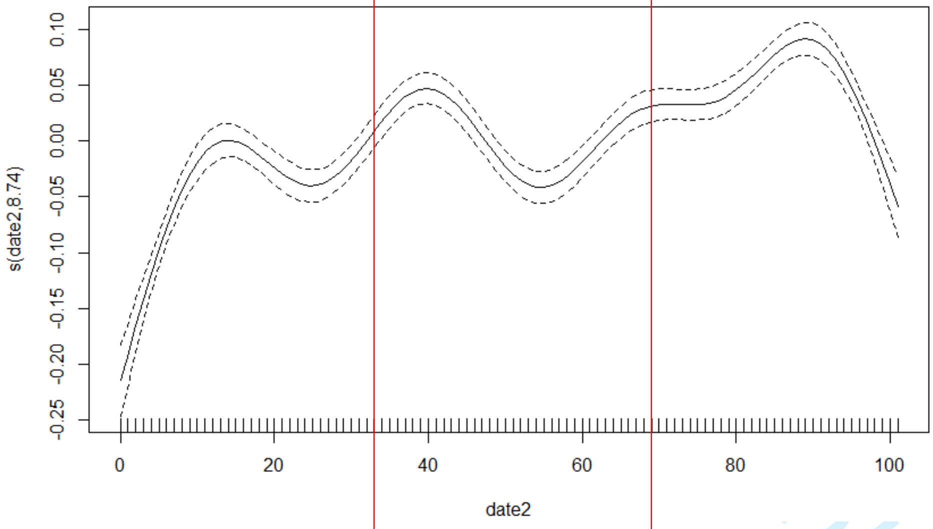

Condition counterfactual

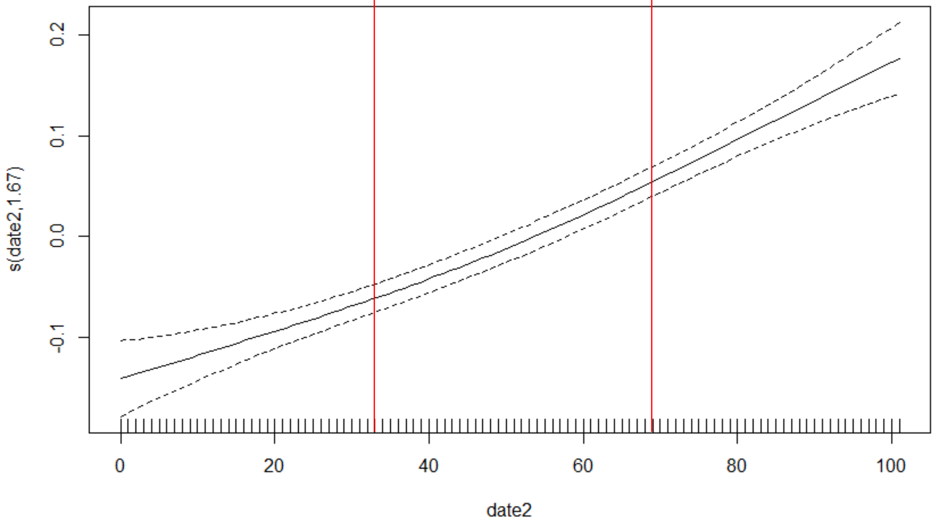

Supplemental file 3 Generalized Additive Mixed Models sensitivity analysis

Defined Daily Doses (DDDs)

Intervention

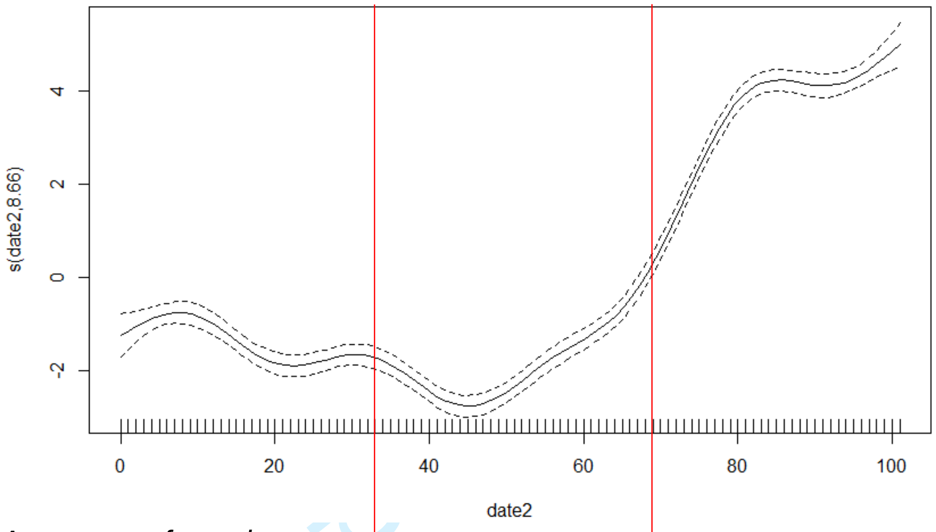

Age counterfactual

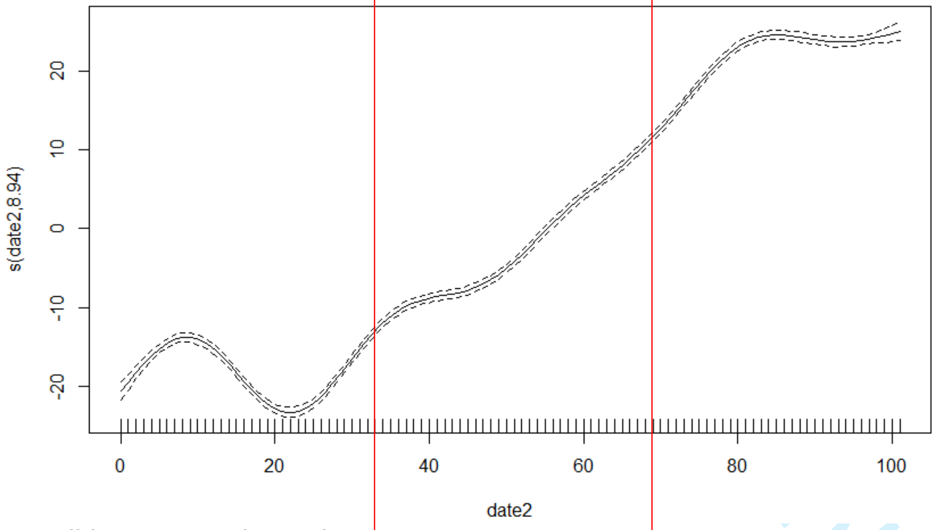

Condition counterfactual

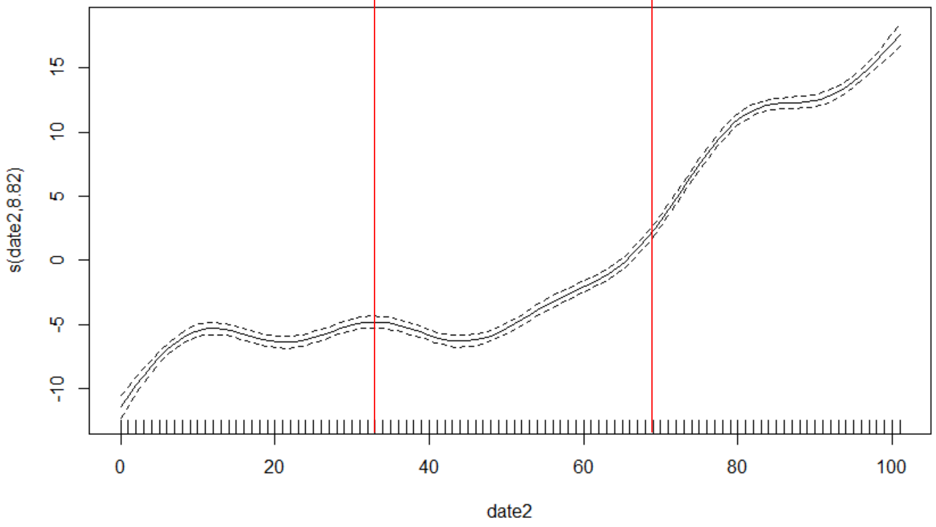

Supplemental file 3 Generalized Additive Mixed Models sensitivity analysis

Cost

Intervention

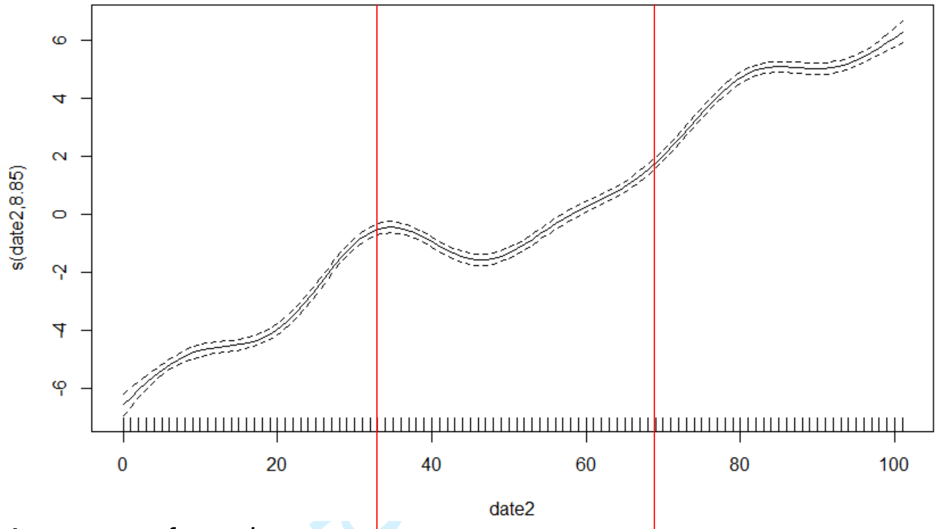

Age counterfactual

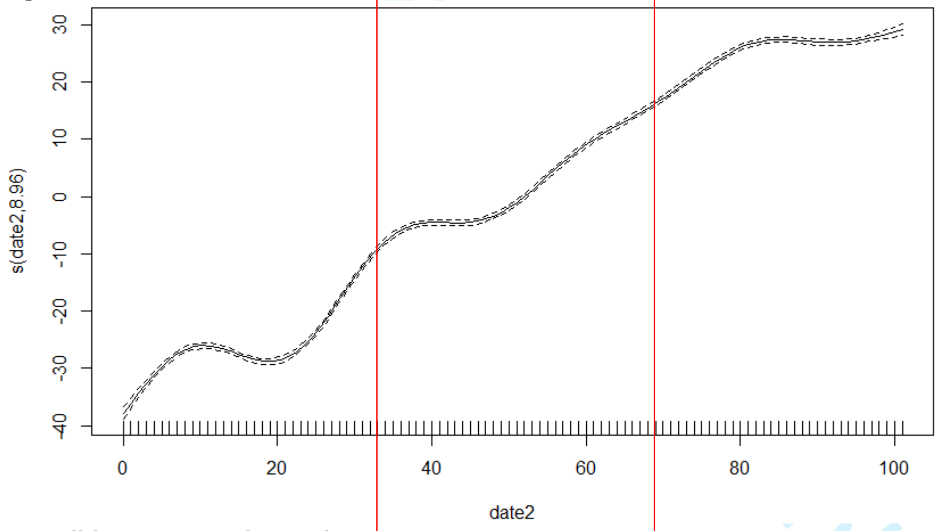

Condition counterfactual

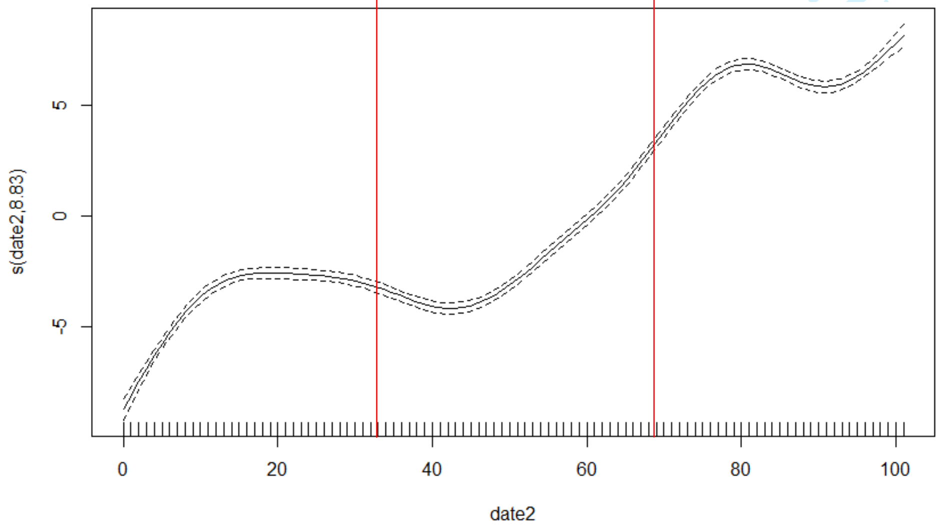

Supplemental file 4 Health board clustering sensitivity analysis

Table S4.1 – Results of interrupted time series analysis of the impact of reducing and abolishing prescription fees in Scotland on hospital admissions (incidence rate ratios) and prescribed medicines (defined daily doses and cost) – adjusting for clustering with General Practice and Health Board

	**Intercept**		**Monthly change**		**Reduction step change**		**Reduction change in slope**		**Abolition step change**		**Abolition change in slope**	
**Admissions**	IR	95% CI	IRR	95% CI	IRR	95% CI	IRR	95% CI	IRR	95% CI	IRR	95% CI
Intervention	$9.04e^{-3}$	$7.75e^{-3}$ to $1.06e^{-2}$	1.00	1.00 to 1.01	1.03	0.98 to 1.08	0.99	0.99 to 0.99	0.81	0.72 to 0.90	1.00	0.99 to 1.00
Age-c'fact	$5.70e^{-2}$	$4.95e^{-2}$ to $6.56e^{-2}$	1.00	1.00 to 1.00	1.03	1.01 to 1.06	1.00	0.99 to 1.00	0.95	0.90 to 1.00	1.00	0.99 to 1.00
Condition-c'fact	$4.72e^{-3}$	$4.14e^{-3}$ to $5.38e^{-3}$	1.00	1.00 to 1.01	0.95	0.88 to 1.03	1.00	1.00 to 1.01	1.01	0.86 to 1.20	1.00	1.00 to 1.01
**Admissions 1 month lag**	IR	95% CI	IRR	95% CI	IRR	95% CI	IRR	95% CI	IRR	95% CI	IRR	95% CI
Intervention	$9.07e^{-3}$	$7.77e^{-3}$ to $1.06e^{-2}$	1.01	1.00 to 1.01	1.01	0.96 to 1.06	0.99	0.99 to 0.99	0.78	0.71 to 0.87	1.00	0.99 to 1.00
Age-c'fact	$5.78e^{-2}$	$5.02e^{-2}$ to $6.65e^{-2}$	1.00	1.00 to 1.00	1.03	1.01 to 1.06	1.00	0.99 to 1.00	0.95	0.90 to 1.00	1.00	0.99 to 1.00
Condition-c'fact	$4.74e^{-3}$	$4.16e^{-3}$ to $5.42e^{-3}$	1.00	1.00 to 1.00	0.98	0.91 to 1.06	1.00	1.00 to 1.00	1.07	0.91 to 1.26	1.00	1.00 to 1.01
**Admissions 2 month lag**	IR	95% CI	IRR	95% CI	IRR	95% CI	IRR	95% CI	IRR	95% CI	IRR	95% CI
Intervention	$9.09e^{-3}$	$7.79e^{-3}$ to $1.06e^{-2}$	1.00	1.00 to 1.01	1.01	0.96 to 1.06	0.99	0.99 to 0.99	0.79	0.71 to 0.87	1.00	0.99 to 1.00
Age-c'fact	$5.80e^{-2}$	$5.04e^{-2}$ to $6.68e^{-2}$	1.00	1.00 to 1.00	1.04	1.02 to 1.07	1.00	0.99 to 1.00	0.97	0.92 to 1.02	1.00	1.00 to 1.00
Condition-c'fact	$4.77e^{-3}$	$4.18e^{-3}$ to $5.44e^{-3}$	1.00	1.00 to 1.00	0.99	0.92 to 1.07	1.00	1.00 to 1.01	1.10	0.93 to 1.29	1.00	1.00 to 1.01
**Admissions 3 month lag**	IR	95% CI	IRR	95% CI	IRR	95% CI	IRR	95% CI	IRR	95% CI	IRR	95% CI
Intervention	$9.07e^{-3}$	$7.77e^{-3}$ to $1.06e^{-2}$	1.01	1.00 to 1.01	0.99	0.94 to 1.04	0.99	0.99 to 0.99	0.78	0.70 to 0.86	1.00	0.99 to 1.00
Age-c'fact	$5.77e^{-2}$	$5.01e^{-2}$ to $6.64e^{-2}$	1.00	1.00 to 1.00	1.02	1.00 to 1.05	1.00	0.99 to 1.00	0.94	0.90 to 0.99	1.00	1.00 to 1.00
Condition-c'fact	$4.74e^{-3}$	$4.15e^{-3}$ to $5.40e^{-3}$	1.00	1.00 to 1.00	0.97	0.90 to 1.05	1.00	1.00 to 1.01	1.08	0.92 to 1.26	1.00	1.00 to 1.00
**Defined Daily Doses (DDDs)**	DDDs	95% CI	ΔDDDs	95% CI	ΔDDDs	95% CI	ΔDDDs	95% CI	ΔDDDs	95% CI	ΔDDDs	95% CI
Intervention	33.21	30.30 to 36.12	-0.04	-0.06 to -0.02	-0.11	-0.61 to 0.38	0.04	0.02 to 0.07	6.03	4.93 to 7.13	0.10	0.08 to 0.13
Age-c'fact	-	-	-	-	-	-	-	-	-	-	-	-
Condition-c'fact	59.82	54.81 to 64.82	0.14	0.10 to 0.18	-1.88	-2.90 to -0.86	-0.05	-0.11 to <0.01	6.02	3.72 to 8.31	0.15	0.09 to 0.20
**Cost**	£	95% CI	Δ£	95% CI	Δ£	95% CI	Δ£	95% CI	Δ£	95% CI	Δ£	95% CI
Intervention	22.44	20.11 to 24.77	0.17	0.15 to 0.18	0.23	-0.20 to 0.65	-0.15	-0.17 to -0.13	-0.97	-1.92 to -0.02	-0.10	-0.12 to -0.08
Age-c'fact	67.90	62.04 to 73.76	0.62	0.56 to 0.68	4.74	3.43 to 6.06	0.03	-0.05 to 0.12	14.26	10.87 to 17.65	-0.36	-0.44 to -0.27
Condition-c'fact	29.61	26.75 to 32.47	0.15	0.13 to 0.17	-3.16	-3.70 to -2.62	-0.01	-0.04 to 0.02	1.99	0.78 to 3.20	-0.11	-0.14 to -0.09

95% CI; 95% confidence interval, c'fact; counterfactual, DDDs; Defined daily doses per 100 patients per practice per month, ΔDDDs; change in DDDs, IR; Incidence rate per 100 patients per practice per month, IRR; incidence rate ratio, £s; gross ingredient cost of medicines before any discount per 100 patients per practice per month, Δ£s; change in £s

Supplemental file 4 Health board clustering sensitivity analysis

Table S4.2 - Results of interrupted time series analysis of the impact of reducing and abolishing prescription fees in Scotland on hospital admissions (incidence rate ratios) and prescribed medicines (defined daily doses and cost) stratified by quintile of the Scottish Index of Multiple Deprivation [19] – adjusting for clustering with General Practice and Health Board

	**Intercept**		**Monthly change**		**Reduction step change**		**Reduction change in slope**		**Abolition step change**		**Abolition change in slope**	
**Admissions**	IR	95% CI	IRR	95% CI	IRR	95% CI	IRR	95% CI	IRR	95% CI	IRR	95% CI
Q1 - most deprived	$9.08e^{-2}$	$7.67e^{-2}$ to $1.08e^{-1}$	1.01	1.00 to 1.01	1.02	0.97 to 1.06	0.99	0.99 to 1.00	0.87	0.79 to 0.97	0.99	0.99 to 1.00
Q2	$1.17e^{-2}$	$9.36e^{-3}$ to $1.47e^{-2}$	1.00	1.00 to 1.01	1.10	0.92 to 1.32	0.99	0.98 to 1.00	0.75	0.50 to 1.12	1.00	0.99 to 1.01
Q3	$9.80e^{-3}$	$7.03e^{-3}$ to $1.37e^{-2}$	1.00	0.99 to 1.01	1.10	0.86 to 1.41	1.00	0.98 to 1.01	1.15	0.68 to 1.94	1.00	0.99 to 1.02
Q4	$6.51e^{-3}$	$4.62e^{-3}$ to $9.18e^{-3}$	0.99	0.98 to 1.00	1.50	1.13 to 1.99	1.00	0.99 to 1.02	1.63	0.89 to 2.99	1.02	1.01 to 1.04
Q5 - least deprived	$6.06e^{-3}$	$4.86e^{-3}$ to $7.54e^{-3}$	1.00	0.99 to 1.00	1.00	0.84 to 1.21	1.00	1.00 to 1.01	1.24	0.85 to 1.82	1.00	0.99 to 1.01
**Admissions 1 month lag**	IR	95% CI	IRR	95% CI	IRR	95% CI	IRR	95% CI	IRR	95% CI	IRR	95% CI
Q1 - most deprived	$1.45e^{-2}$	$1.23e^{-2}$ to $1.72e^{-2}$	1.01	1.01 to 1.02	0.94	0.86 to 1.03	0.99	0.98 to 0.99	0.60	0.49 to 0.73	0.99	0.98 to 0.99
Q2	$1.20e^{-2}$	$9.58e^{-3}$ to $1.51e^{-2}$	1.00	1.00 to 1.01	1.12	0.93 to 1.34	0.99	0.98 to 1.00	0.79	0.53 to 1.16	1.00	0.99 to 1.01
Q3	$9.77e^{-3}$	$6.99e^{-3}$ to $1.37e^{-2}$	1.00	0.99 to 1.01	1.00	0.78 to 1.27	1.00	0.99 to 1.01	0.99	0.60 to 1.65	1.00	0.99 to 1.02
Q4	$6.84e^{-3}$	$4.83e^{-3}$ to $9.67e^{-3}$	0.99	0.98 to 1.00	1.53	1.16 to 2.02	1.00	0.99 to 1.01	1.66	0.91 to 3.00	1.02	1.01 to 1.04
Q5 - least deprived	$6.12e^{-3}$	$4.90e^{-3}$ to $7.64e^{-3}$	1.00	0.99 to 1.00	1.03	0.86 to 1.23	1.01	1.00 to 1.01	1.27	0.87 to 1.85	1.00	0.99 to 1.01
**Admissions 2 month lag**	IR	95% CI	IRR	95% CI	IRR	95% CI	IRR	95% CI	IRR	95% CI	IRR	95% CI
Q1 - most deprived	$1.46e^{-2}$	$1.24e^{-2}$ to $1.73e^{-2}$	1.01	1.01 to 1.01	0.94	0.86 to 1.03	0.99	0.98 to 0.99	0.60	0.50 to 0.73	0.99	0.98 to 0.99
Q2	$1.20e^{-2}$	$9.58e^{-3}$ to $1.51e^{-2}$	1.00	1.00 to 1.01	1.11	0.92 to 1.33	0.99	0.98 to 1.00	0.78	0.54 to 1.15	1.00	0.99 to 1.01
Q3	$1.00e^{-2}$	$1.00e^{-2}$ to $1.40e^{-2}$	1.00	0.99 to 1.01	1.07	0.84 to 1.37	1.00	0.99 to 1.01	1.12	0.68 to 1.84	1.00	0.99 to 1.02
Q4	$6.80e^{-3}$	$4.81e^{-3}$ to $9.60e^{-3}$	0.99	0.98 to 1.00	1.57	1.19 to 2.07	1.00	0.98 to 1.01	1.70	0.95 to 3.05	1.02	1.00 to 1.03
Q5 - least deprived	$6.21e^{-3}$	$4.98e^{-3}$ to $7.75e^{-3}$	0.99	0.99 to 1.00	1.09	0.91 to 1.31	1.00	1.00 to 1.01	1.38	0.95 to 1.99	1.00	0.99 to 1.01
**Admissions 3 month lag**	IR	95% CI	IRR	95% CI	IRR	95% CI	IRR	95% CI	IRR	95% CI	IRR	95% CI
Q1 - most deprived	$1.47e^{-2}$	$1.24e^{-2}$ to $1.74e^{-2}$	1.01	1.01 to 1.01	0.94	0.86 to 1.02	0.99	0.98 to 0.99	0.60	0.50 to 0.73	0.99	0.98 to 0.99
Q2	$1.18e^{-2}$	$9.40e^{-3}$ to $1.48e^{-2}$	1.01	1.00 to 1.01	1.03	0.86 to 1.24	0.99	0.98 to 1.00	0.72	0.50 to 1.05	1.00	0.99 to 1.01
Q3	$1.03e^{-2}$	$7.38e^{-3}$ to $1.43e^{-2}$	1.00	0.99 to 1.00	1.18	0.92 to 1.51	1.00	0.99 to 1.01	1.25	0.76 to 2.05	1.01	0.99 to 1.02
Q4	$6.43e^{-3}$	$4.55e^{-3}$ to $9.07e^{-3}$	0.99	0.98 to 1.00	1.34	1.02 to 1.77	1.00	0.98 to 1.01	1.36	0.77 to 2.39	1.01	1.00 to 1.03
Q5 - least deprived	$6.15e^{-3}$	$4.93e^{-3}$ to $7.67e^{-3}$	1.00	0.99 to 1.00	1.07	0.89 to 1.28	1.00	0.99 to 1.01	1.35	0.94 to 1.94	1.00	0.99 to 1.01
**Defined Daily Doses (DDDs)**	DDDs	95% CI	ΔDDDs	95% CI	ΔDDDs	95% CI	ΔDDDs	95% CI	ΔDDDs	95% CI	ΔDDDs	95% CI
Q1 - most deprived	24.46	21.29 to 27.62	-0.07	-0.10 to -0.04	0.25	-0.50 to 1.01	0.08	0.04 to 0.12	6.23	4.59 to 7.86	0.14	0.10 to 0.18
Q2	29.64	25.30 to 33.99	0.05	-0.03 to 0.12	-1.83	-3.80 to 0.15	-0.05	-0.15 to 0.05	2.09	-2.19 to 6.37	0.03	-0.07 to 0.14
Q3	35.64	31.29 to 39.98	-0.06	-0.18 to 0.06	1.11	-2.07 to 4.28	-0.03	-0.20 to 0.13	7.32	0.27 to 14.37	0.15	-0.02 to 0.32
Q4	37.99	34.12 to 41.87	-0.05	-0.14 to 0.03	0.12	-2.06 to 2.31	0.06	-0.05 to 0.17	7.62	2.87 to 12.38	0.09	-0.03 to 0.21
Q5 - least deprived	30.20	28.03 to 32.37	0.02	-0.02 to 0.06	-1.15	-2.24 to -0.05	$9.05e^{-4}$	-0.06 to 0.06	4.21	1.83 to 6.60	0.01	-0.05 to 0.06

Supplemental file 4 Health board clustering sensitivity analysis

	Intercept		Monthly change		Reduction step change		Reduction change in slope		Abolition step change		Abolition change in slope	
**Cost**	£s	95% CI	Δ£s	95% CI	Δ£s	95% CI	Δ£s	95% CI	Δ£s	95% CI	Δ£s	95% CI
Q1 - most deprived	17.49	14.36 to 20.62	0.07	0.05 to 0.09	0.37	-0.24 to 0.98	-0.06	-0.09 to -0.03	1.02	-0.32 to 2.36	-0.02	-0.05 to 0.02
Q2	22.94	17.26 to 17.26	0.21	0.14 to 0.28	-0.88	-2.67 to 0.90	-0.21	-0.30 to -0.12	-2.63	-6.59 to 1.32	-0.18	-0.28 to -0.09
Q3	22.97	19.33 to 26.60	0.20	0.10 to 0.29	0.83	-1.55 to 3.20	-0.25	-0.38 to -0.13	-1.67	-6.95 to 3.62	-0.08	-0.21 to 0.04
Q4	24.86	21.27 to 28.46	0.22	0.14 to 0.30	0.14	-1.92 to 2.21	-0.24	-0.35 to -0.13	-2.63	-7.26 to 2.01	-0.19	-0.30 to -0.08
Q5 - least deprived	20.76	17.57 to 23.94	0.20	0.17 to 0.24	-0.78	-1.72 to 0.16	-0.18	-0.23 to -0.14	-2.67	-4.76 to -0.58	-0.18	-0.23 to -0.13

95% CI; 95% confidence interval; DDDs; Defined daily doses per 100 patients per practice per month, ΔDDDs; change in DDDs, IR; Incidence rate per 100 patients per practice per month, IRR; incidence rate ratio, £s; gross ingredient cost of medicines before any discount per 100 patients per practice per month, Δ£s; change in £s, Q1-5; quintiles of the Scottish Index of Multiple Deprivation from most to least deprived

For peer review only

Supplemental file 5 Health board clustering sensitivity analysis

Table S5 - Quadratic time effects

	Intercept		Monthly change		Quadratic monthly change	
**Admissions**	IRR	95% CI	ΔIRR	95% CI	ΔIRR	95% CI
Intervention						
Linear secular and intervention time effects	0.010	0.009 to 0.011	1.005	1.003 to 1.007	-	-
plus quadratic time effect	0.010	0.009 to 0.011	1.008	1.004 to 1.012	1.000	1.000 to 1.000
plus quadratic intervention effects	0.010	0.009 to 0.011	1.005	1.003 to 1.007	-	-
Age-based counterfactual						
Linear secular and intervention time effects	0.066	0.064 to 0.069	1.003	1.002 to 1.004	-	-
plus quadratic time effect	0.065	0.062 to 0.068	1.008	1.006 to 1.010	1.000	1.000 to 1.000
plus quadratic intervention effects	0.067	0.064 to 0.070	1.003	1.002 to 1.004	-	-
Condition-based counterfactual						
Linear secular and intervention time effects	0.005	0.004 to 0.005	1.003	1.000 to 1.006	-	-
plus quadratic time effect	0.005	0.004 to 0.005	1.004	0.998 to 1.011	1.000	1.000 to 1.000
plus quadratic intervention effects	0.005	0.004 to 0.005	1.003	1.000 to 1.006	-	-
**Defined Daily Doses (DDDs)**	DDDs	95% CI	ΔDDDs	95% CI	ΔDDDs	95% CI
Intervention						
Linear secular and intervention time effects	29.648	28.711 to 30.585	-0.036	-0.055 to -0.017	-	-
plus quadratic time effect	29.504	28.549 to 30.459	-0.007	-0.049 to 0.036	-0.001	-0.002 to <0.001
plus quadratic intervention effects	29.662	28.725 to 30.600	-0.037	-0.056 to -0.017	-	-
Age-based counterfactual						
Linear secular and intervention time effects	100.885	98.067 to 103.703	-0.052	-0.113 to 0.009	-	-
plus quadratic time effect	100.370	97.508 to 103.232	0.068	-0.064 to 0.200	-0.004	-0.008 to <0.001
plus quadratic intervention effects	101.024	98.203 to 103.845	-0.053	-0.115 to 0.009	-	-
Condition-based counterfactual						
Linear secular and intervention time effects	55.612	53.698 to 57.527	0.143	0.104 to 0.183	-	-
plus quadratic time effect	55.119	53.169 to 57.069	0.248	0.160 to 0.336	-0.003	-0.006 to -0.001
plus quadratic intervention effects	55.645	53.729 to 57.561	0.141	0.101 to 0.181	-	-
**Cost**	£	95% CI	Δ£	95% CI	Δ£	95% CI
Intervention						
Linear secular and intervention time effects	19.364	18.535 to 20.194	0.165	0.148 to 0.182	-	-
plus quadratic time effect	19.558	18.714 to 20.402	0.124	0.088 to 0.161	0.001	<0.001 to 0.002
plus quadratic intervention effects	19.350	18.520 to 20.180	0.165	0.149 to 0.182	-	-
Age-based counterfactual						
Linear secular and intervention time effects	69.920	67.349 to 72.492	0.621	0.563 to 0.679	-	-
plus quadratic time effect	70.090	67.480 to 72.700	0.579	0.455 to 0.704	0.001	-0.002 to 0.005
plus quadratic intervention effects	69.998	67.422 to 72.574	0.621	0.561 to 0.680	-	-
Condition-based counterfactual						
Linear secular and intervention time effects	26.345	25.349 to 27.341	0.149	0.128 to 0.170	-	-
plus quadratic time effect	25.960	24.945 to 26.976	0.229	0.182 to 0.275	-0.003	-0.004 to -0.001
plus quadratic intervention effects	26.291	26.291 to 27.287	0.150	0.129 to 0.171	-	-

95% CI; 95% confidence interval, DDDs; Defined daily doses per 100 patients per practice per month, ΔDDDs; change in DDDs, IR; Incidence rate per 100 patients per practice per month, IRR; incidence rate ratio, £s; gross ingredient cost of medicines before any discount per 100 patients per practice per month, Δ£s; change in £s

For peer review only

Supplemental file 5 Health board clustering sensitivity analysis

Table S5 (continued) - Quadratic time effects

	Reduction step change		Reduction change in slope		Reduction change in slope (quadratic)	
**Admissions**	ΔIRR	95% CI	ΔIRR	95% CI	ΔIRR	95% CI
Intervention						
Linear secular and intervention time effects	1.032	0.982 to 1.084	0.992	0.989 to 0.994	-	-
plus quadratic time effect	1.030	0.980 to 1.083	0.999	0.990 to 1.007	-	-
plus quadratic intervention effects	1.082	1.018 to 1.150	0.983	0.977 to 0.990	1.000	1.000 to 1.000
Age-based counterfactual						
Linear secular and intervention time effects	1.033	1.007 to 1.059	0.995	0.994 to 0.997	-	-
plus quadratic time effect	1.030	1.004 to 1.057	1.005	1.001 to 1.009	-	-
plus quadratic intervention effects	1.069	1.037 to 1.102	0.989	0.986 to 0.992	1.000	1.000 to 1.000
Condition-based counterfactual						
Linear secular and intervention time effects	0.952	0.880 to 1.029	1.002	0.998 to 1.005	-	-
plus quadratic time effect	0.951	0.879 to 1.028	1.005	0.993 to 1.018	-	-
plus quadratic intervention effects	0.947	0.860 to 1.043	1.002	0.992 to 1.012	1.000	1.000 to 1.000
**Defined Daily Doses (DDDs)**	ΔDDDs	95% CI	ΔDDDs	95% CI	ΔDDDs	95% CI
Intervention						
Linear secular and intervention time effects	-0.113	-0.612 to 0.386	0.042	0.016 to 0.068	-	-
plus quadratic time effect	-0.107	-0.606 to 0.393	0.107	0.020 to 0.194	-	-
plus quadratic intervention effects	-0.230	-0.815 to 0.355	0.065	-0.002 to 0.132	-0.001	-0.002 to 0.001
Age-based counterfactual						
Linear secular and intervention time effects	5.065	3.614 to 6.515	0.681	0.594 to 0.768	-	-
plus quadratic time effect	5.144	3.692 to 6.597	0.957	0.674 to 1.240	-	-
plus quadratic intervention effects	4.749	3.158 to 6.340	0.759	0.548 to 0.970	-0.003	-0.008 to 0.002
Condition-based counterfactual						
Linear secular and intervention time effects	-1.876	-2.897 to -0.855	-0.054	-0.108 to 0.001	-	-
plus quadratic time effect	-1.839	-2.860 to -0.817	0.179	-0.003 to 0.362	-	-
plus quadratic intervention effects	-2.226	-3.401 to -1.052	0.025	-0.114 to 0.164	-0.002	-0.006 to 0.001
**Cost**	Δ£	95% CI	Δ£	95% CI	Δ£	95% CI
Intervention						
Linear secular and intervention time effects	0.230	-0.195 to 0.656	-0.150	-0.173 to -0.128	-	-
plus quadratic time effect	0.217	-0.208 to 0.642	-0.241	-0.316 to -0.165	-	-
plus quadratic intervention effects	0.305	-0.186 to 0.796	-0.165	-0.223 to -0.107	$4.35e^{-4}$	-0.001 to 0.002
Age-based counterfactual						
Linear secular and intervention time effects	4.740	3.425 to 6.055	0.034	-0.051 to 0.119	-	-
plus quadratic time effect	4.707	3.389 to 6.025	-0.063	-0.333 to 0.206	-	-
plus quadratic intervention effects	4.607	3.193 to 6.020	0.070	-0.131 to 0.271	-0.002	-0.006 to 0.003
Condition-based counterfactual						
Linear secular and intervention time effects	-3.159	-3.703 to -2.616	-0.010	-0.039 to 0.018	-	-
plus quadratic time effect	-3.137	-3.681 to -2.594	0.166	0.071 to 0.262	-	-
plus quadratic intervention effects	-2.701	-3.333 to -2.069	-0.105	-0.178 to -0.031	0.003	0.001 to 0.005

95% CI; 95% confidence interval, DDDs; Defined daily doses per 100 patients per practice per month, ΔDDDs; change in DDDs, IR; Incidence rate per 100 patients per practice per month, IRR; incidence rate ratio, £s; gross ingredient cost of medicines before any discount per 100 patients per practice per month, Δ£s; change in £

For peer review only

Supplemental file 5 Health board clustering sensitivity analysis

Table S5 (continued) - Quadratic time effects

	Abolition step change		Abolition change in slope		Abolition change in slope (quadratic)	
**Admissions**	ΔIRR	95% C1	ΔIRR	95% CI	ΔIRR	95% CI
Intervention						
Linear secular and intervention time effects	0.807	0.725 to 0.899	0.995	0.993 to 0.998	-	-
plus quadratic time effect	1.033	0.755 to 1.413	1.009	0.993 to 1.026	-	-
plus quadratic intervention effects	0.750	0.669 to 0.842	1.008	1.000 to 1.016	1.000	0.999 to 1.000
Age-based counterfactual						
Linear secular and intervention time effects	0.948	0.897 to 1.001	0.996	0.995 to 0.998	-	-
plus quadratic time effect	1.356	1.160 to 1.585	1.016	1.008 to 1.024	-	-
plus quadratic intervention effects	0.890	0.840 to 0.943	1.007	1.004 to 1.011	1.000	1.000 to 1.000
Condition-based counterfactual						
Linear secular and intervention time effects	1.015	0.858 to 1.200	1.001	0.997 to 1.005	-	-
plus quadratic time effect	1.171	0.730 to 1.880	1.009	0.985 to 1.034	-	-
plus quadratic intervention effects	0.970	0.812 to 1.159	1.009	0.998 to 1.020	1.000	0.999 to 1.000
**Defined Daily Doses (DDDs)**	ΔDDDs	95% CI	ΔDDDs	95% CI	ΔDDDs	95% CI
Intervention						
Linear secular and intervention time effects	6.034	4.935 to 7.133	0.105	0.078 to 0.132	-	-
plus quadratic time effect	8.441	5.179 to 11.702	0.235	0.067 to 0.403	-	-
plus quadratic intervention effects	5.914	4.754 to 7.074	0.131	0.056 to 0.207	-0.001	-0.003 to 0.001
Age-based counterfactual						
Linear secular and intervention time effects	38.373	34.843 to 41.903	0.336	0.250 to 0.421	-	-
plus quadratic time effect	48.598	38.035 to 59.161	0.889	0.344 to 1.433	-	-
plus quadratic intervention effects	35.666	31.986 to 39.347	0.931	0.702 to 1.159	-0.019	-0.025 to -0.012
Condition-based counterfactual						
Linear secular and intervention time effects	6.024	3.729 to 8.319	0.146	0.090 to 0.203	-	-
plus quadratic time effect	14.645	7.802 to 21.488	0.612	0.259 to 0.965	-	-
plus quadratic intervention effects	6.369	3.954 to 8.784	0.101	-0.054 to 0.256	0.002	-0.003 to 0.006
**Cost**	Δ£	95% CI	Δ£	95% CI	Δ£	95% CI
Intervention						
Linear secular and intervention time effects	-0.965	-1.916 to -0.013	-0.100	-0.124 to -0.077	-	-
plus quadratic time effect	-4.315	-7.148 to -1.482	-0.281	-0.427 to -0.135	-	-
plus quadratic intervention effects	-0.773	-1.774 to 0.229	-0.141	-0.205 to -0.076	0.001	-0.001 to 0.003
Age-based counterfactual						
Linear secular and intervention time effects	14.242	10.857 to 17.627	-0.356	-0.438 to -0.275	-	-
plus quadratic time effect	10.647	0.585 to 20.709	-0.551	-1.069 to -0.032	-	-
plus quadratic intervention effects	12.558	9.036 to 16.080	0.024	-0.191 to 0.238	-0.012	-0.018 to -0.006
Condition-based counterfactual						
Linear secular and intervention time effects	1.997	0.791 to 3.203	-0.115	-0.144 to -0.085	-	-
plus quadratic time effect	8.544	4.956 to 12.131	0.239	0.054 to 0.424	-	-
plus quadratic intervention effects	2.256	0.98 to 3.527	-0.184	-0.266 to -0.102	0.002	<0.001 to 0.004

95% CI; 95% confidence interval, DDDs; Defined daily doses per 100 patients per practice per month, ΔDDDs; change in DDDs, IR; Incidence rate per 100 patients per practice per month, IRR; incidence rate ratio, £s; gross ingredient cost of medicines before any discount per 100 patients per practice per month, Δ£s; change in £s

For peer review only

Supplemental file 5 Health board clustering sensitivity analysis

Figure S5 – Plots comparing the linear and quadratic time effects within the interrupted time series analysis

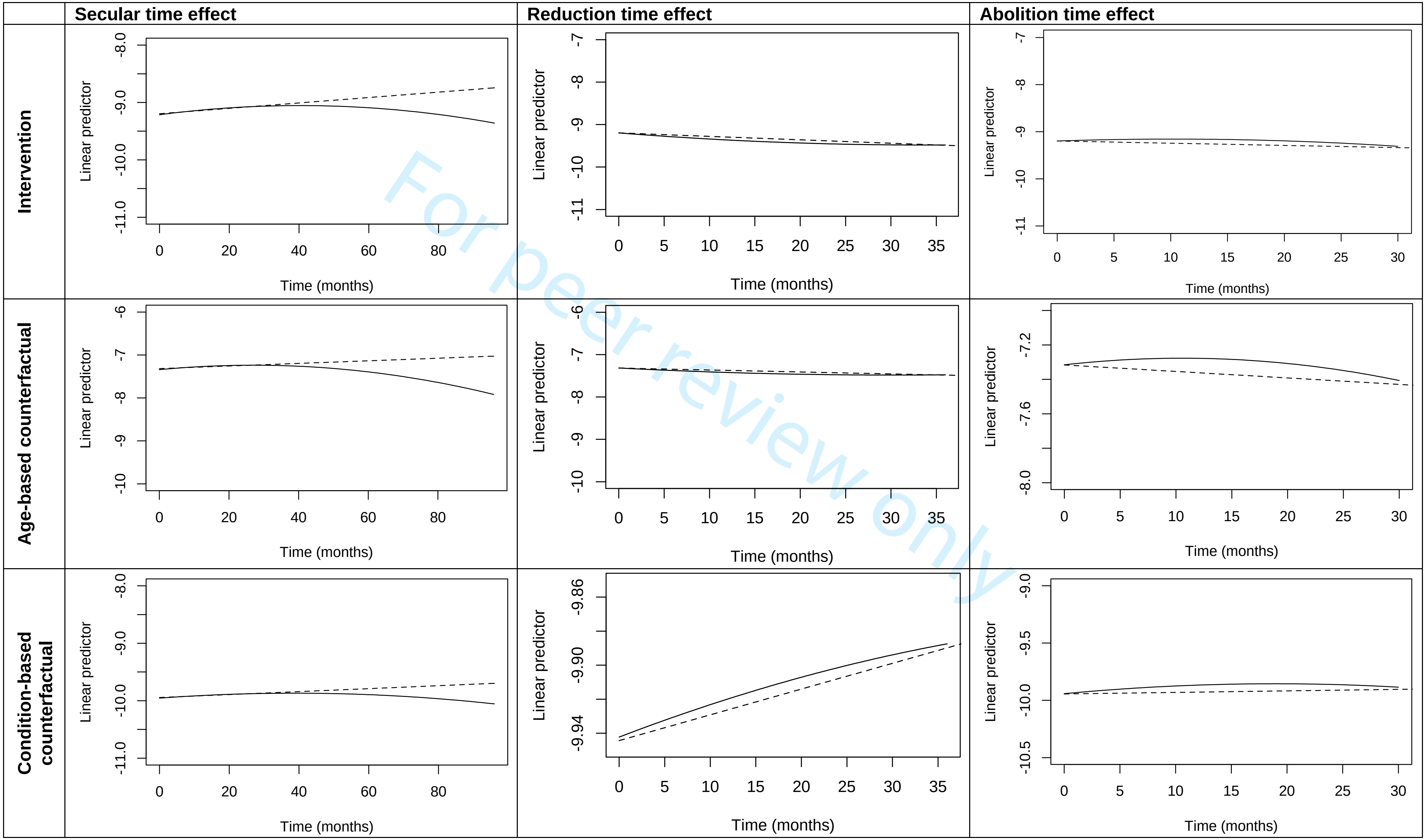

For peer review only

STROBE 2007 (v4) Statement—Checklist of items that should be included in reports of *cohort studies*

Manuscript title: Interrupted time series evaluation of the impact of abolishing prescription fees in Scotland on hospital admissions and prescribed medicines
Authors: Andrew James Williams, William Henley and John Frank

Section/Topic	Item #	Recommendation	Reported on page #
**Title and abstract**	1	(*a*) Indicate the study's design with a commonly used term in the title or the abstract	1
		(*b*) Provide in the abstract an informative and balanced summary of what was done and what was found	2
**Introduction**			
Background/rationale	2	Explain the scientific background and rationale for the investigation being reported	5-6
Objectives	3	State specific objectives, including any prespecified hypotheses	6
**Methods**			
Study design	4	Present key elements of study design early in the paper	6-10
Setting	5	Describe the setting, locations, and relevant dates, including periods of recruitment, exposure, follow-up, and data collection	6-7
Participants	6	(*a*) Give the eligibility criteria, and the sources and methods of selection of participants. Describe methods of follow-up	6-9
		(*b*) For matched studies, give matching criteria and number of exposed and unexposed	N/A
Variables	7	Clearly define all outcomes, exposures, predictors, potential confounders, and effect modifiers. Give diagnostic criteria, if applicable	6-10
Data sources/ measurement	8*	For each variable of interest, give sources of data and details of methods of assessment (measurement). Describe comparability of assessment methods if there is more than one group	6-10
Bias	9	Describe any efforts to address potential sources of bias	6-18
Study size	10	Explain how the study size was arrived at	10-11
Quantitative variables	11	Explain how quantitative variables were handled in the analyses. If applicable, describe which groupings were chosen and why	6-10
Statistical methods	12	(*a*) Describe all statistical methods, including those used to control for confounding	9-10
		(*b*) Describe any methods used to examine subgroups and interactions	9-10
		(*c*) Explain how missing data were addressed	NA
		(*d*) If applicable, explain how loss to follow-up was addressed	NA

For peer review only

		(*e*) Describe any sensitivity analyses	17-18
**Results**			
Participants	13*	(a) Report numbers of individuals at each stage of study—eg numbers potentially eligible, examined for eligibility, confirmed eligible, included in the study, completing follow-up, and analysed	10-11
		(b) Give reasons for non-participation at each stage	NA
		(c) Consider use of a flow diagram	NA
Descriptive data	14*	(a) Give characteristics of study participants (eg demographic, clinical, social) and information on exposures and potential confounders	Table 2 page 11
		(b) Indicate number of participants with missing data for each variable of interest	NA – complete case analysis
		(c) Summarise follow-up time (eg, average and total amount)	6-7
Outcome data	15*	Report numbers of outcome events or summary measures over time	Tables 3 and 4
Main results	16	(*a*) Give unadjusted estimates and, if applicable, confounder-adjusted estimates and their precision (eg, 95% confidence interval). Make clear which confounders were adjusted for and why they were included	Tables 3 and 4
		(*b*) Report category boundaries when continuous variables were categorized	NA
		(*c*) If relevant, consider translating estimates of relative risk into absolute risk for a meaningful time period	NA
Other analyses	17	Report other analyses done—eg analyses of subgroups and interactions, and sensitivity analyses	14-18
**Discussion**			
Key results	18	Summarise key results with reference to study objectives	18
**Limitations**			
Interpretation	20	Give a cautious overall interpretation of results considering objectives, limitations, multiplicity of analyses, results from similar studies, and other relevant evidence	18-20
Generalisability	21	Discuss the generalisability (external validity) of the study results	18-20
**Other information**			
Funding	22	Give the source of funding and the role of the funders for the present study and, if applicable, for the original study on which the present article is based	3

*Give information separately for cases and controls in case-control studies and, if applicable, for exposed and unexposed groups in cohort and cross-sectional studies.

Note: An Explanation and Elaboration article discusses each checklist item and gives methodological background and published examples of transparent reporting. The STROBE checklist is best used in conjunction with this article (freely available on the Web sites of PLoS Medicine at http://www.plosmedicine.org/, Annals of Internal Medicine at http://www.annals.org/, and Epidemiology at http://www.epidem.com/). Information on the STROBE Initiative is available at www.strobe-statement.org.

For peer review only
